# Supplementary material for: An immunoevasive strategy through clinically-relevant pan-cancer genomic and transcriptomic alterations of JAK-STAT signaling components
Source: Mol Med. 2019 Nov 4;25:46. doi: 10.1186/s10020-019-0114-1 (PMC6829980; doi:10.1186/s10020-019-0114-1)
Supplement: Supplementary file 1 — Additional file 1. List of 133 JAK-STAT pathway genes. [file 10020_2019_114_MOESM1_ESM.docx]

Additional file 1: Table S1 List of 133 JAK-STAT pathway genes.

|  |  |
| --- | --- |
| **Gene Symbol** | **Description** |
| AOX1 | aldehyde oxidase 1 |
| BCL2 | BCL2, apoptosis regulator |
| BCL2L1 | BCL2 like 1 |
| CDKN1A | cyclin dependent kinase inhibitor 1A |
| CISH | cytokine inducible SH2 containing protein |
| CNTF | ciliary neurotrophic factor |
| CNTFR | ciliary neurotrophic factor receptor |
| CRLF2 | cytokine receptor like factor 2 |
| CSF2 | colony stimulating factor 2 |
| CSF2RA | colony stimulating factor 2 receptor alpha subunit |
| CSF2RB | colony stimulating factor 2 receptor beta common subunit |
| CSF3 | colony stimulating factor 3 |
| CSF3R | colony stimulating factor 3 receptor |
| CSH1 | chorionic somatomammotropin hormone 1 |
| CSH2 | chorionic somatomammotropin hormone 2 |
| CTF1 | cardiotrophin 1 |
| EPO | erythropoietin |
| EPOR | erythropoietin receptor |
| FHL1 | four and a half LIM domains 1 |
| GFAP | glial fibrillary acidic protein |
| GH1 | growth hormone 1 |
| GH2 | growth hormone 2 |
| GHR | growth hormone receptor |
| IFNA1 | interferon alpha 1 |
| IFNA10 | interferon alpha 10 |
| IFNA13 | interferon alpha 13 |
| IFNA14 | interferon alpha 14 |
| IFNA16 | interferon alpha 16 |
| IFNA17 | interferon alpha 17 |
| IFNA2 | interferon alpha 2 |
| IFNA21 | interferon alpha 21 |
| IFNA4 | interferon alpha 4 |
| IFNA5 | interferon alpha 5 |
| IFNA6 | interferon alpha 6 |
| IFNA7 | interferon alpha 7 |
| IFNA8 | interferon alpha 8 |
| IFNAR1 | interferon alpha and beta receptor subunit 1 |
| IFNAR2 | interferon alpha and beta receptor subunit 2 |
| IFNB1 | interferon beta 1 |
| IFNE | interferon epsilon |
| IFNG | interferon gamma |
| IFNGR1 | interferon gamma receptor 1 |
| IFNGR2 | interferon gamma receptor 2 |
| IFNK | interferon kappa |
| IFNL1 | interferon lambda 1 |
| IFNL2 | interferon lambda 2 |
| IFNL3 | interferon lambda 3 |
| IFNLR1 | interferon lambda receptor 1 |
| IFNW1 | interferon omega 1 |
| IL10 | interleukin 10 |
| IL10RA | interleukin 10 receptor subunit alpha |
| IL10RB | interleukin 10 receptor subunit beta |
| IL11 | interleukin 11 |
| IL11RA | interleukin 11 receptor subunit alpha |
| IL12A | interleukin 12A |
| IL12B | interleukin 12B |
| IL12RB1 | interleukin 12 receptor subunit beta 1 |
| IL12RB2 | interleukin 12 receptor subunit beta 2 |
| IL13 | interleukin 13 |
| IL13RA1 | interleukin 13 receptor subunit alpha 1 |
| IL13RA2 | interleukin 13 receptor subunit alpha 2 |
| IL15 | interleukin 15 |
| IL15RA | interleukin 15 receptor subunit alpha |
| IL17D | interleukin 17D |
| IL19 | interleukin 19 |
| IL2 | interleukin 2 |
| IL20 | interleukin 20 |
| IL20RA | interleukin 20 receptor subunit alpha |
| IL20RB | interleukin 20 receptor subunit beta |
| IL21 | interleukin 21 |
| IL21R | interleukin 21 receptor |
| IL22 | interleukin 22 |
| IL22RA1 | interleukin 22 receptor subunit alpha 1 |
| IL22RA2 | interleukin 22 receptor subunit alpha 2 |
| IL23A | interleukin 23 subunit alpha |
| IL23R | interleukin 23 receptor |
| IL24 | interleukin 24 |
| IL27RA | interleukin 27 receptor subunit alpha |
| IL2RA | interleukin 2 receptor subunit alpha |
| IL2RB | interleukin 2 receptor subunit beta |
| IL2RG | interleukin 2 receptor subunit gamma |
| IL3 | interleukin 3 |
| IL3RA | interleukin 3 receptor subunit alpha |
| IL4 | interleukin 4 |
| IL4R | interleukin 4 receptor |
| IL5 | interleukin 5 |
| IL5RA | interleukin 5 receptor subunit alpha |
| IL6 | interleukin 6 |
| IL6R | interleukin 6 receptor |
| IL6ST | interleukin 6 signal transducer |
| IL7 | interleukin 7 |
| IL7R | interleukin 7 receptor |
| IL9 | interleukin 9 |
| IL9R | interleukin 9 receptor |
| IRF9 | interferon regulatory factor 9 |
| JAK1 | Janus kinase 1 |
| JAK2 | Janus kinase 2 |
| JAK3 | Janus kinase 3 |
| LIF | LIF, interleukin 6 family cytokine |
| LIFR | LIF receptor alpha |
| MCL1 | MCL1, BCL2 family apoptosis regulator |
| MPL | MPL proto-oncogene, thrombopoietin receptor |
| OSM | oncostatin M |
| OSMR | oncostatin M receptor |
| PIAS1 | protein inhibitor of activated STAT 1 |
| PIAS2 | protein inhibitor of activated STAT 2 |
| PIAS3 | protein inhibitor of activated STAT 3 |
| PIAS4 | protein inhibitor of activated STAT 4 |
| PIM1 | Pim-1 proto-oncogene, serine/threonine kinase |
| PRL | prolactin |
| PRLR | prolactin receptor |
| PTPN11 | protein tyrosine phosphatase, non-receptor type 11 |
| PTPN2 | protein tyrosine phosphatase, non-receptor type 2 |
| PTPN6 | protein tyrosine phosphatase, non-receptor type 6 |
| SOCS1 | suppressor of cytokine signaling 1 |
| SOCS2 | suppressor of cytokine signaling 2 |
| SOCS3 | suppressor of cytokine signaling 3 |
| SOCS4 | suppressor of cytokine signaling 4 |
| SOCS5 | suppressor of cytokine signaling 5 |
| SOCS6 | suppressor of cytokine signaling 6 |
| SOCS7 | suppressor of cytokine signaling 7 |
| STAM | signal transducing adaptor molecule |
| STAM2 | signal transducing adaptor molecule 2 |
| STAT1 | signal transducer and activator of transcription 1 |
| STAT2 | signal transducer and activator of transcription 2 |
| STAT3 | signal transducer and activator of transcription 3 |
| STAT4 | signal transducer and activator of transcription 4 |
| STAT5A | signal transducer and activator of transcription 5A |
| STAT5B | signal transducer and activator of transcription 5B |
| STAT6 | signal transducer and activator of transcription 6 |
| THPO | thrombopoietin |
| TSLP | thymic stromal lymphopoietin |
| TYK2 | tyrosine kinase 2 |
