# Supplementary material for: An immunoevasive strategy through clinically-relevant pan-cancer genomic and transcriptomic alterations of JAK-STAT signaling components
Source: Mol Med. 2019 Nov 4;25:46. doi: 10.1186/s10020-019-0114-1 (PMC6829980; doi:10.1186/s10020-019-0114-1)
Supplement: Supplementary file 2 — Additional file 2. Fraction of somatic copy number alterations above 20% of samples within each cancer type. [file 10020_2019_114_MOESM2_ESM.docx]

**Additional file 2. Fraction of somatic copy number alterations above 20% of samples within each cancer type.**

|  |  |  |  |  |
| --- | --- | --- | --- | --- |
| **Gene Symbol** | **Alteration type** | **Sample number** | **Fraction altered** | **Cancer abbreviation** |
| AOX1 | gain | 52 | 0.282608696 | ESCA |
| AOX1 | gain | 138 | 0.26744186 | LUAD |
| AOX1 | gain | 138 | 0.275449102 | LUSC |
| AOX1 | gain | 127 | 0.2032 | STES |
| BCL2 | gain | 16 | 0.242424242 | KICH |
| BCL2L1 | gain | 263 | 0.644607843 | BLCA |
| BCL2L1 | gain | 454 | 0.42037037 | BRCA |
| BCL2L1 | gain | 135 | 0.457627119 | CESC |
| BCL2L1 | gain | 13 | 0.361111111 | CHOL |
| BCL2L1 | gain | 323 | 0.716186253 | COAD |
| BCL2L1 | gain | 121 | 0.657608696 | ESCA |
| BCL2L1 | gain | 225 | 0.389948007 | GBM |
| BCL2L1 | gain | 269 | 0.246788991 | GBMLGG |
| BCL2L1 | gain | 231 | 0.442528736 | HNSC |
| BCL2L1 | gain | 22 | 0.333333333 | KICH |
| BCL2L1 | gain | 241 | 0.27324263 | KIPAN |
| BCL2L1 | gain | 118 | 0.223484848 | KIRC |
| BCL2L1 | gain | 101 | 0.350694444 | KIRP |
| BCL2L1 | gain | 113 | 0.305405405 | LIHC |
| BCL2L1 | gain | 230 | 0.445736434 | LUAD |
| BCL2L1 | gain | 306 | 0.610778443 | LUSC |
| BCL2L1 | gain | 41 | 0.222826087 | PAAD |
| BCL2L1 | gain | 105 | 0.408560311 | SARC |
| BCL2L1 | gain | 277 | 0.628117914 | STAD |
| BCL2L1 | gain | 398 | 0.6368 | STES |
| BCL2L1 | gain | 152 | 0.282003711 | UCEC |
| CDKN1A | gain | 93 | 0.227941176 | BLCA |
| CDKN1A | gain | 257 | 0.237962963 | BRCA |
| CDKN1A | gain | 68 | 0.230508475 | CESC |
| CDKN1A | gain | 9 | 0.25 | CHOL |
| CDKN1A | gain | 51 | 0.277173913 | ESCA |
| CDKN1A | gain | 138 | 0.372972973 | LIHC |
| CDKN1A | gain | 187 | 0.362403101 | LUAD |
| CDKN1A | gain | 128 | 0.255489022 | LUSC |
| CDKN1A | gain | 53 | 0.206225681 | SARC |
| CDKN1A | gain | 100 | 0.22675737 | STAD |
| CDKN1A | gain | 151 | 0.2416 | STES |
| CISH | gain | 99 | 0.242647059 | BLCA |
| CISH | gain | 72 | 0.25 | KIRP |
| CNTF | gain | 97 | 0.237745098 | BLCA |
| CNTF | gain | 45 | 0.244565217 | ESCA |
| CNTF | gain | 113 | 0.216475096 | HNSC |
| CNTF | gain | 18 | 0.272727273 | KICH |
| CNTF | gain | 122 | 0.236434109 | LUAD |
| CNTF | gain | 113 | 0.225548902 | LUSC |
| CNTFR | gain | 37 | 0.201086957 | ESCA |
| CNTFR | gain | 137 | 0.262452107 | HNSC |
| CNTFR | gain | 135 | 0.269461078 | LUSC |
| CNTFR | gain | 53 | 0.206225681 | SARC |
| CNTFR | gain | 125 | 0.2 | STES |
| CSF2 | gain | 11 | 0.305555556 | CHOL |
| CSF2 | gain | 358 | 0.405895692 | KIPAN |
| CSF2 | gain | 314 | 0.59469697 | KIRC |
| CSF2 | gain | 115 | 0.310810811 | LIHC |
| CSF2 | gain | 64 | 0.249027237 | SARC |
| CSF2RB | gain | 112 | 0.214559387 | HNSC |
| CSF2RB | gain | 20 | 0.303030303 | KICH |
| CSF2RB | gain | 233 | 0.46506986 | LUSC |
| CSF2RB | gain | 66 | 0.256809339 | SARC |
| CSF3 | gain | 159 | 0.389705882 | BLCA |
| CSF3 | gain | 310 | 0.287037037 | BRCA |
| CSF3 | gain | 8 | 0.222222222 | CHOL |
| CSF3 | gain | 111 | 0.246119734 | COAD |
| CSF3 | gain | 71 | 0.385869565 | ESCA |
| CSF3 | gain | 219 | 0.24829932 | KIPAN |
| CSF3 | gain | 189 | 0.65625 | KIRP |
| CSF3 | gain | 95 | 0.256756757 | LIHC |
| CSF3 | gain | 221 | 0.428294574 | LUAD |
| CSF3 | gain | 167 | 0.333333333 | LUSC |
| CSF3 | gain | 134 | 0.303854875 | STAD |
| CSF3 | gain | 205 | 0.328 | STES |
| CSF3R | gain | 84 | 0.284745763 | CESC |
| CSF3R | gain | 128 | 0.248062016 | LUAD |
| CSF3R | gain | 74 | 0.287937743 | SARC |
| CSH1 | gain | 195 | 0.477941176 | BLCA |
| CSH1 | gain | 426 | 0.394444444 | BRCA |
| CSH1 | gain | 70 | 0.237288136 | CESC |
| CSH1 | gain | 9 | 0.25 | CHOL |
| CSH1 | gain | 103 | 0.228381375 | COAD |
| CSH1 | gain | 67 | 0.364130435 | ESCA |
| CSH1 | gain | 111 | 0.212643678 | HNSC |
| CSH1 | gain | 239 | 0.270975057 | KIPAN |
| CSH1 | gain | 199 | 0.690972222 | KIRP |
| CSH1 | gain | 129 | 0.348648649 | LIHC |
| CSH1 | gain | 249 | 0.48255814 | LUAD |
| CSH1 | gain | 225 | 0.449101796 | LUSC |
| CSH1 | gain | 75 | 0.291828794 | SARC |
| CSH1 | gain | 97 | 0.219954649 | STAD |
| CSH1 | gain | 164 | 0.2624 | STES |
| CSH2 | gain | 195 | 0.477941176 | BLCA |
| CSH2 | gain | 426 | 0.394444444 | BRCA |
| CSH2 | gain | 70 | 0.237288136 | CESC |
| CSH2 | gain | 9 | 0.25 | CHOL |
| CSH2 | gain | 103 | 0.228381375 | COAD |
| CSH2 | gain | 67 | 0.364130435 | ESCA |
| CSH2 | gain | 111 | 0.212643678 | HNSC |
| CSH2 | gain | 239 | 0.270975057 | KIPAN |
| CSH2 | gain | 199 | 0.690972222 | KIRP |
| CSH2 | gain | 129 | 0.348648649 | LIHC |
| CSH2 | gain | 249 | 0.48255814 | LUAD |
| CSH2 | gain | 225 | 0.449101796 | LUSC |
| CSH2 | gain | 75 | 0.291828794 | SARC |
| CSH2 | gain | 97 | 0.219954649 | STAD |
| CSH2 | gain | 164 | 0.2624 | STES |
| CTF1 | gain | 88 | 0.215686275 | BLCA |
| CTF1 | gain | 549 | 0.508333333 | BRCA |
| CTF1 | gain | 125 | 0.277161863 | COAD |
| CTF1 | gain | 46 | 0.25 | ESCA |
| CTF1 | gain | 114 | 0.218390805 | HNSC |
| CTF1 | gain | 22 | 0.333333333 | KICH |
| CTF1 | gain | 284 | 0.321995465 | KIPAN |
| CTF1 | gain | 108 | 0.204545455 | KIRC |
| CTF1 | gain | 154 | 0.534722222 | KIRP |
| CTF1 | gain | 168 | 0.325581395 | LUAD |
| CTF1 | gain | 121 | 0.241516966 | LUSC |
| CTF1 | gain | 125 | 0.2 | STES |
| EPO | gain | 152 | 0.37254902 | BLCA |
| EPO | gain | 300 | 0.277777778 | BRCA |
| EPO | gain | 221 | 0.490022173 | COAD |
| EPO | gain | 103 | 0.559782609 | ESCA |
| EPO | gain | 472 | 0.818024263 | GBM |
| EPO | gain | 639 | 0.586238532 | GBMLGG |
| EPO | gain | 167 | 0.319923372 | HNSC |
| EPO | gain | 25 | 0.378787879 | KICH |
| EPO | gain | 373 | 0.422902494 | KIPAN |
| EPO | gain | 175 | 0.331439394 | KIRC |
| EPO | gain | 173 | 0.600694444 | KIRP |
| EPO | gain | 117 | 0.316216216 | LIHC |
| EPO | gain | 224 | 0.434108527 | LUAD |
| EPO | gain | 253 | 0.50499002 | LUSC |
| EPO | gain | 53 | 0.288043478 | PAAD |
| EPO | gain | 69 | 0.26848249 | SARC |
| EPO | gain | 197 | 0.446712018 | STAD |
| EPO | gain | 300 | 0.48 | STES |
| EPOR | gain | 237 | 0.410745234 | GBM |
| EPOR | gain | 354 | 0.324770642 | GBMLGG |
| EPOR | gain | 20 | 0.303030303 | KICH |
| EPOR | gain | 103 | 0.40077821 | SARC |
| FHL1 | gain | 62 | 0.336956522 | ESCA |
| FHL1 | gain | 129 | 0.247126437 | HNSC |
| FHL1 | gain | 92 | 0.319444444 | KIRP |
| FHL1 | gain | 74 | 0.2 | LIHC |
| FHL1 | gain | 136 | 0.263565891 | LUAD |
| FHL1 | gain | 110 | 0.219560878 | LUSC |
| FHL1 | gain | 144 | 0.2304 | STES |
| GFAP | gain | 148 | 0.362745098 | BLCA |
| GFAP | gain | 8 | 0.222222222 | CHOL |
| GFAP | gain | 100 | 0.22172949 | COAD |
| GFAP | gain | 61 | 0.331521739 | ESCA |
| GFAP | gain | 223 | 0.252834467 | KIPAN |
| GFAP | gain | 191 | 0.663194444 | KIRP |
| GFAP | gain | 103 | 0.278378378 | LIHC |
| GFAP | gain | 222 | 0.430232558 | LUAD |
| GFAP | gain | 170 | 0.339321357 | LUSC |
| GFAP | gain | 59 | 0.229571984 | SARC |
| GFAP | gain | 89 | 0.201814059 | STAD |
| GFAP | gain | 150 | 0.24 | STES |
| GH1 | gain | 195 | 0.477941176 | BLCA |
| GH1 | gain | 426 | 0.394444444 | BRCA |
| GH1 | gain | 70 | 0.237288136 | CESC |
| GH1 | gain | 9 | 0.25 | CHOL |
| GH1 | gain | 103 | 0.228381375 | COAD |
| GH1 | gain | 67 | 0.364130435 | ESCA |
| GH1 | gain | 111 | 0.212643678 | HNSC |
| GH1 | gain | 239 | 0.270975057 | KIPAN |
| GH1 | gain | 199 | 0.690972222 | KIRP |
| GH1 | gain | 129 | 0.348648649 | LIHC |
| GH1 | gain | 250 | 0.484496124 | LUAD |
| GH1 | gain | 225 | 0.449101796 | LUSC |
| GH1 | gain | 75 | 0.291828794 | SARC |
| GH1 | gain | 97 | 0.219954649 | STAD |
| GH1 | gain | 164 | 0.2624 | STES |
| GH2 | gain | 195 | 0.477941176 | BLCA |
| GH2 | gain | 426 | 0.394444444 | BRCA |
| GH2 | gain | 70 | 0.237288136 | CESC |
| GH2 | gain | 9 | 0.25 | CHOL |
| GH2 | gain | 103 | 0.228381375 | COAD |
| GH2 | gain | 67 | 0.364130435 | ESCA |
| GH2 | gain | 111 | 0.212643678 | HNSC |
| GH2 | gain | 239 | 0.270975057 | KIPAN |
| GH2 | gain | 199 | 0.690972222 | KIRP |
| GH2 | gain | 129 | 0.348648649 | LIHC |
| GH2 | gain | 249 | 0.48255814 | LUAD |
| GH2 | gain | 225 | 0.449101796 | LUSC |
| GH2 | gain | 75 | 0.291828794 | SARC |
| GH2 | gain | 97 | 0.219954649 | STAD |
| GH2 | gain | 164 | 0.2624 | STES |
| GHR | gain | 192 | 0.470588235 | BLCA |
| GHR | gain | 358 | 0.331481481 | BRCA |
| GHR | gain | 119 | 0.403389831 | CESC |
| GHR | gain | 11 | 0.305555556 | CHOL |
| GHR | gain | 87 | 0.472826087 | ESCA |
| GHR | gain | 209 | 0.400383142 | HNSC |
| GHR | gain | 212 | 0.240362812 | KIPAN |
| GHR | gain | 172 | 0.325757576 | KIRC |
| GHR | gain | 139 | 0.375675676 | LIHC |
| GHR | gain | 299 | 0.579457364 | LUAD |
| GHR | gain | 363 | 0.724550898 | LUSC |
| GHR | gain | 110 | 0.428015564 | SARC |
| GHR | gain | 126 | 0.285714286 | STAD |
| GHR | gain | 213 | 0.3408 | STES |
| IFNAR1 | gain | 131 | 0.321078431 | BLCA |
| IFNAR1 | gain | 227 | 0.210185185 | BRCA |
| IFNAR1 | gain | 61 | 0.237354086 | SARC |
| IFNAR2 | gain | 132 | 0.323529412 | BLCA |
| IFNAR2 | gain | 224 | 0.207407407 | BRCA |
| IFNAR2 | gain | 60 | 0.233463035 | SARC |
| IFNG | gain | 108 | 0.264705882 | BLCA |
| IFNG | gain | 262 | 0.242592593 | BRCA |
| IFNG | gain | 9 | 0.25 | CHOL |
| IFNG | gain | 46 | 0.25 | ESCA |
| IFNG | gain | 21 | 0.318181818 | KICH |
| IFNG | gain | 256 | 0.290249433 | KIPAN |
| IFNG | gain | 122 | 0.231060606 | KIRC |
| IFNG | gain | 113 | 0.392361111 | KIRP |
| IFNG | gain | 162 | 0.313953488 | LUAD |
| IFNG | gain | 176 | 0.351297405 | LUSC |
| IFNG | gain | 64 | 0.249027237 | SARC |
| IFNG | gain | 102 | 0.231292517 | STAD |
| IFNG | gain | 148 | 0.2368 | STES |
| IFNGR1 | gain | 42 | 0.22826087 | ESCA |
| IFNGR1 | gain | 101 | 0.201596806 | LUSC |
| IFNGR1 | gain | 68 | 0.26459144 | SARC |
| IFNGR1 | gain | 129 | 0.2064 | STES |
| IFNGR2 | gain | 131 | 0.321078431 | BLCA |
| IFNGR2 | gain | 227 | 0.210185185 | BRCA |
| IFNGR2 | gain | 61 | 0.237354086 | SARC |
| IFNL1 | gain | 165 | 0.404411765 | BLCA |
| IFNL1 | gain | 264 | 0.244444444 | BRCA |
| IFNL1 | gain | 90 | 0.305084746 | CESC |
| IFNL1 | gain | 93 | 0.206208426 | COAD |
| IFNL1 | gain | 46 | 0.25 | ESCA |
| IFNL1 | gain | 197 | 0.341421144 | GBM |
| IFNL1 | gain | 229 | 0.210091743 | GBMLGG |
| IFNL1 | gain | 18 | 0.272727273 | KICH |
| IFNL1 | gain | 78 | 0.210810811 | LIHC |
| IFNL1 | gain | 132 | 0.255813953 | LUAD |
| IFNL1 | gain | 227 | 0.453093812 | LUSC |
| IFNL1 | gain | 47 | 0.255434783 | PAAD |
| IFNL1 | gain | 74 | 0.287937743 | SARC |
| IFNL1 | gain | 100 | 0.22675737 | STAD |
| IFNL1 | gain | 146 | 0.2336 | STES |
| IFNL2 | gain | 164 | 0.401960784 | BLCA |
| IFNL2 | gain | 262 | 0.242592593 | BRCA |
| IFNL2 | gain | 90 | 0.305084746 | CESC |
| IFNL2 | gain | 93 | 0.206208426 | COAD |
| IFNL2 | gain | 47 | 0.255434783 | ESCA |
| IFNL2 | gain | 197 | 0.341421144 | GBM |
| IFNL2 | gain | 229 | 0.210091743 | GBMLGG |
| IFNL2 | gain | 18 | 0.272727273 | KICH |
| IFNL2 | gain | 78 | 0.210810811 | LIHC |
| IFNL2 | gain | 131 | 0.253875969 | LUAD |
| IFNL2 | gain | 227 | 0.453093812 | LUSC |
| IFNL2 | gain | 47 | 0.255434783 | PAAD |
| IFNL2 | gain | 74 | 0.287937743 | SARC |
| IFNL2 | gain | 100 | 0.22675737 | STAD |
| IFNL2 | gain | 147 | 0.2352 | STES |
| IFNL3 | gain | 164 | 0.401960784 | BLCA |
| IFNL3 | gain | 262 | 0.242592593 | BRCA |
| IFNL3 | gain | 90 | 0.305084746 | CESC |
| IFNL3 | gain | 93 | 0.206208426 | COAD |
| IFNL3 | gain | 47 | 0.255434783 | ESCA |
| IFNL3 | gain | 197 | 0.341421144 | GBM |
| IFNL3 | gain | 228 | 0.209174312 | GBMLGG |
| IFNL3 | gain | 18 | 0.272727273 | KICH |
| IFNL3 | gain | 77 | 0.208108108 | LIHC |
| IFNL3 | gain | 131 | 0.253875969 | LUAD |
| IFNL3 | gain | 227 | 0.453093812 | LUSC |
| IFNL3 | gain | 47 | 0.255434783 | PAAD |
| IFNL3 | gain | 73 | 0.284046693 | SARC |
| IFNL3 | gain | 100 | 0.22675737 | STAD |
| IFNL3 | gain | 147 | 0.2352 | STES |
| IFNLR1 | gain | 86 | 0.210784314 | BLCA |
| IFNLR1 | gain | 81 | 0.274576271 | CESC |
| IFNLR1 | gain | 115 | 0.222868217 | LUAD |
| IFNLR1 | gain | 79 | 0.307392996 | SARC |
| IL10 | gain | 131 | 0.321078431 | BLCA |
| IL10 | gain | 811 | 0.750925926 | BRCA |
| IL10 | gain | 151 | 0.511864407 | CESC |
| IL10 | gain | 22 | 0.611111111 | CHOL |
| IL10 | gain | 98 | 0.2172949 | COAD |
| IL10 | gain | 86 | 0.467391304 | ESCA |
| IL10 | gain | 139 | 0.266283525 | HNSC |
| IL10 | gain | 271 | 0.732432432 | LIHC |
| IL10 | gain | 364 | 0.705426357 | LUAD |
| IL10 | gain | 254 | 0.506986028 | LUSC |
| IL10 | gain | 56 | 0.304347826 | PAAD |
| IL10 | gain | 128 | 0.290249433 | STAD |
| IL10 | gain | 214 | 0.3424 | STES |
| IL10 | gain | 222 | 0.41187384 | UCEC |
| IL10RA | gain | 17 | 0.257575758 | KICH |
| IL10RA | gain | 121 | 0.234496124 | LUAD |
| IL10RB | gain | 132 | 0.323529412 | BLCA |
| IL10RB | gain | 224 | 0.207407407 | BRCA |
| IL10RB | gain | 61 | 0.237354086 | SARC |
| IL11 | gain | 160 | 0.392156863 | BLCA |
| IL11 | gain | 272 | 0.251851852 | BRCA |
| IL11 | gain | 95 | 0.322033898 | CESC |
| IL11 | gain | 91 | 0.201773836 | COAD |
| IL11 | gain | 56 | 0.304347826 | ESCA |
| IL11 | gain | 180 | 0.311958406 | GBM |
| IL11 | gain | 18 | 0.272727273 | KICH |
| IL11 | gain | 81 | 0.218918919 | LIHC |
| IL11 | gain | 184 | 0.367265469 | LUSC |
| IL11 | gain | 108 | 0.244897959 | STAD |
| IL11 | gain | 164 | 0.2624 | STES |
| IL11RA | gain | 37 | 0.201086957 | ESCA |
| IL11RA | gain | 136 | 0.260536398 | HNSC |
| IL11RA | gain | 135 | 0.269461078 | LUSC |
| IL11RA | gain | 53 | 0.206225681 | SARC |
| IL11RA | gain | 125 | 0.2 | STES |
| IL12A | gain | 206 | 0.504901961 | BLCA |
| IL12A | gain | 302 | 0.27962963 | BRCA |
| IL12A | gain | 215 | 0.728813559 | CESC |
| IL12A | gain | 8 | 0.222222222 | CHOL |
| IL12A | gain | 118 | 0.641304348 | ESCA |
| IL12A | gain | 362 | 0.69348659 | HNSC |
| IL12A | gain | 186 | 0.210884354 | KIPAN |
| IL12A | gain | 96 | 0.333333333 | KIRP |
| IL12A | gain | 139 | 0.269379845 | LUAD |
| IL12A | gain | 427 | 0.852295409 | LUSC |
| IL12A | gain | 140 | 0.317460317 | STAD |
| IL12A | gain | 258 | 0.4128 | STES |
| IL12A | gain | 135 | 0.250463822 | UCEC |
| IL12B | gain | 236 | 0.218518519 | BRCA |
| IL12B | gain | 12 | 0.333333333 | CHOL |
| IL12B | gain | 371 | 0.420634921 | KIPAN |
| IL12B | gain | 328 | 0.621212121 | KIRC |
| IL12B | gain | 123 | 0.332432432 | LIHC |
| IL12B | gain | 112 | 0.217054264 | LUAD |
| IL12B | gain | 63 | 0.245136187 | SARC |
| IL12RB1 | gain | 221 | 0.20462963 | BRCA |
| IL12RB1 | gain | 228 | 0.395147314 | GBM |
| IL12RB1 | gain | 337 | 0.309174312 | GBMLGG |
| IL12RB1 | gain | 20 | 0.303030303 | KICH |
| IL12RB1 | gain | 128 | 0.255489022 | LUSC |
| IL12RB1 | gain | 92 | 0.357976654 | SARC |
| IL12RB2 | gain | 88 | 0.298305085 | CESC |
| IL12RB2 | gain | 9 | 0.25 | CHOL |
| IL12RB2 | gain | 128 | 0.248062016 | LUAD |
| IL12RB2 | gain | 81 | 0.315175097 | SARC |
| IL13 | gain | 12 | 0.333333333 | CHOL |
| IL13 | gain | 357 | 0.404761905 | KIPAN |
| IL13 | gain | 313 | 0.59280303 | KIRC |
| IL13 | gain | 117 | 0.316216216 | LIHC |
| IL13 | gain | 65 | 0.252918288 | SARC |
| IL13RA1 | gain | 59 | 0.320652174 | ESCA |
| IL13RA1 | gain | 121 | 0.231800766 | HNSC |
| IL13RA1 | gain | 96 | 0.333333333 | KIRP |
| IL13RA1 | gain | 118 | 0.228682171 | LUAD |
| IL13RA1 | gain | 115 | 0.229540918 | LUSC |
| IL13RA1 | gain | 133 | 0.2128 | STES |
| IL13RA2 | gain | 59 | 0.320652174 | ESCA |
| IL13RA2 | gain | 123 | 0.235632184 | HNSC |
| IL13RA2 | gain | 97 | 0.336805556 | KIRP |
| IL13RA2 | gain | 115 | 0.222868217 | LUAD |
| IL13RA2 | gain | 116 | 0.231536926 | LUSC |
| IL13RA2 | gain | 129 | 0.2064 | STES |
| IL15 | gain | 23 | 0.348484848 | KICH |
| IL15RA | gain | 178 | 0.43627451 | BLCA |
| IL15RA | gain | 299 | 0.276851852 | BRCA |
| IL15RA | gain | 51 | 0.277173913 | ESCA |
| IL15RA | gain | 131 | 0.253875969 | LUAD |
| IL15RA | gain | 109 | 0.247165533 | STAD |
| IL15RA | gain | 160 | 0.256 | STES |
| IL15RA | gain | 130 | 0.241187384 | UCEC |
| IL17D | gain | 111 | 0.272058824 | BLCA |
| IL17D | gain | 269 | 0.596452328 | COAD |
| IL17D | gain | 40 | 0.217391304 | ESCA |
| IL17D | gain | 157 | 0.35600907 | STAD |
| IL17D | gain | 197 | 0.3152 | STES |
| IL19 | gain | 131 | 0.321078431 | BLCA |
| IL19 | gain | 811 | 0.750925926 | BRCA |
| IL19 | gain | 151 | 0.511864407 | CESC |
| IL19 | gain | 22 | 0.611111111 | CHOL |
| IL19 | gain | 98 | 0.2172949 | COAD |
| IL19 | gain | 87 | 0.472826087 | ESCA |
| IL19 | gain | 139 | 0.266283525 | HNSC |
| IL19 | gain | 271 | 0.732432432 | LIHC |
| IL19 | gain | 364 | 0.705426357 | LUAD |
| IL19 | gain | 254 | 0.506986028 | LUSC |
| IL19 | gain | 56 | 0.304347826 | PAAD |
| IL19 | gain | 129 | 0.292517007 | STAD |
| IL19 | gain | 216 | 0.3456 | STES |
| IL19 | gain | 222 | 0.41187384 | UCEC |
| IL2 | gain | 23 | 0.348484848 | KICH |
| IL20 | gain | 131 | 0.321078431 | BLCA |
| IL20 | gain | 812 | 0.751851852 | BRCA |
| IL20 | gain | 151 | 0.511864407 | CESC |
| IL20 | gain | 22 | 0.611111111 | CHOL |
| IL20 | gain | 98 | 0.2172949 | COAD |
| IL20 | gain | 87 | 0.472826087 | ESCA |
| IL20 | gain | 139 | 0.266283525 | HNSC |
| IL20 | gain | 271 | 0.732432432 | LIHC |
| IL20 | gain | 364 | 0.705426357 | LUAD |
| IL20 | gain | 254 | 0.506986028 | LUSC |
| IL20 | gain | 56 | 0.304347826 | PAAD |
| IL20 | gain | 130 | 0.29478458 | STAD |
| IL20 | gain | 217 | 0.3472 | STES |
| IL20 | gain | 222 | 0.41187384 | UCEC |
| IL20RA | gain | 41 | 0.222826087 | ESCA |
| IL20RA | gain | 101 | 0.201596806 | LUSC |
| IL20RA | gain | 67 | 0.260700389 | SARC |
| IL20RA | gain | 127 | 0.2032 | STES |
| IL20RB | gain | 200 | 0.490196078 | BLCA |
| IL20RB | gain | 264 | 0.244444444 | BRCA |
| IL20RB | gain | 198 | 0.671186441 | CESC |
| IL20RB | gain | 99 | 0.538043478 | ESCA |
| IL20RB | gain | 322 | 0.616858238 | HNSC |
| IL20RB | gain | 179 | 0.202947846 | KIPAN |
| IL20RB | gain | 95 | 0.329861111 | KIRP |
| IL20RB | gain | 121 | 0.234496124 | LUAD |
| IL20RB | gain | 384 | 0.766467066 | LUSC |
| IL20RB | gain | 119 | 0.26984127 | STAD |
| IL20RB | gain | 218 | 0.3488 | STES |
| IL20RB | gain | 116 | 0.215213358 | UCEC |
| IL21 | gain | 23 | 0.348484848 | KICH |
| IL21R | gain | 92 | 0.225490196 | BLCA |
| IL21R | gain | 545 | 0.50462963 | BRCA |
| IL21R | gain | 8 | 0.222222222 | CHOL |
| IL21R | gain | 116 | 0.257206208 | COAD |
| IL21R | gain | 42 | 0.22826087 | ESCA |
| IL21R | gain | 114 | 0.218390805 | HNSC |
| IL21R | gain | 22 | 0.333333333 | KICH |
| IL21R | gain | 283 | 0.320861678 | KIPAN |
| IL21R | gain | 108 | 0.204545455 | KIRC |
| IL21R | gain | 153 | 0.53125 | KIRP |
| IL21R | gain | 159 | 0.308139535 | LUAD |
| IL21R | gain | 114 | 0.22754491 | LUSC |
| IL21R | gain | 54 | 0.210116732 | SARC |
| IL22 | gain | 107 | 0.262254902 | BLCA |
| IL22 | gain | 263 | 0.243518519 | BRCA |
| IL22 | gain | 9 | 0.25 | CHOL |
| IL22 | gain | 46 | 0.25 | ESCA |
| IL22 | gain | 21 | 0.318181818 | KICH |
| IL22 | gain | 256 | 0.290249433 | KIPAN |
| IL22 | gain | 122 | 0.231060606 | KIRC |
| IL22 | gain | 113 | 0.392361111 | KIRP |
| IL22 | gain | 162 | 0.313953488 | LUAD |
| IL22 | gain | 176 | 0.351297405 | LUSC |
| IL22 | gain | 64 | 0.249027237 | SARC |
| IL22 | gain | 103 | 0.233560091 | STAD |
| IL22 | gain | 149 | 0.2384 | STES |
| IL22RA1 | gain | 85 | 0.208333333 | BLCA |
| IL22RA1 | gain | 81 | 0.274576271 | CESC |
| IL22RA1 | gain | 115 | 0.222868217 | LUAD |
| IL22RA1 | gain | 79 | 0.307392996 | SARC |
| IL22RA2 | gain | 42 | 0.22826087 | ESCA |
| IL22RA2 | gain | 101 | 0.201596806 | LUSC |
| IL22RA2 | gain | 67 | 0.260700389 | SARC |
| IL22RA2 | gain | 129 | 0.2064 | STES |
| IL23A | gain | 91 | 0.223039216 | BLCA |
| IL23A | gain | 222 | 0.205555556 | BRCA |
| IL23A | gain | 8 | 0.222222222 | CHOL |
| IL23A | gain | 95 | 0.210643016 | COAD |
| IL23A | gain | 40 | 0.217391304 | ESCA |
| IL23A | gain | 21 | 0.318181818 | KICH |
| IL23A | gain | 255 | 0.289115646 | KIPAN |
| IL23A | gain | 121 | 0.229166667 | KIRC |
| IL23A | gain | 113 | 0.392361111 | KIRP |
| IL23A | gain | 159 | 0.308139535 | LUAD |
| IL23A | gain | 158 | 0.315369261 | LUSC |
| IL23A | gain | 97 | 0.219954649 | STAD |
| IL23A | gain | 137 | 0.2192 | STES |
| IL23R | gain | 88 | 0.298305085 | CESC |
| IL23R | gain | 9 | 0.25 | CHOL |
| IL23R | gain | 128 | 0.248062016 | LUAD |
| IL23R | gain | 80 | 0.311284047 | SARC |
| IL24 | gain | 131 | 0.321078431 | BLCA |
| IL24 | gain | 812 | 0.751851852 | BRCA |
| IL24 | gain | 151 | 0.511864407 | CESC |
| IL24 | gain | 22 | 0.611111111 | CHOL |
| IL24 | gain | 98 | 0.2172949 | COAD |
| IL24 | gain | 87 | 0.472826087 | ESCA |
| IL24 | gain | 139 | 0.266283525 | HNSC |
| IL24 | gain | 271 | 0.732432432 | LIHC |
| IL24 | gain | 364 | 0.705426357 | LUAD |
| IL24 | gain | 254 | 0.506986028 | LUSC |
| IL24 | gain | 56 | 0.304347826 | PAAD |
| IL24 | gain | 131 | 0.297052154 | STAD |
| IL24 | gain | 218 | 0.3488 | STES |
| IL24 | gain | 222 | 0.41187384 | UCEC |
| IL27RA | gain | 219 | 0.202777778 | BRCA |
| IL27RA | gain | 232 | 0.402079723 | GBM |
| IL27RA | gain | 344 | 0.31559633 | GBMLGG |
| IL27RA | gain | 20 | 0.303030303 | KICH |
| IL27RA | gain | 114 | 0.22754491 | LUSC |
| IL27RA | gain | 100 | 0.389105058 | SARC |
| IL2RA | gain | 178 | 0.43627451 | BLCA |
| IL2RA | gain | 300 | 0.277777778 | BRCA |
| IL2RA | gain | 51 | 0.277173913 | ESCA |
| IL2RA | gain | 131 | 0.253875969 | LUAD |
| IL2RA | gain | 108 | 0.244897959 | STAD |
| IL2RA | gain | 159 | 0.2544 | STES |
| IL2RA | gain | 130 | 0.241187384 | UCEC |
| IL2RB | gain | 112 | 0.214559387 | HNSC |
| IL2RB | gain | 19 | 0.287878788 | KICH |
| IL2RB | gain | 234 | 0.467065868 | LUSC |
| IL2RB | gain | 64 | 0.249027237 | SARC |
| IL2RG | gain | 49 | 0.266304348 | ESCA |
| IL2RG | gain | 100 | 0.347222222 | KIRP |
| IL2RG | gain | 55 | 0.214007782 | SARC |
| IL3 | gain | 11 | 0.305555556 | CHOL |
| IL3 | gain | 358 | 0.405895692 | KIPAN |
| IL3 | gain | 314 | 0.59469697 | KIRC |
| IL3 | gain | 115 | 0.310810811 | LIHC |
| IL3 | gain | 64 | 0.249027237 | SARC |
| IL4 | gain | 12 | 0.333333333 | CHOL |
| IL4 | gain | 357 | 0.404761905 | KIPAN |
| IL4 | gain | 313 | 0.59280303 | KIRC |
| IL4 | gain | 117 | 0.316216216 | LIHC |
| IL4 | gain | 65 | 0.252918288 | SARC |
| IL4R | gain | 92 | 0.225490196 | BLCA |
| IL4R | gain | 546 | 0.505555556 | BRCA |
| IL4R | gain | 8 | 0.222222222 | CHOL |
| IL4R | gain | 116 | 0.257206208 | COAD |
| IL4R | gain | 42 | 0.22826087 | ESCA |
| IL4R | gain | 114 | 0.218390805 | HNSC |
| IL4R | gain | 22 | 0.333333333 | KICH |
| IL4R | gain | 283 | 0.320861678 | KIPAN |
| IL4R | gain | 108 | 0.204545455 | KIRC |
| IL4R | gain | 153 | 0.53125 | KIRP |
| IL4R | gain | 159 | 0.308139535 | LUAD |
| IL4R | gain | 113 | 0.225548902 | LUSC |
| IL4R | gain | 53 | 0.206225681 | SARC |
| IL5 | gain | 12 | 0.333333333 | CHOL |
| IL5 | gain | 357 | 0.404761905 | KIPAN |
| IL5 | gain | 313 | 0.59280303 | KIRC |
| IL5 | gain | 116 | 0.313513514 | LIHC |
| IL5 | gain | 64 | 0.249027237 | SARC |
| IL5RA | gain | 156 | 0.382352941 | BLCA |
| IL5RA | gain | 72 | 0.25 | KIRP |
| IL5RA | gain | 70 | 0.272373541 | SARC |
| IL6 | gain | 182 | 0.446078431 | BLCA |
| IL6 | gain | 347 | 0.321296296 | BRCA |
| IL6 | gain | 8 | 0.222222222 | CHOL |
| IL6 | gain | 255 | 0.5654102 | COAD |
| IL6 | gain | 125 | 0.679347826 | ESCA |
| IL6 | gain | 458 | 0.793760832 | GBM |
| IL6 | gain | 575 | 0.527522936 | GBMLGG |
| IL6 | gain | 196 | 0.375478927 | HNSC |
| IL6 | gain | 24 | 0.363636364 | KICH |
| IL6 | gain | 369 | 0.418367347 | KIPAN |
| IL6 | gain | 174 | 0.329545455 | KIRC |
| IL6 | gain | 171 | 0.59375 | KIRP |
| IL6 | gain | 118 | 0.318918919 | LIHC |
| IL6 | gain | 283 | 0.548449612 | LUAD |
| IL6 | gain | 256 | 0.510978044 | LUSC |
| IL6 | gain | 52 | 0.282608696 | PAAD |
| IL6 | gain | 89 | 0.346303502 | SARC |
| IL6 | gain | 217 | 0.492063492 | STAD |
| IL6 | gain | 342 | 0.5472 | STES |
| IL6R | gain | 189 | 0.463235294 | BLCA |
| IL6R | gain | 791 | 0.732407407 | BRCA |
| IL6R | gain | 158 | 0.53559322 | CESC |
| IL6R | gain | 23 | 0.638888889 | CHOL |
| IL6R | gain | 91 | 0.201773836 | COAD |
| IL6R | gain | 89 | 0.483695652 | ESCA |
| IL6R | gain | 133 | 0.254789272 | HNSC |
| IL6R | gain | 277 | 0.748648649 | LIHC |
| IL6R | gain | 382 | 0.740310078 | LUAD |
| IL6R | gain | 263 | 0.5249501 | LUSC |
| IL6R | gain | 62 | 0.336956522 | PAAD |
| IL6R | gain | 99 | 0.385214008 | SARC |
| IL6R | gain | 160 | 0.362811791 | STAD |
| IL6R | gain | 249 | 0.3984 | STES |
| IL6R | gain | 239 | 0.443413729 | UCEC |
| IL6ST | gain | 8 | 0.222222222 | CHOL |
| IL6ST | gain | 203 | 0.23015873 | KIPAN |
| IL6ST | gain | 165 | 0.3125 | KIRC |
| IL6ST | gain | 106 | 0.286486486 | LIHC |
| IL6ST | gain | 129 | 0.25 | LUAD |
| IL6ST | gain | 72 | 0.280155642 | SARC |
| IL7 | gain | 230 | 0.56372549 | BLCA |
| IL7 | gain | 614 | 0.568518519 | BRCA |
| IL7 | gain | 96 | 0.325423729 | CESC |
| IL7 | gain | 13 | 0.361111111 | CHOL |
| IL7 | gain | 248 | 0.549889135 | COAD |
| IL7 | gain | 111 | 0.60326087 | ESCA |
| IL7 | gain | 339 | 0.649425287 | HNSC |
| IL7 | gain | 19 | 0.287878788 | KICH |
| IL7 | gain | 208 | 0.562162162 | LIHC |
| IL7 | gain | 276 | 0.534883721 | LUAD |
| IL7 | gain | 276 | 0.550898204 | LUSC |
| IL7 | gain | 57 | 0.309782609 | PAAD |
| IL7 | gain | 82 | 0.319066148 | SARC |
| IL7 | gain | 261 | 0.591836735 | STAD |
| IL7 | gain | 372 | 0.5952 | STES |
| IL7 | gain | 172 | 0.319109462 | UCEC |
| IL7R | gain | 197 | 0.482843137 | BLCA |
| IL7R | gain | 357 | 0.330555556 | BRCA |
| IL7R | gain | 121 | 0.410169492 | CESC |
| IL7R | gain | 12 | 0.333333333 | CHOL |
| IL7R | gain | 88 | 0.47826087 | ESCA |
| IL7R | gain | 221 | 0.423371648 | HNSC |
| IL7R | gain | 214 | 0.242630385 | KIPAN |
| IL7R | gain | 170 | 0.321969697 | KIRC |
| IL7R | gain | 145 | 0.391891892 | LIHC |
| IL7R | gain | 308 | 0.596899225 | LUAD |
| IL7R | gain | 371 | 0.740518962 | LUSC |
| IL7R | gain | 112 | 0.435797665 | SARC |
| IL7R | gain | 127 | 0.287981859 | STAD |
| IL7R | gain | 215 | 0.344 | STES |
| IL9 | gain | 12 | 0.333333333 | CHOL |
| IL9 | gain | 358 | 0.405895692 | KIPAN |
| IL9 | gain | 316 | 0.598484848 | KIRC |
| IL9 | gain | 114 | 0.308108108 | LIHC |
| IL9 | gain | 66 | 0.256809339 | SARC |
| IRF9 | gain | 54 | 0.293478261 | ESCA |
| IRF9 | gain | 149 | 0.285440613 | HNSC |
| IRF9 | gain | 21 | 0.318181818 | KICH |
| IRF9 | gain | 166 | 0.321705426 | LUAD |
| IRF9 | gain | 140 | 0.279441118 | LUSC |
| IRF9 | gain | 77 | 0.299610895 | SARC |
| JAK1 | gain | 93 | 0.315254237 | CESC |
| JAK1 | gain | 9 | 0.25 | CHOL |
| JAK1 | gain | 125 | 0.242248062 | LUAD |
| JAK1 | gain | 81 | 0.315175097 | SARC |
| JAK2 | gain | 87 | 0.213235294 | BLCA |
| JAK2 | gain | 62 | 0.210169492 | CESC |
| JAK2 | gain | 124 | 0.237547893 | HNSC |
| JAK2 | gain | 55 | 0.214007782 | SARC |
| JAK3 | gain | 220 | 0.203703704 | BRCA |
| JAK3 | gain | 228 | 0.395147314 | GBM |
| JAK3 | gain | 337 | 0.309174312 | GBMLGG |
| JAK3 | gain | 20 | 0.303030303 | KICH |
| JAK3 | gain | 125 | 0.249500998 | LUSC |
| JAK3 | gain | 91 | 0.354085603 | SARC |
| LIF | gain | 84 | 0.205882353 | BLCA |
| LIF | gain | 120 | 0.229885057 | HNSC |
| LIF | gain | 19 | 0.287878788 | KICH |
| LIF | gain | 257 | 0.512974052 | LUSC |
| LIF | gain | 67 | 0.260700389 | SARC |
| LIFR | gain | 194 | 0.475490196 | BLCA |
| LIFR | gain | 355 | 0.328703704 | BRCA |
| LIFR | gain | 123 | 0.416949153 | CESC |
| LIFR | gain | 12 | 0.333333333 | CHOL |
| LIFR | gain | 91 | 0.494565217 | ESCA |
| LIFR | gain | 220 | 0.421455939 | HNSC |
| LIFR | gain | 214 | 0.242630385 | KIPAN |
| LIFR | gain | 171 | 0.323863636 | KIRC |
| LIFR | gain | 143 | 0.386486486 | LIHC |
| LIFR | gain | 304 | 0.589147287 | LUAD |
| LIFR | gain | 369 | 0.736526946 | LUSC |
| LIFR | gain | 115 | 0.447470817 | SARC |
| LIFR | gain | 128 | 0.290249433 | STAD |
| LIFR | gain | 219 | 0.3504 | STES |
| MCL1 | gain | 203 | 0.49754902 | BLCA |
| MCL1 | gain | 779 | 0.721296296 | BRCA |
| MCL1 | gain | 157 | 0.53220339 | CESC |
| MCL1 | gain | 22 | 0.611111111 | CHOL |
| MCL1 | gain | 89 | 0.483695652 | ESCA |
| MCL1 | gain | 132 | 0.252873563 | HNSC |
| MCL1 | gain | 272 | 0.735135135 | LIHC |
| MCL1 | gain | 375 | 0.726744186 | LUAD |
| MCL1 | gain | 258 | 0.51497006 | LUSC |
| MCL1 | gain | 59 | 0.320652174 | PAAD |
| MCL1 | gain | 101 | 0.392996109 | SARC |
| MCL1 | gain | 157 | 0.35600907 | STAD |
| MCL1 | gain | 246 | 0.3936 | STES |
| MCL1 | gain | 243 | 0.450834879 | UCEC |
| MPL | gain | 89 | 0.218137255 | BLCA |
| MPL | gain | 95 | 0.322033898 | CESC |
| MPL | gain | 8 | 0.222222222 | CHOL |
| MPL | gain | 143 | 0.277131783 | LUAD |
| MPL | gain | 74 | 0.287937743 | SARC |
| OSM | gain | 84 | 0.205882353 | BLCA |
| OSM | gain | 120 | 0.229885057 | HNSC |
| OSM | gain | 19 | 0.287878788 | KICH |
| OSM | gain | 256 | 0.510978044 | LUSC |
| OSM | gain | 67 | 0.260700389 | SARC |
| OSMR | gain | 190 | 0.465686275 | BLCA |
| OSMR | gain | 357 | 0.330555556 | BRCA |
| OSMR | gain | 123 | 0.416949153 | CESC |
| OSMR | gain | 11 | 0.305555556 | CHOL |
| OSMR | gain | 92 | 0.5 | ESCA |
| OSMR | gain | 219 | 0.41954023 | HNSC |
| OSMR | gain | 214 | 0.242630385 | KIPAN |
| OSMR | gain | 171 | 0.323863636 | KIRC |
| OSMR | gain | 142 | 0.383783784 | LIHC |
| OSMR | gain | 305 | 0.591085271 | LUAD |
| OSMR | gain | 369 | 0.736526946 | LUSC |
| OSMR | gain | 115 | 0.447470817 | SARC |
| OSMR | gain | 129 | 0.292517007 | STAD |
| OSMR | gain | 221 | 0.3536 | STES |
| PIAS1 | gain | 63 | 0.213559322 | CESC |
| PIAS1 | gain | 22 | 0.333333333 | KICH |
| PIAS1 | gain | 130 | 0.259481038 | LUSC |
| PIAS1 | gain | 74 | 0.287937743 | SARC |
| PIAS2 | gain | 16 | 0.242424242 | KICH |
| PIAS2 | gain | 103 | 0.205588822 | LUSC |
| PIAS3 | gain | 184 | 0.450980392 | BLCA |
| PIAS3 | gain | 686 | 0.635185185 | BRCA |
| PIAS3 | gain | 136 | 0.461016949 | CESC |
| PIAS3 | gain | 19 | 0.527777778 | CHOL |
| PIAS3 | gain | 76 | 0.413043478 | ESCA |
| PIAS3 | gain | 237 | 0.640540541 | LIHC |
| PIAS3 | gain | 328 | 0.635658915 | LUAD |
| PIAS3 | gain | 213 | 0.425149701 | LUSC |
| PIAS3 | gain | 57 | 0.309782609 | PAAD |
| PIAS3 | gain | 93 | 0.361867704 | SARC |
| PIAS3 | gain | 134 | 0.303854875 | STAD |
| PIAS3 | gain | 210 | 0.336 | STES |
| PIAS3 | gain | 223 | 0.413729128 | UCEC |
| PIAS4 | gain | 234 | 0.405545927 | GBM |
| PIAS4 | gain | 349 | 0.320183486 | GBMLGG |
| PIAS4 | gain | 19 | 0.287878788 | KICH |
| PIAS4 | gain | 97 | 0.377431907 | SARC |
| PIM1 | gain | 96 | 0.235294118 | BLCA |
| PIM1 | gain | 258 | 0.238888889 | BRCA |
| PIM1 | gain | 68 | 0.230508475 | CESC |
| PIM1 | gain | 9 | 0.25 | CHOL |
| PIM1 | gain | 92 | 0.203991131 | COAD |
| PIM1 | gain | 54 | 0.293478261 | ESCA |
| PIM1 | gain | 143 | 0.386486486 | LIHC |
| PIM1 | gain | 190 | 0.368217054 | LUAD |
| PIM1 | gain | 128 | 0.255489022 | LUSC |
| PIM1 | gain | 55 | 0.214007782 | SARC |
| PIM1 | gain | 95 | 0.215419501 | STAD |
| PIM1 | gain | 149 | 0.2384 | STES |
| PRL | gain | 155 | 0.379901961 | BLCA |
| PRL | gain | 290 | 0.268518519 | BRCA |
| PRL | gain | 70 | 0.237288136 | CESC |
| PRL | gain | 10 | 0.277777778 | CHOL |
| PRL | gain | 42 | 0.22826087 | ESCA |
| PRL | gain | 152 | 0.410810811 | LIHC |
| PRL | gain | 184 | 0.356589147 | LUAD |
| PRL | gain | 125 | 0.249500998 | LUSC |
| PRL | gain | 52 | 0.20233463 | SARC |
| PRL | gain | 130 | 0.208 | STES |
| PRLR | gain | 195 | 0.477941176 | BLCA |
| PRLR | gain | 361 | 0.334259259 | BRCA |
| PRLR | gain | 121 | 0.410169492 | CESC |
| PRLR | gain | 12 | 0.333333333 | CHOL |
| PRLR | gain | 88 | 0.47826087 | ESCA |
| PRLR | gain | 220 | 0.421455939 | HNSC |
| PRLR | gain | 216 | 0.244897959 | KIPAN |
| PRLR | gain | 170 | 0.321969697 | KIRC |
| PRLR | gain | 146 | 0.394594595 | LIHC |
| PRLR | gain | 308 | 0.596899225 | LUAD |
| PRLR | gain | 371 | 0.740518962 | LUSC |
| PRLR | gain | 114 | 0.443579767 | SARC |
| PRLR | gain | 126 | 0.285714286 | STAD |
| PRLR | gain | 214 | 0.3424 | STES |
| PTPN11 | gain | 106 | 0.259803922 | BLCA |
| PTPN11 | gain | 8 | 0.222222222 | CHOL |
| PTPN11 | gain | 41 | 0.222826087 | ESCA |
| PTPN11 | gain | 20 | 0.303030303 | KICH |
| PTPN11 | gain | 254 | 0.287981859 | KIPAN |
| PTPN11 | gain | 121 | 0.229166667 | KIRC |
| PTPN11 | gain | 113 | 0.392361111 | KIRP |
| PTPN11 | gain | 121 | 0.234496124 | LUAD |
| PTPN11 | gain | 129 | 0.25748503 | LUSC |
| PTPN11 | gain | 54 | 0.210116732 | SARC |
| PTPN11 | gain | 91 | 0.206349206 | STAD |
| PTPN11 | gain | 132 | 0.2112 | STES |
| PTPN2 | gain | 126 | 0.308823529 | BLCA |
| PTPN2 | gain | 58 | 0.315217391 | ESCA |
| PTPN2 | gain | 135 | 0.25862069 | HNSC |
| PTPN2 | gain | 17 | 0.257575758 | KICH |
| PTPN2 | gain | 114 | 0.220930233 | LUAD |
| PTPN2 | gain | 164 | 0.327345309 | LUSC |
| PTPN2 | gain | 66 | 0.256809339 | SARC |
| PTPN2 | gain | 100 | 0.22675737 | STAD |
| PTPN2 | gain | 158 | 0.2528 | STES |
| PTPN6 | gain | 131 | 0.321078431 | BLCA |
| PTPN6 | gain | 272 | 0.251851852 | BRCA |
| PTPN6 | gain | 12 | 0.333333333 | CHOL |
| PTPN6 | gain | 104 | 0.23059867 | COAD |
| PTPN6 | gain | 61 | 0.331521739 | ESCA |
| PTPN6 | gain | 164 | 0.314176245 | HNSC |
| PTPN6 | gain | 21 | 0.318181818 | KICH |
| PTPN6 | gain | 257 | 0.29138322 | KIPAN |
| PTPN6 | gain | 123 | 0.232954545 | KIRC |
| PTPN6 | gain | 113 | 0.392361111 | KIRP |
| PTPN6 | gain | 147 | 0.284883721 | LUAD |
| PTPN6 | gain | 257 | 0.512974052 | LUSC |
| PTPN6 | gain | 104 | 0.235827664 | STAD |
| PTPN6 | gain | 165 | 0.264 | STES |
| SOCS1 | gain | 89 | 0.218137255 | BLCA |
| SOCS1 | gain | 566 | 0.524074074 | BRCA |
| SOCS1 | gain | 60 | 0.203389831 | CESC |
| SOCS1 | gain | 109 | 0.241685144 | COAD |
| SOCS1 | gain | 39 | 0.211956522 | ESCA |
| SOCS1 | gain | 108 | 0.206896552 | HNSC |
| SOCS1 | gain | 22 | 0.333333333 | KICH |
| SOCS1 | gain | 284 | 0.321995465 | KIPAN |
| SOCS1 | gain | 109 | 0.206439394 | KIRC |
| SOCS1 | gain | 153 | 0.53125 | KIRP |
| SOCS1 | gain | 166 | 0.321705426 | LUAD |
| SOCS1 | gain | 104 | 0.20758483 | LUSC |
| SOCS1 | gain | 61 | 0.237354086 | SARC |
| SOCS2 | gain | 95 | 0.232843137 | BLCA |
| SOCS2 | gain | 8 | 0.222222222 | CHOL |
| SOCS2 | gain | 20 | 0.303030303 | KICH |
| SOCS2 | gain | 255 | 0.289115646 | KIPAN |
| SOCS2 | gain | 122 | 0.231060606 | KIRC |
| SOCS2 | gain | 113 | 0.392361111 | KIRP |
| SOCS2 | gain | 120 | 0.23255814 | LUAD |
| SOCS2 | gain | 138 | 0.275449102 | LUSC |
| SOCS3 | gain | 193 | 0.473039216 | BLCA |
| SOCS3 | gain | 400 | 0.37037037 | BRCA |
| SOCS3 | gain | 66 | 0.223728814 | CESC |
| SOCS3 | gain | 8 | 0.222222222 | CHOL |
| SOCS3 | gain | 105 | 0.232815965 | COAD |
| SOCS3 | gain | 62 | 0.336956522 | ESCA |
| SOCS3 | gain | 111 | 0.212643678 | HNSC |
| SOCS3 | gain | 244 | 0.276643991 | KIPAN |
| SOCS3 | gain | 200 | 0.694444444 | KIRP |
| SOCS3 | gain | 138 | 0.372972973 | LIHC |
| SOCS3 | gain | 261 | 0.505813953 | LUAD |
| SOCS3 | gain | 229 | 0.457085828 | LUSC |
| SOCS3 | gain | 76 | 0.295719844 | SARC |
| SOCS3 | gain | 90 | 0.204081633 | STAD |
| SOCS3 | gain | 152 | 0.2432 | STES |
| SOCS4 | gain | 57 | 0.309782609 | ESCA |
| SOCS4 | gain | 160 | 0.30651341 | HNSC |
| SOCS4 | gain | 21 | 0.318181818 | KICH |
| SOCS4 | gain | 155 | 0.300387597 | LUAD |
| SOCS4 | gain | 126 | 0.251497006 | LUSC |
| SOCS4 | gain | 56 | 0.217898833 | SARC |
| SOCS5 | gain | 132 | 0.323529412 | BLCA |
| SOCS5 | gain | 73 | 0.247457627 | CESC |
| SOCS5 | gain | 69 | 0.375 | ESCA |
| SOCS5 | gain | 107 | 0.204980843 | HNSC |
| SOCS5 | gain | 154 | 0.298449612 | LUAD |
| SOCS5 | gain | 259 | 0.516966068 | LUSC |
| SOCS5 | gain | 157 | 0.2512 | STES |
| SOCS5 | gain | 115 | 0.213358071 | UCEC |
| SOCS6 | gain | 16 | 0.242424242 | KICH |
| SOCS7 | gain | 157 | 0.384803922 | BLCA |
| SOCS7 | gain | 266 | 0.246296296 | BRCA |
| SOCS7 | gain | 10 | 0.277777778 | CHOL |
| SOCS7 | gain | 107 | 0.237250554 | COAD |
| SOCS7 | gain | 61 | 0.331521739 | ESCA |
| SOCS7 | gain | 219 | 0.24829932 | KIPAN |
| SOCS7 | gain | 188 | 0.652777778 | KIRP |
| SOCS7 | gain | 94 | 0.254054054 | LIHC |
| SOCS7 | gain | 220 | 0.426356589 | LUAD |
| SOCS7 | gain | 155 | 0.309381238 | LUSC |
| SOCS7 | gain | 118 | 0.267573696 | STAD |
| SOCS7 | gain | 179 | 0.2864 | STES |
| STAM | gain | 145 | 0.355392157 | BLCA |
| STAM | gain | 278 | 0.257407407 | BRCA |
| STAM | gain | 46 | 0.25 | ESCA |
| STAM | gain | 124 | 0.240310078 | LUAD |
| STAM | gain | 105 | 0.238095238 | STAD |
| STAM | gain | 151 | 0.2416 | STES |
| STAM | gain | 129 | 0.239332096 | UCEC |
| STAM2 | gain | 51 | 0.277173913 | ESCA |
| STAM2 | gain | 152 | 0.294573643 | LUAD |
| STAM2 | gain | 136 | 0.271457086 | LUSC |
| STAT1 | gain | 57 | 0.309782609 | ESCA |
| STAT1 | gain | 147 | 0.284883721 | LUAD |
| STAT1 | gain | 152 | 0.303393214 | LUSC |
| STAT1 | gain | 134 | 0.2144 | STES |
| STAT2 | gain | 91 | 0.223039216 | BLCA |
| STAT2 | gain | 222 | 0.205555556 | BRCA |
| STAT2 | gain | 8 | 0.222222222 | CHOL |
| STAT2 | gain | 95 | 0.210643016 | COAD |
| STAT2 | gain | 40 | 0.217391304 | ESCA |
| STAT2 | gain | 21 | 0.318181818 | KICH |
| STAT2 | gain | 255 | 0.289115646 | KIPAN |
| STAT2 | gain | 121 | 0.229166667 | KIRC |
| STAT2 | gain | 113 | 0.392361111 | KIRP |
| STAT2 | gain | 159 | 0.308139535 | LUAD |
| STAT2 | gain | 158 | 0.315369261 | LUSC |
| STAT2 | gain | 97 | 0.219954649 | STAD |
| STAT2 | gain | 137 | 0.2192 | STES |
| STAT3 | gain | 149 | 0.365196078 | BLCA |
| STAT3 | gain | 218 | 0.201851852 | BRCA |
| STAT3 | gain | 8 | 0.222222222 | CHOL |
| STAT3 | gain | 103 | 0.228381375 | COAD |
| STAT3 | gain | 58 | 0.315217391 | ESCA |
| STAT3 | gain | 220 | 0.249433107 | KIPAN |
| STAT3 | gain | 189 | 0.65625 | KIRP |
| STAT3 | gain | 96 | 0.259459459 | LIHC |
| STAT3 | gain | 219 | 0.424418605 | LUAD |
| STAT3 | gain | 159 | 0.317365269 | LUSC |
| STAT3 | gain | 96 | 0.217687075 | STAD |
| STAT3 | gain | 154 | 0.2464 | STES |
| STAT4 | gain | 57 | 0.309782609 | ESCA |
| STAT4 | gain | 148 | 0.286821705 | LUAD |
| STAT4 | gain | 151 | 0.301397206 | LUSC |
| STAT4 | gain | 134 | 0.2144 | STES |
| STAT5A | gain | 150 | 0.367647059 | BLCA |
| STAT5A | gain | 8 | 0.222222222 | CHOL |
| STAT5A | gain | 103 | 0.228381375 | COAD |
| STAT5A | gain | 58 | 0.315217391 | ESCA |
| STAT5A | gain | 220 | 0.249433107 | KIPAN |
| STAT5A | gain | 189 | 0.65625 | KIRP |
| STAT5A | gain | 94 | 0.254054054 | LIHC |
| STAT5A | gain | 220 | 0.426356589 | LUAD |
| STAT5A | gain | 159 | 0.317365269 | LUSC |
| STAT5A | gain | 98 | 0.222222222 | STAD |
| STAT5A | gain | 156 | 0.2496 | STES |
| STAT5B | gain | 150 | 0.367647059 | BLCA |
| STAT5B | gain | 8 | 0.222222222 | CHOL |
| STAT5B | gain | 105 | 0.232815965 | COAD |
| STAT5B | gain | 59 | 0.320652174 | ESCA |
| STAT5B | gain | 220 | 0.249433107 | KIPAN |
| STAT5B | gain | 189 | 0.65625 | KIRP |
| STAT5B | gain | 94 | 0.254054054 | LIHC |
| STAT5B | gain | 220 | 0.426356589 | LUAD |
| STAT5B | gain | 160 | 0.319361277 | LUSC |
| STAT5B | gain | 99 | 0.224489796 | STAD |
| STAT5B | gain | 158 | 0.2528 | STES |
| STAT6 | gain | 87 | 0.213235294 | BLCA |
| STAT6 | gain | 9 | 0.25 | CHOL |
| STAT6 | gain | 95 | 0.210643016 | COAD |
| STAT6 | gain | 41 | 0.222826087 | ESCA |
| STAT6 | gain | 21 | 0.318181818 | KICH |
| STAT6 | gain | 255 | 0.289115646 | KIPAN |
| STAT6 | gain | 121 | 0.229166667 | KIRC |
| STAT6 | gain | 113 | 0.392361111 | KIRP |
| STAT6 | gain | 158 | 0.30620155 | LUAD |
| STAT6 | gain | 157 | 0.313373253 | LUSC |
| STAT6 | gain | 97 | 0.219954649 | STAD |
| STAT6 | gain | 138 | 0.2208 | STES |
| THPO | gain | 218 | 0.534313725 | BLCA |
| THPO | gain | 314 | 0.290740741 | BRCA |
| THPO | gain | 221 | 0.749152542 | CESC |
| THPO | gain | 10 | 0.277777778 | CHOL |
| THPO | gain | 120 | 0.652173913 | ESCA |
| THPO | gain | 377 | 0.722222222 | HNSC |
| THPO | gain | 189 | 0.214285714 | KIPAN |
| THPO | gain | 99 | 0.34375 | KIRP |
| THPO | gain | 142 | 0.275193798 | LUAD |
| THPO | gain | 449 | 0.896207585 | LUSC |
| THPO | gain | 153 | 0.346938776 | STAD |
| THPO | gain | 273 | 0.4368 | STES |
| THPO | gain | 153 | 0.283858998 | UCEC |
| TSLP | gain | 10 | 0.277777778 | CHOL |
| TSLP | gain | 316 | 0.358276644 | KIPAN |
| TSLP | gain | 272 | 0.515151515 | KIRC |
| TSLP | gain | 114 | 0.308108108 | LIHC |
| TSLP | gain | 70 | 0.272373541 | SARC |
| TYK2 | gain | 82 | 0.200980392 | BLCA |
| TYK2 | gain | 241 | 0.417677643 | GBM |
| TYK2 | gain | 361 | 0.331192661 | GBMLGG |
| TYK2 | gain | 20 | 0.303030303 | KICH |
| TYK2 | gain | 108 | 0.420233463 | SARC |
| AOX1 | loss | 145 | 0.355392157 | BLCA |
| BCL2 | loss | 181 | 0.443627451 | BLCA |
| CDKN1A | loss | 94 | 0.230392157 | BLCA |
| CISH | loss | 124 | 0.303921569 | BLCA |
| CNTF | loss | 88 | 0.215686275 | BLCA |
| CNTFR | loss | 172 | 0.421568627 | BLCA |
| CSF2 | loss | 189 | 0.463235294 | BLCA |
| CSF2RB | loss | 162 | 0.397058824 | BLCA |
| CTF1 | loss | 95 | 0.232843137 | BLCA |
| EPOR | loss | 118 | 0.289215686 | BLCA |
| IFNA1 | loss | 212 | 0.519607843 | BLCA |
| IFNA10 | loss | 205 | 0.50245098 | BLCA |
| IFNA13 | loss | 210 | 0.514705882 | BLCA |
| IFNA14 | loss | 206 | 0.504901961 | BLCA |
| IFNA16 | loss | 205 | 0.50245098 | BLCA |
| IFNA17 | loss | 205 | 0.50245098 | BLCA |
| IFNA2 | loss | 210 | 0.514705882 | BLCA |
| IFNA21 | loss | 204 | 0.5 | BLCA |
| IFNA4 | loss | 205 | 0.50245098 | BLCA |
| IFNA5 | loss | 207 | 0.507352941 | BLCA |
| IFNA6 | loss | 209 | 0.512254902 | BLCA |
| IFNA7 | loss | 205 | 0.50245098 | BLCA |
| IFNA8 | loss | 211 | 0.517156863 | BLCA |
| IFNB1 | loss | 201 | 0.492647059 | BLCA |
| IFNE | loss | 212 | 0.519607843 | BLCA |
| IFNGR1 | loss | 174 | 0.426470588 | BLCA |
| IFNK | loss | 180 | 0.441176471 | BLCA |
| IFNW1 | loss | 202 | 0.495098039 | BLCA |
| IL10RA | loss | 154 | 0.37745098 | BLCA |
| IL11RA | loss | 170 | 0.416666667 | BLCA |
| IL12B | loss | 193 | 0.473039216 | BLCA |
| IL12RB1 | loss | 111 | 0.272058824 | BLCA |
| IL13 | loss | 190 | 0.465686275 | BLCA |
| IL15 | loss | 158 | 0.387254902 | BLCA |
| IL17D | loss | 88 | 0.215686275 | BLCA |
| IL2 | loss | 161 | 0.394607843 | BLCA |
| IL20RA | loss | 173 | 0.424019608 | BLCA |
| IL21 | loss | 161 | 0.394607843 | BLCA |
| IL21R | loss | 100 | 0.245098039 | BLCA |
| IL22RA2 | loss | 174 | 0.426470588 | BLCA |
| IL27RA | loss | 117 | 0.286764706 | BLCA |
| IL2RB | loss | 163 | 0.399509804 | BLCA |
| IL2RG | loss | 86 | 0.210784314 | BLCA |
| IL3 | loss | 189 | 0.463235294 | BLCA |
| IL4 | loss | 190 | 0.465686275 | BLCA |
| IL4R | loss | 100 | 0.245098039 | BLCA |
| IL5 | loss | 189 | 0.463235294 | BLCA |
| IL6ST | loss | 188 | 0.460784314 | BLCA |
| IL9 | loss | 191 | 0.468137255 | BLCA |
| IRF9 | loss | 119 | 0.291666667 | BLCA |
| JAK2 | loss | 175 | 0.428921569 | BLCA |
| JAK3 | loss | 113 | 0.276960784 | BLCA |
| LIF | loss | 136 | 0.333333333 | BLCA |
| OSM | loss | 136 | 0.333333333 | BLCA |
| PIAS1 | loss | 142 | 0.348039216 | BLCA |
| PIAS2 | loss | 170 | 0.416666667 | BLCA |
| PIAS4 | loss | 144 | 0.352941176 | BLCA |
| PIM1 | loss | 91 | 0.223039216 | BLCA |
| PTPN2 | loss | 90 | 0.220588235 | BLCA |
| SOCS1 | loss | 115 | 0.281862745 | BLCA |
| SOCS4 | loss | 125 | 0.306372549 | BLCA |
| SOCS6 | loss | 181 | 0.443627451 | BLCA |
| STAM2 | loss | 117 | 0.286764706 | BLCA |
| STAT1 | loss | 122 | 0.299019608 | BLCA |
| STAT4 | loss | 122 | 0.299019608 | BLCA |
| TSLP | loss | 198 | 0.485294118 | BLCA |
| TYK2 | loss | 118 | 0.289215686 | BLCA |
| AOX1 | loss | 231 | 0.213888889 | BRCA |
| BCL2 | loss | 336 | 0.311111111 | BRCA |
| CISH | loss | 348 | 0.322222222 | BRCA |
| CNTF | loss | 234 | 0.216666667 | BRCA |
| CNTFR | loss | 279 | 0.258333333 | BRCA |
| CSF2 | loss | 257 | 0.237962963 | BRCA |
| CSF2RB | loss | 495 | 0.458333333 | BRCA |
| CSF3 | loss | 284 | 0.262962963 | BRCA |
| CSF3R | loss | 320 | 0.296296296 | BRCA |
| EPOR | loss | 233 | 0.215740741 | BRCA |
| GFAP | loss | 364 | 0.337037037 | BRCA |
| IFNA1 | loss | 341 | 0.315740741 | BRCA |
| IFNA10 | loss | 337 | 0.312037037 | BRCA |
| IFNA13 | loss | 337 | 0.312037037 | BRCA |
| IFNA14 | loss | 338 | 0.312962963 | BRCA |
| IFNA16 | loss | 337 | 0.312037037 | BRCA |
| IFNA17 | loss | 337 | 0.312037037 | BRCA |
| IFNA2 | loss | 337 | 0.312037037 | BRCA |
| IFNA21 | loss | 335 | 0.310185185 | BRCA |
| IFNA4 | loss | 338 | 0.312962963 | BRCA |
| IFNA5 | loss | 338 | 0.312962963 | BRCA |
| IFNA6 | loss | 337 | 0.312037037 | BRCA |
| IFNA7 | loss | 337 | 0.312037037 | BRCA |
| IFNA8 | loss | 341 | 0.315740741 | BRCA |
| IFNB1 | loss | 335 | 0.310185185 | BRCA |
| IFNE | loss | 342 | 0.316666667 | BRCA |
| IFNGR1 | loss | 312 | 0.288888889 | BRCA |
| IFNK | loss | 317 | 0.293518519 | BRCA |
| IFNLR1 | loss | 437 | 0.40462963 | BRCA |
| IFNW1 | loss | 336 | 0.311111111 | BRCA |
| IL10RA | loss | 542 | 0.501851852 | BRCA |
| IL11RA | loss | 276 | 0.255555556 | BRCA |
| IL12B | loss | 236 | 0.218518519 | BRCA |
| IL12RB2 | loss | 315 | 0.291666667 | BRCA |
| IL13 | loss | 256 | 0.237037037 | BRCA |
| IL15 | loss | 304 | 0.281481481 | BRCA |
| IL17D | loss | 434 | 0.401851852 | BRCA |
| IL2 | loss | 308 | 0.285185185 | BRCA |
| IL20RA | loss | 314 | 0.290740741 | BRCA |
| IL21 | loss | 307 | 0.284259259 | BRCA |
| IL22RA1 | loss | 437 | 0.40462963 | BRCA |
| IL22RA2 | loss | 312 | 0.288888889 | BRCA |
| IL23R | loss | 314 | 0.290740741 | BRCA |
| IL2RB | loss | 491 | 0.45462963 | BRCA |
| IL3 | loss | 257 | 0.237962963 | BRCA |
| IL4 | loss | 256 | 0.237037037 | BRCA |
| IL5 | loss | 256 | 0.237037037 | BRCA |
| IL6ST | loss | 268 | 0.248148148 | BRCA |
| IL9 | loss | 249 | 0.230555556 | BRCA |
| IRF9 | loss | 248 | 0.22962963 | BRCA |
| JAK1 | loss | 325 | 0.300925926 | BRCA |
| JAK2 | loss | 324 | 0.3 | BRCA |
| LIF | loss | 467 | 0.432407407 | BRCA |
| MPL | loss | 292 | 0.27037037 | BRCA |
| OSM | loss | 467 | 0.432407407 | BRCA |
| PIAS1 | loss | 324 | 0.3 | BRCA |
| PIAS2 | loss | 344 | 0.318518519 | BRCA |
| PIAS4 | loss | 312 | 0.288888889 | BRCA |
| PTPN2 | loss | 301 | 0.278703704 | BRCA |
| SOCS4 | loss | 286 | 0.264814815 | BRCA |
| SOCS6 | loss | 350 | 0.324074074 | BRCA |
| SOCS7 | loss | 316 | 0.292592593 | BRCA |
| STAM2 | loss | 256 | 0.237037037 | BRCA |
| STAT1 | loss | 226 | 0.209259259 | BRCA |
| STAT3 | loss | 364 | 0.337037037 | BRCA |
| STAT4 | loss | 223 | 0.206481481 | BRCA |
| STAT5A | loss | 364 | 0.337037037 | BRCA |
| STAT5B | loss | 363 | 0.336111111 | BRCA |
| TSLP | loss | 268 | 0.248148148 | BRCA |
| TYK2 | loss | 226 | 0.209259259 | BRCA |
| AOX1 | loss | 62 | 0.210169492 | CESC |
| BCL2 | loss | 81 | 0.274576271 | CESC |
| CISH | loss | 122 | 0.413559322 | CESC |
| CNTF | loss | 66 | 0.223728814 | CESC |
| CSF2 | loss | 69 | 0.233898305 | CESC |
| CSF2RB | loss | 69 | 0.233898305 | CESC |
| EPOR | loss | 78 | 0.26440678 | CESC |
| IFNA1 | loss | 59 | 0.2 | CESC |
| IFNA13 | loss | 59 | 0.2 | CESC |
| IFNA2 | loss | 59 | 0.2 | CESC |
| IFNA6 | loss | 59 | 0.2 | CESC |
| IFNA8 | loss | 59 | 0.2 | CESC |
| IFNE | loss | 59 | 0.2 | CESC |
| IFNGR1 | loss | 89 | 0.301694915 | CESC |
| IL10RA | loss | 161 | 0.545762712 | CESC |
| IL12B | loss | 65 | 0.220338983 | CESC |
| IL12RB1 | loss | 71 | 0.240677966 | CESC |
| IL13 | loss | 69 | 0.233898305 | CESC |
| IL15 | loss | 103 | 0.349152542 | CESC |
| IL15RA | loss | 64 | 0.216949153 | CESC |
| IL17D | loss | 100 | 0.338983051 | CESC |
| IL2 | loss | 100 | 0.338983051 | CESC |
| IL20RA | loss | 89 | 0.301694915 | CESC |
| IL21 | loss | 100 | 0.338983051 | CESC |
| IL22RA2 | loss | 89 | 0.301694915 | CESC |
| IL27RA | loss | 72 | 0.244067797 | CESC |
| IL2RA | loss | 64 | 0.216949153 | CESC |
| IL2RB | loss | 69 | 0.233898305 | CESC |
| IL2RG | loss | 65 | 0.220338983 | CESC |
| IL3 | loss | 69 | 0.233898305 | CESC |
| IL4 | loss | 69 | 0.233898305 | CESC |
| IL5 | loss | 69 | 0.233898305 | CESC |
| IL5RA | loss | 108 | 0.366101695 | CESC |
| IL6ST | loss | 71 | 0.240677966 | CESC |
| IL9 | loss | 65 | 0.220338983 | CESC |
| JAK3 | loss | 72 | 0.244067797 | CESC |
| LIF | loss | 63 | 0.213559322 | CESC |
| OSM | loss | 63 | 0.213559322 | CESC |
| PIAS2 | loss | 75 | 0.254237288 | CESC |
| PIAS4 | loss | 101 | 0.342372881 | CESC |
| SOCS6 | loss | 81 | 0.274576271 | CESC |
| STAM | loss | 66 | 0.223728814 | CESC |
| TSLP | loss | 74 | 0.250847458 | CESC |
| TYK2 | loss | 78 | 0.26440678 | CESC |
| BCL2 | loss | 13 | 0.361111111 | CHOL |
| CISH | loss | 29 | 0.805555556 | CHOL |
| CNTF | loss | 10 | 0.277777778 | CHOL |
| CNTFR | loss | 16 | 0.444444444 | CHOL |
| CSF3R | loss | 13 | 0.361111111 | CHOL |
| FHL1 | loss | 15 | 0.416666667 | CHOL |
| IFNA1 | loss | 20 | 0.555555556 | CHOL |
| IFNA10 | loss | 19 | 0.527777778 | CHOL |
| IFNA13 | loss | 19 | 0.527777778 | CHOL |
| IFNA14 | loss | 19 | 0.527777778 | CHOL |
| IFNA16 | loss | 19 | 0.527777778 | CHOL |
| IFNA17 | loss | 19 | 0.527777778 | CHOL |
| IFNA2 | loss | 19 | 0.527777778 | CHOL |
| IFNA21 | loss | 19 | 0.527777778 | CHOL |
| IFNA4 | loss | 19 | 0.527777778 | CHOL |
| IFNA5 | loss | 19 | 0.527777778 | CHOL |
| IFNA6 | loss | 19 | 0.527777778 | CHOL |
| IFNA7 | loss | 19 | 0.527777778 | CHOL |
| IFNA8 | loss | 19 | 0.527777778 | CHOL |
| IFNAR1 | loss | 15 | 0.416666667 | CHOL |
| IFNAR2 | loss | 15 | 0.416666667 | CHOL |
| IFNB1 | loss | 19 | 0.527777778 | CHOL |
| IFNE | loss | 20 | 0.555555556 | CHOL |
| IFNGR1 | loss | 23 | 0.638888889 | CHOL |
| IFNGR2 | loss | 15 | 0.416666667 | CHOL |
| IFNK | loss | 17 | 0.472222222 | CHOL |
| IFNLR1 | loss | 29 | 0.805555556 | CHOL |
| IFNW1 | loss | 19 | 0.527777778 | CHOL |
| IL10RA | loss | 12 | 0.333333333 | CHOL |
| IL10RB | loss | 15 | 0.416666667 | CHOL |
| IL11RA | loss | 16 | 0.444444444 | CHOL |
| IL13RA1 | loss | 15 | 0.416666667 | CHOL |
| IL13RA2 | loss | 15 | 0.416666667 | CHOL |
| IL15 | loss | 14 | 0.388888889 | CHOL |
| IL17D | loss | 18 | 0.5 | CHOL |
| IL2 | loss | 16 | 0.444444444 | CHOL |
| IL20RA | loss | 23 | 0.638888889 | CHOL |
| IL21 | loss | 16 | 0.444444444 | CHOL |
| IL22RA1 | loss | 29 | 0.805555556 | CHOL |
| IL22RA2 | loss | 23 | 0.638888889 | CHOL |
| IL2RG | loss | 17 | 0.472222222 | CHOL |
| IL5RA | loss | 26 | 0.722222222 | CHOL |
| IRF9 | loss | 14 | 0.388888889 | CHOL |
| JAK2 | loss | 16 | 0.444444444 | CHOL |
| MPL | loss | 10 | 0.277777778 | CHOL |
| PIAS2 | loss | 12 | 0.333333333 | CHOL |
| PTPN11 | loss | 9 | 0.25 | CHOL |
| SOCS4 | loss | 16 | 0.444444444 | CHOL |
| SOCS6 | loss | 13 | 0.361111111 | CHOL |
| BCL2 | loss | 284 | 0.629711752 | COAD |
| CSF2 | loss | 101 | 0.223946785 | COAD |
| CSF2RB | loss | 147 | 0.32594235 | COAD |
| CSF3R | loss | 120 | 0.266075388 | COAD |
| IFNAR1 | loss | 124 | 0.274944568 | COAD |
| IFNAR2 | loss | 125 | 0.277161863 | COAD |
| IFNGR2 | loss | 122 | 0.270509978 | COAD |
| IFNLR1 | loss | 163 | 0.361419069 | COAD |
| IL10RB | loss | 124 | 0.274944568 | COAD |
| IL12RB2 | loss | 128 | 0.283813747 | COAD |
| IL13 | loss | 101 | 0.223946785 | COAD |
| IL15 | loss | 137 | 0.303769401 | COAD |
| IL2 | loss | 139 | 0.308203991 | COAD |
| IL21 | loss | 140 | 0.310421286 | COAD |
| IL22RA1 | loss | 163 | 0.361419069 | COAD |
| IL23R | loss | 128 | 0.283813747 | COAD |
| IL2RB | loss | 149 | 0.33037694 | COAD |
| IL3 | loss | 101 | 0.223946785 | COAD |
| IL4 | loss | 101 | 0.223946785 | COAD |
| IL5 | loss | 101 | 0.223946785 | COAD |
| IL6ST | loss | 97 | 0.215077605 | COAD |
| IL9 | loss | 97 | 0.215077605 | COAD |
| IRF9 | loss | 142 | 0.314855876 | COAD |
| JAK1 | loss | 127 | 0.281596452 | COAD |
| LIF | loss | 146 | 0.323725055 | COAD |
| MPL | loss | 110 | 0.243902439 | COAD |
| OSM | loss | 146 | 0.323725055 | COAD |
| PIAS1 | loss | 158 | 0.350332594 | COAD |
| PIAS2 | loss | 275 | 0.609756098 | COAD |
| PTPN2 | loss | 243 | 0.538802661 | COAD |
| SOCS4 | loss | 138 | 0.305986696 | COAD |
| SOCS6 | loss | 284 | 0.629711752 | COAD |
| TSLP | loss | 127 | 0.281596452 | COAD |
| BCL2 | loss | 112 | 0.608695652 | ESCA |
| CISH | loss | 123 | 0.668478261 | ESCA |
| CNTFR | loss | 89 | 0.483695652 | ESCA |
| CSF2 | loss | 95 | 0.516304348 | ESCA |
| CSF2RB | loss | 74 | 0.402173913 | ESCA |
| CSF3R | loss | 57 | 0.309782609 | ESCA |
| CTF1 | loss | 45 | 0.244565217 | ESCA |
| EPOR | loss | 73 | 0.39673913 | ESCA |
| GHR | loss | 37 | 0.201086957 | ESCA |
| IFNA1 | loss | 115 | 0.625 | ESCA |
| IFNA10 | loss | 114 | 0.619565217 | ESCA |
| IFNA13 | loss | 115 | 0.625 | ESCA |
| IFNA14 | loss | 114 | 0.619565217 | ESCA |
| IFNA16 | loss | 114 | 0.619565217 | ESCA |
| IFNA17 | loss | 114 | 0.619565217 | ESCA |
| IFNA2 | loss | 115 | 0.625 | ESCA |
| IFNA21 | loss | 114 | 0.619565217 | ESCA |
| IFNA4 | loss | 114 | 0.619565217 | ESCA |
| IFNA5 | loss | 115 | 0.625 | ESCA |
| IFNA6 | loss | 115 | 0.625 | ESCA |
| IFNA7 | loss | 114 | 0.619565217 | ESCA |
| IFNA8 | loss | 115 | 0.625 | ESCA |
| IFNAR1 | loss | 101 | 0.548913043 | ESCA |
| IFNAR2 | loss | 101 | 0.548913043 | ESCA |
| IFNB1 | loss | 113 | 0.614130435 | ESCA |
| IFNE | loss | 115 | 0.625 | ESCA |
| IFNGR1 | loss | 42 | 0.22826087 | ESCA |
| IFNGR2 | loss | 100 | 0.543478261 | ESCA |
| IFNK | loss | 102 | 0.554347826 | ESCA |
| IFNL1 | loss | 48 | 0.260869565 | ESCA |
| IFNL2 | loss | 47 | 0.255434783 | ESCA |
| IFNL3 | loss | 47 | 0.255434783 | ESCA |
| IFNLR1 | loss | 67 | 0.364130435 | ESCA |
| IFNW1 | loss | 114 | 0.619565217 | ESCA |
| IL10RA | loss | 75 | 0.407608696 | ESCA |
| IL10RB | loss | 101 | 0.548913043 | ESCA |
| IL11 | loss | 46 | 0.25 | ESCA |
| IL11RA | loss | 89 | 0.483695652 | ESCA |
| IL12B | loss | 86 | 0.467391304 | ESCA |
| IL12RB1 | loss | 62 | 0.336956522 | ESCA |
| IL12RB2 | loss | 55 | 0.298913043 | ESCA |
| IL13 | loss | 95 | 0.516304348 | ESCA |
| IL15 | loss | 83 | 0.451086957 | ESCA |
| IL15RA | loss | 49 | 0.266304348 | ESCA |
| IL17D | loss | 76 | 0.413043478 | ESCA |
| IL2 | loss | 92 | 0.5 | ESCA |
| IL20RA | loss | 42 | 0.22826087 | ESCA |
| IL21 | loss | 92 | 0.5 | ESCA |
| IL21R | loss | 50 | 0.27173913 | ESCA |
| IL22RA1 | loss | 67 | 0.364130435 | ESCA |
| IL22RA2 | loss | 42 | 0.22826087 | ESCA |
| IL23A | loss | 38 | 0.206521739 | ESCA |
| IL23R | loss | 55 | 0.298913043 | ESCA |
| IL27RA | loss | 66 | 0.358695652 | ESCA |
| IL2RA | loss | 49 | 0.266304348 | ESCA |
| IL2RB | loss | 73 | 0.39673913 | ESCA |
| IL3 | loss | 95 | 0.516304348 | ESCA |
| IL4 | loss | 96 | 0.52173913 | ESCA |
| IL4R | loss | 50 | 0.27173913 | ESCA |
| IL5 | loss | 95 | 0.516304348 | ESCA |
| IL5RA | loss | 111 | 0.60326087 | ESCA |
| IL6ST | loss | 107 | 0.581521739 | ESCA |
| IL9 | loss | 91 | 0.494565217 | ESCA |
| IRF9 | loss | 47 | 0.255434783 | ESCA |
| JAK1 | loss | 55 | 0.298913043 | ESCA |
| JAK2 | loss | 107 | 0.581521739 | ESCA |
| JAK3 | loss | 63 | 0.342391304 | ESCA |
| LIF | loss | 73 | 0.39673913 | ESCA |
| MPL | loss | 47 | 0.255434783 | ESCA |
| OSM | loss | 73 | 0.39673913 | ESCA |
| PIAS1 | loss | 56 | 0.304347826 | ESCA |
| PIAS2 | loss | 109 | 0.592391304 | ESCA |
| PIAS4 | loss | 88 | 0.47826087 | ESCA |
| PRL | loss | 52 | 0.282608696 | ESCA |
| PTPN11 | loss | 45 | 0.244565217 | ESCA |
| PTPN2 | loss | 64 | 0.347826087 | ESCA |
| SOCS1 | loss | 52 | 0.282608696 | ESCA |
| SOCS2 | loss | 48 | 0.260869565 | ESCA |
| SOCS4 | loss | 45 | 0.244565217 | ESCA |
| SOCS6 | loss | 112 | 0.608695652 | ESCA |
| STAM | loss | 53 | 0.288043478 | ESCA |
| STAT2 | loss | 38 | 0.206521739 | ESCA |
| STAT6 | loss | 39 | 0.211956522 | ESCA |
| TSLP | loss | 100 | 0.543478261 | ESCA |
| TYK2 | loss | 78 | 0.423913043 | ESCA |
| CNTFR | loss | 182 | 0.31542461 | GBM |
| CSF2RB | loss | 197 | 0.341421144 | GBM |
| IFNA1 | loss | 408 | 0.707105719 | GBM |
| IFNA10 | loss | 391 | 0.677642981 | GBM |
| IFNA13 | loss | 403 | 0.698440208 | GBM |
| IFNA14 | loss | 395 | 0.68457539 | GBM |
| IFNA16 | loss | 391 | 0.677642981 | GBM |
| IFNA17 | loss | 393 | 0.681109185 | GBM |
| IFNA2 | loss | 402 | 0.696707106 | GBM |
| IFNA21 | loss | 390 | 0.675909879 | GBM |
| IFNA4 | loss | 392 | 0.679376083 | GBM |
| IFNA5 | loss | 400 | 0.693240901 | GBM |
| IFNA6 | loss | 403 | 0.698440208 | GBM |
| IFNA7 | loss | 391 | 0.677642981 | GBM |
| IFNA8 | loss | 404 | 0.70017331 | GBM |
| IFNB1 | loss | 375 | 0.649913345 | GBM |
| IFNE | loss | 409 | 0.708838821 | GBM |
| IFNGR1 | loss | 165 | 0.285961872 | GBM |
| IFNK | loss | 293 | 0.50779896 | GBM |
| IFNW1 | loss | 386 | 0.66897747 | GBM |
| IL11RA | loss | 177 | 0.306759099 | GBM |
| IL15RA | loss | 470 | 0.814558059 | GBM |
| IL17D | loss | 186 | 0.322357019 | GBM |
| IL20RA | loss | 166 | 0.287694974 | GBM |
| IL22RA2 | loss | 166 | 0.287694974 | GBM |
| IL2RA | loss | 471 | 0.816291161 | GBM |
| IL2RB | loss | 195 | 0.337954939 | GBM |
| IRF9 | loss | 174 | 0.301559792 | GBM |
| JAK2 | loss | 249 | 0.431542461 | GBM |
| LIF | loss | 196 | 0.339688042 | GBM |
| OSM | loss | 196 | 0.339688042 | GBM |
| SOCS4 | loss | 164 | 0.284228769 | GBM |
| STAM | loss | 473 | 0.819757366 | GBM |
| CNTFR | loss | 269 | 0.246788991 | GBMLGG |
| CSF2RB | loss | 258 | 0.236697248 | GBMLGG |
| IFNA1 | loss | 578 | 0.530275229 | GBMLGG |
| IFNA10 | loss | 558 | 0.511926606 | GBMLGG |
| IFNA13 | loss | 572 | 0.524770642 | GBMLGG |
| IFNA14 | loss | 562 | 0.51559633 | GBMLGG |
| IFNA16 | loss | 558 | 0.511926606 | GBMLGG |
| IFNA17 | loss | 560 | 0.513761468 | GBMLGG |
| IFNA2 | loss | 571 | 0.523853211 | GBMLGG |
| IFNA21 | loss | 556 | 0.510091743 | GBMLGG |
| IFNA4 | loss | 559 | 0.512844037 | GBMLGG |
| IFNA5 | loss | 569 | 0.522018349 | GBMLGG |
| IFNA6 | loss | 572 | 0.524770642 | GBMLGG |
| IFNA7 | loss | 558 | 0.511926606 | GBMLGG |
| IFNA8 | loss | 573 | 0.525688073 | GBMLGG |
| IFNB1 | loss | 541 | 0.496330275 | GBMLGG |
| IFNE | loss | 579 | 0.531192661 | GBMLGG |
| IFNGR1 | loss | 244 | 0.223853211 | GBMLGG |
| IFNK | loss | 430 | 0.394495413 | GBMLGG |
| IFNL1 | loss | 274 | 0.251376147 | GBMLGG |
| IFNL2 | loss | 274 | 0.251376147 | GBMLGG |
| IFNL3 | loss | 275 | 0.252293578 | GBMLGG |
| IFNLR1 | loss | 245 | 0.224770642 | GBMLGG |
| IFNW1 | loss | 552 | 0.506422018 | GBMLGG |
| IL11 | loss | 365 | 0.334862385 | GBMLGG |
| IL11RA | loss | 264 | 0.242201835 | GBMLGG |
| IL15RA | loss | 553 | 0.50733945 | GBMLGG |
| IL17D | loss | 307 | 0.281651376 | GBMLGG |
| IL20RA | loss | 246 | 0.225688073 | GBMLGG |
| IL22RA1 | loss | 246 | 0.225688073 | GBMLGG |
| IL22RA2 | loss | 245 | 0.224770642 | GBMLGG |
| IL2RA | loss | 554 | 0.508256881 | GBMLGG |
| IL2RB | loss | 257 | 0.235779817 | GBMLGG |
| IRF9 | loss | 244 | 0.223853211 | GBMLGG |
| JAK2 | loss | 395 | 0.362385321 | GBMLGG |
| LIF | loss | 251 | 0.230275229 | GBMLGG |
| OSM | loss | 251 | 0.230275229 | GBMLGG |
| SOCS4 | loss | 254 | 0.233027523 | GBMLGG |
| STAM | loss | 554 | 0.508256881 | GBMLGG |
| BCL2 | loss | 279 | 0.534482759 | HNSC |
| CISH | loss | 377 | 0.722222222 | HNSC |
| CNTFR | loss | 164 | 0.314176245 | HNSC |
| CSF2 | loss | 205 | 0.392720307 | HNSC |
| EPOR | loss | 111 | 0.212643678 | HNSC |
| IFNA1 | loss | 237 | 0.454022989 | HNSC |
| IFNA10 | loss | 235 | 0.450191571 | HNSC |
| IFNA13 | loss | 236 | 0.45210728 | HNSC |
| IFNA14 | loss | 236 | 0.45210728 | HNSC |
| IFNA16 | loss | 235 | 0.450191571 | HNSC |
| IFNA17 | loss | 235 | 0.450191571 | HNSC |
| IFNA2 | loss | 237 | 0.454022989 | HNSC |
| IFNA21 | loss | 234 | 0.448275862 | HNSC |
| IFNA4 | loss | 235 | 0.450191571 | HNSC |
| IFNA5 | loss | 235 | 0.450191571 | HNSC |
| IFNA6 | loss | 235 | 0.450191571 | HNSC |
| IFNA7 | loss | 235 | 0.450191571 | HNSC |
| IFNA8 | loss | 237 | 0.454022989 | HNSC |
| IFNAR1 | loss | 188 | 0.360153257 | HNSC |
| IFNAR2 | loss | 188 | 0.360153257 | HNSC |
| IFNB1 | loss | 234 | 0.448275862 | HNSC |
| IFNE | loss | 237 | 0.454022989 | HNSC |
| IFNGR2 | loss | 186 | 0.356321839 | HNSC |
| IFNK | loss | 217 | 0.415708812 | HNSC |
| IFNW1 | loss | 234 | 0.448275862 | HNSC |
| IL10RA | loss | 245 | 0.469348659 | HNSC |
| IL10RB | loss | 188 | 0.360153257 | HNSC |
| IL11RA | loss | 164 | 0.314176245 | HNSC |
| IL12B | loss | 197 | 0.377394636 | HNSC |
| IL12RB1 | loss | 105 | 0.201149425 | HNSC |
| IL13 | loss | 207 | 0.396551724 | HNSC |
| IL15 | loss | 163 | 0.312260536 | HNSC |
| IL15RA | loss | 163 | 0.312260536 | HNSC |
| IL17D | loss | 232 | 0.444444444 | HNSC |
| IL2 | loss | 156 | 0.298850575 | HNSC |
| IL21 | loss | 156 | 0.298850575 | HNSC |
| IL2RA | loss | 163 | 0.312260536 | HNSC |
| IL3 | loss | 205 | 0.392720307 | HNSC |
| IL4 | loss | 207 | 0.396551724 | HNSC |
| IL5 | loss | 207 | 0.396551724 | HNSC |
| IL5RA | loss | 350 | 0.670498084 | HNSC |
| IL6ST | loss | 201 | 0.385057471 | HNSC |
| IL9 | loss | 201 | 0.385057471 | HNSC |
| JAK2 | loss | 215 | 0.411877395 | HNSC |
| JAK3 | loss | 106 | 0.203065134 | HNSC |
| PIAS1 | loss | 112 | 0.214559387 | HNSC |
| PIAS2 | loss | 247 | 0.473180077 | HNSC |
| PIAS4 | loss | 154 | 0.295019157 | HNSC |
| SOCS6 | loss | 277 | 0.530651341 | HNSC |
| STAM | loss | 169 | 0.323754789 | HNSC |
| TSLP | loss | 208 | 0.398467433 | HNSC |
| TYK2 | loss | 119 | 0.227969349 | HNSC |
| AOX1 | loss | 47 | 0.712121212 | KICH |
| CDKN1A | loss | 51 | 0.772727273 | KICH |
| CSF3 | loss | 50 | 0.757575758 | KICH |
| CSF3R | loss | 53 | 0.803030303 | KICH |
| CSH1 | loss | 50 | 0.757575758 | KICH |
| CSH2 | loss | 50 | 0.757575758 | KICH |
| FHL1 | loss | 38 | 0.575757576 | KICH |
| GFAP | loss | 50 | 0.757575758 | KICH |
| GH1 | loss | 50 | 0.757575758 | KICH |
| GH2 | loss | 50 | 0.757575758 | KICH |
| IFNAR1 | loss | 35 | 0.53030303 | KICH |
| IFNAR2 | loss | 35 | 0.53030303 | KICH |
| IFNGR1 | loss | 51 | 0.772727273 | KICH |
| IFNGR2 | loss | 35 | 0.53030303 | KICH |
| IFNLR1 | loss | 53 | 0.803030303 | KICH |
| IL10 | loss | 51 | 0.772727273 | KICH |
| IL10RB | loss | 35 | 0.53030303 | KICH |
| IL12RB2 | loss | 53 | 0.803030303 | KICH |
| IL13RA1 | loss | 39 | 0.590909091 | KICH |
| IL13RA2 | loss | 39 | 0.590909091 | KICH |
| IL15RA | loss | 48 | 0.727272727 | KICH |
| IL17D | loss | 44 | 0.666666667 | KICH |
| IL19 | loss | 51 | 0.772727273 | KICH |
| IL20 | loss | 51 | 0.772727273 | KICH |
| IL20RA | loss | 51 | 0.772727273 | KICH |
| IL22RA1 | loss | 53 | 0.803030303 | KICH |
| IL22RA2 | loss | 51 | 0.772727273 | KICH |
| IL23R | loss | 53 | 0.803030303 | KICH |
| IL24 | loss | 51 | 0.772727273 | KICH |
| IL2RA | loss | 48 | 0.727272727 | KICH |
| IL2RG | loss | 40 | 0.606060606 | KICH |
| IL6R | loss | 50 | 0.757575758 | KICH |
| JAK1 | loss | 53 | 0.803030303 | KICH |
| MCL1 | loss | 50 | 0.757575758 | KICH |
| MPL | loss | 53 | 0.803030303 | KICH |
| PIAS3 | loss | 52 | 0.787878788 | KICH |
| PIM1 | loss | 51 | 0.772727273 | KICH |
| PRL | loss | 51 | 0.772727273 | KICH |
| SOCS3 | loss | 48 | 0.727272727 | KICH |
| SOCS5 | loss | 46 | 0.696969697 | KICH |
| SOCS7 | loss | 50 | 0.757575758 | KICH |
| STAM | loss | 48 | 0.727272727 | KICH |
| STAM2 | loss | 46 | 0.696969697 | KICH |
| STAT1 | loss | 47 | 0.712121212 | KICH |
| STAT3 | loss | 50 | 0.757575758 | KICH |
| STAT4 | loss | 47 | 0.712121212 | KICH |
| STAT5A | loss | 50 | 0.757575758 | KICH |
| STAT5B | loss | 50 | 0.757575758 | KICH |
| CDKN1A | loss | 177 | 0.200680272 | KIPAN |
| CISH | loss | 499 | 0.565759637 | KIPAN |
| CNTFR | loss | 188 | 0.213151927 | KIPAN |
| CSF3R | loss | 178 | 0.201814059 | KIPAN |
| IFNA1 | loss | 202 | 0.229024943 | KIPAN |
| IFNA10 | loss | 201 | 0.227891156 | KIPAN |
| IFNA13 | loss | 202 | 0.229024943 | KIPAN |
| IFNA14 | loss | 201 | 0.227891156 | KIPAN |
| IFNA16 | loss | 201 | 0.227891156 | KIPAN |
| IFNA17 | loss | 201 | 0.227891156 | KIPAN |
| IFNA2 | loss | 202 | 0.229024943 | KIPAN |
| IFNA21 | loss | 201 | 0.227891156 | KIPAN |
| IFNA4 | loss | 201 | 0.227891156 | KIPAN |
| IFNA5 | loss | 202 | 0.229024943 | KIPAN |
| IFNA6 | loss | 202 | 0.229024943 | KIPAN |
| IFNA7 | loss | 201 | 0.227891156 | KIPAN |
| IFNA8 | loss | 202 | 0.229024943 | KIPAN |
| IFNB1 | loss | 201 | 0.227891156 | KIPAN |
| IFNE | loss | 203 | 0.23015873 | KIPAN |
| IFNGR1 | loss | 231 | 0.261904762 | KIPAN |
| IFNK | loss | 196 | 0.222222222 | KIPAN |
| IFNLR1 | loss | 200 | 0.22675737 | KIPAN |
| IFNW1 | loss | 201 | 0.227891156 | KIPAN |
| IL11RA | loss | 188 | 0.213151927 | KIPAN |
| IL20RA | loss | 232 | 0.263038549 | KIPAN |
| IL22RA1 | loss | 200 | 0.22675737 | KIPAN |
| IL22RA2 | loss | 231 | 0.261904762 | KIPAN |
| IL5RA | loss | 489 | 0.554421769 | KIPAN |
| IRF9 | loss | 258 | 0.292517007 | KIPAN |
| JAK2 | loss | 193 | 0.218820862 | KIPAN |
| PRL | loss | 178 | 0.201814059 | KIPAN |
| SOCS4 | loss | 269 | 0.304988662 | KIPAN |
| CISH | loss | 463 | 0.876893939 | KIRC |
| CNTFR | loss | 146 | 0.276515152 | KIRC |
| IFNA1 | loss | 153 | 0.289772727 | KIRC |
| IFNA10 | loss | 153 | 0.289772727 | KIRC |
| IFNA13 | loss | 153 | 0.289772727 | KIRC |
| IFNA14 | loss | 153 | 0.289772727 | KIRC |
| IFNA16 | loss | 153 | 0.289772727 | KIRC |
| IFNA17 | loss | 153 | 0.289772727 | KIRC |
| IFNA2 | loss | 153 | 0.289772727 | KIRC |
| IFNA21 | loss | 153 | 0.289772727 | KIRC |
| IFNA4 | loss | 153 | 0.289772727 | KIRC |
| IFNA5 | loss | 153 | 0.289772727 | KIRC |
| IFNA6 | loss | 153 | 0.289772727 | KIRC |
| IFNA7 | loss | 153 | 0.289772727 | KIRC |
| IFNA8 | loss | 153 | 0.289772727 | KIRC |
| IFNB1 | loss | 153 | 0.289772727 | KIRC |
| IFNE | loss | 154 | 0.291666667 | KIRC |
| IFNGR1 | loss | 149 | 0.28219697 | KIRC |
| IFNK | loss | 152 | 0.287878788 | KIRC |
| IFNW1 | loss | 153 | 0.289772727 | KIRC |
| IL11RA | loss | 146 | 0.276515152 | KIRC |
| IL20RA | loss | 150 | 0.284090909 | KIRC |
| IL22RA2 | loss | 149 | 0.28219697 | KIRC |
| IL5RA | loss | 456 | 0.863636364 | KIRC |
| IRF9 | loss | 206 | 0.390151515 | KIRC |
| JAK2 | loss | 149 | 0.28219697 | KIRC |
| SOCS4 | loss | 219 | 0.414772727 | KIRC |
| CSF2RB | loss | 59 | 0.204861111 | KIRP |
| IL2RB | loss | 60 | 0.208333333 | KIRP |
| LIF | loss | 63 | 0.21875 | KIRP |
| OSM | loss | 63 | 0.21875 | KIRP |
| BCL2 | loss | 91 | 0.245945946 | LIHC |
| CNTFR | loss | 101 | 0.272972973 | LIHC |
| CSF3R | loss | 116 | 0.313513514 | LIHC |
| CTF1 | loss | 91 | 0.245945946 | LIHC |
| EPOR | loss | 77 | 0.208108108 | LIHC |
| IFNA1 | loss | 133 | 0.359459459 | LIHC |
| IFNA10 | loss | 129 | 0.348648649 | LIHC |
| IFNA13 | loss | 130 | 0.351351351 | LIHC |
| IFNA14 | loss | 129 | 0.348648649 | LIHC |
| IFNA16 | loss | 129 | 0.348648649 | LIHC |
| IFNA17 | loss | 129 | 0.348648649 | LIHC |
| IFNA2 | loss | 130 | 0.351351351 | LIHC |
| IFNA21 | loss | 128 | 0.345945946 | LIHC |
| IFNA4 | loss | 128 | 0.345945946 | LIHC |
| IFNA5 | loss | 129 | 0.348648649 | LIHC |
| IFNA6 | loss | 129 | 0.348648649 | LIHC |
| IFNA7 | loss | 129 | 0.348648649 | LIHC |
| IFNA8 | loss | 131 | 0.354054054 | LIHC |
| IFNAR1 | loss | 108 | 0.291891892 | LIHC |
| IFNAR2 | loss | 108 | 0.291891892 | LIHC |
| IFNB1 | loss | 129 | 0.348648649 | LIHC |
| IFNE | loss | 134 | 0.362162162 | LIHC |
| IFNGR1 | loss | 129 | 0.348648649 | LIHC |
| IFNGR2 | loss | 108 | 0.291891892 | LIHC |
| IFNK | loss | 119 | 0.321621622 | LIHC |
| IFNLR1 | loss | 137 | 0.37027027 | LIHC |
| IFNW1 | loss | 128 | 0.345945946 | LIHC |
| IL10RA | loss | 92 | 0.248648649 | LIHC |
| IL10RB | loss | 108 | 0.291891892 | LIHC |
| IL11RA | loss | 102 | 0.275675676 | LIHC |
| IL12RB2 | loss | 91 | 0.245945946 | LIHC |
| IL15 | loss | 154 | 0.416216216 | LIHC |
| IL17D | loss | 127 | 0.343243243 | LIHC |
| IL2 | loss | 157 | 0.424324324 | LIHC |
| IL20RA | loss | 129 | 0.348648649 | LIHC |
| IL21 | loss | 155 | 0.418918919 | LIHC |
| IL21R | loss | 91 | 0.245945946 | LIHC |
| IL22RA1 | loss | 136 | 0.367567568 | LIHC |
| IL22RA2 | loss | 129 | 0.348648649 | LIHC |
| IL23R | loss | 91 | 0.245945946 | LIHC |
| IL2RG | loss | 75 | 0.202702703 | LIHC |
| IL4R | loss | 91 | 0.245945946 | LIHC |
| IRF9 | loss | 82 | 0.221621622 | LIHC |
| JAK1 | loss | 94 | 0.254054054 | LIHC |
| JAK2 | loss | 120 | 0.324324324 | LIHC |
| LIF | loss | 74 | 0.2 | LIHC |
| MPL | loss | 104 | 0.281081081 | LIHC |
| OSM | loss | 74 | 0.2 | LIHC |
| PIAS2 | loss | 92 | 0.248648649 | LIHC |
| PIAS4 | loss | 88 | 0.237837838 | LIHC |
| PTPN6 | loss | 76 | 0.205405405 | LIHC |
| SOCS1 | loss | 102 | 0.275675676 | LIHC |
| SOCS4 | loss | 107 | 0.289189189 | LIHC |
| SOCS6 | loss | 89 | 0.240540541 | LIHC |
| TYK2 | loss | 83 | 0.224324324 | LIHC |
| BCL2 | loss | 249 | 0.48255814 | LUAD |
| CISH | loss | 242 | 0.468992248 | LUAD |
| CNTFR | loss | 220 | 0.426356589 | LUAD |
| CSF2 | loss | 208 | 0.403100775 | LUAD |
| CSF2RB | loss | 226 | 0.437984496 | LUAD |
| EPOR | loss | 269 | 0.521317829 | LUAD |
| IFNA1 | loss | 286 | 0.554263566 | LUAD |
| IFNA10 | loss | 285 | 0.552325581 | LUAD |
| IFNA13 | loss | 285 | 0.552325581 | LUAD |
| IFNA14 | loss | 285 | 0.552325581 | LUAD |
| IFNA16 | loss | 285 | 0.552325581 | LUAD |
| IFNA17 | loss | 285 | 0.552325581 | LUAD |
| IFNA2 | loss | 285 | 0.552325581 | LUAD |
| IFNA21 | loss | 284 | 0.550387597 | LUAD |
| IFNA4 | loss | 285 | 0.552325581 | LUAD |
| IFNA5 | loss | 285 | 0.552325581 | LUAD |
| IFNA6 | loss | 285 | 0.552325581 | LUAD |
| IFNA7 | loss | 285 | 0.552325581 | LUAD |
| IFNA8 | loss | 285 | 0.552325581 | LUAD |
| IFNAR1 | loss | 168 | 0.325581395 | LUAD |
| IFNAR2 | loss | 168 | 0.325581395 | LUAD |
| IFNB1 | loss | 284 | 0.550387597 | LUAD |
| IFNE | loss | 287 | 0.55620155 | LUAD |
| IFNGR1 | loss | 249 | 0.48255814 | LUAD |
| IFNGR2 | loss | 167 | 0.323643411 | LUAD |
| IFNK | loss | 267 | 0.51744186 | LUAD |
| IFNL1 | loss | 129 | 0.25 | LUAD |
| IFNL2 | loss | 130 | 0.251937984 | LUAD |
| IFNL3 | loss | 131 | 0.253875969 | LUAD |
| IFNLR1 | loss | 127 | 0.246124031 | LUAD |
| IFNW1 | loss | 283 | 0.548449612 | LUAD |
| IL10RA | loss | 114 | 0.220930233 | LUAD |
| IL10RB | loss | 168 | 0.325581395 | LUAD |
| IL11 | loss | 172 | 0.333333333 | LUAD |
| IL11RA | loss | 220 | 0.426356589 | LUAD |
| IL12A | loss | 115 | 0.222868217 | LUAD |
| IL12B | loss | 175 | 0.339147287 | LUAD |
| IL12RB1 | loss | 242 | 0.468992248 | LUAD |
| IL12RB2 | loss | 120 | 0.23255814 | LUAD |
| IL13 | loss | 207 | 0.401162791 | LUAD |
| IL15 | loss | 161 | 0.312015504 | LUAD |
| IL15RA | loss | 120 | 0.23255814 | LUAD |
| IL17D | loss | 294 | 0.569767442 | LUAD |
| IL2 | loss | 167 | 0.323643411 | LUAD |
| IL20RA | loss | 248 | 0.480620155 | LUAD |
| IL20RB | loss | 119 | 0.230620155 | LUAD |
| IL21 | loss | 169 | 0.32751938 | LUAD |
| IL22RA1 | loss | 126 | 0.244186047 | LUAD |
| IL22RA2 | loss | 249 | 0.48255814 | LUAD |
| IL23R | loss | 120 | 0.23255814 | LUAD |
| IL27RA | loss | 269 | 0.521317829 | LUAD |
| IL2RA | loss | 120 | 0.23255814 | LUAD |
| IL2RB | loss | 225 | 0.436046512 | LUAD |
| IL3 | loss | 208 | 0.403100775 | LUAD |
| IL4 | loss | 207 | 0.401162791 | LUAD |
| IL5 | loss | 208 | 0.403100775 | LUAD |
| IL5RA | loss | 229 | 0.44379845 | LUAD |
| IL6ST | loss | 162 | 0.313953488 | LUAD |
| IL9 | loss | 208 | 0.403100775 | LUAD |
| JAK1 | loss | 123 | 0.238372093 | LUAD |
| JAK2 | loss | 271 | 0.525193798 | LUAD |
| JAK3 | loss | 242 | 0.468992248 | LUAD |
| LIF | loss | 217 | 0.420542636 | LUAD |
| OSM | loss | 217 | 0.420542636 | LUAD |
| PIAS1 | loss | 241 | 0.467054264 | LUAD |
| PIAS2 | loss | 247 | 0.478682171 | LUAD |
| PIAS4 | loss | 280 | 0.542635659 | LUAD |
| PTPN11 | loss | 125 | 0.242248062 | LUAD |
| PTPN2 | loss | 161 | 0.312015504 | LUAD |
| PTPN6 | loss | 126 | 0.244186047 | LUAD |
| SOCS2 | loss | 124 | 0.240310078 | LUAD |
| SOCS4 | loss | 117 | 0.226744186 | LUAD |
| SOCS6 | loss | 249 | 0.48255814 | LUAD |
| STAM | loss | 115 | 0.222868217 | LUAD |
| THPO | loss | 132 | 0.255813953 | LUAD |
| TSLP | loss | 207 | 0.401162791 | LUAD |
| TYK2 | loss | 268 | 0.519379845 | LUAD |
| BCL2 | loss | 213 | 0.425149701 | LUSC |
| CDKN1A | loss | 117 | 0.233532934 | LUSC |
| CISH | loss | 437 | 0.872255489 | LUSC |
| CNTF | loss | 107 | 0.213572854 | LUSC |
| CNTFR | loss | 258 | 0.51497006 | LUSC |
| CSF2 | loss | 385 | 0.768463074 | LUSC |
| CSF3 | loss | 112 | 0.223552894 | LUSC |
| CSF3R | loss | 187 | 0.373253493 | LUSC |
| CTF1 | loss | 130 | 0.259481038 | LUSC |
| EPOR | loss | 194 | 0.387225549 | LUSC |
| FHL1 | loss | 102 | 0.203592814 | LUSC |
| GFAP | loss | 101 | 0.201596806 | LUSC |
| IFNA1 | loss | 359 | 0.716566866 | LUSC |
| IFNA10 | loss | 356 | 0.710578842 | LUSC |
| IFNA13 | loss | 357 | 0.71257485 | LUSC |
| IFNA14 | loss | 357 | 0.71257485 | LUSC |
| IFNA16 | loss | 356 | 0.710578842 | LUSC |
| IFNA17 | loss | 356 | 0.710578842 | LUSC |
| IFNA2 | loss | 356 | 0.710578842 | LUSC |
| IFNA21 | loss | 354 | 0.706586826 | LUSC |
| IFNA4 | loss | 355 | 0.708582834 | LUSC |
| IFNA5 | loss | 357 | 0.71257485 | LUSC |
| IFNA6 | loss | 357 | 0.71257485 | LUSC |
| IFNA7 | loss | 356 | 0.710578842 | LUSC |
| IFNA8 | loss | 358 | 0.714570858 | LUSC |
| IFNAR1 | loss | 262 | 0.522954092 | LUSC |
| IFNAR2 | loss | 263 | 0.5249501 | LUSC |
| IFNB1 | loss | 353 | 0.704590818 | LUSC |
| IFNE | loss | 360 | 0.718562874 | LUSC |
| IFNGR1 | loss | 120 | 0.239520958 | LUSC |
| IFNGR2 | loss | 261 | 0.520958084 | LUSC |
| IFNK | loss | 326 | 0.650698603 | LUSC |
| IFNLR1 | loss | 226 | 0.451097804 | LUSC |
| IFNW1 | loss | 354 | 0.706586826 | LUSC |
| IL10RA | loss | 175 | 0.349301397 | LUSC |
| IL10RB | loss | 261 | 0.520958084 | LUSC |
| IL11 | loss | 127 | 0.253493014 | LUSC |
| IL11RA | loss | 257 | 0.512974052 | LUSC |
| IL12B | loss | 369 | 0.736526946 | LUSC |
| IL12RB1 | loss | 156 | 0.311377246 | LUSC |
| IL12RB2 | loss | 196 | 0.391217565 | LUSC |
| IL13 | loss | 383 | 0.764471058 | LUSC |
| IL15 | loss | 311 | 0.620758483 | LUSC |
| IL15RA | loss | 210 | 0.419161677 | LUSC |
| IL17D | loss | 340 | 0.678642715 | LUSC |
| IL2 | loss | 307 | 0.612774451 | LUSC |
| IL20RA | loss | 120 | 0.239520958 | LUSC |
| IL21 | loss | 307 | 0.612774451 | LUSC |
| IL21R | loss | 131 | 0.261477046 | LUSC |
| IL22RA1 | loss | 226 | 0.451097804 | LUSC |
| IL22RA2 | loss | 120 | 0.239520958 | LUSC |
| IL23R | loss | 196 | 0.391217565 | LUSC |
| IL27RA | loss | 175 | 0.349301397 | LUSC |
| IL2RA | loss | 210 | 0.419161677 | LUSC |
| IL2RG | loss | 128 | 0.255489022 | LUSC |
| IL3 | loss | 385 | 0.768463074 | LUSC |
| IL4 | loss | 383 | 0.764471058 | LUSC |
| IL4R | loss | 132 | 0.263473054 | LUSC |
| IL5 | loss | 383 | 0.764471058 | LUSC |
| IL5RA | loss | 386 | 0.770459082 | LUSC |
| IL6ST | loss | 364 | 0.726546906 | LUSC |
| IL9 | loss | 379 | 0.756487026 | LUSC |
| IRF9 | loss | 177 | 0.353293413 | LUSC |
| JAK1 | loss | 196 | 0.391217565 | LUSC |
| JAK2 | loss | 328 | 0.654690619 | LUSC |
| JAK3 | loss | 158 | 0.315369261 | LUSC |
| MPL | loss | 167 | 0.333333333 | LUSC |
| PIAS1 | loss | 134 | 0.26746507 | LUSC |
| PIAS2 | loss | 201 | 0.401197605 | LUSC |
| PIAS4 | loss | 219 | 0.437125749 | LUSC |
| PIM1 | loss | 116 | 0.231536926 | LUSC |
| PRL | loss | 129 | 0.25748503 | LUSC |
| PTPN2 | loss | 128 | 0.255489022 | LUSC |
| SOCS1 | loss | 151 | 0.301397206 | LUSC |
| SOCS4 | loss | 184 | 0.367265469 | LUSC |
| SOCS6 | loss | 216 | 0.431137725 | LUSC |
| SOCS7 | loss | 120 | 0.239520958 | LUSC |
| STAM | loss | 206 | 0.411177645 | LUSC |
| STAT3 | loss | 116 | 0.231536926 | LUSC |
| STAT5A | loss | 116 | 0.231536926 | LUSC |
| STAT5B | loss | 116 | 0.231536926 | LUSC |
| TSLP | loss | 386 | 0.770459082 | LUSC |
| TYK2 | loss | 194 | 0.387225549 | LUSC |
| BCL2 | loss | 124 | 0.673913043 | PAAD |
| CDKN1A | loss | 61 | 0.331521739 | PAAD |
| CISH | loss | 51 | 0.277173913 | PAAD |
| CNTFR | loss | 59 | 0.320652174 | PAAD |
| CSF2RB | loss | 46 | 0.25 | PAAD |
| CSF3R | loss | 46 | 0.25 | PAAD |
| CSH1 | loss | 40 | 0.217391304 | PAAD |
| CSH2 | loss | 40 | 0.217391304 | PAAD |
| GH1 | loss | 40 | 0.217391304 | PAAD |
| GH2 | loss | 40 | 0.217391304 | PAAD |
| IFNA1 | loss | 101 | 0.548913043 | PAAD |
| IFNA10 | loss | 98 | 0.532608696 | PAAD |
| IFNA13 | loss | 100 | 0.543478261 | PAAD |
| IFNA14 | loss | 99 | 0.538043478 | PAAD |
| IFNA16 | loss | 98 | 0.532608696 | PAAD |
| IFNA17 | loss | 99 | 0.538043478 | PAAD |
| IFNA2 | loss | 100 | 0.543478261 | PAAD |
| IFNA21 | loss | 95 | 0.516304348 | PAAD |
| IFNA4 | loss | 96 | 0.52173913 | PAAD |
| IFNA5 | loss | 100 | 0.543478261 | PAAD |
| IFNA6 | loss | 100 | 0.543478261 | PAAD |
| IFNA7 | loss | 98 | 0.532608696 | PAAD |
| IFNA8 | loss | 100 | 0.543478261 | PAAD |
| IFNAR1 | loss | 57 | 0.309782609 | PAAD |
| IFNAR2 | loss | 57 | 0.309782609 | PAAD |
| IFNB1 | loss | 94 | 0.510869565 | PAAD |
| IFNE | loss | 102 | 0.554347826 | PAAD |
| IFNGR1 | loss | 87 | 0.472826087 | PAAD |
| IFNGR2 | loss | 57 | 0.309782609 | PAAD |
| IFNK | loss | 84 | 0.456521739 | PAAD |
| IFNLR1 | loss | 59 | 0.320652174 | PAAD |
| IFNW1 | loss | 95 | 0.516304348 | PAAD |
| IL10RB | loss | 57 | 0.309782609 | PAAD |
| IL11RA | loss | 59 | 0.320652174 | PAAD |
| IL12RB2 | loss | 46 | 0.25 | PAAD |
| IL15RA | loss | 39 | 0.211956522 | PAAD |
| IL20RA | loss | 87 | 0.472826087 | PAAD |
| IL22RA1 | loss | 59 | 0.320652174 | PAAD |
| IL22RA2 | loss | 87 | 0.472826087 | PAAD |
| IL23R | loss | 46 | 0.25 | PAAD |
| IL2RA | loss | 39 | 0.211956522 | PAAD |
| IL2RB | loss | 46 | 0.25 | PAAD |
| IL5RA | loss | 43 | 0.233695652 | PAAD |
| JAK1 | loss | 46 | 0.25 | PAAD |
| JAK2 | loss | 88 | 0.47826087 | PAAD |
| LIF | loss | 50 | 0.27173913 | PAAD |
| MPL | loss | 43 | 0.233695652 | PAAD |
| OSM | loss | 50 | 0.27173913 | PAAD |
| PIAS2 | loss | 124 | 0.673913043 | PAAD |
| PIM1 | loss | 61 | 0.331521739 | PAAD |
| PRL | loss | 72 | 0.391304348 | PAAD |
| PTPN11 | loss | 41 | 0.222826087 | PAAD |
| PTPN2 | loss | 53 | 0.288043478 | PAAD |
| SOCS2 | loss | 43 | 0.233695652 | PAAD |
| SOCS3 | loss | 40 | 0.217391304 | PAAD |
| SOCS6 | loss | 122 | 0.663043478 | PAAD |
| AOX1 | loss | 76 | 0.295719844 | SARC |
| BCL2 | loss | 81 | 0.315175097 | SARC |
| CDKN1A | loss | 70 | 0.272373541 | SARC |
| CISH | loss | 65 | 0.252918288 | SARC |
| CNTF | loss | 63 | 0.245136187 | SARC |
| CNTFR | loss | 79 | 0.307392996 | SARC |
| CSF2RB | loss | 68 | 0.26459144 | SARC |
| CSF3 | loss | 67 | 0.260700389 | SARC |
| CTF1 | loss | 59 | 0.229571984 | SARC |
| FHL1 | loss | 129 | 0.501945525 | SARC |
| GFAP | loss | 55 | 0.214007782 | SARC |
| IFNA1 | loss | 103 | 0.40077821 | SARC |
| IFNA10 | loss | 101 | 0.392996109 | SARC |
| IFNA13 | loss | 101 | 0.392996109 | SARC |
| IFNA14 | loss | 101 | 0.392996109 | SARC |
| IFNA16 | loss | 101 | 0.392996109 | SARC |
| IFNA17 | loss | 101 | 0.392996109 | SARC |
| IFNA2 | loss | 101 | 0.392996109 | SARC |
| IFNA21 | loss | 100 | 0.389105058 | SARC |
| IFNA4 | loss | 101 | 0.392996109 | SARC |
| IFNA5 | loss | 101 | 0.392996109 | SARC |
| IFNA6 | loss | 101 | 0.392996109 | SARC |
| IFNA7 | loss | 101 | 0.392996109 | SARC |
| IFNA8 | loss | 101 | 0.392996109 | SARC |
| IFNAR1 | loss | 55 | 0.214007782 | SARC |
| IFNAR2 | loss | 56 | 0.217898833 | SARC |
| IFNB1 | loss | 95 | 0.369649805 | SARC |
| IFNE | loss | 103 | 0.40077821 | SARC |
| IFNG | loss | 54 | 0.210116732 | SARC |
| IFNGR2 | loss | 55 | 0.214007782 | SARC |
| IFNK | loss | 97 | 0.377431907 | SARC |
| IFNL1 | loss | 61 | 0.237354086 | SARC |
| IFNL2 | loss | 60 | 0.233463035 | SARC |
| IFNL3 | loss | 60 | 0.233463035 | SARC |
| IFNW1 | loss | 97 | 0.377431907 | SARC |
| IL10 | loss | 75 | 0.291828794 | SARC |
| IL10RA | loss | 119 | 0.463035019 | SARC |
| IL10RB | loss | 56 | 0.217898833 | SARC |
| IL11 | loss | 88 | 0.342412451 | SARC |
| IL11RA | loss | 79 | 0.307392996 | SARC |
| IL12A | loss | 52 | 0.20233463 | SARC |
| IL13RA1 | loss | 129 | 0.501945525 | SARC |
| IL13RA2 | loss | 124 | 0.482490272 | SARC |
| IL15 | loss | 60 | 0.233463035 | SARC |
| IL15RA | loss | 117 | 0.455252918 | SARC |
| IL17D | loss | 122 | 0.474708171 | SARC |
| IL19 | loss | 75 | 0.291828794 | SARC |
| IL2 | loss | 62 | 0.241245136 | SARC |
| IL20 | loss | 75 | 0.291828794 | SARC |
| IL21 | loss | 63 | 0.245136187 | SARC |
| IL21R | loss | 59 | 0.229571984 | SARC |
| IL22 | loss | 57 | 0.221789883 | SARC |
| IL23A | loss | 69 | 0.26848249 | SARC |
| IL24 | loss | 75 | 0.291828794 | SARC |
| IL2RA | loss | 118 | 0.459143969 | SARC |
| IL2RB | loss | 69 | 0.26848249 | SARC |
| IL2RG | loss | 90 | 0.350194553 | SARC |
| IL4R | loss | 60 | 0.233463035 | SARC |
| JAK2 | loss | 94 | 0.365758755 | SARC |
| LIF | loss | 70 | 0.272373541 | SARC |
| OSM | loss | 70 | 0.272373541 | SARC |
| PIAS2 | loss | 81 | 0.315175097 | SARC |
| PIM1 | loss | 67 | 0.260700389 | SARC |
| PRL | loss | 70 | 0.272373541 | SARC |
| PTPN11 | loss | 60 | 0.233463035 | SARC |
| PTPN2 | loss | 56 | 0.217898833 | SARC |
| PTPN6 | loss | 80 | 0.311284047 | SARC |
| SOCS1 | loss | 53 | 0.206225681 | SARC |
| SOCS2 | loss | 65 | 0.252918288 | SARC |
| SOCS4 | loss | 70 | 0.272373541 | SARC |
| SOCS5 | loss | 86 | 0.33463035 | SARC |
| SOCS6 | loss | 92 | 0.357976654 | SARC |
| SOCS7 | loss | 66 | 0.256809339 | SARC |
| STAM | loss | 116 | 0.451361868 | SARC |
| STAM2 | loss | 69 | 0.26848249 | SARC |
| STAT1 | loss | 73 | 0.284046693 | SARC |
| STAT2 | loss | 69 | 0.26848249 | SARC |
| STAT3 | loss | 62 | 0.241245136 | SARC |
| STAT4 | loss | 73 | 0.284046693 | SARC |
| STAT5A | loss | 62 | 0.241245136 | SARC |
| STAT5B | loss | 63 | 0.245136187 | SARC |
| STAT6 | loss | 66 | 0.256809339 | SARC |
| BCL2 | loss | 201 | 0.455782313 | STAD |
| CISH | loss | 149 | 0.337868481 | STAD |
| CNTFR | loss | 114 | 0.258503401 | STAD |
| CSF2 | loss | 150 | 0.340136054 | STAD |
| CSF2RB | loss | 147 | 0.333333333 | STAD |
| EPOR | loss | 134 | 0.303854875 | STAD |
| IFNA1 | loss | 184 | 0.41723356 | STAD |
| IFNA10 | loss | 182 | 0.412698413 | STAD |
| IFNA13 | loss | 184 | 0.41723356 | STAD |
| IFNA14 | loss | 184 | 0.41723356 | STAD |
| IFNA16 | loss | 182 | 0.412698413 | STAD |
| IFNA17 | loss | 184 | 0.41723356 | STAD |
| IFNA2 | loss | 184 | 0.41723356 | STAD |
| IFNA21 | loss | 183 | 0.414965986 | STAD |
| IFNA4 | loss | 183 | 0.414965986 | STAD |
| IFNA5 | loss | 185 | 0.419501134 | STAD |
| IFNA6 | loss | 184 | 0.41723356 | STAD |
| IFNA7 | loss | 182 | 0.412698413 | STAD |
| IFNA8 | loss | 184 | 0.41723356 | STAD |
| IFNAR1 | loss | 162 | 0.367346939 | STAD |
| IFNAR2 | loss | 164 | 0.371882086 | STAD |
| IFNB1 | loss | 181 | 0.410430839 | STAD |
| IFNE | loss | 184 | 0.41723356 | STAD |
| IFNGR2 | loss | 162 | 0.367346939 | STAD |
| IFNK | loss | 165 | 0.37414966 | STAD |
| IFNLR1 | loss | 114 | 0.258503401 | STAD |
| IFNW1 | loss | 183 | 0.414965986 | STAD |
| IL10RB | loss | 163 | 0.369614512 | STAD |
| IL11RA | loss | 113 | 0.256235828 | STAD |
| IL12B | loss | 128 | 0.290249433 | STAD |
| IL12RB1 | loss | 122 | 0.276643991 | STAD |
| IL13 | loss | 148 | 0.335600907 | STAD |
| IL15 | loss | 166 | 0.376417234 | STAD |
| IL2 | loss | 169 | 0.383219955 | STAD |
| IL21 | loss | 169 | 0.383219955 | STAD |
| IL22RA1 | loss | 112 | 0.253968254 | STAD |
| IL27RA | loss | 127 | 0.287981859 | STAD |
| IL2RB | loss | 147 | 0.333333333 | STAD |
| IL3 | loss | 151 | 0.342403628 | STAD |
| IL4 | loss | 148 | 0.335600907 | STAD |
| IL5 | loss | 151 | 0.342403628 | STAD |
| IL5RA | loss | 139 | 0.315192744 | STAD |
| IL6ST | loss | 149 | 0.337868481 | STAD |
| IL9 | loss | 144 | 0.326530612 | STAD |
| IRF9 | loss | 112 | 0.253968254 | STAD |
| JAK2 | loss | 174 | 0.394557823 | STAD |
| JAK3 | loss | 124 | 0.281179138 | STAD |
| LIF | loss | 143 | 0.324263039 | STAD |
| OSM | loss | 143 | 0.324263039 | STAD |
| PIAS1 | loss | 94 | 0.213151927 | STAD |
| PIAS2 | loss | 191 | 0.433106576 | STAD |
| PIAS4 | loss | 164 | 0.371882086 | STAD |
| PTPN2 | loss | 101 | 0.229024943 | STAD |
| SOCS4 | loss | 117 | 0.265306122 | STAD |
| SOCS6 | loss | 195 | 0.442176871 | STAD |
| TSLP | loss | 162 | 0.367346939 | STAD |
| TYK2 | loss | 134 | 0.303854875 | STAD |
| BCL2 | loss | 313 | 0.5008 | STES |
| CISH | loss | 272 | 0.4352 | STES |
| CNTFR | loss | 203 | 0.3248 | STES |
| CSF2 | loss | 245 | 0.392 | STES |
| CSF2RB | loss | 221 | 0.3536 | STES |
| CSF3R | loss | 138 | 0.2208 | STES |
| EPOR | loss | 207 | 0.3312 | STES |
| IFNA1 | loss | 299 | 0.4784 | STES |
| IFNA10 | loss | 296 | 0.4736 | STES |
| IFNA13 | loss | 299 | 0.4784 | STES |
| IFNA14 | loss | 298 | 0.4768 | STES |
| IFNA16 | loss | 296 | 0.4736 | STES |
| IFNA17 | loss | 298 | 0.4768 | STES |
| IFNA2 | loss | 299 | 0.4784 | STES |
| IFNA21 | loss | 297 | 0.4752 | STES |
| IFNA4 | loss | 297 | 0.4752 | STES |
| IFNA5 | loss | 300 | 0.48 | STES |
| IFNA6 | loss | 299 | 0.4784 | STES |
| IFNA7 | loss | 296 | 0.4736 | STES |
| IFNA8 | loss | 299 | 0.4784 | STES |
| IFNAR1 | loss | 263 | 0.4208 | STES |
| IFNAR2 | loss | 265 | 0.424 | STES |
| IFNB1 | loss | 294 | 0.4704 | STES |
| IFNE | loss | 299 | 0.4784 | STES |
| IFNGR2 | loss | 262 | 0.4192 | STES |
| IFNK | loss | 267 | 0.4272 | STES |
| IFNLR1 | loss | 181 | 0.2896 | STES |
| IFNW1 | loss | 297 | 0.4752 | STES |
| IL10RA | loss | 163 | 0.2608 | STES |
| IL10RB | loss | 264 | 0.4224 | STES |
| IL11RA | loss | 202 | 0.3232 | STES |
| IL12B | loss | 214 | 0.3424 | STES |
| IL12RB1 | loss | 184 | 0.2944 | STES |
| IL12RB2 | loss | 136 | 0.2176 | STES |
| IL13 | loss | 243 | 0.3888 | STES |
| IL15 | loss | 249 | 0.3984 | STES |
| IL17D | loss | 132 | 0.2112 | STES |
| IL2 | loss | 261 | 0.4176 | STES |
| IL21 | loss | 261 | 0.4176 | STES |
| IL21R | loss | 137 | 0.2192 | STES |
| IL22RA1 | loss | 179 | 0.2864 | STES |
| IL23R | loss | 136 | 0.2176 | STES |
| IL27RA | loss | 193 | 0.3088 | STES |
| IL2RB | loss | 220 | 0.352 | STES |
| IL3 | loss | 246 | 0.3936 | STES |
| IL4 | loss | 244 | 0.3904 | STES |
| IL4R | loss | 137 | 0.2192 | STES |
| IL5 | loss | 246 | 0.3936 | STES |
| IL5RA | loss | 250 | 0.4 | STES |
| IL6ST | loss | 256 | 0.4096 | STES |
| IL9 | loss | 235 | 0.376 | STES |
| IRF9 | loss | 159 | 0.2544 | STES |
| JAK1 | loss | 134 | 0.2144 | STES |
| JAK2 | loss | 281 | 0.4496 | STES |
| JAK3 | loss | 187 | 0.2992 | STES |
| LIF | loss | 216 | 0.3456 | STES |
| OSM | loss | 216 | 0.3456 | STES |
| PIAS1 | loss | 150 | 0.24 | STES |
| PIAS2 | loss | 300 | 0.48 | STES |
| PIAS4 | loss | 252 | 0.4032 | STES |
| PTPN2 | loss | 165 | 0.264 | STES |
| SOCS1 | loss | 136 | 0.2176 | STES |
| SOCS4 | loss | 162 | 0.2592 | STES |
| SOCS6 | loss | 307 | 0.4912 | STES |
| TSLP | loss | 262 | 0.4192 | STES |
| TYK2 | loss | 212 | 0.3392 | STES |
| CSF2RB | loss | 108 | 0.200371058 | UCEC |
| IL2 | loss | 108 | 0.200371058 | UCEC |
| IL21 | loss | 108 | 0.200371058 | UCEC |
| PIAS1 | loss | 121 | 0.224489796 | UCEC |
| PIAS4 | loss | 157 | 0.291280148 | UCEC |
| STAT3 | loss | 114 | 0.211502783 | UCEC |
| STAT5A | loss | 113 | 0.209647495 | UCEC |
| STAT5B | loss | 113 | 0.209647495 | UCEC |
