# Supplementary material for: An immunoevasive strategy through clinically-relevant pan-cancer genomic and transcriptomic alterations of JAK-STAT signaling components
Source: Mol Med. 2019 Nov 4;25:46. doi: 10.1186/s10020-019-0114-1 (PMC6829980; doi:10.1186/s10020-019-0114-1)
Supplement: Supplementary file 3 — Additional file 3. Univariate and multivariate Cox proportional hazards regression analyses to determine the independence of 28-gene signature from TNM staging and age. [file 10020_2019_114_MOESM3_ESM.docx]

**Additional file 3. Univariate and multivariate Cox proportional hazards regression analyses to determine the independence of 28-gene signature from TNM staging and age.**

|  |  |  |
| --- | --- | --- |
|  |  | |
|  | **Hazard Ratio (95% CI)** | ***P*-value** |
| **All gliomas** | **Univariate** | |
| Q4 vs. Q1 | 6.832 (4.638 - 10.070) | **< 0.0001** |
| Age | 1.067 (1.053 - 1.081) | **< 0.0001** |
| Gender (male vs. female) | 1.321 (0.945 - 1.848) | 0.104 |
|  |  |  |
|  | **Multivariate** | |
| Q4 vs. Q1 | 4.316 (2.837 - 6.565) | **< 0.0001** |
| Age | 1.046 (1.032 - 1.060) | **< 0.0001** |
|  |  |  |
| **Astrocytoma** | **Univariate** | |
| Q4 vs. Q1 | 2.377 (1.156 - 4.886) | **0.018** |
|  |  |  |
| **Oligoastrocytoma** | **Univariate** | |
| Q4 vs. Q1 | 2.730 (1.109 - 8.031) | **0.038** |
|  |  |  |
| **Pan-kidney** | **Univariate** | |
| Q4 vs. Q1 | 3.335 (2.253 - 4.937) | **< 0.0001** |
| Age | 1.034 (1.019 - 1.048) | **< 0.0001** |
| Gender (male vs. female) | 0.875 (0.612 - 1.247) | 0.456 |
|  |  |  |
|  | **Multivariate** | |
| Q4 vs. Q1 | 2.379 (1.564 - 3.620) | **< 0.0001** |
| Age | 1.034 (1.018 - 1.050) | **< 0.0001** |
| TNM staging | 1.837 (1.550 - 2.178) | **< 0.0001** |
|  |  |  |
| **Clear cell renal cell** | **Univariate** | |
| Q4 vs. Q1 | 4.292 (2.611 - 7.057) | **< 0.0001** |
| Age | 1.017 (0.998 - 1.035) | 0.071 |
| Gender (male vs. female) | 0.879 (0.568 - 1.362) | 0.564 |
|  |  |  |
|  | **Multivariate** | |
| Q4 vs. Q1 | 2.552 (1.510 - 4.314) | **0.00047** |
| TNM staging | 1.839 (1.490 - 2.271) | **< 0.0001** |
|  |  |  |
| **Lung** | **Univariate** | |
| Q4 vs. Q1 | 0.624 (0.391 - 0.788) | **0.028** |
| Age | 1.012 (0.987 - 1.039) | 0.341 |
| Gender (male vs. female) | 0.859 (0.541 - 1.365) | 0.521 |
|  |  |  |
|  | **Multivariate** | |
| Q4 vs. Q1 | 0.636 (0.457 - 0.889) | **0.031** |
| TNM staging | 1.682 (1.352 - 2.094) | **< 0.0001** |
|  |  |  |
| **Endometrium** | **Univariate** | |
| Q4 vs. Q1 | 0.504 (0.233 - 0.087) | **0.027** |
|  |  |  |
|  | **Multivariate** | |
| Q4 vs. Q1 | 0.434 (0.201 - 0.936) | **0.033** |
| TNM staging | 2.306 (1.685 - 3.157) | **< 0.0001** |
|  |  |  |

Significant P values are marked in bold.
