# Supplementary figures and images for: An immunoevasive strategy through clinically-relevant pan-cancer genomic and transcriptomic alterations of JAK-STAT signaling components
Source: Mol Med. 2019 Nov 4;25:46. doi: 10.1186/s10020-019-0114-1 (PMC6829980; doi:10.1186/s10020-019-0114-1)

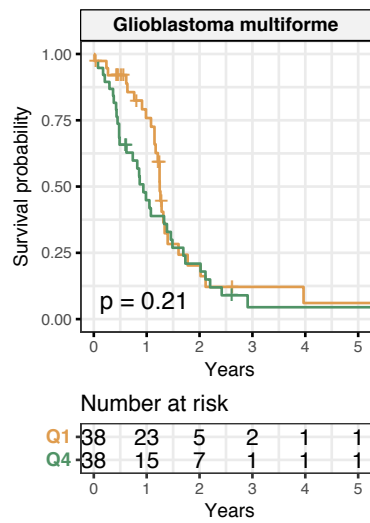

Supplement: Supplementary file 4 — Additional file 4. Kaplan-Meier analyses of patients with glioblastoma multiforme stratified by the 28-gene signature. The log-rank test is used to compare patients within the 1st and 4th survival quartiles. [file 10020_2019_114_MOESM4_ESM.pdf]
