# Supplementary material for: An immunoevasive strategy through clinically-relevant pan-cancer genomic and transcriptomic alterations of JAK-STAT signaling components
Source: Mol Med. 2019 Nov 4;25:46. doi: 10.1186/s10020-019-0114-1 (PMC6829980; doi:10.1186/s10020-019-0114-1)
Supplement: Supplementary file 5 — Additional file 5. Differentially expressed genes between patients separated by the 28-gene signature into 4th and 1st quartiles. [file 10020_2019_114_MOESM5_ESM.docx]

**Additional file 5. Differentially expressed genes between patients separated by the 28-gene signature into 4th and 1st quartiles.**

|  |  |  |  |  |
| --- | --- | --- | --- | --- |
| **Entrez ID** | **Gene Symbol** | **Log2 fold change** | **Adjusted P-value** | **Cancer** |
| 9356 | SLC22A6 | -3.389590419 | 5.38E-12 | Clear cell renal cell |
| 340024 | SLC6A19 | -3.345613605 | 1.86E-11 | Clear cell renal cell |
| 160728 | SLC5A8 | -3.32918932 | 3.38E-12 | Clear cell renal cell |
| 116085 | SLC22A12 | -3.236971899 | 1.09E-12 | Clear cell renal cell |
| 134288 | TMEM174 | -3.230729909 | 1.49E-15 | Clear cell renal cell |
| 4634 | MYL3 | -2.837085479 | 3.55E-20 | Clear cell renal cell |
| 3158 | HMGCS2 | -2.820488994 | 6.93E-11 | Clear cell renal cell |
| 5105 | PCK1 | -2.795882871 | 1.35E-14 | Clear cell renal cell |
| 2538 | G6PC | -2.770045969 | 2.04E-12 | Clear cell renal cell |
| 762 | CA4 | -2.7484082 | 3.22E-19 | Clear cell renal cell |
| 5972 | REN | -2.683863151 | 1.03E-09 | Clear cell renal cell |
| 10590 | SCGN | -2.681782527 | 6.34E-11 | Clear cell renal cell |
| 5053 | PAH | -2.640571362 | 1.16E-09 | Clear cell renal cell |
| 5340 | PLG | -2.631211672 | 1.21E-07 | Clear cell renal cell |
| 158326 | FREM1 | -2.629472257 | 1.50E-15 | Clear cell renal cell |
| 27004 | None | -2.614858666 | 1.58E-15 | Clear cell renal cell |
| 643236 | TMEM72 | -2.538333317 | 2.75E-17 | Clear cell renal cell |
| 6561 | SLC13A1 | -2.491005753 | 7.82E-08 | Clear cell renal cell |
| 1579 | CYP4A11 | -2.48949607 | 7.73E-07 | Clear cell renal cell |
| 10117 | ENAM | -2.453332139 | 2.80E-18 | Clear cell renal cell |
| 375057 | STUM | -2.441510738 | 1.21E-17 | Clear cell renal cell |
| 256764 | WDR72 | -2.41514495 | 6.69E-24 | Clear cell renal cell |
| 10647 | SCGB1D2 | -2.387393128 | 5.80E-21 | Clear cell renal cell |
| 149466 | C1orf210 | -2.355619787 | 2.89E-16 | Clear cell renal cell |
| 29953 | TRHDE | -2.345512081 | 4.48E-12 | Clear cell renal cell |
| 9376 | SLC22A8 | -2.340739532 | 2.15E-11 | Clear cell renal cell |
| 1586 | CYP17A1 | -2.295981685 | 3.62E-11 | Clear cell renal cell |
| 9390 | SLC22A13 | -2.289144113 | 1.02E-13 | Clear cell renal cell |
| 10840 | ALDH1L1 | -2.266583212 | 4.13E-16 | Clear cell renal cell |
| 2167 | FABP4 | -2.200049088 | 1.12E-11 | Clear cell renal cell |
| 4883 | NPR3 | -2.195467363 | 8.10E-16 | Clear cell renal cell |
| 80157 | CWH43 | -2.192706142 | 9.95E-11 | Clear cell renal cell |
| 283238 | SLC22A24 | -2.1655784 | 9.20E-18 | Clear cell renal cell |
| 134285 | TMEM171 | -2.160739616 | 1.95E-24 | Clear cell renal cell |
| 153328 | SLC25A48 | -2.149266172 | 5.88E-15 | Clear cell renal cell |
| 10891 | PPARGC1A | -2.130118599 | 6.18E-20 | Clear cell renal cell |
| 80763 | SPX | -2.129411604 | 4.18E-12 | Clear cell renal cell |
| 387700 | SLC16A12 | -2.102008798 | 3.50E-13 | Clear cell renal cell |
| 348932 | SLC6A18 | -2.093713859 | 4.38E-07 | Clear cell renal cell |
| 388387 | None | -2.076884133 | 1.04E-07 | Clear cell renal cell |
| 283392 | None | -2.069873176 | 5.13E-11 | Clear cell renal cell |
| 197257 | LDHD | -2.055597007 | 4.31E-23 | Clear cell renal cell |
| 9058 | SLC13A2 | -2.028434983 | 8.92E-11 | Clear cell renal cell |
| 8825 | LIN7A | -2.000127824 | 3.89E-18 | Clear cell renal cell |
| 51179 | HAO2 | -1.994627809 | 2.81E-07 | Clear cell renal cell |
| 80036 | TRPM3 | -1.98916676 | 3.12E-10 | Clear cell renal cell |
| 146894 | CD300LG | -1.986994479 | 2.04E-13 | Clear cell renal cell |
| 6000 | RGS7 | -1.968743149 | 1.46E-09 | Clear cell renal cell |
| 79614 | None | -1.964189026 | 8.35E-15 | Clear cell renal cell |
| 55753 | OGDHL | -1.959654732 | 4.17E-13 | Clear cell renal cell |
| 6523 | SLC5A1 | -1.929938279 | 2.56E-06 | Clear cell renal cell |
| 266722 | HS6ST3 | -1.908777295 | 5.23E-10 | Clear cell renal cell |
| 84803 | GPAT3 | -1.899759382 | 4.95E-22 | Clear cell renal cell |
| 229 | ALDOB | -1.888909994 | 1.53E-05 | Clear cell renal cell |
| 185 | AGTR1 | -1.880755544 | 4.06E-13 | Clear cell renal cell |
| 6555 | SLC10A2 | -1.877868231 | 6.85E-06 | Clear cell renal cell |
| 196047 | None | -1.873806627 | 4.39E-16 | Clear cell renal cell |
| 11001 | SLC27A2 | -1.86862134 | 2.93E-13 | Clear cell renal cell |
| 120071 | LARGE2 | -1.860722594 | 3.48E-13 | Clear cell renal cell |
| 345079 | SOWAHB | -1.855131343 | 7.83E-18 | Clear cell renal cell |
| 364 | AQP7 | -1.836645309 | 3.98E-14 | Clear cell renal cell |
| 59272 | ACE2 | -1.821889461 | 1.66E-05 | Clear cell renal cell |
| 5314 | PKHD1 | -1.811929944 | 3.13E-09 | Clear cell renal cell |
| 4329 | ALDH6A1 | -1.807544504 | 2.56E-29 | Clear cell renal cell |
| 723809 | None | -1.805963873 | 1.42E-09 | Clear cell renal cell |
| 220963 | SLC16A9 | -1.761433114 | 9.04E-09 | Clear cell renal cell |
| 2104 | ESRRG | -1.756572229 | 1.56E-10 | Clear cell renal cell |
| 53354 | PANK1 | -1.75232683 | 1.22E-32 | Clear cell renal cell |
| 155006 | TMEM213 | -1.73000394 | 0.000124076 | Clear cell renal cell |
| 55586 | MIOX | -1.728877355 | 5.64E-06 | Clear cell renal cell |
| 284422 | SMIM24 | -1.719295693 | 1.07E-11 | Clear cell renal cell |
| 9053 | MAP7 | -1.702997582 | 3.56E-26 | Clear cell renal cell |
| 10913 | EDAR | -1.701155139 | 6.66E-10 | Clear cell renal cell |
| 27233 | SULT1C4 | -1.698059468 | 1.40E-10 | Clear cell renal cell |
| 151126 | ZNF385B | -1.694824189 | 4.99E-08 | Clear cell renal cell |
| 57733 | GBA3 | -1.666888998 | 5.37E-06 | Clear cell renal cell |
| 399823 | FOXI2 | -1.661886512 | 3.38E-07 | Clear cell renal cell |
| 374383 | NCR3LG1 | -1.66047995 | 2.10E-21 | Clear cell renal cell |
| 6329 | SCN4A | -1.6601547 | 2.13E-13 | Clear cell renal cell |
| 79041 | TMEM38A | -1.655324873 | 6.44E-16 | Clear cell renal cell |
| 123264 | SLC51B | -1.652601281 | 1.18E-11 | Clear cell renal cell |
| 84570 | COL25A1 | -1.648513242 | 7.87E-11 | Clear cell renal cell |
| 8972 | MGAM | -1.646372499 | 9.31E-07 | Clear cell renal cell |
| 27324 | TOX3 | -1.632303266 | 2.25E-06 | Clear cell renal cell |
| 54511 | HMGCLL1 | -1.631804792 | 1.39E-12 | Clear cell renal cell |
| 400961 | PAIP2B | -1.627260954 | 1.01E-23 | Clear cell renal cell |
| 5745 | PTH1R | -1.62200768 | 2.15E-12 | Clear cell renal cell |
| 4137 | MAPT | -1.611101584 | 2.78E-12 | Clear cell renal cell |
| 129049 | SGSM1 | -1.606209509 | 4.35E-19 | Clear cell renal cell |
| 341640 | FREM2 | -1.60573881 | 5.25E-07 | Clear cell renal cell |
| 51471 | NAT8B | -1.598919438 | 2.70E-08 | Clear cell renal cell |
| 9099 | USP2 | -1.593235985 | 1.10E-14 | Clear cell renal cell |
| 25928 | SOSTDC1 | -1.592569596 | 7.70E-07 | Clear cell renal cell |
| 388595 | TMEM82 | -1.58313274 | 9.56E-06 | Clear cell renal cell |
| 50617 | ATP6V0A4 | -1.57491669 | 0.000793566 | Clear cell renal cell |
| 2299 | FOXI1 | -1.570250274 | 0.000108255 | Clear cell renal cell |
| 54886 | PLPPR1 | -1.567179275 | 7.66E-07 | Clear cell renal cell |
| 5017 | OVOL1 | -1.561309559 | 3.65E-09 | Clear cell renal cell |
| 1551 | CYP3A7 | -1.560672365 | 5.16E-09 | Clear cell renal cell |
| 137970 | UNC5D | -1.550719488 | 1.84E-05 | Clear cell renal cell |
| 2018 | EMX2 | -1.549613177 | 2.45E-11 | Clear cell renal cell |
| 170690 | ADAMTS16 | -1.548470852 | 2.31E-09 | Clear cell renal cell |
| 146456 | TMED6 | -1.534775378 | 7.03E-08 | Clear cell renal cell |
| 54756 | IL17RD | -1.532893375 | 4.95E-22 | Clear cell renal cell |
| 8715 | NOL4 | -1.511749919 | 2.59E-07 | Clear cell renal cell |
| 54836 | BSPRY | -1.511383808 | 3.75E-09 | Clear cell renal cell |
| 223117 | SEMA3D | -1.509524027 | 3.46E-07 | Clear cell renal cell |
| 9398 | CD101 | 1.500076944 | 1.70E-28 | Clear cell renal cell |
| 389643 | NUGGC | 1.501505961 | 1.78E-09 | Clear cell renal cell |
| 4261 | CIITA | 1.503114803 | 2.77E-28 | Clear cell renal cell |
| 51513 | ETV7 | 1.503347154 | 8.53E-16 | Clear cell renal cell |
| 51338 | MS4A4A | 1.506207915 | 4.42E-24 | Clear cell renal cell |
| 284233 | None | 1.506254047 | 2.12E-11 | Clear cell renal cell |
| 1893 | ECM1 | 1.507547464 | 8.98E-22 | Clear cell renal cell |
| 9928 | KIF14 | 1.507547637 | 3.16E-20 | Clear cell renal cell |
| 1462 | VCAN | 1.507592347 | 8.08E-12 | Clear cell renal cell |
| 64231 | MS4A6A | 1.508207541 | 9.14E-30 | Clear cell renal cell |
| 8842 | PROM1 | 1.509084951 | 0.00082986 | Clear cell renal cell |
| 729786 | None | 1.510535734 | 5.08E-14 | Clear cell renal cell |
| 93589 | CACNA2D4 | 1.511325341 | 1.45E-21 | Clear cell renal cell |
| 56992 | KIF15 | 1.511446065 | 2.08E-22 | Clear cell renal cell |
| 91584 | PLXNA4 | 1.511570839 | 8.50E-11 | Clear cell renal cell |
| 65989 | DLK2 | 1.511675525 | 8.70E-09 | Clear cell renal cell |
| 55165 | CEP55 | 1.512132889 | 2.31E-25 | Clear cell renal cell |
| 2921 | CXCL3 | 1.512159157 | 1.42E-10 | Clear cell renal cell |
| 140686 | WFDC3 | 1.512889676 | 3.22E-11 | Clear cell renal cell |
| 2357 | FPR1 | 1.515808452 | 6.25E-18 | Clear cell renal cell |
| 100188947 | None | 1.516086283 | 3.49E-10 | Clear cell renal cell |
| 26548 | ITGB1BP2 | 1.516528722 | 9.32E-19 | Clear cell renal cell |
| 432 | ASGR1 | 1.517515621 | 7.58E-21 | Clear cell renal cell |
| 115572 | TENT5B | 1.51987219 | 1.95E-13 | Clear cell renal cell |
| 10859 | LILRB1 | 1.520474188 | 3.23E-32 | Clear cell renal cell |
| 80380 | PDCD1LG2 | 1.521347414 | 7.63E-26 | Clear cell renal cell |
| 57105 | CYSLTR2 | 1.523216512 | 4.65E-08 | Clear cell renal cell |
| 6271 | S100A1 | 1.52355113 | 4.86E-07 | Clear cell renal cell |
| 285154 | None | 1.524537493 | 2.51E-15 | Clear cell renal cell |
| 922 | CD5L | 1.525321109 | 1.31E-05 | Clear cell renal cell |
| 6404 | SELPLG | 1.525438615 | 4.17E-37 | Clear cell renal cell |
| 128414 | NKAIN4 | 1.526333528 | 3.91E-05 | Clear cell renal cell |
| 2633 | GBP1 | 1.527060887 | 8.35E-23 | Clear cell renal cell |
| 3918 | LAMC2 | 1.528065126 | 4.43E-08 | Clear cell renal cell |
| 2274 | FHL2 | 1.528658016 | 5.70E-17 | Clear cell renal cell |
| 5328 | PLAU | 1.528828065 | 2.47E-26 | Clear cell renal cell |
| 402665 | IGLON5 | 1.530364191 | 0.000143962 | Clear cell renal cell |
| 6367 | CCL22 | 1.530404288 | 1.98E-14 | Clear cell renal cell |
| 2208 | FCER2 | 1.530600286 | 1.73E-12 | Clear cell renal cell |
| 5348 | FXYD1 | 1.530759211 | 6.54E-07 | Clear cell renal cell |
| 2920 | CXCL2 | 1.532667959 | 1.30E-09 | Clear cell renal cell |
| 10288 | LILRB2 | 1.532766114 | 1.82E-33 | Clear cell renal cell |
| 2175 | FANCA | 1.533062713 | 1.04E-28 | Clear cell renal cell |
| 2568 | GABRP | 1.53320177 | 1.74E-07 | Clear cell renal cell |
| 51303 | FKBP11 | 1.533215121 | 4.71E-33 | Clear cell renal cell |
| 6507 | SLC1A3 | 1.533842122 | 1.29E-18 | Clear cell renal cell |
| 149297 | FAM78B | 1.534782128 | 1.60E-26 | Clear cell renal cell |
| 199 | AIF1 | 1.535071804 | 3.97E-36 | Clear cell renal cell |
| 66002 | CYP4F12 | 1.535119028 | 1.97E-09 | Clear cell renal cell |
| 10077 | TSPAN32 | 1.535542385 | 7.52E-20 | Clear cell renal cell |
| 55384 | None | 1.535965062 | 1.06E-08 | Clear cell renal cell |
| 113177 | IZUMO4 | 1.536221788 | 2.10E-13 | Clear cell renal cell |
| 283551 | None | 1.536873849 | 3.13E-23 | Clear cell renal cell |
| 126353 | MISP | 1.53816159 | 6.52E-06 | Clear cell renal cell |
| 23025 | UNC13A | 1.538316026 | 5.01E-13 | Clear cell renal cell |
| 54551 | MAGEL2 | 1.538870889 | 1.29E-10 | Clear cell renal cell |
| 57482 | KIAA1211 | 1.539237732 | 1.78E-14 | Clear cell renal cell |
| 126549 | ANKLE1 | 1.540523393 | 4.40E-18 | Clear cell renal cell |
| 9046 | DOK2 | 1.54151526 | 4.18E-28 | Clear cell renal cell |
| 144455 | E2F7 | 1.543461894 | 6.79E-22 | Clear cell renal cell |
| 5122 | PCSK1 | 1.543857335 | 1.84E-10 | Clear cell renal cell |
| 54209 | TREM2 | 1.543903833 | 9.15E-21 | Clear cell renal cell |
| 155185 | AMZ1 | 1.544206051 | 1.17E-09 | Clear cell renal cell |
| 1058 | CENPA | 1.54574843 | 1.44E-21 | Clear cell renal cell |
| 79865 | TREML2 | 1.545963204 | 2.34E-21 | Clear cell renal cell |
| 8529 | CYP4F2 | 1.546003212 | 8.11E-07 | Clear cell renal cell |
| 1591 | CYP24A1 | 1.547506326 | 0.000126305 | Clear cell renal cell |
| 1506 | CTRL | 1.548187312 | 2.97E-20 | Clear cell renal cell |
| 943 | TNFRSF8 | 1.548999353 | 8.26E-31 | Clear cell renal cell |
| 9253 | NUMBL | 1.549205781 | 1.16E-28 | Clear cell renal cell |
| 1536 | CYBB | 1.550806684 | 8.74E-19 | Clear cell renal cell |
| 22801 | ITGA11 | 1.550810257 | 4.58E-14 | Clear cell renal cell |
| 57464 | STRIP2 | 1.550966565 | 3.12E-09 | Clear cell renal cell |
| 6334 | SCN8A | 1.551056237 | 9.19E-13 | Clear cell renal cell |
| 9976 | CLEC2B | 1.551790113 | 8.08E-30 | Clear cell renal cell |
| 388325 | SCIMP | 1.552816675 | 9.60E-23 | Clear cell renal cell |
| 11010 | GLIPR1 | 1.553557066 | 4.66E-33 | Clear cell renal cell |
| 7477 | WNT7B | 1.55487023 | 1.50E-08 | Clear cell renal cell |
| 64581 | CLEC7A | 1.555333779 | 1.34E-24 | Clear cell renal cell |
| 7305 | TYROBP | 1.556035344 | 3.62E-31 | Clear cell renal cell |
| 84000 | TMPRSS13 | 1.556479886 | 2.65E-16 | Clear cell renal cell |
| 9048 | ARTN | 1.556630011 | 2.20E-13 | Clear cell renal cell |
| 3897 | L1CAM | 1.557746354 | 4.25E-06 | Clear cell renal cell |
| 219833 | C11orf45 | 1.557957698 | 4.13E-23 | Clear cell renal cell |
| 387097 | None | 1.558337404 | 5.17E-16 | Clear cell renal cell |
| 1606 | DGKA | 1.559344469 | 3.04E-34 | Clear cell renal cell |
| 283663 | None | 1.559523446 | 1.46E-19 | Clear cell renal cell |
| 162514 | TRPV3 | 1.561051485 | 1.02E-15 | Clear cell renal cell |
| 6261 | RYR1 | 1.561507971 | 6.27E-21 | Clear cell renal cell |
| 79626 | TNFAIP8L2 | 1.561644441 | 3.91E-32 | Clear cell renal cell |
| 51314 | NME8 | 1.562307846 | 4.87E-21 | Clear cell renal cell |
| 125488 | TTC39C | 1.562695281 | 6.09E-33 | Clear cell renal cell |
| 84689 | None | 1.563404876 | 1.68E-17 | Clear cell renal cell |
| 1475 | CSTA | 1.564573601 | 3.85E-26 | Clear cell renal cell |
| 27180 | SIGLEC9 | 1.565030434 | 2.93E-26 | Clear cell renal cell |
| 64073 | C19orf33 | 1.565208803 | 1.01E-07 | Clear cell renal cell |
| 2335 | FN1 | 1.565484713 | 3.92E-22 | Clear cell renal cell |
| 2634 | GBP2 | 1.566341762 | 2.37E-42 | Clear cell renal cell |
| 81794 | ADAMTS10 | 1.566417165 | 1.03E-16 | Clear cell renal cell |
| 401563 | C9orf139 | 1.566945074 | 3.27E-16 | Clear cell renal cell |
| 54829 | ASPN | 1.567784989 | 3.67E-16 | Clear cell renal cell |
| 8635 | RNASET2 | 1.568317163 | 4.82E-16 | Clear cell renal cell |
| 80714 | PBX4 | 1.568813994 | 8.14E-15 | Clear cell renal cell |
| 125 | ADH1B | 1.569782389 | 1.03E-06 | Clear cell renal cell |
| 10112 | KIF20A | 1.571285356 | 1.07E-19 | Clear cell renal cell |
| 2123 | EVI2A | 1.572577724 | 1.51E-28 | Clear cell renal cell |
| 7278 | TUBA3C | 1.57283874 | 2.32E-08 | Clear cell renal cell |
| 6355 | CCL8 | 1.572912165 | 3.01E-17 | Clear cell renal cell |
| 4494 | MT1F | 1.573186165 | 5.04E-12 | Clear cell renal cell |
| 126014 | OSCAR | 1.573436276 | 9.43E-31 | Clear cell renal cell |
| 3237 | HOXD11 | 1.573897714 | 6.70E-15 | Clear cell renal cell |
| 27087 | B3GAT1 | 1.5764143 | 4.22E-13 | Clear cell renal cell |
| 4501 | MT1X | 1.576610457 | 2.73E-11 | Clear cell renal cell |
| 6775 | STAT4 | 1.578403178 | 1.34E-25 | Clear cell renal cell |
| 158314 | None | 1.578629795 | 8.41E-10 | Clear cell renal cell |
| 57348 | TTYH1 | 1.57919955 | 3.74E-12 | Clear cell renal cell |
| 26011 | TENM4 | 1.580130539 | 1.25E-13 | Clear cell renal cell |
| 259197 | NCR3 | 1.580677662 | 1.50E-20 | Clear cell renal cell |
| 10350 | ABCA9 | 1.58090297 | 3.48E-14 | Clear cell renal cell |
| 1890 | TYMP | 1.581471729 | 7.28E-22 | Clear cell renal cell |
| 225 | ABCD2 | 1.582148397 | 5.48E-21 | Clear cell renal cell |
| 343413 | FCRL6 | 1.583955943 | 3.37E-20 | Clear cell renal cell |
| 260436 | FDCSP | 1.583958088 | 4.87E-07 | Clear cell renal cell |
| 64926 | RASAL3 | 1.584188756 | 2.40E-32 | Clear cell renal cell |
| 7020 | TFAP2A | 1.584763423 | 5.13E-08 | Clear cell renal cell |
| 165904 | XIRP1 | 1.584771287 | 3.85E-13 | Clear cell renal cell |
| 10148 | EBI3 | 1.585046248 | 6.03E-26 | Clear cell renal cell |
| 84695 | LOXL3 | 1.585375836 | 4.23E-30 | Clear cell renal cell |
| 83593 | RASSF5 | 1.585457183 | 3.02E-34 | Clear cell renal cell |
| 8477 | GPR65 | 1.586217964 | 1.61E-29 | Clear cell renal cell |
| 942 | CD86 | 1.586669311 | 7.57E-30 | Clear cell renal cell |
| 55013 | MCUB | 1.587189238 | 2.02E-34 | Clear cell renal cell |
| 8718 | TNFRSF25 | 1.587902239 | 1.08E-15 | Clear cell renal cell |
| 4057 | LTF | 1.58799803 | 1.36E-06 | Clear cell renal cell |
| 2124 | EVI2B | 1.588104359 | 2.76E-29 | Clear cell renal cell |
| 2192 | FBLN1 | 1.588181033 | 2.73E-17 | Clear cell renal cell |
| 6348 | CCL3 | 1.589053268 | 8.66E-18 | Clear cell renal cell |
| 332 | BIRC5 | 1.589178673 | 2.81E-20 | Clear cell renal cell |
| 3779 | KCNMB1 | 1.590300946 | 3.62E-27 | Clear cell renal cell |
| 117144 | CATSPER1 | 1.590471598 | 2.20E-19 | Clear cell renal cell |
| 3118 | HLA-DQA2 | 1.590480975 | 1.63E-12 | Clear cell renal cell |
| 1380 | CR2 | 1.591679042 | 1.20E-08 | Clear cell renal cell |
| 10871 | CD300C | 1.592859253 | 2.01E-26 | Clear cell renal cell |
| 254263 | CNIH2 | 1.593191577 | 2.64E-23 | Clear cell renal cell |
| 6503 | SLA | 1.593388931 | 7.44E-34 | Clear cell renal cell |
| 90381 | TICRR | 1.594143702 | 1.04E-25 | Clear cell renal cell |
| 3669 | ISG20 | 1.59567515 | 6.96E-27 | Clear cell renal cell |
| 100 | ADA | 1.595877695 | 1.25E-28 | Clear cell renal cell |
| 2250 | FGF5 | 1.596620168 | 4.74E-09 | Clear cell renal cell |
| 9290 | GPR55 | 1.59694294 | 2.58E-24 | Clear cell renal cell |
| 778 | CACNA1F | 1.598546356 | 4.69E-11 | Clear cell renal cell |
| 5023 | P2RX1 | 1.59920472 | 5.78E-19 | Clear cell renal cell |
| 27036 | SIGLEC7 | 1.599663964 | 3.64E-24 | Clear cell renal cell |
| 1545 | CYP1B1 | 1.5999978 | 1.44E-14 | Clear cell renal cell |
| 148229 | ATP8B3 | 1.601028923 | 3.05E-12 | Clear cell renal cell |
| 169355 | IDO2 | 1.601522378 | 1.26E-16 | Clear cell renal cell |
| 6444 | SGCD | 1.601754052 | 1.79E-10 | Clear cell renal cell |
| 114132 | SIGLEC11 | 1.602514388 | 4.63E-18 | Clear cell renal cell |
| 3624 | INHBA | 1.602872831 | 9.47E-17 | Clear cell renal cell |
| 3117 | HLA-DQA1 | 1.603241759 | 5.34E-20 | Clear cell renal cell |
| 3687 | ITGAX | 1.603716175 | 1.79E-25 | Clear cell renal cell |
| 29881 | NPC1L1 | 1.604216968 | 2.24E-06 | Clear cell renal cell |
| 963 | CD53 | 1.605165997 | 1.97E-37 | Clear cell renal cell |
| 768 | CA9 | 1.60535676 | 4.08E-05 | Clear cell renal cell |
| 6398 | SECTM1 | 1.605589777 | 2.18E-25 | Clear cell renal cell |
| 165 | AEBP1 | 1.605615536 | 2.83E-13 | Clear cell renal cell |
| 64838 | FNDC4 | 1.605877106 | 3.16E-16 | Clear cell renal cell |
| 10740 | None | 1.606275939 | 6.70E-28 | Clear cell renal cell |
| 163175 | LGI4 | 1.60662329 | 1.97E-07 | Clear cell renal cell |
| 257106 | ARHGAP30 | 1.606754154 | 1.41E-33 | Clear cell renal cell |
| 148345 | C1orf127 | 1.607059256 | 4.73E-27 | Clear cell renal cell |
| 2769 | GNA15 | 1.607188194 | 2.51E-37 | Clear cell renal cell |
| 389206 | BEND4 | 1.608172198 | 8.86E-17 | Clear cell renal cell |
| 3576 | CXCL8 | 1.610091064 | 1.83E-10 | Clear cell renal cell |
| 246329 | STAC3 | 1.610247182 | 2.44E-33 | Clear cell renal cell |
| 9381 | OTOF | 1.611713215 | 6.40E-18 | Clear cell renal cell |
| 6402 | SELL | 1.612046159 | 7.16E-33 | Clear cell renal cell |
| 1308 | COL17A1 | 1.613709023 | 1.80E-09 | Clear cell renal cell |
| 1441 | CSF3R | 1.615151424 | 2.23E-29 | Clear cell renal cell |
| 148641 | SLC35F3 | 1.615158756 | 3.35E-08 | Clear cell renal cell |
| 91752 | ZNF804A | 1.615207114 | 2.12E-25 | Clear cell renal cell |
| 57126 | CD177 | 1.615286213 | 1.06E-09 | Clear cell renal cell |
| 3310 | HSPA6 | 1.615868159 | 2.13E-29 | Clear cell renal cell |
| 5228 | PGF | 1.616088995 | 1.50E-10 | Clear cell renal cell |
| 283234 | CCDC88B | 1.61691931 | 3.24E-29 | Clear cell renal cell |
| 29968 | PSAT1 | 1.617268968 | 6.20E-09 | Clear cell renal cell |
| 50649 | ARHGEF4 | 1.617379869 | 2.25E-10 | Clear cell renal cell |
| 1235 | CCR6 | 1.617617143 | 2.67E-18 | Clear cell renal cell |
| 55521 | TRIM36 | 1.617737483 | 2.53E-30 | Clear cell renal cell |
| 25903 | OLFML2B | 1.620880348 | 1.09E-26 | Clear cell renal cell |
| 1264 | CNN1 | 1.620960829 | 4.63E-16 | Clear cell renal cell |
| 960 | CD44 | 1.621059708 | 1.14E-31 | Clear cell renal cell |
| 313 | AOAH | 1.622109861 | 1.57E-24 | Clear cell renal cell |
| 7373 | COL14A1 | 1.622133163 | 4.39E-14 | Clear cell renal cell |
| 6364 | CCL20 | 1.622191812 | 1.33E-06 | Clear cell renal cell |
| 951 | CD37 | 1.622302123 | 2.95E-39 | Clear cell renal cell |
| 654433 | None | 1.622676902 | 5.01E-16 | Clear cell renal cell |
| 860 | RUNX2 | 1.622704911 | 5.34E-29 | Clear cell renal cell |
| 11040 | PIM2 | 1.623353278 | 2.21E-33 | Clear cell renal cell |
| 56097 | PCDHGC5 | 1.624422145 | 2.02E-16 | Clear cell renal cell |
| 23616 | SH3BP1 | 1.626183526 | 3.19E-44 | Clear cell renal cell |
| 9232 | PTTG1 | 1.626467226 | 3.66E-28 | Clear cell renal cell |
| 54869 | EPS8L1 | 1.62703996 | 5.02E-11 | Clear cell renal cell |
| 1062 | CENPE | 1.627261304 | 2.50E-27 | Clear cell renal cell |
| 8911 | CACNA1I | 1.627503247 | 2.82E-15 | Clear cell renal cell |
| 929 | CD14 | 1.628898464 | 5.18E-31 | Clear cell renal cell |
| 285093 | RTP5 | 1.629470349 | 1.62E-17 | Clear cell renal cell |
| 140564 | APOBEC3D | 1.629692111 | 3.21E-35 | Clear cell renal cell |
| 4046 | LSP1 | 1.629712546 | 1.81E-29 | Clear cell renal cell |
| 151056 | PLB1 | 1.629902268 | 1.73E-35 | Clear cell renal cell |
| 3738 | KCNA3 | 1.630204182 | 1.66E-19 | Clear cell renal cell |
| 79037 | PVRIG | 1.632628887 | 1.52E-34 | Clear cell renal cell |
| 285220 | EPHA6 | 1.632908079 | 2.59E-08 | Clear cell renal cell |
| 8638 | OASL | 1.633990472 | 4.12E-25 | Clear cell renal cell |
| 221472 | FGD2 | 1.634104936 | 8.64E-31 | Clear cell renal cell |
| 729230 | CCR2 | 1.634748358 | 2.54E-19 | Clear cell renal cell |
| 653390 | None | 1.635123526 | 4.42E-22 | Clear cell renal cell |
| 64221 | ROBO3 | 1.636761013 | 1.75E-20 | Clear cell renal cell |
| 7805 | LAPTM5 | 1.637773552 | 1.51E-40 | Clear cell renal cell |
| 1292 | COL6A2 | 1.638563497 | 1.21E-23 | Clear cell renal cell |
| 5347 | PLK1 | 1.640521165 | 8.69E-25 | Clear cell renal cell |
| 400759 | None | 1.641565274 | 1.23E-20 | Clear cell renal cell |
| 5806 | PTX3 | 1.641576802 | 1.98E-14 | Clear cell renal cell |
| 6688 | SPI1 | 1.642716807 | 1.30E-38 | Clear cell renal cell |
| 29933 | GPR132 | 1.643171165 | 1.20E-38 | Clear cell renal cell |
| 79930 | DOK3 | 1.644261133 | 5.09E-37 | Clear cell renal cell |
| 330 | BIRC3 | 1.645122287 | 2.42E-20 | Clear cell renal cell |
| 270 | AMPD1 | 1.646347922 | 1.02E-13 | Clear cell renal cell |
| 5742 | PTGS1 | 1.646500602 | 2.30E-15 | Clear cell renal cell |
| 79187 | FSD1 | 1.6466904 | 8.82E-15 | Clear cell renal cell |
| 221393 | ADGRF4 | 1.648580842 | 1.67E-09 | Clear cell renal cell |
| 83729 | INHBE | 1.649383208 | 5.99E-09 | Clear cell renal cell |
| 339488 | TFAP2E | 1.650551692 | 2.29E-32 | Clear cell renal cell |
| 962 | CD48 | 1.650632071 | 2.37E-29 | Clear cell renal cell |
| 221091 | LRRN4CL | 1.652699362 | 3.37E-07 | Clear cell renal cell |
| 56938 | ARNTL2 | 1.652843712 | 3.96E-20 | Clear cell renal cell |
| 246778 | IL27 | 1.653127052 | 1.14E-23 | Clear cell renal cell |
| 5446 | PON3 | 1.65333896 | 5.01E-11 | Clear cell renal cell |
| 710 | SERPING1 | 1.653505425 | 3.55E-25 | Clear cell renal cell |
| 4166 | CHST6 | 1.653575168 | 1.18E-14 | Clear cell renal cell |
| 51311 | TLR8 | 1.653593099 | 1.86E-19 | Clear cell renal cell |
| 4914 | NTRK1 | 1.654154124 | 2.19E-18 | Clear cell renal cell |
| 7504 | XK | 1.654346513 | 8.62E-11 | Clear cell renal cell |
| 163351 | GBP6 | 1.654862307 | 4.49E-22 | Clear cell renal cell |
| 3805 | KIR2DL4 | 1.655482974 | 2.67E-18 | Clear cell renal cell |
| 8522 | GAS7 | 1.6559175 | 2.47E-31 | Clear cell renal cell |
| 29108 | PYCARD | 1.656080855 | 5.26E-29 | Clear cell renal cell |
| 43 | ACHE | 1.656758215 | 7.60E-14 | Clear cell renal cell |
| 164312 | LRRN4 | 1.657183924 | 9.28E-09 | Clear cell renal cell |
| 4051 | CYP4F3 | 1.658609392 | 8.51E-07 | Clear cell renal cell |
| 115273 | RAB42 | 1.658910409 | 3.99E-10 | Clear cell renal cell |
| 2999 | GZMH | 1.659912027 | 2.13E-19 | Clear cell renal cell |
| 54739 | XAF1 | 1.66005197 | 1.66E-28 | Clear cell renal cell |
| 3004 | GZMM | 1.661101276 | 1.13E-21 | Clear cell renal cell |
| 79690 | GAL3ST4 | 1.661378531 | 2.08E-34 | Clear cell renal cell |
| 5788 | PTPRC | 1.662328248 | 2.83E-32 | Clear cell renal cell |
| 8792 | TNFRSF11A | 1.662474396 | 2.09E-21 | Clear cell renal cell |
| 1230 | CCR1 | 1.662523812 | 1.66E-23 | Clear cell renal cell |
| 72 | ACTG2 | 1.663086146 | 1.11E-12 | Clear cell renal cell |
| 1281 | COL3A1 | 1.663273958 | 1.53E-19 | Clear cell renal cell |
| 51555 | PEX5L | 1.663540724 | 1.75E-13 | Clear cell renal cell |
| 4210 | MEFV | 1.663592951 | 1.60E-27 | Clear cell renal cell |
| 3689 | ITGB2 | 1.663907259 | 8.66E-31 | Clear cell renal cell |
| 2529 | FUT7 | 1.6648534 | 6.82E-27 | Clear cell renal cell |
| 407977 | TNFSF12-TNFSF13 | 1.665970673 | 3.32E-14 | Clear cell renal cell |
| 85480 | TSLP | 1.667501542 | 1.48E-17 | Clear cell renal cell |
| 2859 | GPR35 | 1.668739306 | 2.90E-11 | Clear cell renal cell |
| 219670 | ENKUR | 1.671229758 | 2.22E-15 | Clear cell renal cell |
| 284297 | SSC5D | 1.671818664 | 5.49E-15 | Clear cell renal cell |
| 1734 | DIO2 | 1.672122433 | 7.52E-11 | Clear cell renal cell |
| 4542 | MYO1F | 1.672766831 | 8.64E-35 | Clear cell renal cell |
| 9641 | IKBKE | 1.672987851 | 2.00E-35 | Clear cell renal cell |
| 55655 | NLRP2 | 1.675777442 | 4.05E-09 | Clear cell renal cell |
| 23643 | LY96 | 1.677022936 | 1.48E-30 | Clear cell renal cell |
| 3557 | IL1RN | 1.67731132 | 6.59E-21 | Clear cell renal cell |
| 255426 | RASGEF1C | 1.677576433 | 6.02E-10 | Clear cell renal cell |
| 3752 | KCND3 | 1.677718448 | 1.07E-11 | Clear cell renal cell |
| 27033 | ZBTB32 | 1.677928614 | 4.28E-23 | Clear cell renal cell |
| 84623 | KIRREL3 | 1.679536505 | 1.99E-11 | Clear cell renal cell |
| 10874 | NMU | 1.679544727 | 1.00E-09 | Clear cell renal cell |
| 388610 | TRNP1 | 1.679733342 | 1.89E-11 | Clear cell renal cell |
| 5579 | PRKCB | 1.682580417 | 2.86E-25 | Clear cell renal cell |
| 864 | RUNX3 | 1.683186299 | 7.05E-28 | Clear cell renal cell |
| 4326 | MMP17 | 1.684834314 | 3.48E-11 | Clear cell renal cell |
| 3936 | LCP1 | 1.684970006 | 1.83E-32 | Clear cell renal cell |
| 284276 | None | 1.686402333 | 6.49E-09 | Clear cell renal cell |
| 79776 | ZFHX4 | 1.686752867 | 1.58E-14 | Clear cell renal cell |
| 3263 | HPX | 1.687915017 | 7.77E-17 | Clear cell renal cell |
| 2919 | CXCL1 | 1.689017421 | 2.10E-08 | Clear cell renal cell |
| 401115 | C4orf48 | 1.693700526 | 1.76E-12 | Clear cell renal cell |
| 27350 | APOBEC3C | 1.694410948 | 6.72E-27 | Clear cell renal cell |
| 4495 | MT1G | 1.694591462 | 1.18E-06 | Clear cell renal cell |
| 60489 | APOBEC3G | 1.694791803 | 3.84E-32 | Clear cell renal cell |
| 55355 | HJURP | 1.695441791 | 3.30E-24 | Clear cell renal cell |
| 247 | ALOX15B | 1.696259022 | 6.00E-11 | Clear cell renal cell |
| 1382 | CRABP2 | 1.696764996 | 8.09E-09 | Clear cell renal cell |
| 3821 | KLRC1 | 1.697057185 | 7.67E-19 | Clear cell renal cell |
| 645090 | None | 1.697583033 | 4.36E-11 | Clear cell renal cell |
| 1510 | CTSE | 1.699464897 | 3.03E-07 | Clear cell renal cell |
| 8284 | KDM5D | 1.700572099 | 0.001005545 | Clear cell renal cell |
| 83706 | FERMT3 | 1.702972245 | 9.83E-42 | Clear cell renal cell |
| 10024 | TROAP | 1.704071043 | 5.25E-22 | Clear cell renal cell |
| 10333 | TLR6 | 1.704841126 | 2.03E-31 | Clear cell renal cell |
| 5021 | OXTR | 1.705071828 | 1.88E-21 | Clear cell renal cell |
| 283748 | PLA2G4D | 1.706739897 | 2.68E-12 | Clear cell renal cell |
| 284417 | TMEM150B | 1.707565612 | 1.65E-21 | Clear cell renal cell |
| 3751 | KCND2 | 1.708151945 | 4.71E-11 | Clear cell renal cell |
| 55350 | VNN3 | 1.708502696 | 5.35E-17 | Clear cell renal cell |
| 64332 | NFKBIZ | 1.70946662 | 1.28E-20 | Clear cell renal cell |
| 27128 | CYTH4 | 1.709734212 | 2.59E-40 | Clear cell renal cell |
| 1278 | COL1A2 | 1.710681949 | 1.64E-19 | Clear cell renal cell |
| 133418 | EMB | 1.71167478 | 3.30E-38 | Clear cell renal cell |
| 89857 | KLHL6 | 1.711755125 | 6.42E-34 | Clear cell renal cell |
| 2535 | FZD2 | 1.714053878 | 7.64E-27 | Clear cell renal cell |
| 2207 | FCER1G | 1.715124383 | 1.30E-38 | Clear cell renal cell |
| 146850 | PIK3R6 | 1.715872648 | 8.52E-25 | Clear cell renal cell |
| 283316 | CD163L1 | 1.716709967 | 5.96E-19 | Clear cell renal cell |
| 5099 | PCDH7 | 1.71758176 | 6.33E-14 | Clear cell renal cell |
| 23460 | ABCA6 | 1.719486553 | 9.52E-15 | Clear cell renal cell |
| 5349 | FXYD3 | 1.721004878 | 2.08E-07 | Clear cell renal cell |
| 10570 | DPYSL4 | 1.721664566 | 6.23E-08 | Clear cell renal cell |
| 51127 | TRIM17 | 1.72174077 | 2.23E-14 | Clear cell renal cell |
| 221481 | ARMC12 | 1.722286382 | 1.32E-17 | Clear cell renal cell |
| 128346 | C1orf162 | 1.726324212 | 1.64E-30 | Clear cell renal cell |
| 57419 | SLC24A3 | 1.727146419 | 1.08E-13 | Clear cell renal cell |
| 4015 | LOX | 1.728003973 | 1.15E-10 | Clear cell renal cell |
| 55190 | NUDT11 | 1.72805841 | 2.76E-17 | Clear cell renal cell |
| 7103 | TSPAN8 | 1.72953853 | 1.03E-06 | Clear cell renal cell |
| 27181 | SIGLEC8 | 1.72988549 | 1.84E-19 | Clear cell renal cell |
| 84663 | None | 1.730801487 | 1.48E-06 | Clear cell renal cell |
| 6999 | TDO2 | 1.731511088 | 2.94E-10 | Clear cell renal cell |
| 51561 | IL23A | 1.732109179 | 2.29E-44 | Clear cell renal cell |
| 80774 | LIMD2 | 1.733202907 | 8.74E-35 | Clear cell renal cell |
| 10320 | IKZF1 | 1.733999927 | 2.74E-32 | Clear cell renal cell |
| 606724 | None | 1.73404281 | 2.78E-29 | Clear cell renal cell |
| 81029 | WNT5B | 1.734353118 | 1.86E-16 | Clear cell renal cell |
| 116211 | TM4SF19 | 1.735028605 | 1.99E-13 | Clear cell renal cell |
| 4502 | MT2A | 1.735223138 | 5.52E-19 | Clear cell renal cell |
| 283897 | C16orf54 | 1.736167919 | 4.87E-33 | Clear cell renal cell |
| 1821 | DRP2 | 1.736787417 | 1.65E-19 | Clear cell renal cell |
| 5329 | PLAUR | 1.736978161 | 4.10E-31 | Clear cell renal cell |
| 9902 | None | 1.73837096 | 9.89E-26 | Clear cell renal cell |
| 2706 | GJB2 | 1.740743212 | 3.53E-11 | Clear cell renal cell |
| 433 | ASGR2 | 1.740757159 | 7.57E-22 | Clear cell renal cell |
| 9358 | ITGBL1 | 1.742023856 | 5.65E-14 | Clear cell renal cell |
| 3371 | TNC | 1.747064298 | 4.20E-18 | Clear cell renal cell |
| 647121 | None | 1.747133634 | 5.74E-39 | Clear cell renal cell |
| 4489 | MT1A | 1.747452898 | 3.00E-10 | Clear cell renal cell |
| 100130958 | SYCE1L | 1.748042695 | 2.09E-12 | Clear cell renal cell |
| 4837 | NNMT | 1.748571764 | 2.10E-12 | Clear cell renal cell |
| 8875 | VNN2 | 1.749932642 | 2.44E-24 | Clear cell renal cell |
| 256236 | None | 1.750154799 | 3.68E-19 | Clear cell renal cell |
| 100128385 | None | 1.750179819 | 4.78E-29 | Clear cell renal cell |
| 5737 | PTGFR | 1.752088477 | 3.34E-11 | Clear cell renal cell |
| 84940 | CORO6 | 1.752176971 | 1.17E-11 | Clear cell renal cell |
| 1370 | CPN2 | 1.752279388 | 2.22E-05 | Clear cell renal cell |
| 752 | FMNL1 | 1.752705907 | 1.13E-43 | Clear cell renal cell |
| 114897 | C1QTNF1 | 1.757541011 | 4.76E-23 | Clear cell renal cell |
| 3357 | HTR2B | 1.764060395 | 5.84E-16 | Clear cell renal cell |
| 10371 | SEMA3A | 1.764312451 | 1.07E-13 | Clear cell renal cell |
| 353345 | GPR141 | 1.76481184 | 3.27E-23 | Clear cell renal cell |
| 80119 | PIF1 | 1.766466031 | 1.08E-23 | Clear cell renal cell |
| 5341 | PLEK | 1.766687362 | 3.10E-32 | Clear cell renal cell |
| 2810 | SFN | 1.767746505 | 8.67E-11 | Clear cell renal cell |
| 1296 | COL8A2 | 1.76799173 | 5.53E-21 | Clear cell renal cell |
| 85407 | NKD1 | 1.768310088 | 3.70E-22 | Clear cell renal cell |
| 2138 | EYA1 | 1.771005745 | 1.68E-07 | Clear cell renal cell |
| 284749 | None | 1.771885637 | 2.26E-09 | Clear cell renal cell |
| 54440 | SASH3 | 1.771973306 | 1.04E-40 | Clear cell renal cell |
| 266675 | BEST4 | 1.772983778 | 1.98E-13 | Clear cell renal cell |
| 284110 | GSDMA | 1.773086683 | 5.23E-14 | Clear cell renal cell |
| 9402 | GRAP2 | 1.775073398 | 4.45E-28 | Clear cell renal cell |
| 285533 | RNF175 | 1.775233745 | 4.46E-21 | Clear cell renal cell |
| 9536 | PTGES | 1.775424956 | 7.79E-12 | Clear cell renal cell |
| 8630 | HSD17B6 | 1.776183652 | 1.07E-17 | Clear cell renal cell |
| 79713 | IGFLR1 | 1.776262686 | 3.79E-46 | Clear cell renal cell |
| 9212 | AURKB | 1.776800711 | 1.03E-24 | Clear cell renal cell |
| 8843 | HCAR3 | 1.777466708 | 1.75E-13 | Clear cell renal cell |
| 1305 | COL13A1 | 1.777975204 | 5.02E-15 | Clear cell renal cell |
| 1414 | CRYBB1 | 1.778200915 | 3.47E-29 | Clear cell renal cell |
| 11065 | UBE2C | 1.779176307 | 6.57E-23 | Clear cell renal cell |
| 154075 | SAMD3 | 1.780660977 | 2.11E-26 | Clear cell renal cell |
| 1043 | CD52 | 1.781277221 | 4.00E-30 | Clear cell renal cell |
| 10468 | FST | 1.782140282 | 2.58E-09 | Clear cell renal cell |
| 91523 | PCED1B | 1.783637334 | 2.09E-50 | Clear cell renal cell |
| 283777 | FAM169B | 1.784412994 | 1.75E-12 | Clear cell renal cell |
| 25789 | None | 1.784700405 | 7.85E-13 | Clear cell renal cell |
| 919 | CD247 | 1.784785415 | 4.68E-30 | Clear cell renal cell |
| 126393 | HSPB6 | 1.786020709 | 3.82E-17 | Clear cell renal cell |
| 3681 | ITGAD | 1.786840598 | 6.07E-12 | Clear cell renal cell |
| 8091 | HMGA2 | 1.78743764 | 5.71E-09 | Clear cell renal cell |
| 2201 | FBN2 | 1.788211947 | 4.84E-16 | Clear cell renal cell |
| 389634 | None | 1.789831361 | 1.42E-19 | Clear cell renal cell |
| 861 | RUNX1 | 1.792138488 | 5.02E-35 | Clear cell renal cell |
| 148741 | ANKRD35 | 1.792482712 | 5.21E-23 | Clear cell renal cell |
| 2906 | GRIN2D | 1.793190942 | 1.84E-26 | Clear cell renal cell |
| 146909 | KIF18B | 1.79410258 | 1.62E-25 | Clear cell renal cell |
| 9052 | GPRC5A | 1.794631421 | 7.28E-10 | Clear cell renal cell |
| 8302 | KLRC4 | 1.798042071 | 3.13E-19 | Clear cell renal cell |
| 64170 | CARD9 | 1.799796521 | 1.54E-27 | Clear cell renal cell |
| 5996 | RGS1 | 1.800168523 | 2.97E-28 | Clear cell renal cell |
| 23046 | KIF21B | 1.800291902 | 2.16E-36 | Clear cell renal cell |
| 91156 | IGFN1 | 1.80245208 | 1.21E-06 | Clear cell renal cell |
| 1805 | DPT | 1.803082596 | 5.12E-10 | Clear cell renal cell |
| 430 | ASCL2 | 1.803537084 | 1.21E-30 | Clear cell renal cell |
| 7454 | WAS | 1.804030863 | 1.34E-41 | Clear cell renal cell |
| 653361 | NCF1 | 1.804259762 | 6.98E-26 | Clear cell renal cell |
| 5730 | PTGDS | 1.805862783 | 3.08E-11 | Clear cell renal cell |
| 8877 | None | 1.807734693 | 4.17E-24 | Clear cell renal cell |
| 350 | APOH | 1.809998767 | 2.48E-08 | Clear cell renal cell |
| 4689 | NCF4 | 1.810259983 | 7.34E-43 | Clear cell renal cell |
| 969 | CD69 | 1.810322908 | 1.06E-32 | Clear cell renal cell |
| 54072 | None | 1.812093494 | 1.15E-18 | Clear cell renal cell |
| 283078 | MKX | 1.81242472 | 8.00E-13 | Clear cell renal cell |
| 8728 | ADAM19 | 1.815429014 | 1.20E-33 | Clear cell renal cell |
| 629 | CFB | 1.815741025 | 1.77E-17 | Clear cell renal cell |
| 11025 | LILRB3 | 1.817473587 | 2.99E-35 | Clear cell renal cell |
| 80307 | None | 1.817577465 | 5.56E-11 | Clear cell renal cell |
| 151887 | CCDC80 | 1.818032027 | 1.62E-21 | Clear cell renal cell |
| 4602 | MYB | 1.819809519 | 3.05E-22 | Clear cell renal cell |
| 89790 | SIGLEC10 | 1.82004227 | 8.42E-34 | Clear cell renal cell |
| 10481 | HOXB13 | 1.820563164 | 1.24E-08 | Clear cell renal cell |
| 9955 | HS3ST3A1 | 1.822373024 | 7.35E-17 | Clear cell renal cell |
| 81607 | NECTIN4 | 1.823248489 | 1.94E-11 | Clear cell renal cell |
| 5452 | POU2F2 | 1.824891946 | 1.16E-36 | Clear cell renal cell |
| 7076 | TIMP1 | 1.825057487 | 1.59E-30 | Clear cell renal cell |
| 3627 | CXCL10 | 1.825504408 | 3.87E-18 | Clear cell renal cell |
| 11151 | CORO1A | 1.825923389 | 6.29E-37 | Clear cell renal cell |
| 11211 | FZD10 | 1.826692219 | 1.12E-15 | Clear cell renal cell |
| 200162 | SPAG17 | 1.826850669 | 1.40E-11 | Clear cell renal cell |
| 5330 | PLCB2 | 1.828946868 | 2.45E-34 | Clear cell renal cell |
| 79168 | LILRA6 | 1.829973348 | 1.35E-29 | Clear cell renal cell |
| 10870 | HCST | 1.832454496 | 3.81E-38 | Clear cell renal cell |
| 6192 | RPS4Y1 | 1.8336744 | 0.00372463 | Clear cell renal cell |
| 2162 | F13A1 | 1.833823338 | 3.59E-19 | Clear cell renal cell |
| 201501 | ZBTB7C | 1.833976465 | 1.69E-13 | Clear cell renal cell |
| 654817 | None | 1.834651926 | 8.81E-24 | Clear cell renal cell |
| 3071 | NCKAP1L | 1.836480021 | 1.53E-33 | Clear cell renal cell |
| 654816 | None | 1.837209906 | 9.64E-28 | Clear cell renal cell |
| 10216 | PRG4 | 1.83957255 | 6.17E-14 | Clear cell renal cell |
| 4016 | LOXL1 | 1.839983416 | 1.04E-21 | Clear cell renal cell |
| 201294 | UNC13D | 1.844758575 | 8.75E-34 | Clear cell renal cell |
| 6853 | SYN1 | 1.846026658 | 4.98E-23 | Clear cell renal cell |
| 100272216 | None | 1.846317478 | 3.53E-23 | Clear cell renal cell |
| 57705 | WDFY4 | 1.848799024 | 3.17E-28 | Clear cell renal cell |
| 10993 | SDS | 1.848985122 | 3.52E-20 | Clear cell renal cell |
| 9103 | FCGR2A | 1.85005168 | 4.52E-26 | Clear cell renal cell |
| 150696 | PROM2 | 1.850244174 | 4.64E-09 | Clear cell renal cell |
| 2829 | XCR1 | 1.851314017 | 2.56E-18 | Clear cell renal cell |
| 55714 | TENM3 | 1.851553237 | 9.14E-10 | Clear cell renal cell |
| 8701 | DNAH11 | 1.851645354 | 3.77E-06 | Clear cell renal cell |
| 84951 | TNS4 | 1.85519847 | 1.33E-14 | Clear cell renal cell |
| 2214 | FCGR3A | 1.85521714 | 9.27E-33 | Clear cell renal cell |
| 7409 | VAV1 | 1.855565178 | 3.69E-34 | Clear cell renal cell |
| 83879 | CDCA7 | 1.856412982 | 1.06E-27 | Clear cell renal cell |
| 54757 | FAM20A | 1.856419853 | 9.08E-23 | Clear cell renal cell |
| 54210 | TREM1 | 1.856776133 | 1.01E-15 | Clear cell renal cell |
| 9332 | CD163 | 1.858597973 | 2.85E-23 | Clear cell renal cell |
| 341 | APOC1 | 1.859045291 | 4.14E-20 | Clear cell renal cell |
| 284021 | MILR1 | 1.863570782 | 1.09E-35 | Clear cell renal cell |
| 1089 | CEACAM4 | 1.864192799 | 7.07E-23 | Clear cell renal cell |
| 81501 | DCSTAMP | 1.864733999 | 2.00E-18 | Clear cell renal cell |
| 4237 | MFAP2 | 1.867275432 | 5.94E-11 | Clear cell renal cell |
| 6338 | SCNN1B | 1.867580386 | 6.16E-08 | Clear cell renal cell |
| 959 | CD40LG | 1.868272755 | 6.01E-23 | Clear cell renal cell |
| 2048 | EPHB2 | 1.869281658 | 6.98E-27 | Clear cell renal cell |
| 3575 | IL7R | 1.869549942 | 5.14E-18 | Clear cell renal cell |
| 26575 | RGS17 | 1.87041439 | 1.15E-17 | Clear cell renal cell |
| 3903 | LAIR1 | 1.870595009 | 4.08E-41 | Clear cell renal cell |
| 283383 | ADGRD1 | 1.873039955 | 5.07E-24 | Clear cell renal cell |
| 5031 | P2RY6 | 1.873982172 | 2.93E-31 | Clear cell renal cell |
| 7857 | SCG2 | 1.874472175 | 4.72E-14 | Clear cell renal cell |
| 7804 | LRP8 | 1.876092087 | 1.40E-38 | Clear cell renal cell |
| 575 | ADGRB1 | 1.876794577 | 1.52E-11 | Clear cell renal cell |
| 10149 | ADGRG2 | 1.877764088 | 1.07E-10 | Clear cell renal cell |
| 5079 | PAX5 | 1.87838674 | 1.34E-18 | Clear cell renal cell |
| 2213 | FCGR2B | 1.878858498 | 1.05E-22 | Clear cell renal cell |
| 4703 | NEB | 1.879226887 | 6.06E-10 | Clear cell renal cell |
| 129293 | TRABD2A | 1.879369925 | 1.08E-17 | Clear cell renal cell |
| 27071 | DAPP1 | 1.879690342 | 6.45E-33 | Clear cell renal cell |
| 3823 | KLRC3 | 1.879921386 | 3.40E-24 | Clear cell renal cell |
| 326342 | None | 1.880574772 | 1.35E-16 | Clear cell renal cell |
| 80342 | TRAF3IP3 | 1.881646997 | 1.26E-39 | Clear cell renal cell |
| 4488 | MSX2 | 1.88168015 | 2.14E-15 | Clear cell renal cell |
| 1608 | DGKG | 1.881974494 | 2.28E-25 | Clear cell renal cell |
| 64094 | SMOC2 | 1.883683176 | 6.57E-21 | Clear cell renal cell |
| 51450 | PRRX2 | 1.886939793 | 1.20E-16 | Clear cell renal cell |
| 89795 | NAV3 | 1.890182104 | 3.29E-18 | Clear cell renal cell |
| 7226 | TRPM2 | 1.89342973 | 2.77E-38 | Clear cell renal cell |
| 2707 | GJB3 | 1.893493434 | 2.71E-13 | Clear cell renal cell |
| 1066 | CES1 | 1.896439987 | 1.03E-10 | Clear cell renal cell |
| 1794 | DOCK2 | 1.898447933 | 2.93E-33 | Clear cell renal cell |
| 8564 | KMO | 1.899826386 | 1.91E-14 | Clear cell renal cell |
| 8832 | CD84 | 1.900528024 | 7.95E-22 | Clear cell renal cell |
| 10631 | POSTN | 1.901053984 | 8.06E-17 | Clear cell renal cell |
| 80332 | ADAM33 | 1.90150685 | 3.59E-12 | Clear cell renal cell |
| 5616 | None | 1.902438108 | 2.60E-06 | Clear cell renal cell |
| 11117 | EMILIN1 | 1.90296838 | 3.17E-22 | Clear cell renal cell |
| 1134 | CHRNA1 | 1.904181169 | 3.38E-10 | Clear cell renal cell |
| 50619 | DEF6 | 1.905228774 | 5.27E-46 | Clear cell renal cell |
| 1731 | Sep-01 | 1.905848964 | 1.22E-39 | Clear cell renal cell |
| 9033 | PKD2L1 | 1.908950221 | 5.10E-16 | Clear cell renal cell |
| 5308 | PITX2 | 1.909238012 | 2.76E-07 | Clear cell renal cell |
| 1307 | COL16A1 | 1.910502606 | 5.43E-20 | Clear cell renal cell |
| 85449 | KIAA1755 | 1.913769212 | 4.59E-21 | Clear cell renal cell |
| 343450 | KCNT2 | 1.91418131 | 1.53E-18 | Clear cell renal cell |
| 160365 | CLECL1 | 1.915689337 | 1.51E-26 | Clear cell renal cell |
| 597 | BCL2A1 | 1.916576878 | 7.15E-30 | Clear cell renal cell |
| 2153 | F5 | 1.917799936 | 1.46E-08 | Clear cell renal cell |
| 730 | C7 | 1.921854836 | 1.59E-09 | Clear cell renal cell |
| 2903 | GRIN2A | 1.923144377 | 9.61E-10 | Clear cell renal cell |
| 6572 | SLC18A3 | 1.924548872 | 1.44E-07 | Clear cell renal cell |
| 64092 | SAMSN1 | 1.92667716 | 1.19E-41 | Clear cell renal cell |
| 90273 | CEACAM21 | 1.928136889 | 1.52E-33 | Clear cell renal cell |
| 64098 | PARVG | 1.928235371 | 1.86E-40 | Clear cell renal cell |
| 3587 | IL10RA | 1.929413024 | 3.80E-44 | Clear cell renal cell |
| 9480 | ONECUT2 | 1.933575683 | 4.07E-11 | Clear cell renal cell |
| 478 | ATP1A3 | 1.933717835 | 2.27E-14 | Clear cell renal cell |
| 55876 | GSDMB | 1.937408156 | 5.46E-22 | Clear cell renal cell |
| 9022 | CLIC3 | 1.937901666 | 1.27E-18 | Clear cell renal cell |
| 10309 | CCNO | 1.939084834 | 3.37E-11 | Clear cell renal cell |
| 1116 | CHI3L1 | 1.940021189 | 1.35E-12 | Clear cell renal cell |
| 3683 | ITGAL | 1.940248659 | 5.78E-33 | Clear cell renal cell |
| 3560 | IL2RB | 1.941150173 | 2.72E-38 | Clear cell renal cell |
| 6351 | CCL4 | 1.941766597 | 9.52E-31 | Clear cell renal cell |
| 90249 | UNC5A | 1.942062943 | 6.93E-16 | Clear cell renal cell |
| 389558 | FAM180A | 1.942308159 | 9.74E-14 | Clear cell renal cell |
| 80128 | TRIM46 | 1.943443008 | 3.83E-22 | Clear cell renal cell |
| 1233 | CCR4 | 1.94423337 | 1.23E-21 | Clear cell renal cell |
| 714 | C1QC | 1.947297502 | 3.47E-37 | Clear cell renal cell |
| 3866 | KRT15 | 1.948872394 | 4.04E-12 | Clear cell renal cell |
| 3822 | KLRC2 | 1.949877528 | 5.27E-22 | Clear cell renal cell |
| 26059 | ERC2 | 1.950056185 | 1.43E-18 | Clear cell renal cell |
| 1293 | COL6A3 | 1.951707425 | 1.99E-21 | Clear cell renal cell |
| 718 | C3 | 1.952017804 | 6.04E-19 | Clear cell renal cell |
| 29774 | None | 1.955853156 | 1.53E-18 | Clear cell renal cell |
| 1588 | CYP19A1 | 1.958203193 | 1.30E-23 | Clear cell renal cell |
| 84624 | FNDC1 | 1.958216212 | 1.63E-15 | Clear cell renal cell |
| 121355 | GTSF1 | 1.958221453 | 2.06E-21 | Clear cell renal cell |
| 3640 | INSL3 | 1.960871515 | 8.14E-25 | Clear cell renal cell |
| 255231 | MCOLN2 | 1.962971681 | 3.86E-27 | Clear cell renal cell |
| 116535 | MRGPRF | 1.963486894 | 9.30E-20 | Clear cell renal cell |
| 10317 | B3GALT5 | 1.963856107 | 5.03E-12 | Clear cell renal cell |
| 712 | C1QA | 1.964784644 | 7.92E-40 | Clear cell renal cell |
| 51702 | PADI3 | 1.965104368 | 2.95E-08 | Clear cell renal cell |
| 2786 | GNG4 | 1.966974637 | 5.32E-13 | Clear cell renal cell |
| 5740 | PTGIS | 1.967455994 | 2.90E-16 | Clear cell renal cell |
| 2533 | FYB1 | 1.96821411 | 6.26E-40 | Clear cell renal cell |
| 22806 | IKZF3 | 1.968613912 | 9.12E-20 | Clear cell renal cell |
| 54587 | MXRA8 | 1.969935988 | 4.12E-18 | Clear cell renal cell |
| 10630 | PDPN | 1.970539282 | 1.46E-14 | Clear cell renal cell |
| 3101 | HK3 | 1.971818168 | 3.24E-26 | Clear cell renal cell |
| 8482 | SEMA7A | 1.972025984 | 2.49E-44 | Clear cell renal cell |
| 11006 | LILRB4 | 1.972076734 | 6.53E-31 | Clear cell renal cell |
| 11326 | VSIG4 | 1.972686516 | 9.69E-30 | Clear cell renal cell |
| 81796 | SLCO5A1 | 1.976775937 | 1.08E-28 | Clear cell renal cell |
| 2784 | GNB3 | 1.977864623 | 1.21E-17 | Clear cell renal cell |
| 6291 | SAA4 | 1.979351812 | 2.16E-11 | Clear cell renal cell |
| 81793 | TLR10 | 1.980096378 | 9.23E-26 | Clear cell renal cell |
| 3594 | IL12RB1 | 1.980960037 | 2.58E-42 | Clear cell renal cell |
| 286122 | None | 1.98231038 | 2.82E-23 | Clear cell renal cell |
| 116372 | LYPD1 | 1.983647702 | 1.32E-20 | Clear cell renal cell |
| 84740 | None | 1.984178494 | 3.47E-13 | Clear cell renal cell |
| 343637 | RSPO4 | 1.98520192 | 8.10E-16 | Clear cell renal cell |
| 57471 | ERMN | 1.985933729 | 6.63E-24 | Clear cell renal cell |
| 8900 | CCNA1 | 1.985938618 | 5.16E-13 | Clear cell renal cell |
| 2069 | EREG | 1.988482602 | 9.53E-14 | Clear cell renal cell |
| 3766 | KCNJ10 | 1.993291708 | 3.15E-12 | Clear cell renal cell |
| 8600 | TNFSF11 | 1.994388406 | 4.27E-16 | Clear cell renal cell |
| 554202 | None | 1.995341068 | 5.24E-12 | Clear cell renal cell |
| 2841 | GPR18 | 1.995735364 | 6.66E-38 | Clear cell renal cell |
| 51176 | LEF1 | 1.996538029 | 1.25E-22 | Clear cell renal cell |
| 5549 | PRELP | 1.99736406 | 1.27E-16 | Clear cell renal cell |
| 88 | ACTN2 | 1.997542323 | 1.24E-12 | Clear cell renal cell |
| 4818 | NKG7 | 1.999953285 | 3.93E-26 | Clear cell renal cell |
| 165186 | TOGARAM2 | 2.000104597 | 5.29E-24 | Clear cell renal cell |
| 389136 | VGLL3 | 2.00132638 | 1.21E-20 | Clear cell renal cell |
| 404550 | C16orf74 | 2.004631007 | 8.82E-16 | Clear cell renal cell |
| 4969 | OGN | 2.005704219 | 1.24E-08 | Clear cell renal cell |
| 133874 | C5orf58 | 2.005939291 | 9.78E-28 | Clear cell renal cell |
| 10344 | CCL26 | 2.006175558 | 7.90E-19 | Clear cell renal cell |
| 6340 | SCNN1G | 2.009450804 | 1.93E-06 | Clear cell renal cell |
| 1559 | CYP2C9 | 2.010249123 | 1.69E-10 | Clear cell renal cell |
| 1001 | CDH3 | 2.01044644 | 8.42E-17 | Clear cell renal cell |
| 10123 | ARL4C | 2.010662914 | 2.22E-30 | Clear cell renal cell |
| 80183 | RUBCNL | 2.011241 | 1.36E-37 | Clear cell renal cell |
| 6373 | CXCL11 | 2.012565868 | 9.47E-19 | Clear cell renal cell |
| 116844 | LRG1 | 2.015317701 | 9.30E-16 | Clear cell renal cell |
| 64388 | GREM2 | 2.016380219 | 1.08E-14 | Clear cell renal cell |
| 3116 | None | 2.021482031 | 1.44E-25 | Clear cell renal cell |
| 9096 | TBX18 | 2.021794124 | 7.22E-18 | Clear cell renal cell |
| 8140 | SLC7A5 | 2.02272028 | 1.71E-18 | Clear cell renal cell |
| 497189 | TIFAB | 2.027951477 | 5.33E-25 | Clear cell renal cell |
| 7781 | SLC30A3 | 2.03123024 | 1.65E-20 | Clear cell renal cell |
| 2202 | EFEMP1 | 2.033701269 | 1.61E-18 | Clear cell renal cell |
| 2152 | F3 | 2.035763495 | 2.37E-21 | Clear cell renal cell |
| 11187 | PKP3 | 2.041063724 | 2.39E-08 | Clear cell renal cell |
| 113730 | KLHDC7B | 2.043569217 | 2.74E-25 | Clear cell renal cell |
| 6614 | SIGLEC1 | 2.045206966 | 4.30E-34 | Clear cell renal cell |
| 2015 | ADGRE1 | 2.047902981 | 1.10E-26 | Clear cell renal cell |
| 140947 | DCANP1 | 2.048281436 | 1.76E-23 | Clear cell renal cell |
| 240 | ALOX5 | 2.048988666 | 1.94E-31 | Clear cell renal cell |
| 10878 | CFHR3 | 2.049750877 | 8.81E-24 | Clear cell renal cell |
| 2019 | EN1 | 2.051460574 | 3.91E-18 | Clear cell renal cell |
| 1521 | CTSW | 2.054544221 | 2.95E-26 | Clear cell renal cell |
| 147138 | TMC8 | 2.054657088 | 1.82E-43 | Clear cell renal cell |
| 23017 | FAIM2 | 2.055393478 | 5.12E-25 | Clear cell renal cell |
| 64333 | ARHGAP9 | 2.056257131 | 2.26E-46 | Clear cell renal cell |
| 5063 | PAK3 | 2.056853724 | 1.16E-18 | Clear cell renal cell |
| 9840 | TESPA1 | 2.058118696 | 6.68E-27 | Clear cell renal cell |
| 57167 | SALL4 | 2.058304037 | 7.16E-16 | Clear cell renal cell |
| 55359 | STYK1 | 2.059100573 | 8.31E-28 | Clear cell renal cell |
| 5880 | RAC2 | 2.060874865 | 2.01E-43 | Clear cell renal cell |
| 8510 | MMP23B | 2.061255338 | 5.03E-24 | Clear cell renal cell |
| 10290 | SPEG | 2.065646862 | 5.41E-21 | Clear cell renal cell |
| 2318 | FLNC | 2.067480573 | 7.15E-16 | Clear cell renal cell |
| 338 | APOB | 2.067569677 | 1.60E-07 | Clear cell renal cell |
| 5968 | REG1B | 2.069418695 | 3.14E-13 | Clear cell renal cell |
| 8771 | TNFRSF6B | 2.072985176 | 9.31E-18 | Clear cell renal cell |
| 245 | None | 2.073355692 | 8.59E-15 | Clear cell renal cell |
| 4606 | MYBPC2 | 2.077199517 | 7.96E-17 | Clear cell renal cell |
| 25907 | TMEM158 | 2.077210376 | 9.92E-27 | Clear cell renal cell |
| 244 | None | 2.077325439 | 2.19E-11 | Clear cell renal cell |
| 1289 | COL5A1 | 2.078476619 | 5.11E-24 | Clear cell renal cell |
| 5365 | PLXNB3 | 2.07900638 | 1.16E-18 | Clear cell renal cell |
| 85479 | DNAJC5B | 2.080773746 | 1.52E-20 | Clear cell renal cell |
| 923 | CD6 | 2.081352678 | 8.61E-34 | Clear cell renal cell |
| 321 | APBA2 | 2.081475882 | 3.60E-23 | Clear cell renal cell |
| 2662 | GDF10 | 2.083310652 | 7.77E-18 | Clear cell renal cell |
| 84941 | HSH2D | 2.084394945 | 1.97E-35 | Clear cell renal cell |
| 7051 | TGM1 | 2.08669931 | 1.24E-13 | Clear cell renal cell |
| 101 | ADAM8 | 2.08702961 | 3.50E-36 | Clear cell renal cell |
| 85409 | NKD2 | 2.090076089 | 6.67E-10 | Clear cell renal cell |
| 713 | C1QB | 2.091591607 | 9.71E-42 | Clear cell renal cell |
| 23650 | TRIM29 | 2.091746396 | 1.33E-11 | Clear cell renal cell |
| 286467 | None | 2.092464303 | 1.22E-22 | Clear cell renal cell |
| 150726 | FBXO41 | 2.094015023 | 2.20E-23 | Clear cell renal cell |
| 64919 | BCL11B | 2.094015441 | 2.61E-33 | Clear cell renal cell |
| 7036 | TFR2 | 2.094898374 | 3.00E-12 | Clear cell renal cell |
| 340547 | VSIG1 | 2.094942139 | 3.06E-17 | Clear cell renal cell |
| 22914 | KLRK1 | 2.09683504 | 5.20E-33 | Clear cell renal cell |
| 5396 | PRRX1 | 2.098260858 | 1.69E-23 | Clear cell renal cell |
| 5307 | PITX1 | 2.099052584 | 8.45E-11 | Clear cell renal cell |
| 3311 | None | 2.100667759 | 7.65E-22 | Clear cell renal cell |
| 27040 | LAT | 2.101108801 | 5.96E-37 | Clear cell renal cell |
| 10071 | MUC12 | 2.101814757 | 1.01E-14 | Clear cell renal cell |
| 4192 | MDK | 2.104505116 | 1.41E-23 | Clear cell renal cell |
| 8542 | APOL1 | 2.1046458 | 5.24E-32 | Clear cell renal cell |
| 6690 | SPINK1 | 2.104706577 | 2.64E-08 | Clear cell renal cell |
| 8111 | GPR68 | 2.104962364 | 4.66E-54 | Clear cell renal cell |
| 5008 | OSM | 2.108970366 | 3.36E-27 | Clear cell renal cell |
| 7062 | TCHH | 2.112702528 | 7.74E-24 | Clear cell renal cell |
| 7066 | THPO | 2.120361638 | 7.10E-13 | Clear cell renal cell |
| 827 | CAPN6 | 2.122344256 | 6.21E-08 | Clear cell renal cell |
| 164118 | TTC24 | 2.123745079 | 3.79E-26 | Clear cell renal cell |
| 940 | CD28 | 2.124880615 | 2.48E-37 | Clear cell renal cell |
| 3001 | GZMA | 2.125048032 | 6.97E-31 | Clear cell renal cell |
| 727936 | GXYLT2 | 2.125948361 | 1.30E-15 | Clear cell renal cell |
| 1378 | CR1 | 2.127309757 | 2.54E-23 | Clear cell renal cell |
| 26191 | PTPN22 | 2.130463467 | 2.39E-36 | Clear cell renal cell |
| 8530 | CST7 | 2.131474789 | 1.67E-31 | Clear cell renal cell |
| 23495 | TNFRSF13B | 2.131592898 | 1.01E-23 | Clear cell renal cell |
| 10267 | RAMP1 | 2.131620923 | 6.64E-16 | Clear cell renal cell |
| 1236 | CCR7 | 2.132069258 | 1.68E-31 | Clear cell renal cell |
| 8745 | ADAM23 | 2.135329253 | 2.04E-18 | Clear cell renal cell |
| 286333 | None | 2.140246368 | 4.74E-36 | Clear cell renal cell |
| 256380 | SCML4 | 2.140726178 | 1.21E-34 | Clear cell renal cell |
| 57537 | SORCS2 | 2.141174209 | 5.87E-11 | Clear cell renal cell |
| 115350 | FCRL1 | 2.141717373 | 2.75E-23 | Clear cell renal cell |
| 29909 | GPR171 | 2.141854314 | 6.36E-38 | Clear cell renal cell |
| 79054 | TRPM8 | 2.145339572 | 1.09E-17 | Clear cell renal cell |
| 342615 | None | 2.146271994 | 3.92E-33 | Clear cell renal cell |
| 6564 | SLC15A1 | 2.147943165 | 4.26E-08 | Clear cell renal cell |
| 11227 | GALNT5 | 2.148406014 | 5.99E-11 | Clear cell renal cell |
| 84958 | SYTL1 | 2.150041881 | 2.28E-26 | Clear cell renal cell |
| 124460 | SNX20 | 2.151151461 | 3.18E-40 | Clear cell renal cell |
| 5317 | PKP1 | 2.151910187 | 1.26E-10 | Clear cell renal cell |
| 114794 | ELFN2 | 2.152729075 | 8.33E-11 | Clear cell renal cell |
| 63910 | SLC17A9 | 2.153690446 | 7.27E-25 | Clear cell renal cell |
| 79574 | EPS8L3 | 2.154606293 | 6.37E-08 | Clear cell renal cell |
| 5540 | NPY4R | 2.154645335 | 8.33E-21 | Clear cell renal cell |
| 64005 | MYO1G | 2.158615633 | 4.02E-39 | Clear cell renal cell |
| 653145 | ANXA8 | 2.16114037 | 1.46E-11 | Clear cell renal cell |
| 2173 | FABP7 | 2.162334779 | 8.89E-05 | Clear cell renal cell |
| 84830 | ADTRP | 2.163136689 | 1.62E-17 | Clear cell renal cell |
| 10846 | PDE10A | 2.163446049 | 1.01E-21 | Clear cell renal cell |
| 387357 | THEMIS | 2.167424388 | 2.48E-28 | Clear cell renal cell |
| 254439 | C11orf86 | 2.168041902 | 4.01E-11 | Clear cell renal cell |
| 4605 | MYBL2 | 2.169905028 | 8.20E-31 | Clear cell renal cell |
| 338707 | B4GALNT4 | 2.170326722 | 2.69E-09 | Clear cell renal cell |
| 3702 | ITK | 2.170465204 | 8.53E-37 | Clear cell renal cell |
| 79370 | BCL2L14 | 2.180222941 | 3.41E-36 | Clear cell renal cell |
| 3783 | KCNN4 | 2.182551577 | 2.53E-30 | Clear cell renal cell |
| 91683 | SYT12 | 2.183625229 | 1.66E-17 | Clear cell renal cell |
| 55076 | TMEM45A | 2.188799312 | 1.78E-19 | Clear cell renal cell |
| 128434 | VSTM2L | 2.189424911 | 1.63E-13 | Clear cell renal cell |
| 118932 | ANKRD22 | 2.194115778 | 1.85E-19 | Clear cell renal cell |
| 374403 | TBC1D10C | 2.19783299 | 2.84E-40 | Clear cell renal cell |
| 3976 | LIF | 2.198627052 | 1.09E-23 | Clear cell renal cell |
| 1844 | DUSP2 | 2.199752388 | 3.32E-36 | Clear cell renal cell |
| 25823 | TPSG1 | 2.2009338 | 3.28E-09 | Clear cell renal cell |
| 3718 | JAK3 | 2.203357265 | 3.66E-40 | Clear cell renal cell |
| 81849 | ST6GALNAC5 | 2.205066475 | 8.38E-17 | Clear cell renal cell |
| 2210 | FCGR1B | 2.206162469 | 1.20E-47 | Clear cell renal cell |
| 338773 | TMEM119 | 2.20676219 | 2.67E-26 | Clear cell renal cell |
| 401124 | DTHD1 | 2.206798234 | 2.95E-25 | Clear cell renal cell |
| 6474 | SHOX2 | 2.216554643 | 1.13E-20 | Clear cell renal cell |
| 202299 | None | 2.217751584 | 1.03E-09 | Clear cell renal cell |
| 2672 | GFI1 | 2.217908204 | 2.46E-41 | Clear cell renal cell |
| 5054 | SERPINE1 | 2.218003589 | 9.22E-24 | Clear cell renal cell |
| 3581 | IL9R | 2.219412575 | 7.50E-39 | Clear cell renal cell |
| 441168 | CALHM6 | 2.219567521 | 3.80E-34 | Clear cell renal cell |
| 340075 | ARSI | 2.219573027 | 3.09E-14 | Clear cell renal cell |
| 115908 | CTHRC1 | 2.220457762 | 2.66E-21 | Clear cell renal cell |
| 145864 | HAPLN3 | 2.222827631 | 7.89E-45 | Clear cell renal cell |
| 79092 | CARD14 | 2.223146323 | 5.13E-22 | Clear cell renal cell |
| 128611 | ZNF831 | 2.224890886 | 5.16E-32 | Clear cell renal cell |
| 9890 | PLPPR4 | 2.229276394 | 1.66E-26 | Clear cell renal cell |
| 92737 | DNER | 2.229697882 | 3.86E-10 | Clear cell renal cell |
| 941 | CD80 | 2.231723018 | 2.18E-38 | Clear cell renal cell |
| 131450 | CD200R1 | 2.231977217 | 4.31E-31 | Clear cell renal cell |
| 3755 | KCNG1 | 2.235121295 | 1.77E-15 | Clear cell renal cell |
| 645432 | ARRDC5 | 2.236161199 | 1.37E-33 | Clear cell renal cell |
| 1234 | CCR5 | 2.23713882 | 8.09E-33 | Clear cell renal cell |
| 7535 | ZAP70 | 2.2372534 | 3.71E-32 | Clear cell renal cell |
| 139728 | PNCK | 2.238450562 | 2.54E-08 | Clear cell renal cell |
| 284656 | EPHA10 | 2.239156472 | 1.89E-12 | Clear cell renal cell |
| 3932 | LCK | 2.23921745 | 1.24E-33 | Clear cell renal cell |
| 399 | RHOH | 2.24257992 | 1.79E-46 | Clear cell renal cell |
| 9427 | ECEL1 | 2.242795021 | 4.90E-16 | Clear cell renal cell |
| 971 | CD72 | 2.244966779 | 1.91E-47 | Clear cell renal cell |
| 6285 | S100B | 2.246356069 | 1.80E-29 | Clear cell renal cell |
| 55057 | CRYBG2 | 2.247132965 | 6.72E-23 | Clear cell renal cell |
| 132332 | TMEM155 | 2.248208606 | 4.85E-22 | Clear cell renal cell |
| 3026 | HABP2 | 2.249532472 | 5.05E-07 | Clear cell renal cell |
| 3586 | IL10 | 2.2497349 | 5.36E-38 | Clear cell renal cell |
| 100233209 | None | 2.251627161 | 1.62E-40 | Clear cell renal cell |
| 338442 | HCAR2 | 2.253772975 | 1.62E-19 | Clear cell renal cell |
| 50863 | NTM | 2.257162819 | 1.95E-19 | Clear cell renal cell |
| 2172 | FABP6 | 2.264751261 | 1.68E-11 | Clear cell renal cell |
| 8685 | MARCO | 2.265556714 | 7.19E-13 | Clear cell renal cell |
| 29943 | PADI1 | 2.271923445 | 7.43E-12 | Clear cell renal cell |
| 5790 | PTPRCAP | 2.272548117 | 1.80E-35 | Clear cell renal cell |
| 55908 | ANGPTL8 | 2.276260531 | 6.45E-10 | Clear cell renal cell |
| 100132417 | None | 2.279134924 | 5.68E-47 | Clear cell renal cell |
| 6356 | CCL11 | 2.280081083 | 3.60E-19 | Clear cell renal cell |
| 2847 | MCHR1 | 2.282726061 | 1.75E-09 | Clear cell renal cell |
| 348938 | NIPAL4 | 2.282805263 | 4.23E-18 | Clear cell renal cell |
| 56978 | PRDM8 | 2.28531039 | 4.74E-36 | Clear cell renal cell |
| 6846 | XCL2 | 2.287504934 | 8.02E-32 | Clear cell renal cell |
| 79368 | FCRL2 | 2.288669826 | 3.88E-21 | Clear cell renal cell |
| 6508 | SLC4A3 | 2.292404381 | 4.11E-12 | Clear cell renal cell |
| 286133 | SCARA5 | 2.294934955 | 2.32E-18 | Clear cell renal cell |
| 257101 | ZNF683 | 2.302395484 | 1.25E-25 | Clear cell renal cell |
| 2191 | FAP | 2.302437444 | 2.90E-27 | Clear cell renal cell |
| 1118 | CHIT1 | 2.304227674 | 1.15E-09 | Clear cell renal cell |
| 5156 | PDGFRA | 2.304648242 | 1.26E-16 | Clear cell renal cell |
| 6689 | SPIB | 2.30469035 | 1.93E-25 | Clear cell renal cell |
| 3037 | HAS2 | 2.310493701 | 3.23E-23 | Clear cell renal cell |
| 6374 | CXCL5 | 2.310610433 | 1.94E-08 | Clear cell renal cell |
| 8537 | BCAS1 | 2.310694451 | 5.30E-14 | Clear cell renal cell |
| 56667 | MUC13 | 2.312548169 | 4.53E-14 | Clear cell renal cell |
| 3561 | IL2RG | 2.312861312 | 2.80E-42 | Clear cell renal cell |
| 164668 | APOBEC3H | 2.313653714 | 2.87E-36 | Clear cell renal cell |
| 80326 | WNT10A | 2.314347774 | 2.16E-24 | Clear cell renal cell |
| 3075 | CFH | 2.317508618 | 1.01E-31 | Clear cell renal cell |
| 64127 | NOD2 | 2.317648675 | 7.37E-42 | Clear cell renal cell |
| 54900 | LAX1 | 2.319901557 | 4.35E-32 | Clear cell renal cell |
| 24141 | LAMP5 | 2.32102394 | 1.02E-16 | Clear cell renal cell |
| 85016 | CFAP300 | 2.321047214 | 9.11E-22 | Clear cell renal cell |
| 149628 | PYHIN1 | 2.321648936 | 3.22E-32 | Clear cell renal cell |
| 344 | APOC2 | 2.321949636 | 2.27E-21 | Clear cell renal cell |
| 921 | CD5 | 2.333833969 | 2.90E-34 | Clear cell renal cell |
| 7345 | UCHL1 | 2.335762923 | 6.91E-15 | Clear cell renal cell |
| 9760 | TOX | 2.337057007 | 7.84E-30 | Clear cell renal cell |
| 5360 | PLTP | 2.338447993 | 1.92E-29 | Clear cell renal cell |
| 6447 | SCG5 | 2.339309776 | 5.25E-19 | Clear cell renal cell |
| 2900 | GRIK4 | 2.339456229 | 2.34E-17 | Clear cell renal cell |
| 4063 | LY9 | 2.341012675 | 1.01E-38 | Clear cell renal cell |
| 1748 | DLX4 | 2.342070548 | 2.88E-26 | Clear cell renal cell |
| 4050 | LTB | 2.344247729 | 4.78E-32 | Clear cell renal cell |
| 9832 | JAKMIP2 | 2.344531909 | 5.57E-33 | Clear cell renal cell |
| 10663 | CXCR6 | 2.34653449 | 2.13E-37 | Clear cell renal cell |
| 64866 | CDCP1 | 2.347868241 | 6.75E-21 | Clear cell renal cell |
| 7058 | THBS2 | 2.347975575 | 1.19E-22 | Clear cell renal cell |
| 90853 | SPOCD1 | 2.348525062 | 2.15E-27 | Clear cell renal cell |
| 645784 | None | 2.351100132 | 5.95E-21 | Clear cell renal cell |
| 6352 | CCL5 | 2.355209882 | 1.86E-37 | Clear cell renal cell |
| 221336 | BEND6 | 2.355491979 | 1.86E-27 | Clear cell renal cell |
| 10643 | IGF2BP3 | 2.356154919 | 1.72E-18 | Clear cell renal cell |
| 2209 | FCGR1A | 2.35628749 | 1.41E-43 | Clear cell renal cell |
| 10673 | TNFSF13B | 2.356808137 | 5.91E-41 | Clear cell renal cell |
| 10225 | CD96 | 2.357389907 | 7.43E-40 | Clear cell renal cell |
| 924 | CD7 | 2.361660336 | 3.70E-37 | Clear cell renal cell |
| 10409 | BASP1 | 2.363063848 | 8.23E-42 | Clear cell renal cell |
| 1277 | COL1A1 | 2.364267431 | 3.93E-25 | Clear cell renal cell |
| 6036 | RNASE2 | 2.365708975 | 2.35E-37 | Clear cell renal cell |
| 55240 | STEAP3 | 2.367370029 | 4.53E-28 | Clear cell renal cell |
| 165631 | PARP15 | 2.369554173 | 7.98E-36 | Clear cell renal cell |
| 222865 | TMEM130 | 2.371812874 | 7.24E-12 | Clear cell renal cell |
| 917 | CD3G | 2.371841437 | 7.41E-30 | Clear cell renal cell |
| 92211 | CDHR1 | 2.373505018 | 6.86E-11 | Clear cell renal cell |
| 1237 | CCR8 | 2.374481525 | 9.21E-38 | Clear cell renal cell |
| 7634 | ZNF80 | 2.375315484 | 1.06E-33 | Clear cell renal cell |
| 838 | CASP5 | 2.375907968 | 1.30E-36 | Clear cell renal cell |
| 57535 | KIAA1324 | 2.376737899 | 5.71E-33 | Clear cell renal cell |
| 4283 | CXCL9 | 2.379983561 | 2.67E-27 | Clear cell renal cell |
| 356 | FASLG | 2.381599555 | 7.68E-32 | Clear cell renal cell |
| 6504 | SLAMF1 | 2.387948351 | 6.29E-40 | Clear cell renal cell |
| 57689 | LRRC4C | 2.388817782 | 5.09E-22 | Clear cell renal cell |
| 5367 | PMCH | 2.388819966 | 1.75E-21 | Clear cell renal cell |
| 164656 | TMPRSS6 | 2.389915545 | 1.88E-24 | Clear cell renal cell |
| 84174 | SLA2 | 2.391067048 | 7.03E-45 | Clear cell renal cell |
| 1401 | CRP | 2.393080216 | 2.33E-12 | Clear cell renal cell |
| 5798 | PTPRN | 2.395001594 | 2.86E-11 | Clear cell renal cell |
| 114836 | SLAMF6 | 2.396664065 | 3.74E-38 | Clear cell renal cell |
| 4068 | SH2D1A | 2.397464865 | 3.03E-34 | Clear cell renal cell |
| 5157 | PDGFRL | 2.398072461 | 1.34E-27 | Clear cell renal cell |
| 63924 | CIDEC | 2.400923695 | 1.98E-13 | Clear cell renal cell |
| 64220 | STRA6 | 2.410291971 | 2.90E-20 | Clear cell renal cell |
| 91319 | DERL3 | 2.411180065 | 8.50E-37 | Clear cell renal cell |
| 9744 | ACAP1 | 2.412448549 | 3.32E-41 | Clear cell renal cell |
| 50615 | IL21R | 2.414176728 | 9.26E-42 | Clear cell renal cell |
| 11075 | STMN2 | 2.414321941 | 1.64E-15 | Clear cell renal cell |
| 4885 | NPTX2 | 2.416572916 | 1.66E-12 | Clear cell renal cell |
| 8601 | RGS20 | 2.423872436 | 1.01E-23 | Clear cell renal cell |
| 84433 | CARD11 | 2.427095709 | 2.38E-37 | Clear cell renal cell |
| 25975 | EGFL6 | 2.429262709 | 4.70E-14 | Clear cell renal cell |
| 27334 | P2RY10 | 2.436973852 | 1.91E-37 | Clear cell renal cell |
| 9051 | PSTPIP1 | 2.440590002 | 3.57E-36 | Clear cell renal cell |
| 11262 | SP140 | 2.441161681 | 3.64E-55 | Clear cell renal cell |
| 2331 | FMOD | 2.447525178 | 2.13E-26 | Clear cell renal cell |
| 148113 | CILP2 | 2.448183355 | 1.56E-18 | Clear cell renal cell |
| 126306 | JSRP1 | 2.448512493 | 1.26E-17 | Clear cell renal cell |
| 84636 | GPR174 | 2.449615463 | 4.25E-33 | Clear cell renal cell |
| 1946 | EFNA5 | 2.450337525 | 4.48E-17 | Clear cell renal cell |
| 4547 | MTTP | 2.450916011 | 4.98E-11 | Clear cell renal cell |
| 6588 | SLN | 2.452730212 | 1.23E-15 | Clear cell renal cell |
| 4321 | MMP12 | 2.452974618 | 7.73E-15 | Clear cell renal cell |
| 85352 | SHISAL1 | 2.456898603 | 1.70E-18 | Clear cell renal cell |
| 389058 | SP5 | 2.460039445 | 2.15E-15 | Clear cell renal cell |
| 8320 | EOMES | 2.460453162 | 5.15E-30 | Clear cell renal cell |
| 56666 | PANX2 | 2.461092582 | 6.84E-16 | Clear cell renal cell |
| 84561 | SLC12A8 | 2.461287274 | 1.38E-23 | Clear cell renal cell |
| 3914 | LAMB3 | 2.462429492 | 6.62E-25 | Clear cell renal cell |
| 6357 | CCL13 | 2.465142514 | 6.96E-24 | Clear cell renal cell |
| 6489 | ST8SIA1 | 2.466552282 | 5.15E-39 | Clear cell renal cell |
| 151888 | BTLA | 2.468395322 | 2.20E-41 | Clear cell renal cell |
| 11184 | MAP4K1 | 2.469121709 | 4.35E-38 | Clear cell renal cell |
| 57823 | SLAMF7 | 2.474213134 | 1.17E-39 | Clear cell renal cell |
| 4316 | MMP7 | 2.475372785 | 2.22E-15 | Clear cell renal cell |
| 56253 | CRTAM | 2.475768764 | 5.42E-39 | Clear cell renal cell |
| 5778 | PTPN7 | 2.479601045 | 5.80E-46 | Clear cell renal cell |
| 914 | CD2 | 2.483018723 | 5.95E-34 | Clear cell renal cell |
| 7480 | WNT10B | 2.487731426 | 3.61E-28 | Clear cell renal cell |
| 56833 | SLAMF8 | 2.496296174 | 9.27E-51 | Clear cell renal cell |
| 51676 | ASB2 | 2.505220163 | 1.47E-40 | Clear cell renal cell |
| 23149 | FCHO1 | 2.509485186 | 2.30E-33 | Clear cell renal cell |
| 8740 | TNFSF14 | 2.512860297 | 1.34E-28 | Clear cell renal cell |
| 3671 | ISLR | 2.518500739 | 2.35E-22 | Clear cell renal cell |
| 135398 | C6orf141 | 2.519904708 | 3.64E-25 | Clear cell renal cell |
| 3580 | None | 2.529681128 | 1.80E-20 | Clear cell renal cell |
| 6375 | XCL1 | 2.531334109 | 7.33E-33 | Clear cell renal cell |
| 400696 | None | 2.531432744 | 1.96E-21 | Clear cell renal cell |
| 79949 | PLEKHS1 | 2.531463889 | 1.62E-19 | Clear cell renal cell |
| 57214 | CEMIP | 2.541631113 | 4.85E-24 | Clear cell renal cell |
| 121551 | BTBD11 | 2.543268726 | 1.74E-26 | Clear cell renal cell |
| 3706 | ITPKA | 2.545883505 | 9.69E-19 | Clear cell renal cell |
| 26002 | MOXD1 | 2.547659253 | 1.34E-25 | Clear cell renal cell |
| 150365 | MEI1 | 2.549167642 | 1.79E-42 | Clear cell renal cell |
| 916 | CD3E | 2.550179121 | 1.39E-37 | Clear cell renal cell |
| 115352 | FCRL3 | 2.550598815 | 7.80E-26 | Clear cell renal cell |
| 8784 | TNFRSF18 | 2.55232976 | 5.52E-35 | Clear cell renal cell |
| 8076 | MFAP5 | 2.561646578 | 1.74E-17 | Clear cell renal cell |
| 3112 | HLA-DOB | 2.562877999 | 7.81E-37 | Clear cell renal cell |
| 3290 | HSD11B1 | 2.563761277 | 9.68E-26 | Clear cell renal cell |
| 4239 | MFAP4 | 2.572817051 | 1.19E-18 | Clear cell renal cell |
| 55065 | SLC52A1 | 2.573393431 | 5.50E-22 | Clear cell renal cell |
| 6362 | CCL18 | 2.574073054 | 3.84E-14 | Clear cell renal cell |
| 53831 | GPR84 | 2.576721804 | 3.22E-45 | Clear cell renal cell |
| 1117 | CHI3L2 | 2.576971092 | 2.49E-22 | Clear cell renal cell |
| 100188949 | None | 2.587845496 | 3.34E-47 | Clear cell renal cell |
| 127435 | PODN | 2.591322669 | 7.44E-26 | Clear cell renal cell |
| 728239 | MAGED4 | 2.59243286 | 1.63E-17 | Clear cell renal cell |
| 640 | BLK | 2.593809365 | 3.63E-29 | Clear cell renal cell |
| 6695 | SPOCK1 | 2.596919384 | 1.24E-18 | Clear cell renal cell |
| 22917 | ZP1 | 2.603958512 | 2.18E-20 | Clear cell renal cell |
| 374407 | DNAJB13 | 2.604307948 | 1.46E-12 | Clear cell renal cell |
| 50852 | TRAT1 | 2.624782456 | 6.90E-37 | Clear cell renal cell |
| 57159 | TRIM54 | 2.624784724 | 8.81E-13 | Clear cell renal cell |
| 4049 | LTA | 2.631470547 | 2.18E-49 | Clear cell renal cell |
| 119 | ADD2 | 2.638313433 | 1.05E-27 | Clear cell renal cell |
| 84628 | NTNG2 | 2.646512106 | 1.36E-42 | Clear cell renal cell |
| 9047 | SH2D2A | 2.650133123 | 2.10E-42 | Clear cell renal cell |
| 915 | CD3D | 2.654253141 | 5.21E-34 | Clear cell renal cell |
| 259232 | NALCN | 2.655655335 | 5.31E-24 | Clear cell renal cell |
| 203100 | HTRA4 | 2.657313072 | 8.09E-30 | Clear cell renal cell |
| 10538 | BATF | 2.660564242 | 2.08E-47 | Clear cell renal cell |
| 22891 | ZNF365 | 2.661543961 | 1.97E-26 | Clear cell renal cell |
| 27132 | CPNE7 | 2.661747619 | 1.29E-23 | Clear cell renal cell |
| 79827 | CLMP | 2.662765987 | 7.00E-15 | Clear cell renal cell |
| 89822 | KCNK17 | 2.669428633 | 6.22E-21 | Clear cell renal cell |
| 1634 | DCN | 2.670552248 | 1.55E-18 | Clear cell renal cell |
| 5368 | PNOC | 2.671330512 | 1.30E-28 | Clear cell renal cell |
| 2571 | GAD1 | 2.673574119 | 2.65E-13 | Clear cell renal cell |
| 8908 | GYG2 | 2.676346312 | 1.38E-16 | Clear cell renal cell |
| 4747 | NEFL | 2.685483022 | 5.49E-14 | Clear cell renal cell |
| 7221 | None | 2.685566973 | 2.70E-17 | Clear cell renal cell |
| 64699 | TMPRSS3 | 2.687028331 | 1.07E-18 | Clear cell renal cell |
| 162461 | TMEM92 | 2.689277962 | 3.33E-15 | Clear cell renal cell |
| 56265 | CPXM1 | 2.690536698 | 7.97E-23 | Clear cell renal cell |
| 5320 | PLA2G2A | 2.696524978 | 2.55E-17 | Clear cell renal cell |
| 131578 | LRRC15 | 2.697034822 | 5.62E-18 | Clear cell renal cell |
| 643 | CXCR5 | 2.69776472 | 4.33E-43 | Clear cell renal cell |
| 57863 | CADM3 | 2.698076624 | 3.60E-16 | Clear cell renal cell |
| 5522 | PPP2R2C | 2.698498714 | 4.21E-19 | Clear cell renal cell |
| 54575 | UGT1A10 | 2.70001496 | 5.63E-14 | Clear cell renal cell |
| 114614 | None | 2.70439865 | 6.01E-36 | Clear cell renal cell |
| 5176 | SERPINF1 | 2.715252883 | 1.11E-41 | Clear cell renal cell |
| 11341 | SCRG1 | 2.717796218 | 2.15E-24 | Clear cell renal cell |
| 3699 | ITIH3 | 2.718205097 | 2.66E-24 | Clear cell renal cell |
| 4241 | MELTF | 2.718613024 | 9.80E-25 | Clear cell renal cell |
| 952 | CD38 | 2.718850403 | 1.54E-36 | Clear cell renal cell |
| 27240 | SIT1 | 2.722871519 | 6.06E-36 | Clear cell renal cell |
| 27286 | SRPX2 | 2.723817147 | 4.84E-26 | Clear cell renal cell |
| 8532 | CPZ | 2.740280335 | 8.28E-18 | Clear cell renal cell |
| 81557 | MAGED4B | 2.745506723 | 1.36E-16 | Clear cell renal cell |
| 256714 | MAP7D2 | 2.759931448 | 1.33E-13 | Clear cell renal cell |
| 5047 | PAEP | 2.763987323 | 5.32E-14 | Clear cell renal cell |
| 57722 | IGDCC4 | 2.77001513 | 3.93E-19 | Clear cell renal cell |
| 925 | CD8A | 2.775390555 | 2.88E-34 | Clear cell renal cell |
| 84631 | SLITRK2 | 2.780377981 | 1.73E-16 | Clear cell renal cell |
| 84824 | FCRLA | 2.78310844 | 1.51E-33 | Clear cell renal cell |
| 7850 | IL1R2 | 2.783648683 | 3.22E-19 | Clear cell renal cell |
| 115362 | GBP5 | 2.783925869 | 2.29E-42 | Clear cell renal cell |
| 53347 | UBASH3A | 2.792656627 | 4.43E-39 | Clear cell renal cell |
| 1300 | COL10A1 | 2.79906951 | 2.60E-21 | Clear cell renal cell |
| 112714 | TUBA3E | 2.803524663 | 1.04E-13 | Clear cell renal cell |
| 79883 | PODNL1 | 2.811462227 | 5.49E-25 | Clear cell renal cell |
| 2833 | CXCR3 | 2.811641151 | 5.13E-38 | Clear cell renal cell |
| 931 | MS4A1 | 2.812387487 | 5.13E-31 | Clear cell renal cell |
| 926 | CD8B | 2.814317194 | 2.88E-33 | Clear cell renal cell |
| 4753 | NELL2 | 2.821474113 | 6.83E-34 | Clear cell renal cell |
| 54102 | CLIC6 | 2.822791177 | 9.62E-17 | Clear cell renal cell |
| 4143 | MAT1A | 2.836516294 | 1.51E-14 | Clear cell renal cell |
| 29851 | ICOS | 2.836731995 | 1.87E-41 | Clear cell renal cell |
| 970 | None | 2.840932183 | 2.35E-14 | Clear cell renal cell |
| 81030 | ZBP1 | 2.846229783 | 1.13E-46 | Clear cell renal cell |
| 608 | TNFRSF17 | 2.850030107 | 3.88E-27 | Clear cell renal cell |
| 84879 | MFSD2A | 2.850187259 | 1.05E-26 | Clear cell renal cell |
| 930 | CD19 | 2.851536649 | 1.82E-25 | Clear cell renal cell |
| 54039 | PCBP3 | 2.855468505 | 6.13E-19 | Clear cell renal cell |
| 152789 | JAKMIP1 | 2.857692918 | 1.58E-38 | Clear cell renal cell |
| 55034 | MOCOS | 2.867939932 | 1.43E-24 | Clear cell renal cell |
| 7490 | WT1 | 2.870050474 | 2.13E-16 | Clear cell renal cell |
| 4920 | ROR2 | 2.870863715 | 7.15E-22 | Clear cell renal cell |
| 492307 | PPDPFL | 2.874822353 | 3.55E-11 | Clear cell renal cell |
| 3559 | IL2RA | 2.877016149 | 1.58E-38 | Clear cell renal cell |
| 283422 | None | 2.879994672 | 1.79E-12 | Clear cell renal cell |
| 164284 | APCDD1L | 2.881765868 | 1.70E-19 | Clear cell renal cell |
| 201633 | TIGIT | 2.887171707 | 1.46E-43 | Clear cell renal cell |
| 5266 | PI3 | 2.900430476 | 1.51E-17 | Clear cell renal cell |
| 3662 | IRF4 | 2.902878817 | 2.30E-43 | Clear cell renal cell |
| 285180 | RUFY4 | 2.907437378 | 6.36E-31 | Clear cell renal cell |
| 12 | SERPINA3 | 2.916491149 | 1.17E-18 | Clear cell renal cell |
| 366 | AQP9 | 2.917702741 | 4.15E-26 | Clear cell renal cell |
| 79931 | TNIP3 | 2.930270695 | 1.11E-31 | Clear cell renal cell |
| 3381 | IBSP | 2.935408142 | 8.99E-24 | Clear cell renal cell |
| 440823 | None | 2.946624569 | 8.48E-48 | Clear cell renal cell |
| 4318 | MMP9 | 2.967351422 | 4.73E-28 | Clear cell renal cell |
| 146206 | CARMIL2 | 2.968627931 | 7.63E-45 | Clear cell renal cell |
| 51050 | PI15 | 2.969399576 | 5.93E-24 | Clear cell renal cell |
| 26579 | MYEOV | 2.971488859 | 1.92E-13 | Clear cell renal cell |
| 3787 | KCNS1 | 2.97496112 | 1.08E-14 | Clear cell renal cell |
| 715 | C1R | 2.983119693 | 2.58E-42 | Clear cell renal cell |
| 3003 | GZMK | 2.99023838 | 3.16E-33 | Clear cell renal cell |
| 85329 | LGALS12 | 3.00194748 | 1.83E-18 | Clear cell renal cell |
| 25884 | CHRDL2 | 3.00296276 | 5.78E-21 | Clear cell renal cell |
| 716 | C1S | 3.031662428 | 9.94E-38 | Clear cell renal cell |
| 5918 | RARRES1 | 3.034651547 | 1.13E-37 | Clear cell renal cell |
| 7138 | TNNT1 | 3.036170028 | 1.49E-16 | Clear cell renal cell |
| 1493 | CTLA4 | 3.037084877 | 4.24E-45 | Clear cell renal cell |
| 113457 | TUBA3D | 3.040452307 | 4.18E-13 | Clear cell renal cell |
| 153218 | SPINK13 | 3.043588517 | 4.04E-17 | Clear cell renal cell |
| 5967 | REG1A | 3.046761588 | 7.87E-12 | Clear cell renal cell |
| 1674 | DES | 3.072745285 | 5.91E-17 | Clear cell renal cell |
| 57817 | HAMP | 3.074731944 | 1.09E-31 | Clear cell renal cell |
| 717 | C2 | 3.077805209 | 1.30E-39 | Clear cell renal cell |
| 140766 | ADAMTS14 | 3.083920829 | 1.06E-42 | Clear cell renal cell |
| 4060 | LUM | 3.085183767 | 2.85E-23 | Clear cell renal cell |
| 5744 | PTHLH | 3.092358577 | 4.91E-18 | Clear cell renal cell |
| 7018 | TF | 3.093436083 | 1.78E-15 | Clear cell renal cell |
| 387695 | C10orf99 | 3.097983145 | 1.56E-12 | Clear cell renal cell |
| 5794 | PTPRH | 3.127715523 | 3.47E-19 | Clear cell renal cell |
| 50943 | FOXP3 | 3.141399705 | 4.10E-53 | Clear cell renal cell |
| 939 | CD27 | 3.147359255 | 4.29E-46 | Clear cell renal cell |
| 169044 | COL22A1 | 3.156806045 | 1.74E-24 | Clear cell renal cell |
| 9834 | None | 3.181590743 | 5.32E-31 | Clear cell renal cell |
| 3880 | KRT19 | 3.192065936 | 3.47E-15 | Clear cell renal cell |
| 8038 | ADAM12 | 3.193579745 | 2.91E-40 | Clear cell renal cell |
| 91851 | CHRDL1 | 3.194485343 | 9.96E-15 | Clear cell renal cell |
| 55423 | SIRPG | 3.206151009 | 3.96E-41 | Clear cell renal cell |
| 9945 | GFPT2 | 3.209676592 | 1.49E-34 | Clear cell renal cell |
| 6262 | RYR2 | 3.217769473 | 1.49E-28 | Clear cell renal cell |
| 1294 | COL7A1 | 3.236334434 | 5.87E-23 | Clear cell renal cell |
| 5133 | PDCD1 | 3.236867195 | 5.58E-40 | Clear cell renal cell |
| 3700 | ITIH4 | 3.241073714 | 1.01E-31 | Clear cell renal cell |
| 2147 | F2 | 3.257362774 | 9.24E-16 | Clear cell renal cell |
| 3604 | TNFRSF9 | 3.304129914 | 1.12E-36 | Clear cell renal cell |
| 973 | CD79A | 3.320196713 | 5.33E-34 | Clear cell renal cell |
| 6423 | SFRP2 | 3.327086389 | 3.02E-17 | Clear cell renal cell |
| 3902 | LAG3 | 3.331211288 | 4.26E-44 | Clear cell renal cell |
| 10568 | SLC34A2 | 3.332814242 | 2.14E-13 | Clear cell renal cell |
| 144347 | RFLNA | 3.337552736 | 1.96E-27 | Clear cell renal cell |
| 96610 | None | 3.358371858 | 3.27E-32 | Clear cell renal cell |
| 3512 | JCHAIN | 3.36243496 | 1.62E-29 | Clear cell renal cell |
| 22986 | SORCS3 | 3.368830687 | 5.52E-12 | Clear cell renal cell |
| 27299 | ADAMDEC1 | 3.381500501 | 1.39E-32 | Clear cell renal cell |
| 3458 | IFNG | 3.401564148 | 7.32E-39 | Clear cell renal cell |
| 10882 | C1QL1 | 3.402680416 | 1.94E-21 | Clear cell renal cell |
| 9447 | AIM2 | 3.42946727 | 1.62E-51 | Clear cell renal cell |
| 1311 | COMP | 3.439238766 | 4.73E-21 | Clear cell renal cell |
| 145270 | PRIMA1 | 3.507359326 | 4.39E-17 | Clear cell renal cell |
| 1301 | COL11A1 | 3.525352535 | 9.37E-21 | Clear cell renal cell |
| 3484 | IGFBP1 | 3.540768071 | 2.21E-16 | Clear cell renal cell |
| 23532 | PRAME | 3.559879156 | 1.46E-16 | Clear cell renal cell |
| 1356 | CP | 3.574440642 | 1.20E-17 | Clear cell renal cell |
| 3569 | IL6 | 3.638610753 | 3.76E-28 | Clear cell renal cell |
| 7045 | TGFBI | 3.686917864 | 9.25E-40 | Clear cell renal cell |
| 6366 | CCL21 | 3.77163645 | 3.83E-21 | Clear cell renal cell |
| 5450 | POU2AF1 | 3.797678482 | 2.46E-39 | Clear cell renal cell |
| 26279 | PLA2G2D | 3.798432578 | 8.11E-39 | Clear cell renal cell |
| 51200 | CPA4 | 3.871668535 | 3.43E-28 | Clear cell renal cell |
| 389336 | C5orf46 | 3.926219895 | 6.32E-21 | Clear cell renal cell |
| 79413 | ZBED2 | 3.934589409 | 4.90E-49 | Clear cell renal cell |
| 92745 | SLC38A5 | 3.946170953 | 1.43E-36 | Clear cell renal cell |
| 83416 | FCRL5 | 3.993288374 | 2.54E-38 | Clear cell renal cell |
| 8755 | None | 4.020591927 | 1.42E-37 | Clear cell renal cell |
| 26585 | GREM1 | 4.045454849 | 9.12E-35 | Clear cell renal cell |
| 8839 | CCN5 | 4.051656021 | 4.37E-34 | Clear cell renal cell |
| 51237 | MZB1 | 4.053927259 | 1.05E-37 | Clear cell renal cell |
| 2056 | EPO | 4.06345891 | 2.92E-21 | Clear cell renal cell |
| 53833 | IL20RB | 4.176821721 | 1.45E-32 | Clear cell renal cell |
| 2244 | FGB | 4.190655572 | 7.97E-13 | Clear cell renal cell |
| 5502 | PPP1R1A | 4.238639827 | 3.73E-19 | Clear cell renal cell |
| 6363 | CCL19 | 4.282021159 | 1.32E-37 | Clear cell renal cell |
| 2243 | FGA | 4.31871868 | 4.42E-17 | Clear cell renal cell |
| 2266 | FGG | 4.359779371 | 1.27E-14 | Clear cell renal cell |
| 6590 | SLPI | 4.438051183 | 1.01E-23 | Clear cell renal cell |
| 10563 | CXCL13 | 4.482691488 | 5.24E-39 | Clear cell renal cell |
| 3240 | HP | 4.496471147 | 2.87E-23 | Clear cell renal cell |
| 3929 | LBP | 4.532379944 | 2.96E-19 | Clear cell renal cell |
| 6289 | SAA2 | 4.799162302 | 3.20E-24 | Clear cell renal cell |
| 6288 | SAA1 | 5.841130615 | 5.63E-31 | Clear cell renal cell |
| 3512 | JCHAIN | -3.863532628 | 6.59E-20 | Endometrium |
| 8755 | None | -3.791252894 | 9.96E-20 | Endometrium |
| 10563 | CXCL13 | -3.404169691 | 1.03E-14 | Endometrium |
| 3003 | GZMK | -3.309252071 | 3.12E-27 | Endometrium |
| 1469 | CST1 | -3.244286366 | 6.87E-08 | Endometrium |
| 83416 | FCRL5 | -3.222648145 | 6.10E-20 | Endometrium |
| 26279 | PLA2G2D | -3.206021257 | 6.72E-15 | Endometrium |
| 1493 | CTLA4 | -3.17655859 | 1.71E-29 | Endometrium |
| 973 | CD79A | -3.163687992 | 3.45E-20 | Endometrium |
| 6366 | CCL21 | -3.162982013 | 2.44E-12 | Endometrium |
| 3662 | IRF4 | -3.152906224 | 1.68E-27 | Endometrium |
| 96610 | None | -3.115977866 | 1.42E-17 | Endometrium |
| 5133 | PDCD1 | -3.083700644 | 1.24E-27 | Endometrium |
| 3240 | HP | -3.081737168 | 9.52E-09 | Endometrium |
| 5450 | POU2AF1 | -3.079382127 | 3.60E-24 | Endometrium |
| 931 | MS4A1 | -3.064900484 | 2.96E-18 | Endometrium |
| 51237 | MZB1 | -3.032761571 | 1.95E-15 | Endometrium |
| 1472 | CST4 | -3.028597958 | 2.15E-08 | Endometrium |
| 55423 | SIRPG | -3.008910453 | 4.97E-27 | Endometrium |
| 4283 | CXCL9 | -3.008370168 | 9.76E-16 | Endometrium |
| 8530 | CST7 | -2.949972737 | 4.33E-37 | Endometrium |
| 3002 | GZMB | -2.947772971 | 4.00E-24 | Endometrium |
| 915 | CD3D | -2.936012833 | 4.35E-27 | Endometrium |
| 50615 | IL21R | -2.919900819 | 2.73E-33 | Endometrium |
| 921 | CD5 | -2.910953175 | 2.68E-33 | Endometrium |
| 2532 | ACKR1 | -2.910221525 | 5.34E-19 | Endometrium |
| 6504 | SLAMF1 | -2.904919521 | 7.87E-34 | Endometrium |
| 1470 | CST2 | -2.901220808 | 5.56E-10 | Endometrium |
| 7535 | ZAP70 | -2.87103718 | 6.76E-31 | Endometrium |
| 2833 | CXCR3 | -2.867771114 | 6.08E-27 | Endometrium |
| 114836 | SLAMF6 | -2.845129219 | 1.18E-27 | Endometrium |
| 916 | CD3E | -2.844904577 | 1.55E-31 | Endometrium |
| 643 | CXCR5 | -2.841395623 | 2.86E-27 | Endometrium |
| 3250 | HPR | -2.835349575 | 2.39E-08 | Endometrium |
| 6363 | CCL19 | -2.834541886 | 3.25E-16 | Endometrium |
| 4068 | SH2D1A | -2.832487514 | 1.08E-25 | Endometrium |
| 201633 | TIGIT | -2.816997201 | 1.87E-25 | Endometrium |
| 3001 | GZMA | -2.79789519 | 1.40E-26 | Endometrium |
| 6373 | CXCL11 | -2.792893908 | 1.23E-17 | Endometrium |
| 4063 | LY9 | -2.787288004 | 6.90E-27 | Endometrium |
| 22914 | KLRK1 | -2.780483946 | 9.02E-27 | Endometrium |
| 57823 | SLAMF7 | -2.768093689 | 1.44E-23 | Endometrium |
| 3004 | GZMM | -2.758042793 | 6.10E-31 | Endometrium |
| 10663 | CXCR6 | -2.756174665 | 5.01E-29 | Endometrium |
| 1236 | CCR7 | -2.755032027 | 1.74E-24 | Endometrium |
| 914 | CD2 | -2.729160163 | 2.47E-27 | Endometrium |
| 729230 | CCR2 | -2.722826883 | 2.29E-23 | Endometrium |
| 128611 | ZNF831 | -2.721206818 | 8.89E-29 | Endometrium |
| 53347 | UBASH3A | -2.719316781 | 4.04E-29 | Endometrium |
| 9840 | TESPA1 | -2.70237357 | 2.19E-32 | Endometrium |
| 29851 | ICOS | -2.702183106 | 9.23E-29 | Endometrium |
| 257101 | ZNF683 | -2.698508621 | 2.87E-23 | Endometrium |
| 6288 | SAA1 | -2.694760638 | 2.93E-09 | Endometrium |
| 3702 | ITK | -2.684897743 | 1.18E-29 | Endometrium |
| 256380 | SCML4 | -2.680706168 | 1.65E-27 | Endometrium |
| 939 | CD27 | -2.679537035 | 7.17E-28 | Endometrium |
| 256236 | None | -2.678128873 | 3.03E-10 | Endometrium |
| 27240 | SIT1 | -2.669112611 | 7.86E-24 | Endometrium |
| 27334 | P2RY10 | -2.64950402 | 3.29E-29 | Endometrium |
| 8807 | IL18RAP | -2.644916033 | 2.77E-29 | Endometrium |
| 149628 | PYHIN1 | -2.63448442 | 3.83E-25 | Endometrium |
| 6289 | SAA2 | -2.630652433 | 1.67E-09 | Endometrium |
| 283897 | C16orf54 | -2.627657409 | 6.79E-30 | Endometrium |
| 7177 | TPSAB1 | -2.625289876 | 1.79E-16 | Endometrium |
| 1117 | CHI3L2 | -2.619964051 | 2.63E-16 | Endometrium |
| 356 | FASLG | -2.618332572 | 1.01E-23 | Endometrium |
| 4818 | NKG7 | -2.617042389 | 5.34E-26 | Endometrium |
| 2999 | GZMH | -2.613851925 | 1.32E-21 | Endometrium |
| 6358 | CCL14 | -2.608491931 | 5.33E-14 | Endometrium |
| 399 | RHOH | -2.607814794 | 2.79E-34 | Endometrium |
| 27121 | DKK4 | -2.603519064 | 0.00013256 | Endometrium |
| 5788 | PTPRC | -2.598290983 | 2.03E-30 | Endometrium |
| 6402 | SELL | -2.586040937 | 2.90E-26 | Endometrium |
| 115352 | FCRL3 | -2.580937933 | 2.48E-23 | Endometrium |
| 64499 | TPSB2 | -2.579926121 | 5.41E-16 | Endometrium |
| 3594 | IL12RB1 | -2.578987736 | 1.07E-30 | Endometrium |
| 1755 | DMBT1 | -2.576249536 | 2.26E-07 | Endometrium |
| 387357 | THEMIS | -2.568557035 | 2.99E-25 | Endometrium |
| 5284 | PIGR | -2.564380768 | 1.28E-05 | Endometrium |
| 3820 | KLRB1 | -2.558505677 | 3.81E-23 | Endometrium |
| 925 | CD8A | -2.553039946 | 2.29E-23 | Endometrium |
| 53829 | P2RY13 | -2.528603998 | 5.40E-25 | Endometrium |
| 80008 | TMEM156 | -2.521038204 | 4.58E-25 | Endometrium |
| 10578 | GNLY | -2.51886542 | 2.41E-20 | Endometrium |
| 29909 | GPR171 | -2.516683027 | 3.09E-20 | Endometrium |
| 84824 | FCRLA | -2.515752722 | 2.34E-17 | Endometrium |
| 924 | CD7 | -2.514559541 | 2.16E-27 | Endometrium |
| 3117 | HLA-DQA1 | -2.500835326 | 5.31E-24 | Endometrium |
| 50852 | TRAT1 | -2.484419949 | 5.28E-23 | Endometrium |
| 30009 | TBX21 | -2.483484566 | 2.96E-25 | Endometrium |
| 120425 | JAML | -2.480309865 | 3.61E-28 | Endometrium |
| 7412 | VCAM1 | -2.475227385 | 2.27E-24 | Endometrium |
| 959 | CD40LG | -2.472310431 | 1.76E-25 | Endometrium |
| 4057 | LTF | -2.465705776 | 5.02E-05 | Endometrium |
| 5551 | PRF1 | -2.462808922 | 4.18E-29 | Endometrium |
| 286530 | P2RY8 | -2.456913429 | 1.08E-27 | Endometrium |
| 84174 | SLA2 | -2.453783212 | 1.42E-27 | Endometrium |
| 3575 | IL7R | -2.448909526 | 5.00E-21 | Endometrium |
| 12 | SERPINA3 | -2.442587615 | 1.55E-06 | Endometrium |
| 445347 | TRGC1 | -2.439340916 | 3.99E-21 | Endometrium |
| 3560 | IL2RB | -2.429007466 | 9.74E-32 | Endometrium |
| 3738 | KCNA3 | -2.426281842 | 5.60E-26 | Endometrium |
| 919 | CD247 | -2.41630691 | 6.58E-33 | Endometrium |
| 5023 | P2RX1 | -2.41592336 | 1.13E-19 | Endometrium |
| 608 | TNFRSF17 | -2.411237944 | 1.16E-14 | Endometrium |
| 5923 | RASGRF1 | -2.41112391 | 5.59E-11 | Endometrium |
| 3561 | IL2RG | -2.409135374 | 2.57E-28 | Endometrium |
| 926 | CD8B | -2.407208141 | 6.48E-16 | Endometrium |
| 100233209 | None | -2.405302663 | 1.45E-29 | Endometrium |
| 10537 | UBD | -2.402536535 | 3.87E-12 | Endometrium |
| 2124 | EVI2B | -2.395298832 | 1.18E-27 | Endometrium |
| 1439 | CSF2RB | -2.394440728 | 1.03E-30 | Endometrium |
| 26191 | PTPN22 | -2.393508944 | 1.66E-22 | Endometrium |
| 81030 | ZBP1 | -2.39068389 | 3.91E-18 | Endometrium |
| 64092 | SAMSN1 | -2.390597313 | 1.09E-29 | Endometrium |
| 140947 | DCANP1 | -2.385253848 | 1.15E-18 | Endometrium |
| 9447 | AIM2 | -2.382550342 | 2.21E-18 | Endometrium |
| 347 | APOD | -2.381637306 | 1.43E-11 | Endometrium |
| 54900 | LAX1 | -2.380037123 | 1.41E-21 | Endometrium |
| 117289 | TAGAP | -2.379510844 | 3.33E-30 | Endometrium |
| 6352 | CCL5 | -2.378724734 | 1.83E-24 | Endometrium |
| 3118 | HLA-DQA2 | -2.377317738 | 6.93E-21 | Endometrium |
| 10462 | CLEC10A | -2.372390405 | 8.44E-19 | Endometrium |
| 9051 | PSTPIP1 | -2.371768156 | 1.05E-28 | Endometrium |
| 64926 | RASAL3 | -2.369195763 | 1.96E-36 | Endometrium |
| 3683 | ITGAL | -2.363629799 | 2.65E-27 | Endometrium |
| 81793 | TLR10 | -2.35829899 | 1.18E-22 | Endometrium |
| 917 | CD3G | -2.356814994 | 1.95E-25 | Endometrium |
| 387751 | None | -2.354178222 | 1.18E-27 | Endometrium |
| 944 | TNFSF8 | -2.352494715 | 1.76E-20 | Endometrium |
| 9834 | None | -2.352004373 | 6.76E-13 | Endometrium |
| 9595 | CYTIP | -2.350708295 | 1.31E-32 | Endometrium |
| 10538 | BATF | -2.343382184 | 3.52E-23 | Endometrium |
| 6424 | SFRP4 | -2.343284025 | 1.73E-07 | Endometrium |
| 64388 | GREM2 | -2.339017775 | 4.88E-06 | Endometrium |
| 1234 | CCR5 | -2.335014301 | 1.81E-28 | Endometrium |
| 343413 | FCRL6 | -2.334455958 | 1.33E-18 | Endometrium |
| 89790 | SIGLEC10 | -2.333130372 | 1.03E-29 | Endometrium |
| 6279 | S100A8 | -2.33181413 | 3.39E-10 | Endometrium |
| 8698 | S1PR4 | -2.331383534 | 7.15E-30 | Endometrium |
| 1521 | CTSW | -2.329187707 | 6.09E-21 | Endometrium |
| 3559 | IL2RA | -2.325704841 | 3.16E-24 | Endometrium |
| 962 | CD48 | -2.323971188 | 9.06E-29 | Endometrium |
| 51744 | CD244 | -2.323687964 | 2.07E-25 | Endometrium |
| 1440 | CSF3 | -2.315129541 | 1.00E-08 | Endometrium |
| 3627 | CXCL10 | -2.314068917 | 1.55E-15 | Endometrium |
| 51411 | BIN2 | -2.30586175 | 6.88E-29 | Endometrium |
| 2533 | FYB1 | -2.292532523 | 2.97E-21 | Endometrium |
| 6401 | SELE | -2.288684174 | 1.42E-18 | Endometrium |
| 64333 | ARHGAP9 | -2.287913368 | 2.39E-34 | Endometrium |
| 54440 | SASH3 | -2.286328042 | 7.65E-36 | Endometrium |
| 6689 | SPIB | -2.285939204 | 9.48E-15 | Endometrium |
| 3128 | None | -2.285382701 | 1.19E-15 | Endometrium |
| 3112 | HLA-DOB | -2.281785913 | 2.89E-18 | Endometrium |
| 165631 | PARP15 | -2.281751037 | 1.45E-24 | Endometrium |
| 10225 | CD96 | -2.279634887 | 2.00E-26 | Endometrium |
| 9402 | GRAP2 | -2.277526349 | 3.33E-26 | Endometrium |
| 3604 | TNFRSF9 | -2.276713819 | 2.57E-23 | Endometrium |
| 90273 | CEACAM21 | -2.276232343 | 4.56E-10 | Endometrium |
| 5579 | PRKCB | -2.275232415 | 2.14E-23 | Endometrium |
| 640 | BLK | -2.27237357 | 1.46E-15 | Endometrium |
| 3119 | HLA-DQB1 | -2.267964733 | 1.84E-17 | Endometrium |
| 114614 | None | -2.267369004 | 1.05E-19 | Endometrium |
| 5169 | ENPP3 | -2.266679023 | 1.70E-06 | Endometrium |
| 313 | AOAH | -2.263097013 | 4.81E-22 | Endometrium |
| 6693 | SPN | -2.260960671 | 9.75E-30 | Endometrium |
| 115362 | GBP5 | -2.259692351 | 1.53E-16 | Endometrium |
| 952 | CD38 | -2.253117148 | 7.02E-15 | Endometrium |
| 10875 | FGL2 | -2.252752903 | 3.11E-23 | Endometrium |
| 1880 | GPR183 | -2.251986713 | 6.58E-31 | Endometrium |
| 51311 | TLR8 | -2.247185419 | 4.09E-18 | Endometrium |
| 5778 | PTPN7 | -2.244684096 | 5.23E-29 | Endometrium |
| 3113 | HLA-DPA1 | -2.240827261 | 9.21E-25 | Endometrium |
| 923 | CD6 | -2.240517424 | 6.97E-26 | Endometrium |
| 10320 | IKZF1 | -2.237027208 | 8.66E-29 | Endometrium |
| 1621 | DBH | -2.235476002 | 3.04E-10 | Endometrium |
| 389643 | NUGGC | -2.235001655 | 1.92E-18 | Endometrium |
| 56253 | CRTAM | -2.2315527 | 3.49E-19 | Endometrium |
| 5790 | PTPRCAP | -2.229326469 | 8.26E-26 | Endometrium |
| 1359 | CPA3 | -2.228516702 | 2.38E-13 | Endometrium |
| 3902 | LAG3 | -2.224232152 | 2.38E-20 | Endometrium |
| 27299 | ADAMDEC1 | -2.223328915 | 2.01E-13 | Endometrium |
| 3937 | LCP2 | -2.220175997 | 6.91E-37 | Endometrium |
| 54959 | ODAM | -2.21342434 | 2.70E-07 | Endometrium |
| 2123 | EVI2A | -2.209329853 | 1.18E-24 | Endometrium |
| 4250 | SCGB2A2 | -2.201030861 | 1.98E-05 | Endometrium |
| 124460 | SNX20 | -2.2006775 | 1.16E-35 | Endometrium |
| 10288 | LILRB2 | -2.198448889 | 2.16E-30 | Endometrium |
| 10562 | OLFM4 | -2.197063888 | 0.000189499 | Endometrium |
| 4049 | LTA | -2.195086457 | 2.80E-23 | Endometrium |
| 23430 | TPSD1 | -2.194837321 | 4.78E-13 | Endometrium |
| 150365 | MEI1 | -2.193637162 | 9.30E-22 | Endometrium |
| 3586 | IL10 | -2.189921448 | 2.94E-18 | Endometrium |
| 2248 | FGF3 | -2.184053501 | 5.31E-06 | Endometrium |
| 27319 | BHLHE22 | -2.181108864 | 1.52E-17 | Endometrium |
| 2529 | FUT7 | -2.180865425 | 1.26E-24 | Endometrium |
| 55843 | ARHGAP15 | -2.175824586 | 2.67E-26 | Endometrium |
| 10859 | LILRB1 | -2.173110464 | 4.11E-34 | Endometrium |
| 940 | CD28 | -2.171892504 | 2.33E-25 | Endometrium |
| 6403 | SELP | -2.168206486 | 2.24E-19 | Endometrium |
| 57817 | HAMP | -2.167271791 | 4.19E-13 | Endometrium |
| 5265 | SERPINA1 | -2.167067858 | 2.24E-10 | Endometrium |
| 79368 | FCRL2 | -2.161512499 | 4.84E-11 | Endometrium |
| 8477 | GPR65 | -2.153224421 | 8.11E-30 | Endometrium |
| 151888 | BTLA | -2.151856718 | 4.01E-19 | Endometrium |
| 3127 | HLA-DRB5 | -2.148980922 | 4.49E-17 | Endometrium |
| 9046 | DOK2 | -2.147617229 | 1.53E-30 | Endometrium |
| 6362 | CCL18 | -2.147393664 | 1.58E-09 | Endometrium |
| 55340 | GIMAP5 | -2.147158605 | 3.21E-31 | Endometrium |
| 4316 | MMP7 | -2.146420234 | 2.61E-06 | Endometrium |
| 3458 | IFNG | -2.14631826 | 4.45E-16 | Endometrium |
| 3957 | LGALS2 | -2.142658652 | 1.61E-13 | Endometrium |
| 497189 | TIFAB | -2.140143649 | 1.03E-18 | Endometrium |
| 54518 | APBB1IP | -2.139587219 | 3.33E-28 | Endometrium |
| 3111 | HLA-DOA | -2.136434001 | 2.24E-17 | Endometrium |
| 168537 | GIMAP7 | -2.135400248 | 2.55E-25 | Endometrium |
| 2841 | GPR18 | -2.131641221 | 1.61E-24 | Endometrium |
| 154075 | SAMD3 | -2.126229975 | 3.83E-21 | Endometrium |
| 1794 | DOCK2 | -2.124704946 | 8.85E-26 | Endometrium |
| 3587 | IL10RA | -2.118151464 | 4.83E-30 | Endometrium |
| 6846 | XCL2 | -2.116354732 | 8.46E-17 | Endometrium |
| 5294 | PIK3CG | -2.115596576 | 3.56E-23 | Endometrium |
| 8740 | TNFSF14 | -2.114635072 | 5.30E-15 | Endometrium |
| 27128 | CYTH4 | -2.113798492 | 4.63E-35 | Endometrium |
| 3137 | None | -2.109222313 | 2.64E-12 | Endometrium |
| 3071 | NCKAP1L | -2.107827579 | 7.75E-30 | Endometrium |
| 1591 | CYP24A1 | -2.10477319 | 6.22E-08 | Endometrium |
| 1805 | DPT | -2.10342388 | 2.58E-10 | Endometrium |
| 3932 | LCK | -2.102793211 | 4.68E-25 | Endometrium |
| 730 | C7 | -2.099656046 | 7.94E-09 | Endometrium |
| 930 | CD19 | -2.097345418 | 3.13E-11 | Endometrium |
| 9744 | ACAP1 | -2.089613311 | 1.62E-29 | Endometrium |
| 79168 | LILRA6 | -2.088648397 | 2.50E-19 | Endometrium |
| 259197 | NCR3 | -2.087148609 | 4.24E-19 | Endometrium |
| 6351 | CCL4 | -2.086667314 | 4.61E-22 | Endometrium |
| 3248 | HPGD | -2.082951217 | 1.57E-05 | Endometrium |
| 695 | BTK | -2.082935907 | 3.44E-25 | Endometrium |
| 9547 | CXCL14 | -2.082858018 | 4.75E-06 | Endometrium |
| 80231 | CXorf21 | -2.081479752 | 1.44E-23 | Endometrium |
| 1510 | CTSE | -2.080736213 | 1.50E-07 | Endometrium |
| 8320 | EOMES | -2.079144906 | 1.17E-18 | Endometrium |
| 219972 | MPEG1 | -2.079027639 | 9.20E-29 | Endometrium |
| 4332 | MNDA | -2.075402745 | 2.48E-24 | Endometrium |
| 5730 | PTGDS | -2.073861831 | 9.88E-10 | Endometrium |
| 6367 | CCL22 | -2.072309535 | 9.51E-14 | Endometrium |
| 388372 | CCL4L2 | -2.07209009 | 2.34E-20 | Endometrium |
| 146722 | CD300LF | -2.068762553 | 1.45E-21 | Endometrium |
| 974 | CD79B | -2.068232865 | 7.94E-17 | Endometrium |
| 3822 | KLRC2 | -2.068085878 | 1.06E-20 | Endometrium |
| 3115 | HLA-DPB1 | -2.066862443 | 2.07E-24 | Endometrium |
| 3123 | HLA-DRB1 | -2.066115113 | 8.76E-20 | Endometrium |
| 6503 | SLA | -2.061455478 | 9.96E-32 | Endometrium |
| 93035 | PKHD1L1 | -2.060501944 | 0.000332021 | Endometrium |
| 1240 | CMKLR1 | -2.058394876 | 1.39E-25 | Endometrium |
| 344 | APOC2 | -2.057189194 | 1.21E-13 | Endometrium |
| 713 | C1QB | -2.048847437 | 2.74E-22 | Endometrium |
| 80380 | PDCD1LG2 | -2.048185117 | 1.30E-22 | Endometrium |
| 4689 | NCF4 | -2.045284806 | 3.52E-29 | Endometrium |
| 4542 | MYO1F | -2.04472029 | 4.11E-38 | Endometrium |
| 3603 | IL16 | -2.044603264 | 1.58E-30 | Endometrium |
| 440823 | None | -2.044417477 | 3.38E-19 | Endometrium |
| 3120 | HLA-DQB2 | -2.043730227 | 9.92E-17 | Endometrium |
| 1233 | CCR4 | -2.043600471 | 9.49E-19 | Endometrium |
| 85449 | KIAA1755 | -2.042519877 | 1.03E-16 | Endometrium |
| 4050 | LTB | -2.040936432 | 2.55E-14 | Endometrium |
| 4069 | LYZ | -2.040800085 | 1.24E-13 | Endometrium |
| 80833 | APOL3 | -2.038488447 | 7.67E-25 | Endometrium |
| 963 | CD53 | -2.037056885 | 1.21E-31 | Endometrium |
| 433 | ASGR2 | -2.035176094 | 5.02E-18 | Endometrium |
| 22797 | TFEC | -2.034115974 | 1.67E-16 | Endometrium |
| 10261 | IGSF6 | -2.033117 | 2.06E-21 | Endometrium |
| 2672 | GFI1 | -2.031898239 | 6.25E-25 | Endometrium |
| 5996 | RGS1 | -2.026554002 | 8.77E-19 | Endometrium |
| 1378 | CR1 | -2.025347096 | 2.12E-14 | Endometrium |
| 7940 | LST1 | -2.024075852 | 4.92E-29 | Endometrium |
| 64231 | MS4A6A | -2.023327005 | 6.40E-27 | Endometrium |
| 3821 | KLRC1 | -2.022406824 | 1.70E-19 | Endometrium |
| 712 | C1QA | -2.022379985 | 7.59E-23 | Endometrium |
| 597 | BCL2A1 | -2.020323451 | 4.97E-20 | Endometrium |
| 5104 | SERPINA5 | -2.017390313 | 4.06E-06 | Endometrium |
| 202309 | GAPT | -2.014053668 | 1.03E-18 | Endometrium |
| 23533 | PIK3R5 | -2.013830685 | 7.22E-28 | Endometrium |
| 440712 | RHEX | -2.013230278 | 2.84E-06 | Endometrium |
| 115361 | GBP4 | -2.012780527 | 2.12E-25 | Endometrium |
| 9452 | ITM2A | -2.010148314 | 1.65E-16 | Endometrium |
| 951 | CD37 | -2.009250749 | 5.44E-30 | Endometrium |
| 11151 | CORO1A | -2.0041673 | 1.97E-30 | Endometrium |
| 3580 | None | -2.004059561 | 5.90E-10 | Endometrium |
| 1043 | CD52 | -2.003573764 | 2.07E-20 | Endometrium |
| 11262 | SP140 | -2.002366577 | 7.43E-21 | Endometrium |
| 5341 | PLEK | -1.999978745 | 2.45E-23 | Endometrium |
| 131578 | LRRC15 | -1.997266249 | 5.35E-07 | Endometrium |
| 8876 | VNN1 | -1.997012202 | 2.00E-06 | Endometrium |
| 11095 | ADAMTS8 | -1.994593947 | 1.46E-06 | Endometrium |
| 23495 | TNFRSF13B | -1.993961513 | 2.24E-13 | Endometrium |
| 113730 | KLHDC7B | -1.99296293 | 1.64E-11 | Endometrium |
| 26253 | CLEC4E | -1.983908541 | 6.61E-12 | Endometrium |
| 27181 | SIGLEC8 | -1.982045592 | 3.59E-13 | Endometrium |
| 2357 | FPR1 | -1.981638157 | 2.63E-16 | Endometrium |
| 79974 | CPED1 | -1.980942629 | 2.14E-18 | Endometrium |
| 1441 | CSF3R | -1.980412852 | 2.96E-16 | Endometrium |
| 89857 | KLHL6 | -1.980296041 | 5.11E-16 | Endometrium |
| 56833 | SLAMF8 | -1.979691948 | 1.17E-24 | Endometrium |
| 53831 | GPR84 | -1.978962291 | 7.43E-21 | Endometrium |
| 3824 | KLRD1 | -1.977424703 | 1.01E-14 | Endometrium |
| 9242 | MSC | -1.97625675 | 1.25E-17 | Endometrium |
| 133418 | EMB | -1.974490626 | 2.45E-16 | Endometrium |
| 3805 | KIR2DL4 | -1.973971398 | 8.70E-14 | Endometrium |
| 10673 | TNFSF13B | -1.97233181 | 4.12E-22 | Endometrium |
| 1734 | DIO2 | -1.968259004 | 2.35E-11 | Endometrium |
| 3134 | HLA-F | -1.965888587 | 6.75E-19 | Endometrium |
| 911 | CD1C | -1.962874493 | 3.90E-13 | Endometrium |
| 64005 | MYO1G | -1.962671924 | 2.69E-34 | Endometrium |
| 27033 | ZBTB32 | -1.960591439 | 7.61E-20 | Endometrium |
| 909 | CD1A | -1.95962961 | 2.94E-10 | Endometrium |
| 654817 | None | -1.959382164 | 6.11E-21 | Endometrium |
| 64407 | RGS18 | -1.958913141 | 1.23E-21 | Endometrium |
| 9535 | GMFG | -1.956041462 | 5.91E-32 | Endometrium |
| 6374 | CXCL5 | -1.9540877 | 7.88E-07 | Endometrium |
| 84868 | HAVCR2 | -1.95346507 | 6.36E-27 | Endometrium |
| 22915 | MMRN1 | -1.952728261 | 2.82E-12 | Endometrium |
| 942 | CD86 | -1.952272582 | 2.46E-24 | Endometrium |
| 2015 | ADGRE1 | -1.950297005 | 7.96E-18 | Endometrium |
| 1536 | CYBB | -1.948113437 | 1.85E-19 | Endometrium |
| 56667 | MUC13 | -1.944733946 | 7.33E-05 | Endometrium |
| 8771 | TNFRSF6B | -1.943004076 | 1.87E-16 | Endometrium |
| 64919 | BCL11B | -1.942123498 | 4.43E-15 | Endometrium |
| 2167 | FABP4 | -1.938863025 | 3.40E-06 | Endometrium |
| 51284 | TLR7 | -1.938852461 | 4.20E-18 | Endometrium |
| 131450 | CD200R1 | -1.938485418 | 1.10E-21 | Endometrium |
| 969 | CD69 | -1.935745032 | 2.34E-14 | Endometrium |
| 79931 | TNIP3 | -1.934724242 | 1.96E-13 | Endometrium |
| 654816 | None | -1.933969466 | 2.43E-20 | Endometrium |
| 199 | AIF1 | -1.932792399 | 4.28E-26 | Endometrium |
| 26051 | PPP1R16B | -1.932003538 | 1.88E-22 | Endometrium |
| 11006 | LILRB4 | -1.929778668 | 2.19E-17 | Endometrium |
| 1804 | DPP6 | -1.926694174 | 4.89E-07 | Endometrium |
| 7454 | WAS | -1.926420424 | 1.07E-33 | Endometrium |
| 51365 | PLA1A | -1.926115485 | 1.90E-10 | Endometrium |
| 80342 | TRAF3IP3 | -1.924177924 | 2.63E-27 | Endometrium |
| 6364 | CCL20 | -1.921795479 | 4.88E-06 | Endometrium |
| 338773 | TMEM119 | -1.921042223 | 3.83E-18 | Endometrium |
| 653361 | NCF1 | -1.91768688 | 6.23E-20 | Endometrium |
| 3122 | HLA-DRA | -1.913782031 | 2.13E-19 | Endometrium |
| 79626 | TNFAIP8L2 | -1.913391021 | 1.88E-28 | Endometrium |
| 4318 | MMP9 | -1.912870023 | 1.04E-11 | Endometrium |
| 404552 | SCGB1D4 | -1.91096465 | 0.000172081 | Endometrium |
| 23643 | LY96 | -1.910053473 | 2.71E-20 | Endometrium |
| 139716 | GAB3 | -1.90855828 | 8.24E-29 | Endometrium |
| 2219 | FCN1 | -1.908381139 | 2.13E-13 | Endometrium |
| 170575 | GIMAP1 | -1.908203994 | 2.44E-27 | Endometrium |
| 722 | C4BPA | -1.906624991 | 0.000229699 | Endometrium |
| 4033 | LRMP | -1.904743178 | 1.18E-19 | Endometrium |
| 8832 | CD84 | -1.904414253 | 8.14E-20 | Endometrium |
| 116986 | AGAP2 | -1.903343868 | 4.62E-29 | Endometrium |
| 58475 | MS4A7 | -1.903298924 | 6.30E-21 | Endometrium |
| 6688 | SPI1 | -1.902053816 | 2.12E-29 | Endometrium |
| 10666 | CD226 | -1.901436741 | 7.39E-21 | Endometrium |
| 6291 | SAA4 | -1.899516181 | 2.46E-07 | Endometrium |
| 606724 | None | -1.899006022 | 2.00E-25 | Endometrium |
| 920 | CD4 | -1.898656587 | 6.46E-30 | Endometrium |
| 366 | AQP9 | -1.898083574 | 1.34E-12 | Endometrium |
| 1089 | CEACAM4 | -1.897121707 | 7.91E-21 | Endometrium |
| 129293 | TRABD2A | -1.895272994 | 2.99E-09 | Endometrium |
| 9450 | LY86 | -1.894469908 | 3.45E-20 | Endometrium |
| 23460 | ABCA6 | -1.89336053 | 4.81E-18 | Endometrium |
| 1436 | CSF1R | -1.893086414 | 4.92E-25 | Endometrium |
| 3903 | LAIR1 | -1.89218787 | 7.75E-28 | Endometrium |
| 1634 | DCN | -1.891996456 | 1.86E-13 | Endometrium |
| 719 | C3AR1 | -1.891000997 | 2.09E-25 | Endometrium |
| 5452 | POU2F2 | -1.889466456 | 3.33E-22 | Endometrium |
| 51338 | MS4A4A | -1.889315492 | 5.22E-21 | Endometrium |
| 100132417 | None | -1.889002372 | 3.95E-23 | Endometrium |
| 9976 | CLEC2B | -1.888975096 | 1.19E-31 | Endometrium |
| 3689 | ITGB2 | -1.888175773 | 1.89E-24 | Endometrium |
| 2268 | FGR | -1.886818022 | 6.25E-27 | Endometrium |
| 6489 | ST8SIA1 | -1.885173177 | 1.59E-14 | Endometrium |
| 54331 | GNG2 | -1.884864853 | 1.15E-27 | Endometrium |
| 5266 | PI3 | -1.884692438 | 6.25E-05 | Endometrium |
| 92211 | CDHR1 | -1.884378977 | 1.39E-06 | Endometrium |
| 80111 | SLCO2A1 | -1.883872346 | 1.93E-10 | Endometrium |
| 8115 | None | -1.883149577 | 2.48E-12 | Endometrium |
| 2877 | GPX2 | -1.881758141 | 0.000619517 | Endometrium |
| 10235 | RASGRP2 | -1.878186257 | 1.03E-23 | Endometrium |
| 92241 | RCSD1 | -1.876411477 | 4.51E-29 | Endometrium |
| 2918 | GRM8 | -1.875544637 | 1.85E-09 | Endometrium |
| 4321 | MMP12 | -1.874954924 | 2.67E-08 | Endometrium |
| 147111 | NOTUM | -1.874260397 | 0.000914521 | Endometrium |
| 9235 | IL32 | -1.873228164 | 1.73E-16 | Endometrium |
| 645784 | None | -1.872974976 | 4.47E-11 | Endometrium |
| 3620 | IDO1 | -1.871338196 | 1.70E-11 | Endometrium |
| 6404 | SELPLG | -1.870377851 | 8.71E-29 | Endometrium |
| 100188949 | None | -1.869056625 | 8.35E-19 | Endometrium |
| 7472 | WNT2 | -1.868585006 | 5.37E-12 | Endometrium |
| 3290 | HSD11B1 | -1.867162272 | 2.35E-13 | Endometrium |
| 972 | CD74 | -1.86089738 | 7.28E-19 | Endometrium |
| 284110 | GSDMA | -1.860229651 | 4.41E-12 | Endometrium |
| 160365 | CLECL1 | -1.857796721 | 3.58E-21 | Endometrium |
| 10870 | HCST | -1.856851734 | 2.80E-23 | Endometrium |
| 6347 | CCL2 | -1.856830347 | 6.70E-18 | Endometrium |
| 122618 | PLD4 | -1.856767513 | 8.65E-13 | Endometrium |
| 353514 | LILRA5 | -1.85668063 | 1.38E-17 | Endometrium |
| 7805 | LAPTM5 | -1.855323622 | 1.89E-30 | Endometrium |
| 6285 | S100B | -1.855061819 | 2.17E-12 | Endometrium |
| 714 | C1QC | -1.854460882 | 9.26E-21 | Endometrium |
| 10148 | EBI3 | -1.854006964 | 6.38E-15 | Endometrium |
| 933 | CD22 | -1.853758773 | 9.24E-08 | Endometrium |
| 945 | CD33 | -1.853271592 | 1.80E-23 | Endometrium |
| 2568 | GABRP | -1.850871109 | 1.47E-05 | Endometrium |
| 4060 | LUM | -1.847307916 | 2.60E-14 | Endometrium |
| 284759 | SIRPB2 | -1.846606227 | 3.53E-19 | Endometrium |
| 203100 | HTRA4 | -1.844889196 | 3.28E-13 | Endometrium |
| 3687 | ITGAX | -1.844603527 | 1.70E-21 | Endometrium |
| 147138 | TMC8 | -1.843897386 | 3.35E-28 | Endometrium |
| 970 | None | -1.843834218 | 5.81E-15 | Endometrium |
| 860 | RUNX2 | -1.843566329 | 7.51E-14 | Endometrium |
| 727897 | MUC5B | -1.84356173 | 0.001890366 | Endometrium |
| 2210 | FCGR1B | -1.842631218 | 9.52E-23 | Endometrium |
| 128602 | C20orf85 | -1.840519901 | 0.001214741 | Endometrium |
| 4046 | LSP1 | -1.839792178 | 1.20E-25 | Endometrium |
| 1776 | DNASE1L3 | -1.839321872 | 5.53E-17 | Endometrium |
| 64174 | DPEP2 | -1.835465234 | 3.96E-18 | Endometrium |
| 219527 | LRRC55 | -1.832919474 | 5.73E-09 | Endometrium |
| 51703 | ACSL5 | -1.832129003 | 5.40E-10 | Endometrium |
| 9023 | CH25H | -1.83096163 | 1.39E-12 | Endometrium |
| 474344 | GIMAP6 | -1.828682661 | 3.47E-30 | Endometrium |
| 241 | ALOX5AP | -1.827661833 | 1.05E-18 | Endometrium |
| 83706 | FERMT3 | -1.827211522 | 1.11E-31 | Endometrium |
| 2920 | CXCL2 | -1.826536124 | 1.19E-08 | Endometrium |
| 79804 | None | -1.825461198 | 3.77E-09 | Endometrium |
| 79949 | PLEKHS1 | -1.825029464 | 2.47E-05 | Endometrium |
| 4064 | CD180 | -1.824623203 | 2.06E-19 | Endometrium |
| 8600 | TNFSF11 | -1.822574467 | 4.87E-08 | Endometrium |
| 56978 | PRDM8 | -1.819735406 | 1.40E-14 | Endometrium |
| 137835 | TMEM71 | -1.819410503 | 1.60E-20 | Endometrium |
| 913 | CD1E | -1.818520182 | 2.26E-11 | Endometrium |
| 114132 | SIGLEC11 | -1.816782807 | 1.29E-16 | Endometrium |
| 2995 | GYPC | -1.816377385 | 1.09E-25 | Endometrium |
| 9214 | FCMR | -1.814674949 | 4.90E-25 | Endometrium |
| 225 | ABCD2 | -1.813261375 | 5.70E-16 | Endometrium |
| 4837 | NNMT | -1.812974467 | 9.04E-13 | Endometrium |
| 639 | PRDM1 | -1.812309289 | 1.58E-26 | Endometrium |
| 8676 | STX11 | -1.812115249 | 6.99E-27 | Endometrium |
| 943 | TNFRSF8 | -1.811819861 | 4.09E-19 | Endometrium |
| 7456 | WIPF1 | -1.811369884 | 3.36E-35 | Endometrium |
| 3635 | INPP5D | -1.80959853 | 3.43E-26 | Endometrium |
| 79574 | EPS8L3 | -1.806907014 | 1.89E-05 | Endometrium |
| 3766 | KCNJ10 | -1.804709628 | 4.68E-13 | Endometrium |
| 9075 | CLDN2 | -1.803610675 | 8.22E-05 | Endometrium |
| 401498 | TMEM215 | -1.801796441 | 3.48E-09 | Endometrium |
| 8778 | SIGLEC5 | -1.801617741 | 8.51E-18 | Endometrium |
| 100049587 | SIGLEC14 | -1.801062623 | 3.45E-17 | Endometrium |
| 6375 | XCL1 | -1.79928431 | 1.53E-09 | Endometrium |
| 6349 | CCL3L1 | -1.797244936 | 1.18E-14 | Endometrium |
| 57864 | SLC46A2 | -1.796511885 | 5.92E-07 | Endometrium |
| 941 | CD80 | -1.795373096 | 1.16E-17 | Endometrium |
| 4246 | SCGB2A1 | -1.794536551 | 0.000384946 | Endometrium |
| 221472 | FGD2 | -1.792893387 | 9.18E-22 | Endometrium |
| 57699 | CPNE5 | -1.792593901 | 3.32E-18 | Endometrium |
| 4261 | CIITA | -1.792498665 | 4.59E-17 | Endometrium |
| 7293 | TNFRSF4 | -1.79226453 | 2.04E-23 | Endometrium |
| 3549 | IHH | -1.790451328 | 0.002336972 | Endometrium |
| 716 | C1S | -1.790120283 | 9.28E-23 | Endometrium |
| 3936 | LCP1 | -1.790018343 | 6.73E-19 | Endometrium |
| 11309 | SLCO2B1 | -1.786129018 | 3.46E-21 | Endometrium |
| 160364 | CLEC12A | -1.783292695 | 6.78E-14 | Endometrium |
| 2209 | FCGR1A | -1.781829957 | 1.73E-22 | Endometrium |
| 2207 | FCER1G | -1.781325984 | 9.06E-25 | Endometrium |
| 5880 | RAC2 | -1.77912939 | 5.34E-22 | Endometrium |
| 10350 | ABCA9 | -1.776179557 | 2.44E-12 | Endometrium |
| 26228 | STAP1 | -1.775918275 | 9.18E-16 | Endometrium |
| 7133 | TNFRSF1B | -1.775554119 | 6.36E-29 | Endometrium |
| 257106 | ARHGAP30 | -1.774026739 | 1.24E-32 | Endometrium |
| 155038 | GIMAP8 | -1.773781166 | 3.01E-30 | Endometrium |
| 2215 | FCGR3B | -1.773470982 | 2.22E-19 | Endometrium |
| 3078 | CFHR1 | -1.772803888 | 4.99E-12 | Endometrium |
| 284021 | MILR1 | -1.771980834 | 5.85E-16 | Endometrium |
| 1438 | CSF2RA | -1.771441525 | 2.42E-18 | Endometrium |
| 10351 | ABCA8 | -1.770057312 | 7.02E-12 | Endometrium |
| 2213 | FCGR2B | -1.76919418 | 1.11E-16 | Endometrium |
| 84689 | None | -1.768799727 | 3.37E-17 | Endometrium |
| 7475 | WNT6 | -1.768419881 | 2.04E-06 | Endometrium |
| 400759 | None | -1.767955286 | 5.08E-15 | Endometrium |
| 2793 | GNGT2 | -1.767639427 | 2.56E-21 | Endometrium |
| 55303 | GIMAP4 | -1.767548495 | 2.25E-24 | Endometrium |
| 6036 | RNASE2 | -1.767019346 | 2.80E-16 | Endometrium |
| 389118 | CDHR4 | -1.766693555 | 0.000420519 | Endometrium |
| 60489 | APOBEC3G | -1.766059378 | 4.20E-20 | Endometrium |
| 118430 | MUCL1 | -1.76519914 | 0.00013474 | Endometrium |
| 24141 | LAMP5 | -1.764184798 | 4.13E-05 | Endometrium |
| 221091 | LRRN4CL | -1.76294287 | 1.81E-14 | Endometrium |
| 152330 | CNTN4 | -1.76219647 | 8.77E-11 | Endometrium |
| 50846 | DHH | -1.761044577 | 8.06E-17 | Endometrium |
| 3823 | KLRC3 | -1.759591155 | 1.18E-16 | Endometrium |
| 116071 | BATF2 | -1.757853023 | 1.74E-14 | Endometrium |
| 441168 | CALHM6 | -1.757623976 | 6.58E-15 | Endometrium |
| 57863 | CADM3 | -1.755607891 | 3.82E-07 | Endometrium |
| 63940 | GPSM3 | -1.755278332 | 4.76E-30 | Endometrium |
| 10346 | TRIM22 | -1.755175439 | 1.31E-18 | Endometrium |
| 126259 | TMIGD2 | -1.754739723 | 5.46E-17 | Endometrium |
| 146850 | PIK3R6 | -1.754338161 | 2.21E-25 | Endometrium |
| 10990 | LILRB5 | -1.75316154 | 5.68E-18 | Endometrium |
| 84636 | GPR174 | -1.752403288 | 7.09E-16 | Endometrium |
| 50943 | FOXP3 | -1.751917165 | 2.27E-25 | Endometrium |
| 6947 | TCN1 | -1.751913027 | 2.52E-05 | Endometrium |
| 4311 | MME | -1.751731505 | 1.40E-08 | Endometrium |
| 54829 | ASPN | -1.751418745 | 8.47E-09 | Endometrium |
| 9464 | HAND2 | -1.751243036 | 1.67E-10 | Endometrium |
| 7148 | TNXB | -1.750382918 | 3.04E-10 | Endometrium |
| 25900 | IFFO1 | -1.750086808 | 2.76E-30 | Endometrium |
| 330 | BIRC3 | -1.749586228 | 1.88E-11 | Endometrium |
| 3574 | IL7 | -1.746995982 | 3.03E-13 | Endometrium |
| 115019 | SLC26A9 | -1.745414866 | 5.71E-08 | Endometrium |
| 92304 | SCGB3A1 | -1.74365658 | 0.000727455 | Endometrium |
| 3569 | IL6 | -1.743173531 | 4.34E-11 | Endometrium |
| 9103 | FCGR2A | -1.742496046 | 5.23E-22 | Endometrium |
| 5698 | PSMB9 | -1.74215918 | 2.89E-17 | Endometrium |
| 4145 | MATK | -1.741048669 | 2.97E-17 | Endometrium |
| 5008 | OSM | -1.74018233 | 5.13E-16 | Endometrium |
| 8942 | KYNU | -1.736586494 | 1.74E-09 | Endometrium |
| 51513 | ETV7 | -1.736396056 | 1.87E-14 | Endometrium |
| 629 | CFB | -1.736281545 | 2.84E-07 | Endometrium |
| 22943 | DKK1 | -1.736159396 | 0.000848346 | Endometrium |
| 51225 | ABI3 | -1.735722291 | 6.20E-33 | Endometrium |
| 2857 | GPR34 | -1.731821899 | 3.53E-17 | Endometrium |
| 3116 | None | -1.731160495 | 1.47E-14 | Endometrium |
| 55619 | DOCK10 | -1.730215856 | 2.10E-23 | Endometrium |
| 3601 | IL15RA | -1.728351711 | 8.90E-23 | Endometrium |
| 3553 | IL1B | -1.727097127 | 3.48E-15 | Endometrium |
| 27036 | SIGLEC7 | -1.726970113 | 5.55E-18 | Endometrium |
| 4606 | MYBPC2 | -1.725875543 | 6.59E-07 | Endometrium |
| 9622 | KLK4 | -1.725685713 | 5.50E-05 | Endometrium |
| 85453 | TSPYL5 | -1.724672271 | 2.02E-10 | Endometrium |
| 84433 | CARD11 | -1.724024334 | 9.82E-11 | Endometrium |
| 23544 | SEZ6L | -1.723328452 | 6.35E-05 | Endometrium |
| 54739 | XAF1 | -1.722856395 | 4.30E-12 | Endometrium |
| 8875 | VNN2 | -1.721600402 | 9.79E-10 | Endometrium |
| 866 | SERPINA6 | -1.720110351 | 0.000300704 | Endometrium |
| 126364 | LRRC25 | -1.719612707 | 2.72E-23 | Endometrium |
| 114548 | NLRP3 | -1.719464465 | 1.22E-25 | Endometrium |
| 11197 | WIF1 | -1.715968032 | 0.001918132 | Endometrium |
| 84106 | PRAM1 | -1.715748404 | 2.54E-20 | Endometrium |
| 285195 | SLC9A9 | -1.715518748 | 2.89E-22 | Endometrium |
| 3053 | SERPIND1 | -1.713990477 | 4.18E-08 | Endometrium |
| 83593 | RASSF5 | -1.713934415 | 2.70E-29 | Endometrium |
| 89858 | SIGLEC12 | -1.713307611 | 2.40E-09 | Endometrium |
| 1806 | DPYD | -1.711898754 | 9.80E-17 | Endometrium |
| 5967 | REG1A | -1.711476366 | 4.22E-06 | Endometrium |
| 286336 | FAM78A | -1.710832916 | 3.90E-29 | Endometrium |
| 114769 | CARD16 | -1.710810361 | 3.45E-22 | Endometrium |
| 285180 | RUFY4 | -1.710597609 | 1.43E-08 | Endometrium |
| 7434 | VIPR2 | -1.709712948 | 1.23E-10 | Endometrium |
| 23547 | LILRA4 | -1.708288241 | 3.83E-15 | Endometrium |
| 718 | C3 | -1.706960417 | 1.00E-06 | Endometrium |
| 125111 | GJD3 | -1.706893907 | 8.50E-19 | Endometrium |
| 834 | CASP1 | -1.705485868 | 4.28E-20 | Endometrium |
| 5732 | PTGER2 | -1.704982624 | 7.44E-12 | Endometrium |
| 146206 | CARMIL2 | -1.704727581 | 6.78E-13 | Endometrium |
| 50856 | CLEC4A | -1.702153804 | 1.41E-23 | Endometrium |
| 2313 | FLI1 | -1.70033866 | 3.24E-31 | Endometrium |
| 3718 | JAK3 | -1.696386486 | 8.17E-23 | Endometrium |
| 5552 | SRGN | -1.696182542 | 2.72E-23 | Endometrium |
| 84251 | SGIP1 | -1.695925327 | 9.88E-14 | Endometrium |
| 64581 | CLEC7A | -1.695642316 | 1.29E-16 | Endometrium |
| 146177 | VWA3A | -1.695303958 | 0.000113929 | Endometrium |
| 2191 | FAP | -1.694760344 | 3.20E-14 | Endometrium |
| 929 | CD14 | -1.694697972 | 3.83E-22 | Endometrium |
| 7099 | TLR4 | -1.694422654 | 5.51E-13 | Endometrium |
| 124599 | CD300LB | -1.694097749 | 6.45E-17 | Endometrium |
| 114905 | C1QTNF7 | -1.693364793 | 9.64E-11 | Endometrium |
| 127018 | LYPLAL1 | -1.692596205 | 7.54E-09 | Endometrium |
| 2634 | GBP2 | -1.69139533 | 1.68E-21 | Endometrium |
| 10647 | SCGB1D2 | -1.69014655 | 0.000627538 | Endometrium |
| 9839 | ZEB2 | -1.68391779 | 1.36E-27 | Endometrium |
| 342615 | None | -1.683222286 | 1.94E-18 | Endometrium |
| 3101 | HK3 | -1.682054971 | 2.57E-17 | Endometrium |
| 9332 | CD163 | -1.68159445 | 1.60E-14 | Endometrium |
| 11025 | LILRB3 | -1.679774646 | 2.80E-22 | Endometrium |
| 113457 | TUBA3D | -1.678663879 | 1.83E-05 | Endometrium |
| 2359 | FPR3 | -1.678134433 | 4.47E-16 | Endometrium |
| 1800 | DPEP1 | -1.677352047 | 0.000100883 | Endometrium |
| 9934 | P2RY14 | -1.676247563 | 3.15E-12 | Endometrium |
| 9938 | ARHGAP25 | -1.670400033 | 2.34E-29 | Endometrium |
| 5142 | PDE4B | -1.67032483 | 2.86E-24 | Endometrium |
| 9034 | CCRL2 | -1.670213872 | 3.40E-20 | Endometrium |
| 440836 | ODF3B | -1.66816323 | 2.88E-10 | Endometrium |
| 147495 | APCDD1 | -1.667006121 | 5.63E-09 | Endometrium |
| 89870 | TRIM15 | -1.665887861 | 5.04E-06 | Endometrium |
| 5729 | PTGDR | -1.665236219 | 8.15E-15 | Endometrium |
| 352961 | None | -1.66487123 | 1.14E-13 | Endometrium |
| 57644 | MYH7B | -1.664725718 | 1.17E-05 | Endometrium |
| 6999 | TDO2 | -1.663785831 | 1.90E-10 | Endometrium |
| 115727 | RASGRP4 | -1.663197551 | 6.92E-19 | Endometrium |
| 85352 | SHISAL1 | -1.662263478 | 6.21E-09 | Endometrium |
| 7305 | TYROBP | -1.661993936 | 1.45E-21 | Endometrium |
| 2322 | FLT3 | -1.66197628 | 1.28E-13 | Endometrium |
| 5156 | PDGFRA | -1.660463836 | 2.30E-09 | Endometrium |
| 717 | C2 | -1.659450141 | 1.22E-13 | Endometrium |
| 3075 | CFH | -1.658329546 | 1.87E-11 | Endometrium |
| 57616 | TSHZ3 | -1.656586673 | 5.53E-19 | Endometrium |
| 3581 | IL9R | -1.655509375 | 1.48E-15 | Endometrium |
| 6348 | CCL3 | -1.653626777 | 2.11E-16 | Endometrium |
| 84628 | NTNG2 | -1.652758113 | 2.25E-13 | Endometrium |
| 112714 | TUBA3E | -1.652701469 | 2.92E-05 | Endometrium |
| 165186 | TOGARAM2 | -1.651900368 | 3.88E-07 | Endometrium |
| 8542 | APOL1 | -1.650884323 | 7.20E-11 | Endometrium |
| 27123 | DKK2 | -1.650159082 | 2.80E-08 | Endometrium |
| 26157 | GIMAP2 | -1.649829658 | 5.05E-17 | Endometrium |
| 57705 | WDFY4 | -1.649093409 | 1.84E-15 | Endometrium |
| 27180 | SIGLEC9 | -1.648315432 | 2.38E-19 | Endometrium |
| 30835 | CD209 | -1.64782836 | 6.29E-16 | Endometrium |
| 2214 | FCGR3A | -1.647741254 | 1.49E-18 | Endometrium |
| 7223 | TRPC4 | -1.646892868 | 2.12E-13 | Endometrium |
| 720 | C4A | -1.646619641 | 9.91E-14 | Endometrium |
| 1513 | CTSK | -1.646053445 | 6.13E-20 | Endometrium |
| 4487 | MSX1 | -1.645206826 | 0.000317037 | Endometrium |
| 6775 | STAT4 | -1.644024742 | 2.64E-20 | Endometrium |
| 3624 | INHBA | -1.643632486 | 4.13E-10 | Endometrium |
| 10077 | TSPAN32 | -1.64346273 | 1.30E-10 | Endometrium |
| 5920 | RARRES3 | -1.640741632 | 9.26E-12 | Endometrium |
| 1414 | CRYBB1 | -1.639103115 | 3.17E-07 | Endometrium |
| 5267 | SERPINA4 | -1.637668363 | 0.000166583 | Endometrium |
| 440603 | BCL2L15 | -1.636068566 | 1.56E-06 | Endometrium |
| 51316 | PLAC8 | -1.635764413 | 1.12E-09 | Endometrium |
| 3055 | HCK | -1.634649197 | 1.96E-16 | Endometrium |
| 340152 | ZC3H12D | -1.632975657 | 2.91E-18 | Endometrium |
| 79987 | SVEP1 | -1.632590848 | 1.11E-13 | Endometrium |
| 55911 | APOBR | -1.632404357 | 7.02E-25 | Endometrium |
| 2669 | GEM | -1.632385529 | 2.17E-17 | Endometrium |
| 3934 | LCN2 | -1.631562264 | 5.19E-05 | Endometrium |
| 646300 | None | -1.630889169 | 4.60E-08 | Endometrium |
| 4978 | OPCML | -1.63085926 | 1.44E-10 | Endometrium |
| 710 | SERPING1 | -1.629888204 | 7.07E-20 | Endometrium |
| 2919 | CXCL1 | -1.628525 | 1.19E-05 | Endometrium |
| 2208 | FCER2 | -1.627426749 | 2.46E-12 | Endometrium |
| 219285 | SAMD9L | -1.626154487 | 3.49E-15 | Endometrium |
| 388325 | SCIMP | -1.624678634 | 2.84E-17 | Endometrium |
| 6361 | CCL17 | -1.623883518 | 1.17E-10 | Endometrium |
| 3106 | HLA-B | -1.623111623 | 6.17E-16 | Endometrium |
| 6863 | TAC1 | -1.62156157 | 5.93E-05 | Endometrium |
| 10154 | PLXNC1 | -1.619536225 | 4.41E-12 | Endometrium |
| 92745 | SLC38A5 | -1.616750651 | 4.38E-08 | Endometrium |
| 84166 | NLRC5 | -1.614832882 | 1.32E-19 | Endometrium |
| 11314 | CD300A | -1.61315364 | 5.66E-23 | Endometrium |
| 397 | ARHGDIB | -1.612884957 | 7.36E-28 | Endometrium |
| 51705 | EMCN | -1.611998137 | 2.31E-12 | Endometrium |
| 84935 | MEDAG | -1.609004185 | 3.40E-12 | Endometrium |
| 3579 | CXCR2 | -1.604436943 | 9.18E-09 | Endometrium |
| 128346 | C1orf162 | -1.602304961 | 2.17E-21 | Endometrium |
| 126410 | CYP4F22 | -1.602085908 | 3.02E-12 | Endometrium |
| 57091 | CASS4 | -1.601818072 | 1.03E-14 | Endometrium |
| 4973 | OLR1 | -1.600698169 | 3.01E-10 | Endometrium |
| 1842 | ECM2 | -1.600127722 | 2.91E-13 | Endometrium |
| 7123 | CLEC3B | -1.600071981 | 2.53E-11 | Endometrium |
| 120892 | LRRK2 | -1.598961085 | 7.43E-11 | Endometrium |
| 728215 | FAM155A | -1.598880612 | 1.10E-05 | Endometrium |
| 5243 | ABCB1 | -1.598854057 | 2.81E-13 | Endometrium |
| 51393 | TRPV2 | -1.597424945 | 3.71E-31 | Endometrium |
| 2212 | FCGR2A | -1.593728868 | 3.17E-25 | Endometrium |
| 11248 | NXPH3 | -1.591927752 | 6.80E-09 | Endometrium |
| 29802 | VPREB3 | -1.591219196 | 9.08E-09 | Endometrium |
| 9956 | HS3ST2 | -1.591052331 | 2.13E-11 | Endometrium |
| 9459 | ARHGEF6 | -1.590494018 | 1.35E-29 | Endometrium |
| 56923 | NMUR2 | -1.588813385 | 2.64E-07 | Endometrium |
| 8564 | KMO | -1.587011232 | 1.91E-08 | Endometrium |
| 6387 | CXCL12 | -1.58673644 | 5.65E-12 | Endometrium |
| 10335 | MRVI1 | -1.585070993 | 1.42E-17 | Endometrium |
| 10158 | PDZK1IP1 | -1.584530779 | 6.84E-05 | Endometrium |
| 197135 | PATL2 | -1.582685247 | 2.79E-12 | Endometrium |
| 3681 | ITGAD | -1.582443845 | 6.69E-12 | Endometrium |
| 11074 | TRIM31 | -1.582064129 | 0.000435695 | Endometrium |
| 401551 | WDR38 | -1.57855851 | 0.001067053 | Endometrium |
| 3394 | IRF8 | -1.578557752 | 7.69E-14 | Endometrium |
| 4094 | MAF | -1.578249789 | 6.96E-21 | Endometrium |
| 27287 | VENTX | -1.577810612 | 3.07E-17 | Endometrium |
| 3090 | HIC1 | -1.577681949 | 1.14E-19 | Endometrium |
| 79895 | ATP8B4 | -1.576015403 | 3.59E-18 | Endometrium |
| 2350 | FOLR2 | -1.57540579 | 3.76E-13 | Endometrium |
| 1893 | ECM1 | -1.57436378 | 3.96E-14 | Endometrium |
| 257194 | NEGR1 | -1.572845134 | 1.04E-07 | Endometrium |
| 3428 | IFI16 | -1.572835999 | 1.48E-14 | Endometrium |
| 4914 | NTRK1 | -1.572757213 | 1.99E-10 | Endometrium |
| 1731 | Sep-01 | -1.572566219 | 6.86E-20 | Endometrium |
| 64386 | MMP25 | -1.570985955 | 1.66E-15 | Endometrium |
| 117581 | TWIST2 | -1.56969982 | 5.58E-07 | Endometrium |
| 7462 | LAT2 | -1.569513417 | 6.33E-29 | Endometrium |
| 1118 | CHIT1 | -1.569279196 | 4.09E-05 | Endometrium |
| 4629 | MYH11 | -1.569244871 | 1.67E-07 | Endometrium |
| 80341 | BPIFB2 | -1.569096379 | 0.000810121 | Endometrium |
| 2625 | GATA3 | -1.567663193 | 6.51E-09 | Endometrium |
| 1036 | CDO1 | -1.565797467 | 1.79E-06 | Endometrium |
| 387763 | C11orf96 | -1.563323965 | 1.67E-14 | Endometrium |
| 54847 | SIDT1 | -1.561916188 | 2.96E-07 | Endometrium |
| 222487 | ADGRG3 | -1.561496115 | 4.12E-09 | Endometrium |
| 3779 | KCNMB1 | -1.561228415 | 1.56E-10 | Endometrium |
| 8174 | MADCAM1 | -1.560068119 | 3.53E-11 | Endometrium |
| 22885 | ABLIM3 | -1.559966361 | 1.21E-14 | Endometrium |
| 164668 | APOBEC3H | -1.559864406 | 8.05E-13 | Endometrium |
| 25907 | TMEM158 | -1.559470587 | 3.78E-12 | Endometrium |
| 5330 | PLCB2 | -1.558696113 | 4.22E-17 | Endometrium |
| 401124 | DTHD1 | -1.557584868 | 3.24E-06 | Endometrium |
| 1475 | CSTA | -1.556715087 | 1.81E-09 | Endometrium |
| 140862 | ISM1 | -1.555433847 | 3.23E-09 | Endometrium |
| 79413 | ZBED2 | -1.555391286 | 6.32E-08 | Endometrium |
| 50515 | CHST11 | -1.555287491 | 2.24E-13 | Endometrium |
| 80740 | LY6G6C | -1.554303931 | 0.0004278 | Endometrium |
| 79843 | FAM124B | -1.553736907 | 4.42E-12 | Endometrium |
| 2294 | FOXF1 | -1.552948296 | 4.91E-17 | Endometrium |
| 11010 | GLIPR1 | -1.552141977 | 2.66E-27 | Endometrium |
| 29126 | CD274 | -1.551652196 | 3.17E-14 | Endometrium |
| 1230 | CCR1 | -1.551269489 | 6.51E-19 | Endometrium |
| 387695 | C10orf99 | -1.550889475 | 0.000347079 | Endometrium |
| 10417 | SPON2 | -1.550749981 | 1.34E-16 | Endometrium |
| 9750 | RIPOR2 | -1.550539436 | 1.34E-12 | Endometrium |
| 8418 | None | -1.548696843 | 3.27E-10 | Endometrium |
| 3386 | ICAM4 | -1.546510024 | 3.27E-11 | Endometrium |
| 6274 | S100A3 | -1.545089888 | 8.22E-07 | Endometrium |
| 374403 | TBC1D10C | -1.543692743 | 7.23E-13 | Endometrium |
| 94240 | EPSTI1 | -1.542766036 | 3.35E-12 | Endometrium |
| 81615 | TMEM163 | -1.542526519 | 1.73E-08 | Endometrium |
| 5199 | CFP | -1.542155992 | 1.33E-15 | Endometrium |
| 1589 | CYP21A2 | -1.541119655 | 1.45E-09 | Endometrium |
| 5176 | SERPINF1 | -1.540930569 | 3.66E-12 | Endometrium |
| 80830 | APOL6 | -1.540908946 | 1.74E-18 | Endometrium |
| 7292 | TNFSF4 | -1.540429496 | 9.48E-15 | Endometrium |
| 10268 | RAMP3 | -1.539520243 | 7.77E-16 | Endometrium |
| 728264 | None | -1.538740863 | 2.03E-12 | Endometrium |
| 84336 | TMEM101 | -1.536504051 | 1.13E-05 | Endometrium |
| 6943 | TCF21 | -1.53631944 | 1.20E-07 | Endometrium |
| 57569 | ARHGAP20 | -1.535880961 | 1.87E-12 | Endometrium |
| 7409 | VAV1 | -1.535819939 | 1.05E-14 | Endometrium |
| 4210 | MEFV | -1.535260676 | 4.09E-14 | Endometrium |
| 11027 | LILRA2 | -1.535079109 | 1.41E-13 | Endometrium |
| 7185 | TRAF1 | -1.53466291 | 3.32E-27 | Endometrium |
| 1520 | CTSS | -1.534399569 | 3.08E-16 | Endometrium |
| 6916 | TBXAS1 | -1.532840876 | 1.20E-18 | Endometrium |
| 4969 | OGN | -1.532257059 | 3.92E-05 | Endometrium |
| 5918 | RARRES1 | -1.528796819 | 5.87E-07 | Endometrium |
| 29125 | C11orf21 | -1.528711226 | 2.15E-12 | Endometrium |
| 201305 | SPNS3 | -1.527761615 | 1.29E-10 | Endometrium |
| 100126784 | None | -1.526893711 | 1.90E-05 | Endometrium |
| 197358 | NLRC3 | -1.526770544 | 1.77E-24 | Endometrium |
| 9719 | ADAMTSL2 | -1.525636023 | 2.93E-08 | Endometrium |
| 8651 | SOCS1 | -1.525340403 | 1.15E-17 | Endometrium |
| 50863 | NTM | -1.524650979 | 2.36E-11 | Endometrium |
| 9056 | SLC7A7 | -1.524634407 | 2.28E-17 | Endometrium |
| 339145 | FAM92B | -1.524633639 | 0.00049687 | Endometrium |
| 27040 | LAT | -1.524256026 | 2.44E-17 | Endometrium |
| 10871 | CD300C | -1.524216102 | 3.78E-16 | Endometrium |
| 1116 | CHI3L1 | -1.522567829 | 6.17E-07 | Endometrium |
| 3108 | HLA-DMA | -1.52197673 | 1.75E-14 | Endometrium |
| 11254 | SLC6A14 | -1.520875093 | 0.000795988 | Endometrium |
| 3671 | ISLR | -1.520327022 | 5.36E-11 | Endometrium |
| 7052 | TGM2 | -1.518776409 | 8.09E-17 | Endometrium |
| 11026 | None | -1.51816681 | 7.41E-10 | Endometrium |
| 10750 | GRAP | -1.516669601 | 3.49E-16 | Endometrium |
| 149563 | SRARP | -1.516513641 | 0.001858881 | Endometrium |
| 1193 | CLIC2 | -1.515682313 | 3.64E-20 | Endometrium |
| 56659 | KCNK13 | -1.515383793 | 4.39E-12 | Endometrium |
| 5241 | PGR | -1.513267488 | 0.000869525 | Endometrium |
| 7941 | PLA2G7 | -1.513238285 | 6.99E-11 | Endometrium |
| 284417 | TMEM150B | -1.51307392 | 6.29E-10 | Endometrium |
| 862 | RUNX1T1 | -1.51303721 | 6.03E-10 | Endometrium |
| 1890 | TYMP | -1.512044313 | 2.30E-14 | Endometrium |
| 4360 | MRC1 | -1.511778761 | 1.48E-10 | Endometrium |
| 8605 | PLA2G4C | -1.511193153 | 8.49E-18 | Endometrium |
| 50944 | SHANK1 | -1.511046593 | 8.61E-06 | Endometrium |
| 2687 | GGT5 | -1.509550559 | 7.61E-20 | Endometrium |
| 8728 | ADAM19 | -1.509465491 | 2.66E-20 | Endometrium |
| 326624 | RAB37 | -1.509288494 | 1.55E-12 | Endometrium |
| 6372 | CXCL6 | -1.507521134 | 1.58E-06 | Endometrium |
| 126014 | OSCAR | -1.507447494 | 5.29E-17 | Endometrium |
| 1735 | DIO3 | -1.507309918 | 8.24E-08 | Endometrium |
| 79625 | NDNF | -1.505984424 | 3.24E-07 | Endometrium |
| 23166 | STAB1 | -1.505885227 | 8.69E-26 | Endometrium |
| 22806 | IKZF3 | -1.505781051 | 2.42E-08 | Endometrium |
| 79686 | None | -1.504648265 | 1.75E-16 | Endometrium |
| 5293 | PIK3CD | -1.503378924 | 2.13E-21 | Endometrium |
| 3059 | HCLS1 | -1.503176847 | 8.93E-12 | Endometrium |
| 3908 | LAMA2 | -1.500898719 | 1.91E-09 | Endometrium |
| 51087 | YBX2 | 1.601488563 | 4.41E-05 | Endometrium |
| 7138 | TNNT1 | 1.991080951 | 9.64E-05 | Endometrium |
| 114788 | CSMD3 | -4.966501448 | 2.65E-59 | Glioma |
| 2834 | PRLHR | -4.866399162 | 3.79E-54 | Glioma |
| 3822 | KLRC2 | -4.674500775 | 2.34E-41 | Glioma |
| 23316 | CUX2 | -4.557602641 | 6.70E-58 | Glioma |
| 7143 | TNR | -4.541591161 | 1.65E-51 | Glioma |
| 10369 | CACNG2 | -4.45056275 | 1.68E-49 | Glioma |
| 80309 | SPHKAP | -4.443289622 | 1.57E-50 | Glioma |
| 6511 | SLC1A6 | -4.421846038 | 1.27E-51 | Glioma |
| 6751 | SSTR1 | -4.388693867 | 6.92E-54 | Glioma |
| 869 | CBLN1 | -4.377068773 | 1.25E-63 | Glioma |
| 65217 | PCDH15 | -4.282777249 | 6.52E-44 | Glioma |
| 440730 | TRIM67 | -4.131410342 | 5.28E-37 | Glioma |
| 205147 | AMER3 | -4.111700389 | 2.47E-47 | Glioma |
| 9118 | INA | -4.09788131 | 2.01E-47 | Glioma |
| 8001 | GLRA3 | -4.092068151 | 4.32E-51 | Glioma |
| 55530 | SVOP | -4.042362202 | 2.52E-35 | Glioma |
| 57338 | JPH3 | -4.010355527 | 9.43E-53 | Glioma |
| 2334 | AFF2 | -3.955848316 | 3.32E-48 | Glioma |
| 285093 | RTP5 | -3.943133121 | 5.28E-49 | Glioma |
| 2902 | GRIN1 | -3.919509809 | 3.09E-28 | Glioma |
| 161357 | MDGA2 | -3.909927907 | 4.15E-51 | Glioma |
| 114794 | ELFN2 | -3.892266875 | 4.22E-51 | Glioma |
| 27328 | PCDH11X | -3.891475192 | 7.70E-45 | Glioma |
| 51046 | ST8SIA3 | -3.85853514 | 1.14E-44 | Glioma |
| 9699 | RIMS2 | -3.85148854 | 9.77E-44 | Glioma |
| 83482 | SCRT1 | -3.846018447 | 7.97E-49 | Glioma |
| 9892 | SNAP91 | -3.775877748 | 2.51E-46 | Glioma |
| 5067 | CNTN3 | -3.762535348 | 3.38E-41 | Glioma |
| 1016 | CDH18 | -3.740986198 | 5.53E-43 | Glioma |
| 2674 | GFRA1 | -3.739329 | 1.20E-46 | Glioma |
| 57144 | PAK5 | -3.736049526 | 3.64E-49 | Glioma |
| 64093 | SMOC1 | -3.730693147 | 4.63E-42 | Glioma |
| 64901 | RANBP17 | -3.724609885 | 5.26E-57 | Glioma |
| 51617 | NSG2 | -3.720699473 | 5.68E-46 | Glioma |
| 1137 | CHRNA4 | -3.710569542 | 1.56E-48 | Glioma |
| 117154 | DACH2 | -3.692555235 | 8.02E-44 | Glioma |
| 5126 | PCSK2 | -3.658674138 | 3.05E-35 | Glioma |
| 3166 | HMX1 | -3.630014086 | 4.48E-33 | Glioma |
| 1113 | CHGA | -3.625649546 | 2.37E-31 | Glioma |
| 222008 | VSTM2A | -3.57927151 | 3.25E-33 | Glioma |
| 2566 | GABRG2 | -3.569567277 | 2.36E-26 | Glioma |
| 55118 | CRTAC1 | -3.546509353 | 9.09E-55 | Glioma |
| 3823 | KLRC3 | -3.538508238 | 2.20E-41 | Glioma |
| 2840 | GPR17 | -3.537047368 | 8.42E-30 | Glioma |
| 283455 | KSR2 | -3.534217234 | 1.22E-35 | Glioma |
| 654429 | LRTM2 | -3.531332513 | 1.17E-34 | Glioma |
| 129684 | CNTNAP5 | -3.51101036 | 8.64E-45 | Glioma |
| 57453 | DSCAML1 | -3.509035515 | 1.01E-53 | Glioma |
| 11255 | HRH3 | -3.507599041 | 2.00E-37 | Glioma |
| 2562 | GABRB3 | -3.470501587 | 7.06E-46 | Glioma |
| 728643 | None | -3.468458533 | 3.54E-44 | Glioma |
| 7434 | VIPR2 | -3.464791792 | 1.18E-28 | Glioma |
| 2556 | GABRA3 | -3.443328862 | 3.11E-43 | Glioma |
| 11122 | PTPRT | -3.437194419 | 1.73E-45 | Glioma |
| 343702 | XKR7 | -3.428576351 | 1.24E-39 | Glioma |
| 85352 | SHISAL1 | -3.423997815 | 2.60E-42 | Glioma |
| 114805 | GALNT13 | -3.423826124 | 7.39E-41 | Glioma |
| 83955 | None | -3.421165303 | 4.83E-42 | Glioma |
| 202559 | KHDRBS2 | -3.412369946 | 2.62E-46 | Glioma |
| 220164 | DOK6 | -3.401165273 | 7.90E-65 | Glioma |
| 51412 | ACTL6B | -3.390010053 | 8.01E-35 | Glioma |
| 2774 | GNAL | -3.381558119 | 1.96E-56 | Glioma |
| 10814 | CPLX2 | -3.379875094 | 1.04E-30 | Glioma |
| 91156 | IGFN1 | -3.376967013 | 4.43E-25 | Glioma |
| 22941 | SHANK2 | -3.376370787 | 4.21E-51 | Glioma |
| 389206 | BEND4 | -3.366245798 | 4.30E-48 | Glioma |
| 114798 | SLITRK1 | -3.365881411 | 1.01E-44 | Glioma |
| 2565 | GABRG1 | -3.36390829 | 8.00E-28 | Glioma |
| 1038 | CDR1 | -3.361160455 | 7.29E-45 | Glioma |
| 56961 | SHD | -3.357649657 | 8.57E-42 | Glioma |
| 79948 | PLPPR3 | -3.353728449 | 2.03E-35 | Glioma |
| 283576 | ZDHHC22 | -3.352795219 | 2.23E-56 | Glioma |
| 23040 | MYT1L | -3.341341526 | 1.68E-25 | Glioma |
| 56934 | CA10 | -3.329278946 | 2.86E-31 | Glioma |
| 60495 | HPSE2 | -3.271539749 | 9.05E-27 | Glioma |
| 23072 | HECW1 | -3.264504684 | 9.46E-42 | Glioma |
| 1114 | CHGB | -3.26112375 | 7.01E-51 | Glioma |
| 729956 | SHISA7 | -3.258185487 | 2.37E-47 | Glioma |
| 7477 | WNT7B | -3.255963391 | 1.11E-41 | Glioma |
| 5593 | PRKG2 | -3.253511264 | 3.96E-34 | Glioma |
| 26032 | SUSD5 | -3.23730945 | 5.20E-42 | Glioma |
| 85300 | ATCAY | -3.236890498 | 3.57E-43 | Glioma |
| 9671 | WSCD2 | -3.226034177 | 3.43E-29 | Glioma |
| 80070 | ADAMTS20 | -3.217577666 | 2.38E-34 | Glioma |
| 4661 | MYT1 | -3.215747904 | 1.84E-34 | Glioma |
| 223117 | SEMA3D | -3.21407925 | 8.15E-29 | Glioma |
| 3745 | KCNB1 | -3.211562258 | 7.84E-53 | Glioma |
| 2498 | None | -3.210199483 | 6.27E-27 | Glioma |
| 81832 | NETO1 | -3.201874214 | 7.48E-41 | Glioma |
| 147381 | CBLN2 | -3.145664577 | 7.34E-35 | Glioma |
| 84684 | INSM2 | -3.139278088 | 5.01E-38 | Glioma |
| 5995 | RGR | -3.137141026 | 7.21E-40 | Glioma |
| 6543 | SLC8A2 | -3.12894881 | 3.27E-35 | Glioma |
| 10683 | None | -3.125717667 | 1.12E-33 | Glioma |
| 1159 | CKMT1B | -3.124466203 | 1.27E-37 | Glioma |
| 55612 | FERMT1 | -3.094601184 | 2.13E-46 | Glioma |
| 441666 | None | -3.094321312 | 1.30E-40 | Glioma |
| 774 | CACNA1B | -3.09176345 | 8.22E-26 | Glioma |
| 4168 | MCF2 | -3.082197971 | 1.96E-38 | Glioma |
| 5063 | PAK3 | -3.074387082 | 3.42E-41 | Glioma |
| 23544 | SEZ6L | -3.066474914 | 5.86E-42 | Glioma |
| 340745 | LRIT2 | -3.061558237 | 6.95E-57 | Glioma |
| 154822 | None | -3.060359037 | 7.29E-19 | Glioma |
| 153572 | IRX2 | -3.060010145 | 1.50E-17 | Glioma |
| 5075 | PAX1 | -3.053129003 | 2.39E-26 | Glioma |
| 6833 | ABCC8 | -3.051265001 | 1.16E-38 | Glioma |
| 1128 | CHRM1 | -3.050311049 | 1.32E-29 | Glioma |
| 492 | ATP2B3 | -3.043058417 | 2.52E-28 | Glioma |
| 56884 | FSTL5 | -3.040945236 | 7.37E-29 | Glioma |
| 51761 | ATP8A2 | -3.033400189 | 8.50E-29 | Glioma |
| 440040 | None | -3.027508999 | 1.83E-43 | Glioma |
| 9356 | SLC22A6 | -3.025951051 | 1.35E-28 | Glioma |
| 118738 | ZNF488 | -3.009387813 | 2.99E-38 | Glioma |
| 30819 | KCNIP2 | -3.001820911 | 4.86E-50 | Glioma |
| 124739 | USP43 | -2.998556249 | 8.73E-38 | Glioma |
| 50614 | GALNT9 | -2.984681761 | 1.92E-33 | Glioma |
| 83698 | CALN1 | -2.984552548 | 8.08E-32 | Glioma |
| 26047 | CNTNAP2 | -2.983042202 | 1.44E-43 | Glioma |
| 148198 | ZNF98 | -2.982136845 | 1.62E-46 | Glioma |
| 85508 | SCRT2 | -2.976363304 | 4.25E-25 | Glioma |
| 57574 | Mar-04 | -2.974665966 | 4.11E-30 | Glioma |
| 92211 | CDHR1 | -2.9741636 | 8.83E-48 | Glioma |
| 8302 | KLRC4 | -2.968971912 | 6.68E-38 | Glioma |
| 1993 | ELAVL2 | -2.968187264 | 8.82E-38 | Glioma |
| 643387 | None | -2.966877354 | 7.25E-34 | Glioma |
| 340554 | ZC3H12B | -2.964552044 | 1.83E-45 | Glioma |
| 642968 | FAM163B | -2.962609955 | 4.91E-28 | Glioma |
| 338645 | LUZP2 | -2.962599506 | 1.09E-29 | Glioma |
| 121256 | TMEM132D | -2.955813415 | 9.32E-21 | Glioma |
| 139189 | DGKK | -2.935133323 | 2.69E-27 | Glioma |
| 442454 | None | -2.922364708 | 4.11E-43 | Glioma |
| 1641 | DCX | -2.915106193 | 1.02E-33 | Glioma |
| 3897 | L1CAM | -2.913745743 | 2.82E-26 | Glioma |
| 64579 | NDST4 | -2.912553239 | 6.26E-35 | Glioma |
| 548596 | CKMT1A | -2.90279313 | 5.40E-33 | Glioma |
| 3350 | HTR1A | -2.900274785 | 2.12E-33 | Glioma |
| 23671 | TMEFF2 | -2.898709784 | 1.97E-34 | Glioma |
| 285696 | None | -2.898615787 | 1.85E-52 | Glioma |
| 54886 | PLPPR1 | -2.893407739 | 4.11E-35 | Glioma |
| 64211 | LHX5 | -2.8931695 | 1.87E-24 | Glioma |
| 728215 | FAM155A | -2.892193642 | 2.85E-50 | Glioma |
| 442319 | ZNF727 | -2.886165028 | 2.79E-37 | Glioma |
| 286499 | FAM133A | -2.884481786 | 4.48E-46 | Glioma |
| 5949 | RBP3 | -2.883435173 | 1.11E-50 | Glioma |
| 84502 | JPH4 | -2.881217429 | 3.90E-54 | Glioma |
| 23426 | GRIP1 | -2.875974564 | 6.04E-40 | Glioma |
| 8549 | LGR5 | -2.872580236 | 7.02E-41 | Glioma |
| 23732 | FRRS1L | -2.863286689 | 9.59E-38 | Glioma |
| 3765 | KCNJ9 | -2.859191413 | 1.05E-50 | Glioma |
| 80000 | GREB1L | -2.858020596 | 6.22E-32 | Glioma |
| 3777 | KCNK3 | -2.856309674 | 4.05E-43 | Glioma |
| 30812 | SOX8 | -2.85347092 | 8.62E-50 | Glioma |
| 57624 | NYAP2 | -2.852691987 | 1.09E-36 | Glioma |
| 56475 | RPRM | -2.848375789 | 7.13E-37 | Glioma |
| 116443 | GRIN3A | -2.836383579 | 5.27E-36 | Glioma |
| 6456 | SH3GL2 | -2.832079235 | 6.55E-35 | Glioma |
| 57628 | DPP10 | -2.829830249 | 1.69E-34 | Glioma |
| 140886 | PABPC5 | -2.823307112 | 2.70E-38 | Glioma |
| 115827 | RAB3C | -2.820082146 | 4.03E-34 | Glioma |
| 9241 | NOG | -2.816436496 | 3.00E-59 | Glioma |
| 57662 | CAMSAP3 | -2.815750564 | 7.60E-35 | Glioma |
| 145581 | LRFN5 | -2.80624881 | 1.30E-28 | Glioma |
| 221938 | MMD2 | -2.804823739 | 3.95E-37 | Glioma |
| 26507 | CNNM1 | -2.803816241 | 1.74E-28 | Glioma |
| 441032 | None | -2.801858232 | 3.15E-45 | Glioma |
| 93429 | None | -2.80050061 | 8.71E-29 | Glioma |
| 23101 | MCF2L2 | -2.783557003 | 5.43E-48 | Glioma |
| 60680 | CELF5 | -2.782242463 | 8.78E-38 | Glioma |
| 646113 | None | -2.780798771 | 1.07E-26 | Glioma |
| 80059 | LRRTM4 | -2.769717552 | 3.73E-40 | Glioma |
| 2895 | GRID2 | -2.76723013 | 6.36E-30 | Glioma |
| 6712 | SPTBN2 | -2.758709118 | 9.99E-67 | Glioma |
| 114824 | PNMA5 | -2.757333634 | 1.04E-28 | Glioma |
| 3747 | KCNC2 | -2.752935196 | 8.95E-18 | Glioma |
| 9185 | REPS2 | -2.752578999 | 3.19E-56 | Glioma |
| 57578 | UNC79 | -2.750166353 | 7.92E-49 | Glioma |
| 338811 | FAM19A2 | -2.742442955 | 3.24E-42 | Glioma |
| 29953 | TRHDE | -2.73909439 | 2.00E-22 | Glioma |
| 23769 | FLRT1 | -2.731816355 | 1.70E-55 | Glioma |
| 11189 | CELF3 | -2.730227347 | 2.96E-33 | Glioma |
| 284656 | EPHA10 | -2.722695079 | 1.23E-32 | Glioma |
| 10083 | USH1C | -2.720567797 | 9.04E-20 | Glioma |
| 768096 | None | -2.720053972 | 6.04E-34 | Glioma |
| 4741 | NEFM | -2.716856964 | 2.93E-16 | Glioma |
| 93426 | SYCE1 | -2.715275211 | 4.05E-17 | Glioma |
| 90249 | UNC5A | -2.709413301 | 6.71E-43 | Glioma |
| 129049 | SGSM1 | -2.701948626 | 1.20E-45 | Glioma |
| 56853 | CELF4 | -2.701761727 | 2.09E-27 | Glioma |
| 342865 | VSTM2B | -2.699375737 | 1.29E-28 | Glioma |
| 9229 | DLGAP1 | -2.698334041 | 6.77E-62 | Glioma |
| 145282 | MIPOL1 | -2.695908554 | 1.24E-36 | Glioma |
| 478 | ATP1A3 | -2.690620642 | 3.64E-40 | Glioma |
| 441151 | TMEM151B | -2.689056163 | 2.21E-41 | Glioma |
| 6549 | SLC9A2 | -2.688647075 | 2.12E-38 | Glioma |
| 5662 | PSD | -2.688478803 | 1.08E-43 | Glioma |
| 57709 | SLC7A14 | -2.684924025 | 8.96E-33 | Glioma |
| 11141 | IL1RAPL1 | -2.683974255 | 1.71E-53 | Glioma |
| 254559 | None | -2.681295579 | 7.87E-47 | Glioma |
| 27330 | RPS6KA6 | -2.672938716 | 1.81E-29 | Glioma |
| 146713 | RBFOX3 | -2.664602526 | 1.79E-20 | Glioma |
| 78986 | DUSP26 | -2.662072928 | 7.68E-52 | Glioma |
| 440073 | IQSEC3 | -2.660666709 | 1.69E-26 | Glioma |
| 2653 | GCSH | -2.658595166 | 1.62E-40 | Glioma |
| 1996 | ELAVL4 | -2.652164516 | 4.65E-33 | Glioma |
| 84530 | SRRM4 | -2.646962869 | 1.01E-23 | Glioma |
| 6860 | SYT4 | -2.64578455 | 2.28E-20 | Glioma |
| 388585 | HES5 | -2.642227524 | 1.96E-24 | Glioma |
| 4624 | MYH6 | -2.641973543 | 1.64E-32 | Glioma |
| 3358 | HTR2C | -2.641506043 | 2.44E-28 | Glioma |
| 78997 | GDAP1L1 | -2.641234236 | 2.11E-45 | Glioma |
| 646405 | None | -2.640711559 | 2.69E-51 | Glioma |
| 83873 | GPR61 | -2.638667429 | 2.74E-42 | Glioma |
| 283248 | RCOR2 | -2.638395745 | 4.23E-55 | Glioma |
| 5138 | PDE2A | -2.633994918 | 4.28E-55 | Glioma |
| 6423 | SFRP2 | -2.629702203 | 2.97E-16 | Glioma |
| 10777 | ARPP21 | -2.628266449 | 1.15E-46 | Glioma |
| 100188954 | None | -2.628172718 | 6.35E-30 | Glioma |
| 2914 | GRM4 | -2.623063276 | 3.99E-27 | Glioma |
| 5923 | RASGRF1 | -2.608316017 | 4.06E-27 | Glioma |
| 2554 | GABRA1 | -2.607614873 | 1.79E-13 | Glioma |
| 22999 | RIMS1 | -2.605510926 | 6.17E-31 | Glioma |
| 10633 | RASL10A | -2.605169417 | 5.83E-42 | Glioma |
| 440279 | UNC13C | -2.601075892 | 1.91E-16 | Glioma |
| 64881 | PCDH20 | -2.600845632 | 2.47E-37 | Glioma |
| 494470 | RNF165 | -2.600786253 | 2.66E-49 | Glioma |
| 157627 | None | -2.59690812 | 2.07E-26 | Glioma |
| 6547 | SLC8A3 | -2.593356571 | 6.92E-38 | Glioma |
| 55506 | H2AFY2 | -2.59287499 | 1.07E-51 | Glioma |
| 57221 | ARFGEF3 | -2.590393007 | 3.57E-44 | Glioma |
| 25834 | MGAT4C | -2.588834365 | 4.01E-26 | Glioma |
| 440435 | GPR179 | -2.585899782 | 3.78E-45 | Glioma |
| 1005 | CDH7 | -2.582263771 | 8.67E-31 | Glioma |
| 348980 | HCN1 | -2.58142521 | 2.71E-17 | Glioma |
| 8911 | CACNA1I | -2.572048405 | 1.06E-27 | Glioma |
| 30818 | KCNIP3 | -2.571185923 | 1.60E-50 | Glioma |
| 79570 | NKAIN1 | -2.567591244 | 1.61E-36 | Glioma |
| 23025 | UNC13A | -2.564822489 | 1.78E-44 | Glioma |
| 55515 | ASIC4 | -2.561498276 | 2.83E-28 | Glioma |
| 27254 | CSDC2 | -2.550446509 | 3.05E-39 | Glioma |
| 29106 | SCG3 | -2.549631131 | 9.05E-49 | Glioma |
| 1007 | CDH9 | -2.545719686 | 4.50E-23 | Glioma |
| 23057 | NMNAT2 | -2.536558336 | 9.32E-43 | Glioma |
| 1852 | DUSP9 | -2.531114189 | 3.41E-32 | Glioma |
| 6585 | SLIT1 | -2.529646402 | 2.97E-38 | Glioma |
| 10368 | CACNG3 | -2.529012226 | 9.13E-14 | Glioma |
| 55800 | SCN3B | -2.525601821 | 3.06E-34 | Glioma |
| 91752 | ZNF804A | -2.524112027 | 4.49E-51 | Glioma |
| 57582 | KCNT1 | -2.522651957 | 9.64E-18 | Glioma |
| 163223 | ZNF676 | -2.522003197 | 4.65E-22 | Glioma |
| 650 | BMP2 | -2.52128945 | 1.85E-52 | Glioma |
| 575 | ADGRB1 | -2.51979396 | 4.71E-30 | Glioma |
| 147741 | ZNF560 | -2.517069274 | 2.05E-15 | Glioma |
| 84302 | TMEM246 | -2.51399727 | 5.37E-40 | Glioma |
| 64405 | CDH22 | -2.509463952 | 1.58E-26 | Glioma |
| 26038 | CHD5 | -2.507011218 | 8.25E-20 | Glioma |
| 2258 | FGF13 | -2.504723756 | 6.08E-30 | Glioma |
| 158696 | None | -2.500149079 | 8.23E-30 | Glioma |
| 134829 | CLVS2 | -2.498273939 | 1.80E-21 | Glioma |
| 23109 | DDN | -2.497614405 | 1.42E-21 | Glioma |
| 283392 | None | -2.493166114 | 1.50E-37 | Glioma |
| 148145 | None | -2.492884422 | 2.47E-23 | Glioma |
| 3195 | TLX1 | -2.481598348 | 4.19E-14 | Glioma |
| 286411 | None | -2.480855535 | 4.80E-36 | Glioma |
| 2893 | GRIA4 | -2.471660449 | 1.91E-38 | Glioma |
| 2915 | GRM5 | -2.470703721 | 4.88E-23 | Glioma |
| 155185 | AMZ1 | -2.469292761 | 7.69E-41 | Glioma |
| 2740 | GLP1R | -2.46902192 | 1.40E-23 | Glioma |
| 7253 | TSHR | -2.468353314 | 4.64E-17 | Glioma |
| 100192386 | None | -2.466925162 | 2.92E-26 | Glioma |
| 8128 | ST8SIA2 | -2.461556207 | 5.53E-21 | Glioma |
| 2550 | GABBR1 | -2.461254117 | 3.38E-63 | Glioma |
| 4330 | MN1 | -2.461132337 | 1.66E-49 | Glioma |
| 84465 | MEGF11 | -2.455768461 | 1.17E-42 | Glioma |
| 27324 | TOX3 | -2.4541943 | 1.72E-35 | Glioma |
| 26052 | DNM3 | -2.452774699 | 1.26E-64 | Glioma |
| 10900 | RUNDC3A | -2.44937957 | 1.17E-50 | Glioma |
| 116154 | PHACTR3 | -2.446605252 | 6.73E-37 | Glioma |
| 148398 | SAMD11 | -2.442840001 | 7.76E-29 | Glioma |
| 2662 | GDF10 | -2.440572784 | 7.92E-19 | Glioma |
| 134701 | RIPPLY2 | -2.440381356 | 1.90E-41 | Glioma |
| 26050 | SLITRK5 | -2.439092325 | 1.55E-50 | Glioma |
| 2138 | EYA1 | -2.437931884 | 1.39E-22 | Glioma |
| 2047 | EPHB1 | -2.436734923 | 1.23E-42 | Glioma |
| 114786 | XKR4 | -2.425902638 | 6.40E-37 | Glioma |
| 57524 | CASKIN1 | -2.422826127 | 1.15E-44 | Glioma |
| 9312 | KCNB2 | -2.420693657 | 1.31E-23 | Glioma |
| 441061 | Mar-11 | -2.4200171 | 3.47E-32 | Glioma |
| 644936 | None | -2.418692586 | 6.89E-25 | Glioma |
| 80319 | CXXC4 | -2.41567905 | 3.34E-49 | Glioma |
| 5733 | PTGER3 | -2.41512234 | 5.86E-23 | Glioma |
| 26153 | KIF26A | -2.409735953 | 9.09E-42 | Glioma |
| 257194 | NEGR1 | -2.408198535 | 4.33E-34 | Glioma |
| 345630 | FBLL1 | -2.399966427 | 5.54E-35 | Glioma |
| 4625 | MYH7 | -2.396971697 | 4.32E-27 | Glioma |
| 9348 | NDST3 | -2.396835386 | 1.24E-19 | Glioma |
| 8123 | None | -2.39613009 | 3.10E-41 | Glioma |
| 3767 | KCNJ11 | -2.394570644 | 6.94E-36 | Glioma |
| 1141 | CHRNB2 | -2.392956819 | 7.47E-45 | Glioma |
| 58512 | DLGAP3 | -2.391917608 | 9.19E-37 | Glioma |
| 84856 | None | -2.388740435 | 9.39E-20 | Glioma |
| 6444 | SGCD | -2.386001295 | 1.83E-29 | Glioma |
| 55512 | SMPD3 | -2.385938643 | 1.47E-50 | Glioma |
| 8224 | SYN3 | -2.384039393 | 7.98E-40 | Glioma |
| 6328 | SCN3A | -2.378991563 | 9.32E-46 | Glioma |
| 2891 | GRIA2 | -2.372996776 | 1.15E-42 | Glioma |
| 158228 | None | -2.372840745 | 1.36E-28 | Glioma |
| 56479 | KCNQ5 | -2.368232982 | 7.30E-30 | Glioma |
| 105 | ADARB2 | -2.368221935 | 1.89E-25 | Glioma |
| 140767 | NRSN1 | -2.364504247 | 9.14E-37 | Glioma |
| 10202 | DHRS2 | -2.364246538 | 1.35E-23 | Glioma |
| 10690 | FUT9 | -2.358258715 | 6.03E-34 | Glioma |
| 3756 | KCNH1 | -2.357905431 | 1.45E-30 | Glioma |
| 23046 | KIF21B | -2.35547724 | 2.19E-50 | Glioma |
| 25787 | None | -2.355088066 | 3.53E-51 | Glioma |
| 6854 | SYN2 | -2.353463795 | 2.49E-16 | Glioma |
| 401145 | CCSER1 | -2.350175861 | 2.09E-34 | Glioma |
| 57495 | NWD2 | -2.347537853 | 1.30E-16 | Glioma |
| 3760 | KCNJ3 | -2.344218587 | 1.42E-20 | Glioma |
| 50944 | SHANK1 | -2.343766292 | 5.41E-26 | Glioma |
| 29119 | CTNNA3 | -2.340481525 | 1.70E-29 | Glioma |
| 89874 | SLC25A21 | -2.332654816 | 3.92E-41 | Glioma |
| 1047 | CLGN | -2.331902739 | 1.18E-26 | Glioma |
| 83723 | FAM57B | -2.327087891 | 2.22E-43 | Glioma |
| 2904 | GRIN2B | -2.320785048 | 2.37E-22 | Glioma |
| 57586 | SYT13 | -2.319526156 | 2.16E-16 | Glioma |
| 4760 | NEUROD1 | -2.315595105 | 2.87E-21 | Glioma |
| 8499 | PPFIA2 | -2.311411442 | 2.22E-29 | Glioma |
| 5582 | PRKCG | -2.309865171 | 8.51E-15 | Glioma |
| 28514 | DLL1 | -2.307990103 | 2.89E-48 | Glioma |
| 30010 | NXPH1 | -2.305293601 | 6.20E-29 | Glioma |
| 284836 | None | -2.305140331 | 9.69E-38 | Glioma |
| 8927 | BSN | -2.302163598 | 3.20E-45 | Glioma |
| 10815 | CPLX1 | -2.301603356 | 1.76E-33 | Glioma |
| 256130 | TMEM196 | -2.301029967 | 2.77E-16 | Glioma |
| 246213 | SLC17A8 | -2.299865815 | 3.01E-13 | Glioma |
| 781 | CACNA2D1 | -2.299675404 | 2.20E-21 | Glioma |
| 63982 | ANO3 | -2.298335725 | 1.85E-24 | Glioma |
| 7092 | TLL1 | -2.296221354 | 5.25E-23 | Glioma |
| 90134 | KCNH7 | -2.295306585 | 5.47E-20 | Glioma |
| 6262 | RYR2 | -2.293533621 | 7.63E-14 | Glioma |
| 145837 | None | -2.291484928 | 2.15E-49 | Glioma |
| 401190 | RGS7BP | -2.28643935 | 1.70E-32 | Glioma |
| 26222 | None | -2.283420191 | 5.45E-40 | Glioma |
| 1951 | CELSR3 | -2.282924832 | 6.87E-42 | Glioma |
| 129807 | NEU4 | -2.281972936 | 1.44E-29 | Glioma |
| 242 | ALOX12B | -2.281519612 | 2.56E-32 | Glioma |
| 100131897 | INSYN2B | -2.278733597 | 1.65E-26 | Glioma |
| 387590 | None | -2.278050959 | 2.00E-14 | Glioma |
| 1394 | CRHR1 | -2.275914396 | 1.12E-28 | Glioma |
| 1630 | DCC | -2.271273932 | 5.11E-22 | Glioma |
| 9568 | GABBR2 | -2.265864163 | 7.07E-28 | Glioma |
| 2849 | GPR26 | -2.265343593 | 1.77E-12 | Glioma |
| 6752 | SSTR2 | -2.265178214 | 2.51E-38 | Glioma |
| 6457 | SH3GL3 | -2.260925629 | 4.27E-23 | Glioma |
| 9254 | CACNA2D2 | -2.259878103 | 8.23E-39 | Glioma |
| 22844 | FRMPD1 | -2.259420487 | 2.26E-42 | Glioma |
| 9478 | CABP1 | -2.258377716 | 2.01E-22 | Glioma |
| 862 | RUNX1T1 | -2.256820899 | 1.85E-43 | Glioma |
| 136227 | COL26A1 | -2.255395596 | 1.44E-21 | Glioma |
| 259232 | NALCN | -2.253890027 | 1.00E-47 | Glioma |
| 8851 | CDK5R1 | -2.252631631 | 2.56E-59 | Glioma |
| 84915 | FAM222A | -2.250037897 | 1.70E-50 | Glioma |
| 7368 | UGT8 | -2.249412056 | 9.06E-27 | Glioma |
| 9378 | NRXN1 | -2.249205221 | 2.44E-41 | Glioma |
| 23236 | PLCB1 | -2.248932391 | 8.23E-59 | Glioma |
| 386618 | KCTD4 | -2.244824333 | 6.57E-27 | Glioma |
| 112755 | STX1B | -2.241150641 | 1.42E-48 | Glioma |
| 92293 | TMEM132C | -2.240418847 | 6.75E-23 | Glioma |
| 83259 | PCDH11Y | -2.240378042 | 7.24E-20 | Glioma |
| 22983 | MAST1 | -2.239149142 | 4.07E-45 | Glioma |
| 1826 | DSCAM | -2.238489704 | 4.26E-38 | Glioma |
| 50632 | CALY | -2.237554639 | 9.93E-12 | Glioma |
| 84435 | ADGRA1 | -2.235244637 | 1.81E-40 | Glioma |
| 9228 | DLGAP2 | -2.232541381 | 6.39E-16 | Glioma |
| 257068 | PLCXD2 | -2.231570075 | 1.08E-45 | Glioma |
| 58158 | None | -2.231526 | 3.06E-18 | Glioma |
| 3768 | KCNJ12 | -2.228917913 | 1.20E-28 | Glioma |
| 64478 | CSMD1 | -2.228863603 | 3.42E-32 | Glioma |
| 4978 | OPCML | -2.227892888 | 2.57E-30 | Glioma |
| 50801 | KCNK4 | -2.225699694 | 7.78E-29 | Glioma |
| 5099 | PCDH7 | -2.223875103 | 7.49E-32 | Glioma |
| 57540 | DISP3 | -2.22292914 | 4.01E-37 | Glioma |
| 3356 | HTR2A | -2.22214339 | 8.39E-22 | Glioma |
| 9758 | FRMPD4 | -2.222125039 | 1.19E-15 | Glioma |
| 27255 | CNTN6 | -2.217743893 | 1.01E-20 | Glioma |
| 398 | ARHGDIG | -2.212404463 | 4.30E-22 | Glioma |
| 2155 | F7 | -2.199214713 | 1.90E-29 | Glioma |
| 534 | ATP6V1G2 | -2.199125209 | 1.03E-52 | Glioma |
| 389332 | SMIM32 | -2.194016162 | 3.52E-21 | Glioma |
| 55273 | TMEM100 | -2.192089514 | 8.40E-35 | Glioma |
| 84894 | LINGO1 | -2.187692333 | 8.37E-50 | Glioma |
| 285175 | UNC80 | -2.185685062 | 1.82E-44 | Glioma |
| 2571 | GAD1 | -2.180315732 | 1.89E-30 | Glioma |
| 9912 | ARHGAP44 | -2.17852019 | 3.90E-31 | Glioma |
| 653598 | PPIAL4C | -2.177374991 | 2.21E-32 | Glioma |
| 29993 | PACSIN1 | -2.176652091 | 9.90E-12 | Glioma |
| 10396 | ATP8A1 | -2.173138918 | 1.56E-49 | Glioma |
| 100033802 | None | -2.172675409 | 1.09E-28 | Glioma |
| 407738 | FAM19A1 | -2.16220259 | 6.80E-16 | Glioma |
| 5649 | RELN | -2.161974772 | 2.83E-16 | Glioma |
| 55073 | None | -2.160692444 | 7.30E-47 | Glioma |
| 347730 | LRRTM1 | -2.159432525 | 1.46E-39 | Glioma |
| 90113 | VWA5B2 | -2.158646314 | 4.59E-30 | Glioma |
| 7138 | TNNT1 | -2.158027221 | 3.81E-17 | Glioma |
| 146330 | FBXL16 | -2.155120692 | 8.61E-34 | Glioma |
| 3361 | HTR5A | -2.153789538 | 9.67E-15 | Glioma |
| 4878 | NPPA | -2.153743915 | 1.24E-16 | Glioma |
| 386757 | None | -2.151640803 | 6.07E-30 | Glioma |
| 22986 | SORCS3 | -2.147291032 | 1.89E-32 | Glioma |
| 65268 | WNK2 | -2.146514109 | 1.68E-17 | Glioma |
| 758 | MPPED1 | -2.144285015 | 2.17E-12 | Glioma |
| 2254 | FGF9 | -2.144076925 | 3.31E-24 | Glioma |
| 153328 | SLC25A48 | -2.140218763 | 1.99E-19 | Glioma |
| 2561 | GABRB2 | -2.139539105 | 4.20E-13 | Glioma |
| 5444 | PON1 | -2.139047134 | 7.85E-26 | Glioma |
| 26033 | ATRNL1 | -2.136152732 | 2.15E-33 | Glioma |
| 222183 | SRRM3 | -2.133944878 | 2.93E-32 | Glioma |
| 57468 | SLC12A5 | -2.133356037 | 7.88E-15 | Glioma |
| 11250 | GPR45 | -2.131439373 | 3.34E-36 | Glioma |
| 378465 | None | -2.130029276 | 9.91E-37 | Glioma |
| 10740 | None | -2.128770529 | 3.09E-21 | Glioma |
| 57554 | LRRC7 | -2.124360461 | 3.92E-20 | Glioma |
| 1620 | BRINP1 | -2.120339319 | 1.15E-21 | Glioma |
| 54039 | PCBP3 | -2.118403808 | 1.36E-35 | Glioma |
| 59350 | RXFP1 | -2.117255322 | 5.97E-17 | Glioma |
| 340578 | DCAF12L2 | -2.116980443 | 5.08E-22 | Glioma |
| 6656 | SOX1 | -2.116761173 | 6.58E-22 | Glioma |
| 6853 | SYN1 | -2.116693196 | 6.22E-26 | Glioma |
| 56143 | PCDHA5 | -2.115525153 | 5.35E-31 | Glioma |
| 6534 | SLC6A7 | -2.11499152 | 1.04E-09 | Glioma |
| 6620 | SNCB | -2.113607764 | 2.04E-15 | Glioma |
| 79674 | VEPH1 | -2.112010061 | 2.19E-12 | Glioma |
| 1010 | CDH12 | -2.111724275 | 3.14E-25 | Glioma |
| 55359 | STYK1 | -2.108653535 | 1.59E-14 | Glioma |
| 283078 | MKX | -2.107922558 | 4.88E-15 | Glioma |
| 8170 | SLC14A2 | -2.106242453 | 3.72E-20 | Glioma |
| 2563 | GABRD | -2.102961372 | 4.54E-20 | Glioma |
| 6869 | TACR1 | -2.100411517 | 1.24E-19 | Glioma |
| 148014 | TTC9B | -2.098133118 | 1.89E-21 | Glioma |
| 1571 | CYP2E1 | -2.096238573 | 8.21E-35 | Glioma |
| 100130148 | None | -2.095582968 | 7.99E-38 | Glioma |
| 171019 | ADAMTS19 | -2.094645575 | 1.54E-23 | Glioma |
| 11069 | RAPGEF4 | -2.093019265 | 1.64E-41 | Glioma |
| 126520 | PLK5 | -2.092005596 | 7.23E-29 | Glioma |
| 285954 | None | -2.089798396 | 1.91E-21 | Glioma |
| 57642 | COL20A1 | -2.088762614 | 5.09E-11 | Glioma |
| 64409 | GALNT17 | -2.085550993 | 2.81E-13 | Glioma |
| 222901 | None | -2.085250252 | 1.59E-37 | Glioma |
| 283761 | None | -2.085200959 | 8.75E-32 | Glioma |
| 374383 | NCR3LG1 | -2.083115238 | 1.56E-37 | Glioma |
| 131096 | KCNH8 | -2.083033591 | 1.75E-24 | Glioma |
| 84913 | ATOH8 | -2.079021299 | 2.08E-40 | Glioma |
| 339674 | None | -2.078418651 | 1.09E-46 | Glioma |
| 84620 | ST6GAL2 | -2.078224497 | 2.95E-26 | Glioma |
| 79012 | CAMKV | -2.078201951 | 5.61E-19 | Glioma |
| 84628 | NTNG2 | -2.076964258 | 1.90E-48 | Glioma |
| 1272 | CNTN1 | -2.076843486 | 9.84E-31 | Glioma |
| 89958 | SAPCD2 | -2.073779984 | 1.65E-35 | Glioma |
| 9108 | MTMR7 | -2.072310218 | 7.08E-49 | Glioma |
| 605 | BCL7A | -2.064654814 | 3.61E-68 | Glioma |
| 80852 | GRIP2 | -2.062655109 | 7.10E-20 | Glioma |
| 152330 | CNTN4 | -2.062038113 | 4.15E-26 | Glioma |
| 285220 | EPHA6 | -2.061059129 | 3.10E-22 | Glioma |
| 27012 | KCNV1 | -2.059961523 | 9.47E-11 | Glioma |
| 114569 | MAL2 | -2.05949578 | 1.97E-11 | Glioma |
| 57512 | GPR158 | -2.059387585 | 8.41E-45 | Glioma |
| 80312 | TET1 | -2.054684128 | 2.43E-59 | Glioma |
| 10215 | OLIG2 | -2.053330779 | 3.53E-33 | Glioma |
| 190 | NR0B1 | -2.051000625 | 7.22E-15 | Glioma |
| 5874 | RAB27B | -2.049581875 | 6.98E-26 | Glioma |
| 8941 | CDK5R2 | -2.048872884 | 1.16E-21 | Glioma |
| 57615 | ZNF492 | -2.047256906 | 5.94E-27 | Glioma |
| 64850 | ETNPPL | -2.045539591 | 1.82E-12 | Glioma |
| 253314 | EIF4E1B | -2.042686073 | 1.14E-19 | Glioma |
| 9651 | PLCH2 | -2.041959747 | 1.82E-21 | Glioma |
| 6505 | SLC1A1 | -2.03747977 | 5.56E-46 | Glioma |
| 84457 | PHYHIPL | -2.036258502 | 1.13E-50 | Glioma |
| 165257 | C1QL2 | -2.035522859 | 9.99E-16 | Glioma |
| 23105 | FSTL4 | -2.032429525 | 2.58E-14 | Glioma |
| 388021 | TMEM179 | -2.031906009 | 9.03E-22 | Glioma |
| 153571 | C5orf38 | -2.030336179 | 1.37E-10 | Glioma |
| 3798 | KIF5A | -2.029395093 | 1.43E-27 | Glioma |
| 768097 | None | -2.02903675 | 5.38E-27 | Glioma |
| 80069 | None | -2.028257278 | 7.08E-35 | Glioma |
| 773 | CACNA1A | -2.024315298 | 1.47E-36 | Glioma |
| 25862 | USP49 | -2.023710917 | 1.83E-44 | Glioma |
| 2257 | FGF12 | -2.023533028 | 8.03E-41 | Glioma |
| 54550 | NECAB2 | -2.021123971 | 4.91E-28 | Glioma |
| 57644 | MYH7B | -2.018314878 | 9.58E-27 | Glioma |
| 6616 | SNAP25 | -2.014331511 | 7.59E-17 | Glioma |
| 3739 | KCNA4 | -2.013634805 | 2.21E-17 | Glioma |
| 7379 | UPK2 | -2.013285119 | 7.10E-42 | Glioma |
| 57084 | SLC17A6 | -2.012612597 | 8.01E-12 | Glioma |
| 9256 | TSPOAP1 | -2.011473005 | 2.72E-36 | Glioma |
| 56114 | PCDHGA1 | -2.007656837 | 7.49E-17 | Glioma |
| 28316 | CDH20 | -2.002149776 | 2.72E-34 | Glioma |
| 80816 | ASXL3 | -2.001595851 | 2.53E-34 | Glioma |
| 57818 | G6PC2 | -2.000167863 | 3.56E-20 | Glioma |
| 6770 | STAR | -1.999295598 | 1.14E-34 | Glioma |
| 165530 | CLEC4F | -1.997635818 | 5.86E-23 | Glioma |
| 9162 | DGKI | -1.997024078 | 1.80E-43 | Glioma |
| 59277 | NTN4 | -1.996592404 | 1.06E-31 | Glioma |
| 340533 | NEXMIF | -1.996569864 | 3.95E-24 | Glioma |
| 6658 | SOX3 | -1.993867298 | 2.79E-18 | Glioma |
| 150622 | None | -1.993472368 | 3.72E-28 | Glioma |
| 54715 | RBFOX1 | -1.991376232 | 1.49E-12 | Glioma |
| 388662 | SLC6A17 | -1.988716468 | 2.13E-14 | Glioma |
| 83849 | SYT15 | -1.987845122 | 1.89E-40 | Glioma |
| 9024 | BRSK2 | -1.986551244 | 7.90E-35 | Glioma |
| 10718 | NRG3 | -1.986097039 | 2.34E-24 | Glioma |
| 57030 | SLC17A7 | -1.982561691 | 2.75E-08 | Glioma |
| 7275 | TUB | -1.982462256 | 2.50E-61 | Glioma |
| 1607 | DGKB | -1.98240856 | 2.03E-30 | Glioma |
| 80034 | CSRNP3 | -1.981798823 | 4.29E-35 | Glioma |
| 650623 | None | -1.981673396 | 3.69E-42 | Glioma |
| 2898 | GRIK2 | -1.981475721 | 1.67E-31 | Glioma |
| 140679 | SLC32A1 | -1.980600896 | 2.53E-10 | Glioma |
| 375323 | LHFPL4 | -1.97653887 | 2.96E-25 | Glioma |
| 55964 | Sep-03 | -1.975742408 | 4.03E-52 | Glioma |
| 90668 | CARMIL3 | -1.974252073 | 4.10E-37 | Glioma |
| 57528 | KCTD16 | -1.974017686 | 4.23E-34 | Glioma |
| 54511 | HMGCLL1 | -1.971423765 | 6.44E-16 | Glioma |
| 9142 | None | -1.968015358 | 6.47E-28 | Glioma |
| 23217 | ZFR2 | -1.966444811 | 9.30E-13 | Glioma |
| 253559 | CADM2 | -1.966100513 | 8.17E-40 | Glioma |
| 2844 | RABGAP1 | -1.965457808 | 3.65E-26 | Glioma |
| 347731 | LRRTM3 | -1.96532884 | 2.02E-40 | Glioma |
| 4983 | OPHN1 | -1.962922519 | 2.96E-57 | Glioma |
| 134466 | None | -1.959301027 | 2.18E-14 | Glioma |
| 5502 | PPP1R1A | -1.956905711 | 3.79E-15 | Glioma |
| 6000 | RGS7 | -1.954351845 | 1.06E-21 | Glioma |
| 22997 | IGSF9B | -1.953879024 | 1.69E-29 | Glioma |
| 65078 | RTN4R | -1.953816655 | 1.18E-34 | Glioma |
| 4133 | MAP2 | -1.953085519 | 6.18E-63 | Glioma |
| 134111 | UBE2QL1 | -1.953084483 | 1.09E-30 | Glioma |
| 6857 | SYT1 | -1.949971311 | 6.91E-14 | Glioma |
| 140771 | None | -1.945092961 | 1.53E-36 | Glioma |
| 777 | CACNA1E | -1.944843656 | 2.19E-21 | Glioma |
| 51555 | PEX5L | -1.943445393 | 5.12E-19 | Glioma |
| 3131 | HLF | -1.943283987 | 3.56E-39 | Glioma |
| 222865 | TMEM130 | -1.943220418 | 1.01E-13 | Glioma |
| 2153 | F5 | -1.942273476 | 1.38E-10 | Glioma |
| 84618 | NT5C1A | -1.941050586 | 2.75E-21 | Glioma |
| 256536 | TCERG1L | -1.93943585 | 1.07E-14 | Glioma |
| 114795 | TMEM132B | -1.93942157 | 1.93E-22 | Glioma |
| 374786 | EFCAB5 | -1.937592378 | 2.29E-44 | Glioma |
| 111 | ADCY5 | -1.935050438 | 1.15E-34 | Glioma |
| 63974 | NEUROD6 | -1.934550617 | 2.61E-10 | Glioma |
| 29944 | PNMA3 | -1.932932263 | 5.54E-23 | Glioma |
| 3751 | KCND2 | -1.930989602 | 3.90E-23 | Glioma |
| 27099 | None | -1.930381878 | 2.61E-46 | Glioma |
| 2558 | GABRA5 | -1.930229065 | 1.97E-09 | Glioma |
| 2911 | GRM1 | -1.929539003 | 1.03E-21 | Glioma |
| 2850 | GPR27 | -1.928174763 | 4.85E-22 | Glioma |
| 4137 | MAPT | -1.926549546 | 2.79E-54 | Glioma |
| 90362 | FAM110B | -1.925911734 | 3.95E-72 | Glioma |
| 92196 | DAPL1 | -1.925762278 | 7.56E-13 | Glioma |
| 79152 | FA2H | -1.925427072 | 4.69E-17 | Glioma |
| 349136 | WDR86 | -1.924633455 | 1.38E-28 | Glioma |
| 442117 | GALNTL6 | -1.922574479 | 8.35E-20 | Glioma |
| 347252 | IGFBPL1 | -1.918708646 | 4.83E-13 | Glioma |
| 9480 | ONECUT2 | -1.916443039 | 3.09E-21 | Glioma |
| 6440 | SFTPC | -1.91605545 | 1.29E-20 | Glioma |
| 8447 | DOC2B | -1.914877462 | 5.50E-20 | Glioma |
| 100128977 | None | -1.914771558 | 5.63E-28 | Glioma |
| 57497 | LRFN2 | -1.913073191 | 2.29E-18 | Glioma |
| 645974 | PABPC1L2B | -1.912254627 | 2.87E-13 | Glioma |
| 29118 | DDX25 | -1.906176217 | 7.36E-22 | Glioma |
| 40 | ASIC2 | -1.905076751 | 8.10E-13 | Glioma |
| 6579 | SLCO1A2 | -1.904864647 | 2.40E-19 | Glioma |
| 25830 | SULT4A1 | -1.903436274 | 1.11E-09 | Glioma |
| 4842 | NOS1 | -1.900293377 | 1.69E-13 | Glioma |
| 6327 | SCN2B | -1.898977192 | 1.96E-23 | Glioma |
| 29798 | None | -1.894592184 | 1.04E-39 | Glioma |
| 22849 | CPEB3 | -1.894439373 | 3.85E-58 | Glioma |
| 255426 | RASGEF1C | -1.893243559 | 6.97E-28 | Glioma |
| 577 | ADGRB3 | -1.887862098 | 3.29E-49 | Glioma |
| 1132 | CHRM4 | -1.8827366 | 3.95E-28 | Glioma |
| 81796 | SLCO5A1 | -1.881370653 | 3.00E-23 | Glioma |
| 79875 | THSD4 | -1.877756678 | 1.08E-33 | Glioma |
| 63827 | BCAN | -1.877640939 | 2.38E-27 | Glioma |
| 887 | CCKBR | -1.877549635 | 2.22E-11 | Glioma |
| 388419 | BTBD17 | -1.876925229 | 1.07E-19 | Glioma |
| 8707 | B3GALT2 | -1.87586972 | 1.58E-35 | Glioma |
| 139065 | SLITRK4 | -1.873595592 | 1.00E-13 | Glioma |
| 9808 | None | -1.873556561 | 8.60E-22 | Glioma |
| 23026 | MYO16 | -1.873219331 | 2.14E-28 | Glioma |
| 83992 | CTTNBP2 | -1.870341667 | 1.69E-50 | Glioma |
| 11075 | STMN2 | -1.869254382 | 1.36E-10 | Glioma |
| 100170841 | EPOP | -1.869216039 | 2.47E-59 | Glioma |
| 10501 | SEMA6B | -1.86911644 | 1.37E-46 | Glioma |
| 100033416 | None | -1.867944709 | 2.12E-17 | Glioma |
| 5156 | PDGFRA | -1.862612715 | 5.03E-21 | Glioma |
| 100133545 | None | -1.859093404 | 1.62E-26 | Glioma |
| 815 | CAMK2A | -1.858429053 | 7.03E-10 | Glioma |
| 10188 | TNK2 | -1.858266234 | 2.29E-50 | Glioma |
| 65989 | DLK2 | -1.85463508 | 5.03E-25 | Glioma |
| 79623 | GALNT14 | -1.854095163 | 3.90E-27 | Glioma |
| 116448 | OLIG1 | -1.854021218 | 8.28E-27 | Glioma |
| 55553 | SOX6 | -1.853932235 | 2.81E-44 | Glioma |
| 219539 | YPEL4 | -1.853416827 | 1.49E-37 | Glioma |
| 114800 | CCDC85A | -1.850947719 | 1.80E-26 | Glioma |
| 10409 | BASP1 | -1.850137204 | 1.53E-44 | Glioma |
| 6252 | RTN1 | -1.85000131 | 4.00E-37 | Glioma |
| 148979 | GLIS1 | -1.849021534 | 1.36E-17 | Glioma |
| 339761 | CYP27C1 | -1.84793878 | 6.63E-21 | Glioma |
| 2572 | GAD2 | -1.846869333 | 1.33E-09 | Glioma |
| 2557 | GABRA4 | -1.846231598 | 1.33E-09 | Glioma |
| 220965 | FAM13C | -1.845422934 | 8.61E-55 | Glioma |
| 286 | ANK1 | -1.843542599 | 7.80E-24 | Glioma |
| 9026 | HIP1R | -1.843221323 | 8.89E-41 | Glioma |
| 57718 | PPP4R4 | -1.838518239 | 1.55E-12 | Glioma |
| 375704 | ENHO | -1.837500232 | 4.96E-31 | Glioma |
| 6855 | SYP | -1.836363277 | 5.10E-33 | Glioma |
| 6304 | SATB1 | -1.831983582 | 1.15E-62 | Glioma |
| 51127 | TRIM17 | -1.831092086 | 4.33E-28 | Glioma |
| 5522 | PPP2R2C | -1.829825817 | 4.00E-14 | Glioma |
| 8822 | FGF17 | -1.826809281 | 1.05E-15 | Glioma |
| 55022 | PID1 | -1.826766234 | 1.45E-54 | Glioma |
| 5816 | PVALB | -1.826254143 | 2.75E-10 | Glioma |
| 375612 | LHFPL3 | -1.824579828 | 1.13E-16 | Glioma |
| 85407 | NKD1 | -1.823482897 | 8.79E-39 | Glioma |
| 387036 | None | -1.822036233 | 8.63E-35 | Glioma |
| 84870 | RSPO3 | -1.821683417 | 2.13E-14 | Glioma |
| 4974 | OMG | -1.821265356 | 4.86E-35 | Glioma |
| 10129 | FRY | -1.81957846 | 1.60E-68 | Glioma |
| 200407 | CREG2 | -1.819347872 | 5.58E-10 | Glioma |
| 7757 | ZNF208 | -1.818825336 | 1.50E-12 | Glioma |
| 284521 | OR2L13 | -1.81300048 | 1.59E-14 | Glioma |
| 6792 | CDKL5 | -1.809680004 | 7.53E-16 | Glioma |
| 130399 | ACVR1C | -1.809428431 | 3.09E-18 | Glioma |
| 6623 | SNCG | -1.807874657 | 1.83E-17 | Glioma |
| 57687 | VAT1L | -1.805097858 | 1.05E-18 | Glioma |
| 154790 | CLEC2L | -1.804123396 | 1.36E-10 | Glioma |
| 6334 | SCN8A | -1.80290139 | 3.41E-26 | Glioma |
| 57595 | PDZD4 | -1.802045188 | 1.43E-44 | Glioma |
| 29091 | STXBP6 | -1.801231355 | 7.03E-25 | Glioma |
| 23542 | MAPK8IP2 | -1.798947833 | 1.17E-39 | Glioma |
| 146206 | CARMIL2 | -1.797237535 | 3.62E-18 | Glioma |
| 79933 | SYNPO2L | -1.796549777 | 8.29E-29 | Glioma |
| 9481 | SLC25A27 | -1.792111533 | 4.02E-36 | Glioma |
| 9899 | SV2B | -1.791724764 | 1.34E-08 | Glioma |
| 8641 | PCDHGB4 | -1.791384267 | 4.94E-15 | Glioma |
| 148753 | FAM163A | -1.789001937 | 9.97E-15 | Glioma |
| 64084 | CLSTN2 | -1.788504274 | 5.42E-29 | Glioma |
| 441818 | None | -1.788234597 | 1.18E-35 | Glioma |
| 283174 | None | -1.788184373 | 3.00E-32 | Glioma |
| 84206 | MEX3B | -1.787790472 | 1.29E-38 | Glioma |
| 84059 | ADGRV1 | -1.786648976 | 3.96E-23 | Glioma |
| 219287 | AMER2 | -1.786633939 | 2.22E-35 | Glioma |
| 348254 | None | -1.783260185 | 1.36E-22 | Glioma |
| 4675 | NAP1L3 | -1.780417388 | 8.59E-66 | Glioma |
| 84440 | RAB11FIP4 | -1.779704498 | 7.95E-32 | Glioma |
| 284697 | BTBD8 | -1.779371792 | 9.82E-22 | Glioma |
| 10888 | GPR83 | -1.77912278 | 2.51E-13 | Glioma |
| 256987 | SERINC5 | -1.778536929 | 6.44E-28 | Glioma |
| 23017 | FAIM2 | -1.778484881 | 1.40E-42 | Glioma |
| 64101 | LRRC4 | -1.776380751 | 1.06E-56 | Glioma |
| 79856 | SNX22 | -1.776371527 | 7.08E-27 | Glioma |
| 79722 | ANKRD55 | -1.775500799 | 7.27E-17 | Glioma |
| 4674 | NAP1L2 | -1.775310463 | 4.46E-28 | Glioma |
| 3746 | KCNC1 | -1.772552896 | 5.25E-33 | Glioma |
| 100038246 | None | -1.768549004 | 2.87E-13 | Glioma |
| 55079 | FEZF2 | -1.768310257 | 1.37E-14 | Glioma |
| 140733 | MACROD2 | -1.766773201 | 1.95E-24 | Glioma |
| 5727 | PTCH1 | -1.763736457 | 5.21E-39 | Glioma |
| 10276 | NET1 | -1.762436522 | 1.01E-48 | Glioma |
| 5864 | RAB3A | -1.762236289 | 3.19E-25 | Glioma |
| 118427 | OLFM3 | -1.76021334 | 3.97E-10 | Glioma |
| 645455 | None | -1.76004932 | 1.66E-31 | Glioma |
| 653553 | None | -1.759674425 | 1.12E-10 | Glioma |
| 5581 | PRKCE | -1.75935835 | 1.87E-59 | Glioma |
| 93 | ACVR2B | -1.757860844 | 1.49E-71 | Glioma |
| 57476 | GRAMD1B | -1.75750251 | 4.47E-35 | Glioma |
| 4093 | SMAD9 | -1.757197223 | 2.08E-32 | Glioma |
| 1600 | DAB1 | -1.756647155 | 1.26E-17 | Glioma |
| 374864 | CCDC178 | -1.75281066 | 4.00E-14 | Glioma |
| 653308 | ASAH2B | -1.751047992 | 1.54E-22 | Glioma |
| 57369 | GJD2 | -1.749714105 | 1.19E-15 | Glioma |
| 2845 | GPR22 | -1.749460098 | 2.81E-10 | Glioma |
| 30837 | SOCS7 | -1.749454227 | 2.75E-29 | Glioma |
| 114134 | SLC2A13 | -1.749032824 | 4.12E-42 | Glioma |
| 114792 | KLHL32 | -1.74695234 | 6.39E-23 | Glioma |
| 192668 | CYS1 | -1.744290006 | 1.71E-25 | Glioma |
| 11076 | TPPP | -1.743234117 | 2.58E-20 | Glioma |
| 57156 | TMEM63C | -1.742686991 | 4.23E-35 | Glioma |
| 27445 | PCLO | -1.741924851 | 2.93E-14 | Glioma |
| 151742 | PPM1L | -1.739756862 | 1.85E-41 | Glioma |
| 53353 | LRP1B | -1.737855928 | 1.06E-37 | Glioma |
| 3785 | KCNQ2 | -1.735821241 | 3.15E-20 | Glioma |
| 389941 | C1QL3 | -1.735721231 | 2.14E-08 | Glioma |
| 1821 | DRP2 | -1.735426499 | 1.35E-40 | Glioma |
| 4747 | NEFL | -1.735259354 | 5.29E-07 | Glioma |
| 23281 | MTUS2 | -1.732795536 | 2.91E-16 | Glioma |
| 56341 | PRMT8 | -1.724239869 | 2.44E-11 | Glioma |
| 793 | CALB1 | -1.72391902 | 6.57E-12 | Glioma |
| 132204 | SYNPR | -1.723915938 | 9.19E-08 | Glioma |
| 139221 | PWWP3B | -1.723356647 | 3.11E-15 | Glioma |
| 5046 | PCSK6 | -1.722416051 | 1.89E-16 | Glioma |
| 288 | ANK3 | -1.722389192 | 2.97E-21 | Glioma |
| 51764 | GNG13 | -1.720464351 | 2.42E-14 | Glioma |
| 2066 | ERBB4 | -1.718473222 | 7.62E-21 | Glioma |
| 11086 | ADAM29 | -1.71607319 | 5.28E-28 | Glioma |
| 220972 | Mar-08 | -1.715432868 | 6.05E-42 | Glioma |
| 148641 | SLC35F3 | -1.714123402 | 3.52E-14 | Glioma |
| 56112 | PCDHGA3 | -1.712934624 | 9.44E-16 | Glioma |
| 9625 | AATK | -1.712633416 | 1.53E-24 | Glioma |
| 284805 | C20orf203 | -1.711892489 | 6.67E-28 | Glioma |
| 55502 | HES6 | -1.711397661 | 5.31E-29 | Glioma |
| 196500 | PIANP | -1.710710062 | 5.44E-37 | Glioma |
| 56146 | PCDHA2 | -1.710423761 | 3.73E-15 | Glioma |
| 27092 | CACNG4 | -1.708716424 | 8.07E-21 | Glioma |
| 6326 | SCN2A | -1.707999638 | 9.23E-29 | Glioma |
| 27112 | FAM155B | -1.707366263 | 1.60E-22 | Glioma |
| 400940 | None | -1.705767164 | 2.03E-23 | Glioma |
| 55117 | SLC6A15 | -1.705741147 | 1.91E-10 | Glioma |
| 9760 | TOX | -1.703365116 | 4.25E-34 | Glioma |
| 2668 | GDNF | -1.70071107 | 4.14E-25 | Glioma |
| 57484 | RNF150 | -1.698973875 | 5.41E-41 | Glioma |
| 284427 | SLC25A41 | -1.696187267 | 6.43E-29 | Glioma |
| 402117 | VWC2L | -1.695462171 | 4.33E-15 | Glioma |
| 547 | KIF1A | -1.694880194 | 1.26E-30 | Glioma |
| 9127 | P2RX6 | -1.693239117 | 2.16E-27 | Glioma |
| 140862 | ISM1 | -1.69238282 | 3.11E-21 | Glioma |
| 55966 | AJAP1 | -1.690630165 | 1.17E-20 | Glioma |
| 85445 | CNTNAP4 | -1.689242777 | 1.52E-10 | Glioma |
| 7008 | TEF | -1.689034158 | 1.47E-51 | Glioma |
| 402381 | SOHLH1 | -1.68891662 | 3.29E-10 | Glioma |
| 143282 | FGFBP3 | -1.687428568 | 1.14E-33 | Glioma |
| 9244 | CRLF1 | -1.686041062 | 7.77E-09 | Glioma |
| 346007 | EYS | -1.685629584 | 8.51E-35 | Glioma |
| 554235 | ASPDH | -1.68550311 | 2.18E-18 | Glioma |
| 84867 | PTPN5 | -1.684072285 | 2.72E-09 | Glioma |
| 9472 | AKAP6 | -1.684069298 | 2.35E-50 | Glioma |
| 9515 | STXBP5L | -1.683792176 | 1.89E-14 | Glioma |
| 284069 | FAM171A2 | -1.68212072 | 9.85E-49 | Glioma |
| 127833 | SYT2 | -1.681956785 | 4.63E-13 | Glioma |
| 162494 | RHBDL3 | -1.68127995 | 1.34E-34 | Glioma |
| 6545 | SLC7A4 | -1.679172944 | 4.12E-14 | Glioma |
| 53616 | ADAM22 | -1.678101322 | 1.11E-36 | Glioma |
| 10151 | None | -1.677217803 | 2.85E-33 | Glioma |
| 167681 | PRSS35 | -1.670819736 | 2.75E-16 | Glioma |
| 22914 | KLRK1 | -1.668538257 | 1.98E-17 | Glioma |
| 57692 | MAGEE1 | -1.668315687 | 4.25E-31 | Glioma |
| 230 | ALDOC | -1.667104136 | 9.27E-26 | Glioma |
| 79822 | ARHGAP28 | -1.666714148 | 2.00E-21 | Glioma |
| 375057 | STUM | -1.665911696 | 1.80E-19 | Glioma |
| 1428 | CRYM | -1.664275629 | 3.20E-07 | Glioma |
| 10669 | CGREF1 | -1.662000849 | 1.34E-30 | Glioma |
| 23145 | SSPO | -1.659893811 | 6.10E-17 | Glioma |
| 51421 | AMOTL2 | -1.659642796 | 4.97E-48 | Glioma |
| 1740 | DLG2 | -1.659260882 | 4.02E-32 | Glioma |
| 259217 | HSPA12A | -1.657674184 | 5.50E-34 | Glioma |
| 140597 | TCEAL2 | -1.656820696 | 3.08E-27 | Glioma |
| 6753 | SSTR3 | -1.656598636 | 2.69E-13 | Glioma |
| 491 | ATP2B2 | -1.655354458 | 2.37E-25 | Glioma |
| 22989 | MYH15 | -1.654720468 | 5.30E-18 | Glioma |
| 56899 | ANKS1B | -1.654547613 | 3.62E-21 | Glioma |
| 8936 | WASF1 | -1.653656468 | 2.39E-51 | Glioma |
| 10716 | TBR1 | -1.649032713 | 1.67E-08 | Glioma |
| 1404 | HAPLN1 | -1.646532351 | 8.34E-15 | Glioma |
| 282973 | JAKMIP3 | -1.645026877 | 7.21E-24 | Glioma |
| 56660 | KCNK12 | -1.644515623 | 1.78E-17 | Glioma |
| 56978 | PRDM8 | -1.642962981 | 1.85E-17 | Glioma |
| 6812 | STXBP1 | -1.642091325 | 2.21E-38 | Glioma |
| 26045 | LRRTM2 | -1.639898957 | 2.22E-33 | Glioma |
| 2905 | GRIN2C | -1.63862453 | 8.13E-18 | Glioma |
| 414149 | ACBD7 | -1.638553184 | 1.13E-18 | Glioma |
| 55636 | CHD7 | -1.636957532 | 6.36E-42 | Glioma |
| 400591 | C17orf102 | -1.635600109 | 1.40E-16 | Glioma |
| 59353 | TMEM35A | -1.635331792 | 4.87E-40 | Glioma |
| 84553 | FAXC | -1.633450105 | 2.12E-53 | Glioma |
| 10361 | NPM2 | -1.631372186 | 3.07E-11 | Glioma |
| 4613 | MYCN | -1.627122059 | 1.02E-27 | Glioma |
| 387104 | SOGA3 | -1.626460152 | 1.95E-47 | Glioma |
| 81551 | STMN4 | -1.626287173 | 1.99E-22 | Glioma |
| 84458 | LCOR | -1.625998634 | 2.25E-28 | Glioma |
| 3899 | AFF3 | -1.625809684 | 2.67E-35 | Glioma |
| 6323 | SCN1A | -1.624173441 | 7.21E-29 | Glioma |
| 116173 | CMTM5 | -1.623132784 | 3.06E-27 | Glioma |
| 57469 | PNMA8B | -1.622758941 | 1.76E-30 | Glioma |
| 56142 | PCDHA6 | -1.622246939 | 5.41E-11 | Glioma |
| 5334 | PLCL1 | -1.621611054 | 4.79E-37 | Glioma |
| 55799 | CACNA2D3 | -1.619411277 | 1.61E-12 | Glioma |
| 9379 | NRXN2 | -1.618157033 | 4.44E-41 | Glioma |
| 1006 | CDH8 | -1.617900206 | 3.27E-11 | Glioma |
| 5579 | PRKCB | -1.615486153 | 1.30E-18 | Glioma |
| 5590 | PRKCZ | -1.614177069 | 2.50E-26 | Glioma |
| 375607 | NAT16 | -1.613412067 | 9.77E-15 | Glioma |
| 23522 | KAT6B | -1.610968832 | 1.48E-69 | Glioma |
| 9514 | GAL3ST1 | -1.610865652 | 5.19E-21 | Glioma |
| 5650 | KLK7 | -1.610732929 | 7.42E-09 | Glioma |
| 85417 | CCNB3 | -1.604148558 | 6.19E-34 | Glioma |
| 50832 | TAS2R4 | -1.602253584 | 1.61E-36 | Glioma |
| 147660 | ZNF578 | -1.599975973 | 1.45E-22 | Glioma |
| 5818 | NECTIN1 | -1.598390263 | 3.08E-48 | Glioma |
| 6144 | RPL21 | -1.598313695 | 8.41E-21 | Glioma |
| 7222 | TRPC3 | -1.598069048 | 1.22E-25 | Glioma |
| 5746 | PTH2R | -1.597844095 | 1.19E-13 | Glioma |
| 56113 | PCDHGA2 | -1.596810439 | 8.12E-17 | Glioma |
| 151009 | None | -1.59560148 | 1.98E-09 | Glioma |
| 7881 | KCNAB1 | -1.591293167 | 5.36E-25 | Glioma |
| 25806 | VAX2 | -1.591279645 | 4.81E-27 | Glioma |
| 9148 | NEURL1 | -1.59085383 | 3.64E-13 | Glioma |
| 8715 | NOL4 | -1.589422163 | 7.12E-40 | Glioma |
| 27439 | TMEM121B | -1.586238846 | 2.49E-54 | Glioma |
| 3049 | HBQ1 | -1.585369091 | 2.23E-18 | Glioma |
| 10382 | TUBB4A | -1.583762335 | 9.45E-19 | Glioma |
| 6529 | SLC6A1 | -1.583002279 | 2.29E-30 | Glioma |
| 29767 | TMOD2 | -1.582563093 | 3.04E-54 | Glioma |
| 5332 | PLCB4 | -1.581122179 | 2.14E-30 | Glioma |
| 80339 | PNPLA3 | -1.580933518 | 8.67E-17 | Glioma |
| 53826 | FXYD6 | -1.579154885 | 1.84E-40 | Glioma |
| 41 | ASIC1 | -1.578772017 | 5.44E-37 | Glioma |
| 23305 | ACSL6 | -1.578247012 | 4.64E-24 | Glioma |
| 9363 | RAB33A | -1.576914053 | 2.04E-29 | Glioma |
| 4692 | NDN | -1.576759532 | 1.09E-21 | Glioma |
| 84539 | MCHR2 | -1.575805604 | 1.66E-10 | Glioma |
| 7681 | MKRN3 | -1.575593133 | 6.14E-34 | Glioma |
| 161725 | OTUD7A | -1.575476479 | 8.46E-28 | Glioma |
| 138046 | RALYL | -1.575386062 | 2.60E-12 | Glioma |
| 390429 | None | -1.575073414 | 2.60E-09 | Glioma |
| 25858 | CATSPERZ | -1.572479955 | 8.59E-19 | Glioma |
| 392862 | GRID2IP | -1.572153122 | 1.54E-27 | Glioma |
| 619279 | ZNF704 | -1.572099141 | 3.68E-34 | Glioma |
| 4745 | NELL1 | -1.572073681 | 4.73E-08 | Glioma |
| 5241 | PGR | -1.572042275 | 5.82E-16 | Glioma |
| 54551 | MAGEL2 | -1.571362613 | 6.93E-27 | Glioma |
| 160851 | DGKH | -1.569263627 | 5.34E-26 | Glioma |
| 55806 | HR | -1.567636293 | 5.32E-27 | Glioma |
| 29114 | TAGLN3 | -1.567432693 | 4.18E-18 | Glioma |
| 219736 | STOX1 | -1.567421902 | 4.10E-21 | Glioma |
| 2770 | GNAI1 | -1.567362396 | 7.74E-40 | Glioma |
| 1135 | CHRNA2 | -1.56448313 | 2.88E-09 | Glioma |
| 23349 | PHF24 | -1.564279731 | 3.09E-10 | Glioma |
| 51440 | HPCAL4 | -1.564218354 | 5.46E-12 | Glioma |
| 114787 | GPRIN1 | -1.562939772 | 6.81E-36 | Glioma |
| 57462 | MYORG | -1.561735633 | 1.03E-35 | Glioma |
| 7224 | TRPC5 | -1.560517302 | 1.86E-12 | Glioma |
| 50512 | PODXL2 | -1.55988083 | 2.46E-46 | Glioma |
| 613212 | CTXN3 | -1.558606666 | 2.95E-09 | Glioma |
| 390616 | ANKRD34C | -1.557949427 | 7.02E-12 | Glioma |
| 84197 | POMK | -1.55785438 | 3.17E-30 | Glioma |
| 56521 | DNAJC12 | -1.556982611 | 1.69E-52 | Glioma |
| 1012 | CDH13 | -1.556363811 | 6.42E-21 | Glioma |
| 116986 | AGAP2 | -1.554191302 | 5.89E-19 | Glioma |
| 2894 | GRID1 | -1.553351648 | 5.75E-45 | Glioma |
| 159195 | USP54 | -1.552674576 | 2.32E-44 | Glioma |
| 148213 | ZNF681 | -1.551592466 | 3.16E-35 | Glioma |
| 25758 | KIAA1549L | -1.550704274 | 6.46E-30 | Glioma |
| 645683 | None | -1.550453431 | 7.78E-24 | Glioma |
| 3797 | KIF3C | -1.550352716 | 1.13E-52 | Glioma |
| 349667 | RTN4RL2 | -1.549467277 | 1.08E-22 | Glioma |
| 26960 | NBEA | -1.548985783 | 2.97E-44 | Glioma |
| 22866 | CNKSR2 | -1.545190101 | 7.07E-25 | Glioma |
| 6139 | RPL17 | -1.544532822 | 3.60E-41 | Glioma |
| 1795 | DOCK3 | -1.543935254 | 7.49E-27 | Glioma |
| 388228 | SBK1 | -1.54376005 | 1.04E-35 | Glioma |
| 55879 | GABRQ | -1.542846336 | 3.97E-12 | Glioma |
| 4685 | NCAM2 | -1.54163954 | 4.10E-31 | Glioma |
| 157638 | FAM84B | -1.541582226 | 1.60E-29 | Glioma |
| 50940 | PDE11A | -1.541360583 | 2.34E-18 | Glioma |
| 23285 | BTBD8 | -1.540853871 | 3.48E-31 | Glioma |
| 1995 | ELAVL3 | -1.540244886 | 5.46E-30 | Glioma |
| 6710 | SPTB | -1.540025563 | 1.18E-16 | Glioma |
| 5027 | P2RX7 | -1.539905645 | 4.16E-33 | Glioma |
| 7267 | TTC3 | -1.539705577 | 1.87E-77 | Glioma |
| 55607 | PPP1R9A | -1.539690637 | 1.05E-37 | Glioma |
| 387486 | None | -1.538211627 | 1.03E-13 | Glioma |
| 146760 | RTN4RL1 | -1.537897703 | 8.76E-12 | Glioma |
| 146664 | MGAT5B | -1.536857256 | 2.04E-22 | Glioma |
| 10570 | DPYSL4 | -1.533783067 | 2.17E-40 | Glioma |
| 284047 | None | -1.53345314 | 1.15E-14 | Glioma |
| 128414 | NKAIN4 | -1.533325959 | 9.93E-16 | Glioma |
| 9911 | TMCC2 | -1.533181642 | 2.46E-38 | Glioma |
| 5911 | RAP2A | -1.532152922 | 2.04E-66 | Glioma |
| 117 | ADCYAP1R1 | -1.531387335 | 4.74E-16 | Glioma |
| 3362 | HTR6 | -1.530263343 | 4.80E-16 | Glioma |
| 9145 | SYNGR1 | -1.530149984 | 3.73E-40 | Glioma |
| 1408 | CRY2 | -1.529098135 | 1.17E-51 | Glioma |
| 885 | CCK | -1.527820402 | 2.79E-06 | Glioma |
| 57419 | SLC24A3 | -1.525049108 | 4.92E-27 | Glioma |
| 1543 | CYP1A1 | -1.523827632 | 1.75E-20 | Glioma |
| 2775 | GNAO1 | -1.523379008 | 1.22E-37 | Glioma |
| 57699 | CPNE5 | -1.522837332 | 7.98E-22 | Glioma |
| 93377 | OPALIN | -1.522455623 | 8.91E-05 | Glioma |
| 4038 | LRP4 | -1.520463041 | 1.77E-34 | Glioma |
| 4761 | NEUROD2 | -1.520261059 | 6.18E-07 | Glioma |
| 53822 | FXYD7 | -1.519065308 | 1.45E-08 | Glioma |
| 345079 | SOWAHB | -1.518116537 | 1.01E-09 | Glioma |
| 121601 | ANO4 | -1.517317427 | 2.34E-13 | Glioma |
| 2786 | GNG4 | -1.516684164 | 1.07E-30 | Glioma |
| 6750 | SST | -1.516467602 | 2.19E-07 | Glioma |
| 6319 | SCD | -1.516080482 | 1.76E-36 | Glioma |
| 22852 | ANKRD26 | -1.515467649 | 3.59E-60 | Glioma |
| 4654 | MYOD1 | -1.512994492 | 1.53E-14 | Glioma |
| 9796 | PHYHIP | -1.512919203 | 3.32E-12 | Glioma |
| 1129 | CHRM2 | -1.512312164 | 1.46E-16 | Glioma |
| 285780 | None | -1.511372026 | 1.76E-08 | Glioma |
| 9143 | SYNGR3 | -1.510520875 | 8.92E-13 | Glioma |
| 2830 | GPR6 | -1.509150737 | 1.41E-11 | Glioma |
| 3780 | KCNN1 | -1.508980896 | 1.62E-19 | Glioma |
| 253260 | RICTOR | -1.506564429 | 3.55E-62 | Glioma |
| 5798 | PTPRN | -1.504495276 | 5.63E-11 | Glioma |
| 6659 | SOX4 | -1.503666529 | 6.28E-25 | Glioma |
| 340529 | PABPC1L2A | -1.502695218 | 1.87E-11 | Glioma |
| 56977 | STOX2 | -1.501676714 | 3.19E-32 | Glioma |
| 203190 | LGI3 | -1.501488454 | 1.04E-12 | Glioma |
| 55859 | BEX1 | -1.501484208 | 2.70E-29 | Glioma |
| 9720 | CCDC144A | -1.501333765 | 6.62E-19 | Glioma |
| 1271 | CNTFR | -1.501115955 | 5.37E-23 | Glioma |
| 489 | ATP2A3 | 1.500898367 | 3.81E-30 | Glioma |
| 840 | CASP7 | 1.500927088 | 1.82E-63 | Glioma |
| 60401 | EDA2R | 1.50131347 | 1.16E-11 | Glioma |
| 254887 | ZDHHC23 | 1.501999085 | 1.44E-14 | Glioma |
| 6464 | SHC1 | 1.504904044 | 2.88E-50 | Glioma |
| 339512 | CCDC190 | 1.504998974 | 3.10E-15 | Glioma |
| 26031 | OSBPL3 | 1.505968456 | 8.36E-40 | Glioma |
| 1827 | RCAN1 | 1.506282144 | 9.17E-39 | Glioma |
| 1438 | CSF2RA | 1.506419149 | 5.19E-29 | Glioma |
| 5738 | PTGFRN | 1.506653955 | 3.23E-39 | Glioma |
| 8544 | PIR | 1.507246858 | 1.17E-23 | Glioma |
| 124857 | WFIKKN2 | 1.507622638 | 3.77E-10 | Glioma |
| 1508 | CTSB | 1.509240719 | 1.12E-60 | Glioma |
| 79689 | STEAP4 | 1.509470028 | 1.54E-17 | Glioma |
| 4051 | CYP4F3 | 1.510009739 | 1.60E-10 | Glioma |
| 158787 | RIBC1 | 1.510261 | 7.62E-23 | Glioma |
| 55789 | DEPDC1B | 1.510300326 | 3.44E-15 | Glioma |
| 10723 | SLC12A7 | 1.510467464 | 2.03E-63 | Glioma |
| 5308 | PITX2 | 1.510700091 | 3.97E-09 | Glioma |
| 64778 | FNDC3B | 1.511145484 | 7.69E-57 | Glioma |
| 3603 | IL16 | 1.5122143 | 6.51E-52 | Glioma |
| 100130613 | PRR32 | 1.512266066 | 3.84E-12 | Glioma |
| 1999 | ELF3 | 1.512444671 | 1.51E-20 | Glioma |
| 80830 | APOL6 | 1.513042026 | 4.83E-45 | Glioma |
| 81704 | DOCK8 | 1.513572481 | 8.97E-33 | Glioma |
| 3004 | GZMM | 1.513759297 | 2.99E-27 | Glioma |
| 154865 | IQUB | 1.513766616 | 3.20E-23 | Glioma |
| 55858 | TMEM165 | 1.514623003 | 3.06E-65 | Glioma |
| 9156 | EXO1 | 1.514729719 | 9.44E-14 | Glioma |
| 389 | RHOC | 1.515041769 | 2.06E-56 | Glioma |
| 7791 | ZYX | 1.515295831 | 4.62E-56 | Glioma |
| 51203 | NUSAP1 | 1.515508393 | 6.53E-14 | Glioma |
| 360132 | None | 1.515545128 | 1.45E-25 | Glioma |
| 174 | AFP | 1.515849243 | 3.57E-16 | Glioma |
| 5358 | PLS3 | 1.516004609 | 1.54E-45 | Glioma |
| 3219 | HOXB9 | 1.518538925 | 8.97E-14 | Glioma |
| 55020 | TTC38 | 1.518882128 | 3.84E-50 | Glioma |
| 80896 | NPL | 1.5190788 | 1.99E-46 | Glioma |
| 84818 | IL17RC | 1.51917338 | 2.67E-44 | Glioma |
| 200315 | APOBEC3A | 1.520146551 | 4.89E-22 | Glioma |
| 2006 | ELN | 1.520342129 | 7.20E-37 | Glioma |
| 85301 | COL27A1 | 1.520358389 | 6.22E-16 | Glioma |
| 1471 | CST3 | 1.5209318 | 5.98E-33 | Glioma |
| 1848 | DUSP6 | 1.522835732 | 4.98E-30 | Glioma |
| 80336 | PABPC1L | 1.523459347 | 9.80E-24 | Glioma |
| 4005 | LMO2 | 1.524805272 | 1.32E-49 | Glioma |
| 729 | C6 | 1.525344421 | 1.25E-11 | Glioma |
| 8717 | TRADD | 1.525674661 | 3.33E-69 | Glioma |
| 8843 | HCAR3 | 1.525757876 | 8.53E-21 | Glioma |
| 7643 | ZNF90 | 1.527956887 | 1.27E-24 | Glioma |
| 3489 | IGFBP6 | 1.528742025 | 2.61E-17 | Glioma |
| 388335 | TMEM220 | 1.528983923 | 7.18E-45 | Glioma |
| 85474 | LBX2 | 1.529465181 | 4.22E-21 | Glioma |
| 2004 | ELK3 | 1.52950099 | 1.24E-41 | Glioma |
| 144406 | WDR66 | 1.529508578 | 1.88E-22 | Glioma |
| 647309 | GMNC | 1.530155341 | 2.62E-10 | Glioma |
| 222235 | FBXL13 | 1.530417145 | 4.78E-30 | Glioma |
| 200373 | CFAP221 | 1.530563586 | 4.12E-14 | Glioma |
| 202243 | CCDC125 | 1.530851565 | 4.09E-51 | Glioma |
| 81035 | COLEC12 | 1.531447306 | 1.23E-25 | Glioma |
| 54863 | TOR4A | 1.531543328 | 1.20E-53 | Glioma |
| 5165 | PDK3 | 1.531644239 | 1.37E-39 | Glioma |
| 163351 | GBP6 | 1.532197233 | 4.27E-19 | Glioma |
| 6367 | CCL22 | 1.532357597 | 5.12E-21 | Glioma |
| 2306 | FOXD2 | 1.532790477 | 1.38E-19 | Glioma |
| 57538 | ALPK3 | 1.532807813 | 1.29E-43 | Glioma |
| 5330 | PLCB2 | 1.53300076 | 1.71E-38 | Glioma |
| 312 | ANXA13 | 1.53360109 | 3.32E-18 | Glioma |
| 1017 | CDK2 | 1.533702557 | 1.57E-36 | Glioma |
| 116328 | C8orf34 | 1.533757805 | 1.91E-10 | Glioma |
| 373861 | None | 1.53432323 | 3.04E-10 | Glioma |
| 7153 | TOP2A | 1.534368923 | 1.20E-08 | Glioma |
| 1266 | CNN3 | 1.534563769 | 1.37E-54 | Glioma |
| 150223 | YDJC | 1.534730508 | 3.13E-30 | Glioma |
| 3142 | HLX | 1.535152507 | 2.34E-42 | Glioma |
| 203100 | HTRA4 | 1.535234033 | 9.61E-21 | Glioma |
| 286827 | TRIM59 | 1.535909177 | 2.46E-19 | Glioma |
| 3959 | LGALS3BP | 1.536955917 | 7.72E-61 | Glioma |
| 246176 | GAS2L2 | 1.539397502 | 3.26E-11 | Glioma |
| 1757 | SARDH | 1.539824273 | 5.44E-20 | Glioma |
| 2990 | GUSB | 1.540060232 | 1.45E-72 | Glioma |
| 151636 | DTX3L | 1.540114012 | 2.64E-62 | Glioma |
| 11173 | ADAMTS7 | 1.54018339 | 3.47E-18 | Glioma |
| 9249 | DHRS3 | 1.54031084 | 2.26E-33 | Glioma |
| 55303 | GIMAP4 | 1.540620634 | 1.32E-52 | Glioma |
| 93082 | NEURL3 | 1.540836879 | 1.34E-23 | Glioma |
| 23333 | DPY19L1 | 1.541375069 | 1.94E-57 | Glioma |
| 80346 | REEP4 | 1.541703235 | 9.81E-68 | Glioma |
| 3233 | HOXD4 | 1.542181508 | 1.38E-10 | Glioma |
| 8519 | IFITM1 | 1.542971883 | 3.25E-26 | Glioma |
| 2268 | FGR | 1.543031418 | 2.35E-55 | Glioma |
| 221472 | FGD2 | 1.543481391 | 8.29E-39 | Glioma |
| 3645 | INSRR | 1.543599211 | 2.05E-13 | Glioma |
| 343263 | MYBPHL | 1.543976274 | 4.09E-19 | Glioma |
| 8553 | BHLHE40 | 1.544356113 | 6.44E-34 | Glioma |
| 654433 | None | 1.544825812 | 1.18E-08 | Glioma |
| 63926 | ANKEF1 | 1.545263893 | 2.57E-17 | Glioma |
| 1959 | EGR2 | 1.547217551 | 6.13E-17 | Glioma |
| 10333 | TLR6 | 1.547524326 | 2.05E-30 | Glioma |
| 445582 | POTEE | 1.54774588 | 5.65E-23 | Glioma |
| 399669 | None | 1.547784181 | 2.31E-47 | Glioma |
| 54718 | None | 1.5480539 | 4.52E-36 | Glioma |
| 118429 | ANTXR2 | 1.5494406 | 8.25E-47 | Glioma |
| 10223 | GPA33 | 1.54954186 | 3.29E-22 | Glioma |
| 8856 | NR1I2 | 1.550731434 | 1.52E-31 | Glioma |
| 83854 | ANGPTL6 | 1.551052876 | 6.89E-26 | Glioma |
| 3161 | HMMR | 1.551330343 | 7.70E-15 | Glioma |
| 200010 | SLC5A9 | 1.551352494 | 2.40E-27 | Glioma |
| 55211 | DPPA4 | 1.551413388 | 1.56E-20 | Glioma |
| 57705 | WDFY4 | 1.552509712 | 1.02E-29 | Glioma |
| 151313 | FAHD2B | 1.553245496 | 1.81E-21 | Glioma |
| 1062 | CENPE | 1.554766076 | 4.41E-17 | Glioma |
| 149685 | ADIG | 1.555128017 | 4.62E-20 | Glioma |
| 10783 | NEK6 | 1.556067607 | 8.96E-52 | Glioma |
| 3133 | HLA-E | 1.556453926 | 1.79E-69 | Glioma |
| 3635 | INPP5D | 1.556724996 | 5.38E-50 | Glioma |
| 6820 | SULT2B1 | 1.556770351 | 9.03E-22 | Glioma |
| 284013 | VMO1 | 1.556979318 | 8.76E-48 | Glioma |
| 728358 | DEFA1B | 1.55781956 | 7.31E-12 | Glioma |
| 643008 | SMIM5 | 1.558164325 | 1.02E-26 | Glioma |
| 341 | APOC1 | 1.558372856 | 1.91E-31 | Glioma |
| 440101 | None | 1.559102566 | 2.49E-23 | Glioma |
| 4825 | NKX6-1 | 1.559387655 | 1.73E-16 | Glioma |
| 926 | CD8B | 1.559843366 | 3.27E-23 | Glioma |
| 55200 | PLEKHG6 | 1.560963927 | 9.37E-26 | Glioma |
| 2517 | FUCA1 | 1.56138225 | 1.55E-66 | Glioma |
| 5145 | PDE6A | 1.561789157 | 8.41E-21 | Glioma |
| 80320 | SP6 | 1.561865396 | 5.32E-30 | Glioma |
| 2316 | FLNA | 1.562007401 | 2.23E-45 | Glioma |
| 79022 | TMEM106C | 1.562229943 | 9.99E-51 | Glioma |
| 54898 | ELOVL2 | 1.563242022 | 3.68E-15 | Glioma |
| 150365 | MEI1 | 1.563957504 | 5.85E-32 | Glioma |
| 6913 | TBX15 | 1.564181292 | 6.01E-22 | Glioma |
| 53347 | UBASH3A | 1.564582351 | 1.19E-21 | Glioma |
| 400891 | LRRC74B | 1.565167449 | 1.21E-15 | Glioma |
| 9641 | IKBKE | 1.565884045 | 2.58E-53 | Glioma |
| 3074 | HEXB | 1.566630108 | 1.41E-75 | Glioma |
| 5627 | PROS1 | 1.56702457 | 6.06E-39 | Glioma |
| 55630 | SLC39A4 | 1.567461548 | 3.24E-24 | Glioma |
| 7033 | TFF3 | 1.567641503 | 8.38E-14 | Glioma |
| 8363 | HIST1H4J | 1.567858494 | 8.56E-20 | Glioma |
| 84215 | ZNF541 | 1.568127444 | 7.28E-19 | Glioma |
| 9173 | IL1RL1 | 1.568690384 | 6.08E-11 | Glioma |
| 3918 | LAMC2 | 1.56912507 | 2.41E-17 | Glioma |
| 9723 | SEMA3E | 1.569338902 | 6.24E-08 | Glioma |
| 7221 | None | 1.570367581 | 2.42E-29 | Glioma |
| 113115 | MTFR2 | 1.570783048 | 3.30E-24 | Glioma |
| 150468 | CKAP2L | 1.57093944 | 2.56E-12 | Glioma |
| 133690 | CAPSL | 1.571831459 | 3.18E-10 | Glioma |
| 653786 | None | 1.572495576 | 1.31E-18 | Glioma |
| 58985 | IL22RA1 | 1.572529626 | 2.81E-23 | Glioma |
| 129881 | CCDC173 | 1.572689182 | 8.49E-37 | Glioma |
| 22943 | DKK1 | 1.572916401 | 1.18E-08 | Glioma |
| 8444 | DYRK3 | 1.573076476 | 4.78E-55 | Glioma |
| 29109 | FHOD1 | 1.573085754 | 2.82E-66 | Glioma |
| 4210 | MEFV | 1.57433366 | 4.18E-33 | Glioma |
| 51083 | GAL | 1.574581341 | 3.50E-10 | Glioma |
| 9700 | ESPL1 | 1.574590633 | 1.55E-12 | Glioma |
| 6899 | TBX1 | 1.576038592 | 6.07E-15 | Glioma |
| 255809 | C19orf38 | 1.57742545 | 4.34E-42 | Glioma |
| 389058 | SP5 | 1.579038417 | 2.59E-17 | Glioma |
| 9938 | ARHGAP25 | 1.579098697 | 4.11E-60 | Glioma |
| 60681 | FKBP10 | 1.58015473 | 1.59E-38 | Glioma |
| 5360 | PLTP | 1.58030128 | 3.48E-43 | Glioma |
| 85360 | SYDE1 | 1.58105352 | 5.98E-59 | Glioma |
| 8809 | IL18R1 | 1.581432865 | 4.65E-20 | Glioma |
| 730 | C7 | 1.583079252 | 7.81E-12 | Glioma |
| 2022 | ENG | 1.583308846 | 4.31E-59 | Glioma |
| 3672 | ITGA1 | 1.583675428 | 4.64E-28 | Glioma |
| 8743 | TNFSF10 | 1.58372892 | 5.64E-35 | Glioma |
| 11339 | OIP5 | 1.585136232 | 1.65E-19 | Glioma |
| 161753 | ODF3L1 | 1.585293558 | 2.54E-30 | Glioma |
| 3087 | HHEX | 1.585325915 | 7.09E-48 | Glioma |
| 56964 | WDR93 | 1.585483111 | 1.78E-24 | Glioma |
| 6674 | SPAG1 | 1.585876227 | 1.43E-35 | Glioma |
| 85236 | HIST1H2BK | 1.586227402 | 9.82E-34 | Glioma |
| 100271835 | None | 1.586261307 | 7.79E-20 | Glioma |
| 5937 | RBMS1 | 1.588002453 | 3.70E-56 | Glioma |
| 64135 | IFIH1 | 1.588929167 | 1.20E-46 | Glioma |
| 5175 | PECAM1 | 1.589033217 | 9.20E-66 | Glioma |
| 10141 | None | 1.589659562 | 3.73E-18 | Glioma |
| 9322 | TRIP10 | 1.590022328 | 4.47E-52 | Glioma |
| 891 | CCNB1 | 1.590025097 | 4.18E-28 | Glioma |
| 3430 | IFI35 | 1.590187832 | 1.99E-53 | Glioma |
| 8208 | CHAF1B | 1.590385171 | 9.31E-26 | Glioma |
| 1824 | DSC2 | 1.591436777 | 4.21E-32 | Glioma |
| 9076 | CLDN1 | 1.59149174 | 4.20E-29 | Glioma |
| 9435 | CHST2 | 1.592014396 | 7.81E-47 | Glioma |
| 26292 | MYCBP | 1.592505087 | 3.11E-68 | Glioma |
| 8797 | TNFRSF10A | 1.592546888 | 2.68E-30 | Glioma |
| 9064 | MAP3K6 | 1.592993001 | 2.69E-57 | Glioma |
| 400410 | ST20 | 1.59385761 | 8.25E-35 | Glioma |
| 147138 | TMC8 | 1.593967078 | 5.89E-46 | Glioma |
| 114884 | OSBPL10 | 1.594664053 | 3.40E-25 | Glioma |
| 8029 | CUBN | 1.594926744 | 3.02E-39 | Glioma |
| 1358 | CPA2 | 1.595178693 | 1.66E-22 | Glioma |
| 63901 | FAM111A | 1.596593897 | 1.17E-57 | Glioma |
| 6237 | RRAS | 1.596896691 | 4.04E-60 | Glioma |
| 221468 | TMEM217 | 1.597386094 | 5.24E-51 | Glioma |
| 9260 | PDLIM7 | 1.598036478 | 1.92E-47 | Glioma |
| 730971 | None | 1.598279274 | 4.56E-30 | Glioma |
| 6383 | SDC2 | 1.598606795 | 1.46E-32 | Glioma |
| 26150 | RIBC2 | 1.598755932 | 7.88E-13 | Glioma |
| 100130771 | EFCAB10 | 1.599164296 | 9.72E-19 | Glioma |
| 84674 | CARD6 | 1.601437768 | 3.14E-55 | Glioma |
| 256227 | STEAP1B | 1.601725133 | 3.06E-20 | Glioma |
| 129607 | CMPK2 | 1.602469596 | 2.75E-36 | Glioma |
| 136306 | SVOPL | 1.602707097 | 7.74E-15 | Glioma |
| 35 | ACADS | 1.604508815 | 3.89E-39 | Glioma |
| 339778 | C2orf70 | 1.604744824 | 6.35E-20 | Glioma |
| 55062 | WIPI1 | 1.605103312 | 1.96E-68 | Glioma |
| 83593 | RASSF5 | 1.605399138 | 2.52E-44 | Glioma |
| 10865 | ARID5A | 1.6069666 | 1.69E-60 | Glioma |
| 2275 | FHL3 | 1.607701088 | 1.11E-64 | Glioma |
| 79746 | ECHDC3 | 1.608171387 | 4.70E-23 | Glioma |
| 23237 | ARC | 1.608262984 | 4.38E-19 | Glioma |
| 4257 | MGST1 | 1.608933091 | 4.45E-24 | Glioma |
| 205 | AK4 | 1.609116425 | 7.03E-36 | Glioma |
| 1902 | LPAR1 | 1.609119195 | 3.14E-21 | Glioma |
| 23550 | PSD4 | 1.60934918 | 1.07E-42 | Glioma |
| 917 | CD3G | 1.609371139 | 5.40E-23 | Glioma |
| 219833 | C11orf45 | 1.609387485 | 2.07E-41 | Glioma |
| 6335 | SCN9A | 1.609943889 | 1.98E-09 | Glioma |
| 81793 | TLR10 | 1.610872228 | 2.29E-26 | Glioma |
| 10052 | GJC1 | 1.611060131 | 5.67E-34 | Glioma |
| 55283 | MCOLN3 | 1.611186128 | 8.26E-16 | Glioma |
| 4647 | MYO7A | 1.6161552 | 5.38E-44 | Glioma |
| 84958 | SYTL1 | 1.616298939 | 1.31E-21 | Glioma |
| 84793 | None | 1.616544136 | 8.35E-22 | Glioma |
| 10964 | IFI44L | 1.617272218 | 4.25E-19 | Glioma |
| 967 | CD63 | 1.617545692 | 9.11E-68 | Glioma |
| 51473 | DCDC2 | 1.617975261 | 1.08E-19 | Glioma |
| 5376 | PMP22 | 1.619029954 | 9.98E-55 | Glioma |
| 3105 | HLA-A | 1.61905283 | 4.41E-53 | Glioma |
| 221188 | ADGRG5 | 1.621533151 | 5.24E-21 | Glioma |
| 5321 | PLA2G4A | 1.621824825 | 2.97E-36 | Glioma |
| 400710 | None | 1.622660786 | 7.92E-25 | Glioma |
| 59342 | SCPEP1 | 1.622824331 | 7.31E-85 | Glioma |
| 3218 | HOXB8 | 1.623317289 | 1.09E-10 | Glioma |
| 7100 | TLR5 | 1.624204257 | 1.93E-35 | Glioma |
| 29992 | PILRA | 1.624640555 | 1.33E-59 | Glioma |
| 54996 | Mar-02 | 1.627287511 | 3.98E-24 | Glioma |
| 259266 | ASPM | 1.628348761 | 4.27E-12 | Glioma |
| 3487 | IGFBP4 | 1.62863739 | 1.41E-31 | Glioma |
| 7764 | ZNF217 | 1.629251364 | 2.16E-57 | Glioma |
| 9022 | CLIC3 | 1.629936511 | 4.93E-24 | Glioma |
| 169611 | OLFML2A | 1.630170469 | 1.69E-37 | Glioma |
| 10184 | LHFPL2 | 1.630790979 | 5.23E-66 | Glioma |
| 50852 | TRAT1 | 1.630866686 | 4.46E-27 | Glioma |
| 139716 | GAB3 | 1.63113807 | 1.28E-65 | Glioma |
| 132430 | PABPC4L | 1.632373484 | 1.86E-31 | Glioma |
| 10544 | PROCR | 1.6329276 | 7.51E-56 | Glioma |
| 352961 | None | 1.633119207 | 4.11E-26 | Glioma |
| 5345 | SERPINF2 | 1.633276779 | 5.16E-50 | Glioma |
| 100271831 | None | 1.633404231 | 9.35E-13 | Glioma |
| 203328 | SUSD3 | 1.633678164 | 6.83E-28 | Glioma |
| 80328 | ULBP2 | 1.635130711 | 4.14E-24 | Glioma |
| 340152 | ZC3H12D | 1.636187767 | 7.15E-43 | Glioma |
| 65987 | KCTD14 | 1.637439215 | 1.51E-31 | Glioma |
| 8862 | APLN | 1.637994971 | 6.02E-35 | Glioma |
| 2882 | GPX7 | 1.638344718 | 8.43E-43 | Glioma |
| 4751 | NEK2 | 1.638625684 | 1.41E-13 | Glioma |
| 116071 | BATF2 | 1.638793345 | 9.42E-40 | Glioma |
| 10903 | MTMR11 | 1.638835786 | 4.80E-46 | Glioma |
| 5553 | PRG2 | 1.639017141 | 7.26E-25 | Glioma |
| 9516 | LITAF | 1.640955951 | 9.79E-45 | Glioma |
| 389289 | ANXA2R | 1.641233305 | 7.23E-38 | Glioma |
| 91351 | DDX60L | 1.641574227 | 5.61E-55 | Glioma |
| 147841 | SPC24 | 1.642460155 | 1.86E-12 | Glioma |
| 84182 | MINDY4 | 1.642488289 | 2.50E-33 | Glioma |
| 162515 | SLC16A11 | 1.642692944 | 8.12E-21 | Glioma |
| 4778 | NFE2 | 1.643612851 | 3.10E-30 | Glioma |
| 51393 | TRPV2 | 1.644002115 | 1.70E-55 | Glioma |
| 219293 | ATAD3C | 1.644089443 | 1.35E-23 | Glioma |
| 64220 | STRA6 | 1.64466201 | 8.00E-18 | Glioma |
| 26027 | ACOT11 | 1.644943135 | 5.57E-23 | Glioma |
| 3824 | KLRD1 | 1.645847893 | 7.84E-27 | Glioma |
| 10371 | SEMA3A | 1.646610079 | 5.97E-12 | Glioma |
| 389792 | IER5L | 1.647588722 | 2.56E-33 | Glioma |
| 7802 | DNALI1 | 1.648074023 | 1.11E-42 | Glioma |
| 3352 | HTR1D | 1.648207301 | 3.58E-16 | Glioma |
| 3566 | IL4R | 1.652299596 | 3.88E-66 | Glioma |
| 374393 | FAM111B | 1.652771868 | 9.15E-14 | Glioma |
| 10855 | HPSE | 1.65339186 | 7.32E-26 | Glioma |
| 6776 | STAT5A | 1.653872933 | 2.23E-54 | Glioma |
| 146556 | C16orf89 | 1.654214499 | 7.85E-21 | Glioma |
| 79695 | GALNT12 | 1.654264769 | 1.33E-41 | Glioma |
| 79690 | GAL3ST4 | 1.655122277 | 2.14E-65 | Glioma |
| 255783 | INAFM1 | 1.655528676 | 8.91E-48 | Glioma |
| 23498 | HAAO | 1.656377692 | 8.42E-42 | Glioma |
| 23351 | KHNYN | 1.65637811 | 4.62E-62 | Glioma |
| 84898 | PLXDC2 | 1.657495155 | 1.71E-38 | Glioma |
| 4616 | GADD45B | 1.6579406 | 3.51E-33 | Glioma |
| 84981 | None | 1.659315617 | 1.56E-50 | Glioma |
| 2001 | ELF5 | 1.661000242 | 7.33E-21 | Glioma |
| 136895 | C7orf31 | 1.662270383 | 2.83E-37 | Glioma |
| 84329 | HVCN1 | 1.662382126 | 1.84E-44 | Glioma |
| 5473 | PPBP | 1.663020041 | 6.45E-15 | Glioma |
| 400223 | None | 1.664020727 | 2.77E-33 | Glioma |
| 10216 | PRG4 | 1.66451777 | 7.82E-14 | Glioma |
| 27306 | HPGDS | 1.665112126 | 3.94E-29 | Glioma |
| 55872 | PBK | 1.669086459 | 3.05E-11 | Glioma |
| 55561 | CDC42BPG | 1.669169295 | 1.58E-22 | Glioma |
| 1031 | CDKN2C | 1.669591927 | 7.13E-24 | Glioma |
| 51050 | PI15 | 1.669846754 | 2.15E-18 | Glioma |
| 89876 | MAATS1 | 1.670732915 | 8.91E-22 | Glioma |
| 3908 | LAMA2 | 1.670751682 | 6.93E-34 | Glioma |
| 7272 | TTK | 1.673118398 | 2.96E-12 | Glioma |
| 4919 | ROR1 | 1.676420828 | 1.15E-25 | Glioma |
| 132160 | PPM1M | 1.676755392 | 1.13E-76 | Glioma |
| 387097 | None | 1.677680459 | 1.20E-24 | Glioma |
| 340277 | FAM221A | 1.678044178 | 3.05E-31 | Glioma |
| 50865 | HEBP1 | 1.679375955 | 5.28E-39 | Glioma |
| 1545 | CYP1B1 | 1.680936345 | 7.98E-18 | Glioma |
| 2857 | GPR34 | 1.681093241 | 8.19E-35 | Glioma |
| 3067 | HDC | 1.682520439 | 9.29E-15 | Glioma |
| 56938 | ARNTL2 | 1.684697246 | 1.00E-30 | Glioma |
| 84166 | NLRC5 | 1.684931237 | 3.35E-55 | Glioma |
| 30846 | EHD2 | 1.685666358 | 9.50E-50 | Glioma |
| 80723 | SLC35G2 | 1.685898615 | 2.58E-34 | Glioma |
| 54986 | ULK4 | 1.685921794 | 4.61E-50 | Glioma |
| 54869 | EPS8L1 | 1.685962977 | 3.84E-39 | Glioma |
| 58527 | ABRACL | 1.686158372 | 1.93E-52 | Glioma |
| 699 | BUB1 | 1.690147332 | 1.24E-15 | Glioma |
| 51129 | ANGPTL4 | 1.690571237 | 4.28E-21 | Glioma |
| 257106 | ARHGAP30 | 1.691662511 | 4.55E-58 | Glioma |
| 50846 | DHH | 1.692871751 | 2.50E-23 | Glioma |
| 55766 | H2AFJ | 1.6935746 | 9.07E-58 | Glioma |
| 121457 | IKBIP | 1.693579411 | 1.62E-55 | Glioma |
| 5493 | PPL | 1.693650339 | 3.49E-11 | Glioma |
| 7849 | PAX8 | 1.693960774 | 1.01E-14 | Glioma |
| 58484 | NLRC4 | 1.695590464 | 2.77E-53 | Glioma |
| 7298 | TYMS | 1.695784179 | 2.74E-23 | Glioma |
| 23308 | ICOSLG | 1.697290836 | 5.27E-43 | Glioma |
| 1158 | CKM | 1.697335635 | 5.68E-38 | Glioma |
| 25876 | SPEF1 | 1.697483756 | 2.82E-18 | Glioma |
| 79974 | CPED1 | 1.697985936 | 6.70E-31 | Glioma |
| 3663 | IRF5 | 1.699326274 | 1.15E-56 | Glioma |
| 9144 | SYNGR2 | 1.700074246 | 1.66E-61 | Glioma |
| 977 | CD151 | 1.700829304 | 7.20E-64 | Glioma |
| 64127 | NOD2 | 1.701006205 | 9.33E-34 | Glioma |
| 50856 | CLEC4A | 1.702331224 | 2.55E-57 | Glioma |
| 3037 | HAS2 | 1.703388821 | 1.95E-17 | Glioma |
| 2650 | GCNT1 | 1.705164702 | 1.26E-32 | Glioma |
| 7454 | WAS | 1.706365823 | 6.70E-62 | Glioma |
| 3203 | HOXA6 | 1.707701675 | 9.87E-22 | Glioma |
| 1368 | CPM | 1.707799277 | 3.91E-32 | Glioma |
| 84695 | LOXL3 | 1.708942696 | 9.45E-52 | Glioma |
| 4258 | MGST2 | 1.70916942 | 1.93E-89 | Glioma |
| 7040 | None | 1.710347426 | 5.34E-57 | Glioma |
| 10320 | IKZF1 | 1.712266401 | 4.75E-42 | Glioma |
| 117247 | SLC16A10 | 1.712709513 | 5.90E-18 | Glioma |
| 64699 | TMPRSS3 | 1.714323829 | 7.84E-13 | Glioma |
| 222171 | PRR15 | 1.715131962 | 6.77E-26 | Glioma |
| 10449 | ACAA2 | 1.715405185 | 2.90E-52 | Glioma |
| 29946 | SERTAD3 | 1.715955044 | 4.12E-58 | Glioma |
| 9154 | SLC28A1 | 1.716035217 | 7.02E-20 | Glioma |
| 4056 | LTC4S | 1.717144985 | 9.84E-26 | Glioma |
| 29800 | ZDHHC1 | 1.717561569 | 5.04E-37 | Glioma |
| 114041 | None | 1.717928694 | 7.07E-26 | Glioma |
| 2487 | FRZB | 1.718608683 | 5.86E-34 | Glioma |
| 79660 | PPP1R3B | 1.718663098 | 1.22E-45 | Glioma |
| 10123 | ARL4C | 1.72079504 | 9.04E-38 | Glioma |
| 64108 | RTP4 | 1.722531957 | 1.80E-49 | Glioma |
| 10863 | ADAM28 | 1.722727498 | 1.74E-37 | Glioma |
| 29760 | BLNK | 1.723298863 | 7.14E-28 | Glioma |
| 57121 | LPAR5 | 1.723967894 | 6.02E-36 | Glioma |
| 25884 | CHRDL2 | 1.724033082 | 1.52E-10 | Glioma |
| 113763 | ZBED6CL | 1.724210224 | 7.26E-24 | Glioma |
| 2184 | FAH | 1.724797004 | 6.14E-52 | Glioma |
| 389558 | FAM180A | 1.725146592 | 7.99E-13 | Glioma |
| 4065 | LY75 | 1.725442762 | 7.51E-41 | Glioma |
| 6283 | S100A12 | 1.725819853 | 1.24E-20 | Glioma |
| 146857 | SLFN13 | 1.726029086 | 6.31E-30 | Glioma |
| 1843 | DUSP1 | 1.726112142 | 3.45E-34 | Glioma |
| 5971 | RELB | 1.727118002 | 3.88E-63 | Glioma |
| 5624 | PROC | 1.727448926 | 1.30E-22 | Glioma |
| 5158 | PDE6B | 1.731885497 | 2.61E-31 | Glioma |
| 283298 | OLFML1 | 1.732269117 | 1.98E-31 | Glioma |
| 1033 | CDKN3 | 1.732532347 | 5.46E-26 | Glioma |
| 399888 | FAM180B | 1.732763469 | 9.30E-16 | Glioma |
| 4052 | LTBP1 | 1.733182699 | 4.12E-35 | Glioma |
| 149478 | BTBD19 | 1.733414302 | 1.00E-42 | Glioma |
| 3679 | ITGA7 | 1.733445386 | 2.51E-49 | Glioma |
| 2069 | EREG | 1.73530972 | 4.98E-15 | Glioma |
| 388389 | CCDC103 | 1.735386119 | 3.79E-43 | Glioma |
| 3134 | HLA-F | 1.735696682 | 3.70E-50 | Glioma |
| 5468 | PPARG | 1.73580631 | 1.46E-38 | Glioma |
| 57085 | AGTRAP | 1.736471056 | 7.98E-59 | Glioma |
| 3784 | KCNQ1 | 1.736765373 | 5.32E-49 | Glioma |
| 7133 | TNFRSF1B | 1.736772294 | 1.29E-57 | Glioma |
| 54478 | PIMREG | 1.737514058 | 7.53E-13 | Glioma |
| 3675 | ITGA3 | 1.738052458 | 1.99E-31 | Glioma |
| 163479 | FNDC7 | 1.738754712 | 1.72E-16 | Glioma |
| 5154 | PDGFA | 1.739666546 | 1.12E-40 | Glioma |
| 3116 | None | 1.739788107 | 2.60E-25 | Glioma |
| 83690 | CRISPLD1 | 1.741562246 | 2.25E-43 | Glioma |
| 3099 | HK2 | 1.741861806 | 4.05E-39 | Glioma |
| 246329 | STAC3 | 1.741990617 | 7.04E-61 | Glioma |
| 130340 | AP1S3 | 1.742423032 | 1.16E-48 | Glioma |
| 84525 | HOPX | 1.743073746 | 1.07E-20 | Glioma |
| 85016 | CFAP300 | 1.743153597 | 3.11E-16 | Glioma |
| 33 | ACADL | 1.743230235 | 1.87E-14 | Glioma |
| 9871 | SEC24D | 1.744481143 | 1.72E-64 | Glioma |
| 25907 | TMEM158 | 1.746262132 | 3.74E-29 | Glioma |
| 2615 | LRRC32 | 1.746286391 | 1.27E-36 | Glioma |
| 118788 | PIK3AP1 | 1.747566146 | 1.73E-53 | Glioma |
| 81607 | NECTIN4 | 1.747600175 | 8.99E-28 | Glioma |
| 24137 | KIF4A | 1.748031039 | 1.39E-19 | Glioma |
| 151648 | SGO1 | 1.748428807 | 3.34E-14 | Glioma |
| 542767 | PCOTH | 1.749014609 | 3.49E-37 | Glioma |
| 89857 | KLHL6 | 1.74919222 | 6.16E-40 | Glioma |
| 64856 | VWA1 | 1.749485687 | 4.31E-42 | Glioma |
| 151306 | GPBAR1 | 1.750027059 | 2.97E-46 | Glioma |
| 257358 | None | 1.750528685 | 2.10E-39 | Glioma |
| 4998 | ORC1 | 1.750927474 | 9.31E-20 | Glioma |
| 84815 | None | 1.751070424 | 6.36E-24 | Glioma |
| 7006 | TEC | 1.751167984 | 8.82E-41 | Glioma |
| 3613 | IMPA2 | 1.752352772 | 8.92E-54 | Glioma |
| 10261 | IGSF6 | 1.753567269 | 2.70E-41 | Glioma |
| 169792 | GLIS3 | 1.753782498 | 2.82E-30 | Glioma |
| 100130958 | SYCE1L | 1.754860398 | 1.56E-21 | Glioma |
| 8531 | YBX3 | 1.755075201 | 3.77E-53 | Glioma |
| 1288 | COL4A6 | 1.755258193 | 1.33E-15 | Glioma |
| 3673 | ITGA2 | 1.756705884 | 1.38E-31 | Glioma |
| 55647 | RAB20 | 1.756832641 | 4.91E-52 | Glioma |
| 860 | RUNX2 | 1.756927707 | 3.85E-48 | Glioma |
| 7490 | WT1 | 1.757696501 | 1.37E-14 | Glioma |
| 126969 | SLC44A3 | 1.758934954 | 9.41E-31 | Glioma |
| 6362 | CCL18 | 1.759037371 | 3.88E-09 | Glioma |
| 959 | CD40LG | 1.759362324 | 6.46E-30 | Glioma |
| 284415 | VSTM1 | 1.759916936 | 1.08E-19 | Glioma |
| 27120 | DKKL1 | 1.760417989 | 2.00E-23 | Glioma |
| 54436 | SH3TC1 | 1.762169598 | 7.08E-49 | Glioma |
| 1513 | CTSK | 1.763716416 | 2.63E-27 | Glioma |
| 3990 | LIPC | 1.763955719 | 8.29E-23 | Glioma |
| 3656 | IRAK2 | 1.76534202 | 5.26E-43 | Glioma |
| 79778 | MICALL2 | 1.766856657 | 3.67E-55 | Glioma |
| 80008 | TMEM156 | 1.766890105 | 2.05E-31 | Glioma |
| 644538 | SMIM10 | 1.767242047 | 6.31E-47 | Glioma |
| 7380 | UPK3A | 1.76727162 | 9.33E-19 | Glioma |
| 55784 | MCTP2 | 1.769738199 | 1.09E-16 | Glioma |
| 5352 | PLOD2 | 1.77112998 | 2.23E-43 | Glioma |
| 57333 | RCN3 | 1.771273339 | 7.17E-44 | Glioma |
| 2920 | CXCL2 | 1.771481961 | 1.73E-18 | Glioma |
| 79682 | CENPU | 1.773070183 | 2.27E-22 | Glioma |
| 23075 | SWAP70 | 1.773585184 | 9.11E-75 | Glioma |
| 4940 | OAS3 | 1.773784401 | 1.32E-39 | Glioma |
| 10316 | NMUR1 | 1.774626567 | 9.35E-36 | Glioma |
| 64926 | RASAL3 | 1.775158516 | 8.15E-44 | Glioma |
| 128239 | IQGAP3 | 1.776770508 | 2.12E-17 | Glioma |
| 890 | CCNA2 | 1.777035653 | 4.75E-23 | Glioma |
| 11024 | LILRA1 | 1.777050137 | 1.89E-29 | Glioma |
| 79713 | IGFLR1 | 1.777843307 | 1.29E-69 | Glioma |
| 91662 | NLRP12 | 1.778336904 | 1.51E-31 | Glioma |
| 51411 | BIN2 | 1.779356275 | 2.34E-48 | Glioma |
| 3232 | HOXD3 | 1.780531646 | 1.28E-09 | Glioma |
| 401207 | C5orf63 | 1.78089866 | 5.99E-24 | Glioma |
| 4053 | LTBP2 | 1.783194826 | 1.30E-42 | Glioma |
| 8793 | TNFRSF10D | 1.784872023 | 8.30E-48 | Glioma |
| 912 | CD1D | 1.784995012 | 1.43E-33 | Glioma |
| 8877 | None | 1.785047185 | 1.93E-31 | Glioma |
| 9214 | FCMR | 1.785419454 | 1.51E-35 | Glioma |
| 6591 | SNAI2 | 1.786105152 | 1.56E-24 | Glioma |
| 2535 | FZD2 | 1.786677266 | 2.06E-45 | Glioma |
| 1794 | DOCK2 | 1.786688033 | 4.89E-43 | Glioma |
| 54947 | LPCAT2 | 1.786762588 | 1.28E-38 | Glioma |
| 8635 | RNASET2 | 1.787672065 | 7.04E-68 | Glioma |
| 5023 | P2RX1 | 1.78775563 | 3.80E-23 | Glioma |
| 3915 | LAMC1 | 1.788050535 | 2.39E-48 | Glioma |
| 706 | TSPO | 1.788442587 | 7.54E-39 | Glioma |
| 2150 | F2RL1 | 1.788774835 | 4.07E-22 | Glioma |
| 1264 | CNN1 | 1.789279367 | 1.48E-16 | Glioma |
| 60675 | PROK2 | 1.789871908 | 3.45E-22 | Glioma |
| 54796 | BNC2 | 1.790249493 | 1.51E-33 | Glioma |
| 79989 | TTC26 | 1.791704062 | 3.34E-57 | Glioma |
| 257144 | GCSAM | 1.791882645 | 6.14E-34 | Glioma |
| 84057 | MND1 | 1.792266395 | 4.39E-22 | Glioma |
| 29969 | MDFIC | 1.792853674 | 6.90E-39 | Glioma |
| 153768 | PRELID2 | 1.793044042 | 2.46E-32 | Glioma |
| 64151 | NCAPG | 1.793578979 | 3.13E-14 | Glioma |
| 22798 | LAMB4 | 1.794318581 | 7.98E-30 | Glioma |
| 5696 | PSMB8 | 1.795578569 | 9.01E-63 | Glioma |
| 8323 | FZD6 | 1.795625118 | 3.57E-41 | Glioma |
| 440359 | None | 1.797705072 | 1.51E-39 | Glioma |
| 64900 | LPIN3 | 1.798538828 | 2.30E-29 | Glioma |
| 11030 | RBPMS | 1.798873041 | 7.51E-46 | Glioma |
| 112770 | GLMP | 1.79922197 | 9.12E-66 | Glioma |
| 133584 | EGFLAM | 1.799743793 | 6.92E-29 | Glioma |
| 6737 | TRIM21 | 1.800753521 | 1.78E-73 | Glioma |
| 606724 | None | 1.800921804 | 5.91E-54 | Glioma |
| 55803 | ADAP2 | 1.801654655 | 1.81E-70 | Glioma |
| 91937 | TIMD4 | 1.801782502 | 7.82E-22 | Glioma |
| 3601 | IL15RA | 1.802055172 | 4.34E-63 | Glioma |
| 286223 | S1PR3 | 1.80292509 | 1.10E-30 | Glioma |
| 54857 | GDPD2 | 1.804404095 | 6.89E-22 | Glioma |
| 282969 | FUOM | 1.805090474 | 1.50E-41 | Glioma |
| 51237 | MZB1 | 1.805095257 | 3.87E-20 | Glioma |
| 1969 | EPHA2 | 1.807321198 | 7.06E-40 | Glioma |
| 27202 | C5AR2 | 1.808696312 | 2.35E-35 | Glioma |
| 130814 | PQLC3 | 1.808928365 | 9.76E-79 | Glioma |
| 9610 | RIN1 | 1.810258656 | 1.28E-28 | Glioma |
| 6263 | RYR3 | 1.810750942 | 9.31E-17 | Glioma |
| 9121 | SLC16A5 | 1.811165017 | 6.65E-55 | Glioma |
| 122622 | ADSSL1 | 1.811843602 | 2.48E-38 | Glioma |
| 6850 | SYK | 1.8121703 | 2.99E-48 | Glioma |
| 78989 | COLEC11 | 1.812465805 | 3.31E-19 | Glioma |
| 1594 | CYP27B1 | 1.813894392 | 8.55E-18 | Glioma |
| 1945 | EFNA4 | 1.814027288 | 4.28E-42 | Glioma |
| 7098 | TLR3 | 1.814076679 | 6.43E-51 | Glioma |
| 162966 | ZNF600 | 1.814524347 | 2.71E-70 | Glioma |
| 333926 | PPM1J | 1.814948594 | 1.00E-26 | Glioma |
| 8870 | IER3 | 1.815061532 | 7.23E-40 | Glioma |
| 10398 | MYL9 | 1.815279293 | 1.60E-40 | Glioma |
| 130589 | GALM | 1.816170085 | 2.65E-53 | Glioma |
| 10553 | HTATIP2 | 1.816255186 | 5.66E-44 | Glioma |
| 1486 | CTBS | 1.816286575 | 4.60E-65 | Glioma |
| 347454 | SOWAHD | 1.816936147 | 1.65E-43 | Glioma |
| 5724 | PTAFR | 1.817348088 | 9.72E-30 | Glioma |
| 100132707 | None | 1.817444916 | 7.39E-64 | Glioma |
| 79083 | MLPH | 1.818040724 | 5.11E-17 | Glioma |
| 10344 | CCL26 | 1.821688516 | 1.48E-26 | Glioma |
| 653390 | None | 1.822390726 | 2.89E-34 | Glioma |
| 2533 | FYB1 | 1.822545574 | 1.81E-43 | Glioma |
| 145258 | GSC | 1.82257285 | 5.23E-19 | Glioma |
| 6990 | DYNLT3 | 1.823011488 | 3.59E-35 | Glioma |
| 90273 | CEACAM21 | 1.823160599 | 8.30E-39 | Glioma |
| 56659 | KCNK13 | 1.823765836 | 3.20E-42 | Glioma |
| 168090 | C6orf118 | 1.825139209 | 4.47E-20 | Glioma |
| 5271 | SERPINB8 | 1.825753245 | 1.41E-67 | Glioma |
| 2192 | FBLN1 | 1.825788529 | 3.01E-29 | Glioma |
| 995 | CDC25C | 1.827265394 | 4.09E-16 | Glioma |
| 8974 | P4HA2 | 1.82792179 | 1.81E-48 | Glioma |
| 6303 | SAT1 | 1.8303128 | 6.19E-69 | Glioma |
| 9235 | IL32 | 1.830862382 | 2.26E-36 | Glioma |
| 197259 | MLKL | 1.830934684 | 5.52E-76 | Glioma |
| 3687 | ITGAX | 1.831867519 | 6.99E-40 | Glioma |
| 3207 | HOXA11 | 1.832236958 | 7.72E-12 | Glioma |
| 5427 | POLE2 | 1.832815272 | 4.73E-22 | Glioma |
| 26996 | GPR160 | 1.833554131 | 9.89E-54 | Glioma |
| 338773 | TMEM119 | 1.833636337 | 8.95E-27 | Glioma |
| 8784 | TNFRSF18 | 1.835824741 | 2.56E-23 | Glioma |
| 245806 | VGLL2 | 1.835896987 | 2.33E-17 | Glioma |
| 858 | CAV2 | 1.83638581 | 3.06E-38 | Glioma |
| 55911 | APOBR | 1.838623604 | 1.19E-57 | Glioma |
| 8320 | EOMES | 1.839159578 | 7.31E-29 | Glioma |
| 8200 | GDF5 | 1.839196366 | 6.88E-26 | Glioma |
| 8792 | TNFRSF11A | 1.839640357 | 2.18E-29 | Glioma |
| 9168 | TMSB10 | 1.842230833 | 4.04E-56 | Glioma |
| 84179 | SLC49A3 | 1.844296846 | 1.72E-52 | Glioma |
| 9936 | CD302 | 1.844755994 | 1.29E-68 | Glioma |
| 80270 | HSD3B7 | 1.845020896 | 3.28E-72 | Glioma |
| 644943 | RASSF10 | 1.845421431 | 1.69E-16 | Glioma |
| 2735 | GLI1 | 1.846483192 | 3.19E-18 | Glioma |
| 2210 | FCGR1B | 1.846684574 | 6.74E-38 | Glioma |
| 51744 | CD244 | 1.846932064 | 6.70E-27 | Glioma |
| 4887 | NPY2R | 1.847245798 | 1.27E-11 | Glioma |
| 133418 | EMB | 1.848652026 | 3.68E-44 | Glioma |
| 9402 | GRAP2 | 1.84964879 | 1.16E-30 | Glioma |
| 221476 | PI16 | 1.850936751 | 2.29E-12 | Glioma |
| 79958 | DENND1C | 1.850938988 | 8.86E-51 | Glioma |
| 29887 | SNX10 | 1.851137574 | 4.14E-24 | Glioma |
| 79801 | SHCBP1 | 1.851173917 | 3.42E-31 | Glioma |
| 9002 | F2RL3 | 1.852074457 | 3.74E-20 | Glioma |
| 645367 | None | 1.852464207 | 9.93E-22 | Glioma |
| 920 | CD4 | 1.852640664 | 9.28E-66 | Glioma |
| 94059 | LENG9 | 1.85331359 | 3.68E-56 | Glioma |
| 7035 | TFPI | 1.854609523 | 6.45E-20 | Glioma |
| 29950 | SERTAD1 | 1.854754569 | 6.08E-60 | Glioma |
| 3753 | KCNE1 | 1.855110975 | 5.35E-25 | Glioma |
| 6932 | TCF7 | 1.856233 | 1.72E-56 | Glioma |
| 8826 | IQGAP1 | 1.856856037 | 3.44E-63 | Glioma |
| 1593 | CYP27A1 | 1.8571425 | 1.06E-42 | Glioma |
| 308 | ANXA5 | 1.858016095 | 2.16E-81 | Glioma |
| 84689 | None | 1.858806987 | 1.78E-38 | Glioma |
| 8626 | TP63 | 1.859552181 | 3.74E-21 | Glioma |
| 79651 | RHBDF2 | 1.862252484 | 5.06E-51 | Glioma |
| 642273 | FAM110C | 1.862793836 | 7.42E-21 | Glioma |
| 3386 | ICAM4 | 1.863048205 | 4.60E-36 | Glioma |
| 22925 | PLA2R1 | 1.863205174 | 4.79E-22 | Glioma |
| 1635 | DCTD | 1.864954998 | 6.79E-51 | Glioma |
| 6813 | STXBP2 | 1.865163774 | 1.01E-69 | Glioma |
| 338442 | HCAR2 | 1.865767882 | 2.60E-25 | Glioma |
| 4276 | None | 1.865795944 | 2.63E-54 | Glioma |
| 7128 | TNFAIP3 | 1.866124136 | 5.90E-60 | Glioma |
| 4332 | MNDA | 1.866221421 | 3.39E-47 | Glioma |
| 3597 | IL13RA1 | 1.866269128 | 8.38E-65 | Glioma |
| 9464 | HAND2 | 1.86690416 | 1.24E-10 | Glioma |
| 3577 | CXCR1 | 1.866914065 | 1.52E-19 | Glioma |
| 53832 | IL20RA | 1.867873619 | 3.77E-14 | Glioma |
| 54809 | SAMD9 | 1.867891104 | 4.79E-49 | Glioma |
| 2041 | EPHA1 | 1.868481768 | 2.83E-37 | Glioma |
| 2494 | NR5A2 | 1.869658527 | 8.76E-20 | Glioma |
| 567 | B2M | 1.870080502 | 8.67E-77 | Glioma |
| 85363 | TRIM5 | 1.870106442 | 6.90E-60 | Glioma |
| 1050 | CEBPA | 1.872649826 | 1.78E-49 | Glioma |
| 23231 | SEL1L3 | 1.873261989 | 9.23E-24 | Glioma |
| 140576 | S100A16 | 1.875050507 | 1.54E-54 | Glioma |
| 3856 | KRT8 | 1.877982593 | 5.96E-19 | Glioma |
| 3107 | HLA-C | 1.878138107 | 4.39E-67 | Glioma |
| 220134 | SKA1 | 1.878799406 | 1.97E-20 | Glioma |
| 79717 | PPCS | 1.879211409 | 1.33E-56 | Glioma |
| 84795 | PYROXD2 | 1.879368109 | 5.39E-26 | Glioma |
| 29851 | ICOS | 1.880389273 | 1.50E-29 | Glioma |
| 50619 | DEF6 | 1.880640836 | 2.58E-54 | Glioma |
| 7499 | XG | 1.880930798 | 4.42E-24 | Glioma |
| 84634 | KISS1R | 1.881947912 | 2.23E-17 | Glioma |
| 9510 | ADAMTS1 | 1.882586879 | 2.66E-40 | Glioma |
| 1026 | CDKN1A | 1.88263464 | 7.71E-34 | Glioma |
| 64518 | TEKT3 | 1.882858453 | 1.96E-33 | Glioma |
| 3269 | HRH1 | 1.883462316 | 4.84E-44 | Glioma |
| 11184 | MAP4K1 | 1.883554648 | 5.92E-52 | Glioma |
| 374467 | None | 1.884226238 | 1.04E-17 | Glioma |
| 51284 | TLR7 | 1.884488035 | 2.02E-39 | Glioma |
| 2353 | FOS | 1.884697656 | 6.95E-28 | Glioma |
| 7083 | TK1 | 1.885618306 | 3.83E-27 | Glioma |
| 8076 | MFAP5 | 1.886379033 | 3.38E-14 | Glioma |
| 5634 | PRPS2 | 1.88757778 | 1.08E-33 | Glioma |
| 1950 | EGF | 1.887964715 | 1.00E-29 | Glioma |
| 148170 | CDC42EP5 | 1.888689457 | 4.70E-33 | Glioma |
| 643866 | CBLN3 | 1.888698924 | 5.97E-32 | Glioma |
| 6382 | SDC1 | 1.888717747 | 1.04E-27 | Glioma |
| 94120 | SYTL3 | 1.888869422 | 3.24E-64 | Glioma |
| 2995 | GYPC | 1.889007705 | 3.13E-46 | Glioma |
| 201134 | CEP112 | 1.889160552 | 4.94E-59 | Glioma |
| 7465 | WEE1 | 1.889793766 | 1.33E-39 | Glioma |
| 4050 | LTB | 1.891711232 | 8.89E-39 | Glioma |
| 51700 | CYB5R2 | 1.892829479 | 4.05E-18 | Glioma |
| 140462 | ASB9 | 1.892935135 | 1.52E-41 | Glioma |
| 2787 | GNG5 | 1.89325881 | 1.63E-57 | Glioma |
| 11259 | FILIP1L | 1.893531251 | 2.08E-47 | Glioma |
| 2841 | GPR18 | 1.89398031 | 6.13E-35 | Glioma |
| 391712 | TRIM61 | 1.895422074 | 1.03E-25 | Glioma |
| 59 | ACTA2 | 1.897578671 | 1.92E-37 | Glioma |
| 3684 | ITGAM | 1.897986391 | 3.43E-44 | Glioma |
| 400696 | None | 1.897987512 | 4.11E-24 | Glioma |
| 2769 | GNA15 | 1.900055218 | 5.89E-49 | Glioma |
| 83666 | PARP9 | 1.900248554 | 2.11E-82 | Glioma |
| 100216001 | None | 1.902619888 | 3.48E-27 | Glioma |
| 140738 | TMEM37 | 1.902992178 | 2.23E-51 | Glioma |
| 9603 | NFE2L3 | 1.903821018 | 3.97E-51 | Glioma |
| 5167 | ENPP1 | 1.90404628 | 7.21E-41 | Glioma |
| 7903 | ST8SIA4 | 1.904244751 | 1.91E-60 | Glioma |
| 84803 | GPAT3 | 1.905825047 | 9.31E-26 | Glioma |
| 8318 | CDC45 | 1.906285069 | 4.13E-14 | Glioma |
| 90288 | EFCAB12 | 1.90772325 | 3.57E-24 | Glioma |
| 84417 | C2orf40 | 1.908334235 | 1.34E-20 | Glioma |
| 55355 | HJURP | 1.9086543 | 1.35E-16 | Glioma |
| 284756 | C20orf197 | 1.909363829 | 6.24E-37 | Glioma |
| 130813 | C2orf50 | 1.910408022 | 2.06E-18 | Glioma |
| 4804 | NGFR | 1.910672226 | 4.77E-24 | Glioma |
| 6405 | SEMA3F | 1.911199963 | 9.33E-34 | Glioma |
| 3556 | IL1RAP | 1.912900481 | 2.51E-35 | Glioma |
| 54518 | APBB1IP | 1.912948165 | 3.06E-43 | Glioma |
| 56975 | FAM20C | 1.913467735 | 7.61E-60 | Glioma |
| 919 | CD247 | 1.913726536 | 5.25E-42 | Glioma |
| 29923 | HILPDA | 1.915821976 | 1.74E-38 | Glioma |
| 6023 | None | 1.916436741 | 5.96E-16 | Glioma |
| 2672 | GFI1 | 1.917182411 | 2.18E-45 | Glioma |
| 127343 | DMBX1 | 1.917359438 | 3.89E-21 | Glioma |
| 85329 | LGALS12 | 1.918051182 | 1.15E-32 | Glioma |
| 5029 | P2RY2 | 1.918355157 | 1.09E-26 | Glioma |
| 81578 | COL21A1 | 1.920848397 | 8.47E-15 | Glioma |
| 56062 | KLHL4 | 1.92135384 | 6.42E-26 | Glioma |
| 80231 | CXorf21 | 1.92313549 | 6.53E-43 | Glioma |
| 11262 | SP140 | 1.923721197 | 2.03E-61 | Glioma |
| 828 | CAPS | 1.924473514 | 2.76E-31 | Glioma |
| 2242 | FES | 1.925219707 | 2.01E-81 | Glioma |
| 51131 | PHF11 | 1.925413366 | 1.11E-56 | Glioma |
| 79626 | TNFAIP8L2 | 1.925510201 | 3.51E-56 | Glioma |
| 55504 | TNFRSF19 | 1.925903562 | 9.79E-43 | Glioma |
| 64170 | CARD9 | 1.926642236 | 1.86E-54 | Glioma |
| 677779 | None | 1.926705363 | 1.63E-20 | Glioma |
| 80045 | GPR157 | 1.928652579 | 6.02E-39 | Glioma |
| 64407 | RGS18 | 1.929432095 | 4.99E-45 | Glioma |
| 1326 | MAP3K8 | 1.929874183 | 6.53E-59 | Glioma |
| 93589 | CACNA2D4 | 1.930952182 | 1.12E-43 | Glioma |
| 290 | ANPEP | 1.93365644 | 5.51E-32 | Glioma |
| 115727 | RASGRP4 | 1.933661012 | 8.16E-50 | Glioma |
| 59341 | TRPV4 | 1.934152657 | 1.32E-37 | Glioma |
| 100128927 | None | 1.934396892 | 6.42E-47 | Glioma |
| 283487 | None | 1.935137257 | 4.84E-43 | Glioma |
| 128178 | EDARADD | 1.935792131 | 4.83E-33 | Glioma |
| 10677 | AVIL | 1.936897355 | 3.80E-33 | Glioma |
| 80149 | ZC3H12A | 1.937658281 | 1.18E-63 | Glioma |
| 2121 | EVC | 1.938119459 | 2.87E-34 | Glioma |
| 2116 | ETV2 | 1.938534623 | 2.25E-29 | Glioma |
| 400804 | None | 1.939086902 | 4.10E-28 | Glioma |
| 199675 | MCEMP1 | 1.939432394 | 1.05E-20 | Glioma |
| 54558 | SPATA6 | 1.939918855 | 4.21E-25 | Glioma |
| 4582 | MUC1 | 1.940138126 | 1.01E-52 | Glioma |
| 7351 | UCP2 | 1.940295038 | 1.54E-52 | Glioma |
| 63940 | GPSM3 | 1.940720626 | 8.13E-76 | Glioma |
| 90226 | UCN2 | 1.941206867 | 1.08E-24 | Glioma |
| 54830 | NUP62CL | 1.944135808 | 4.12E-22 | Glioma |
| 6916 | TBXAS1 | 1.945017972 | 5.61E-57 | Glioma |
| 5873 | RAB27A | 1.945597651 | 1.03E-68 | Glioma |
| 127733 | UBXN10 | 1.946108468 | 4.60E-12 | Glioma |
| 8970 | HIST1H2BJ | 1.947158342 | 6.16E-32 | Glioma |
| 120939 | TMEM52B | 1.94743176 | 2.75E-36 | Glioma |
| 196 | AHR | 1.94871525 | 3.00E-36 | Glioma |
| 150372 | NFAM1 | 1.949735941 | 4.27E-61 | Glioma |
| 283130 | SLC25A45 | 1.95140057 | 5.32E-49 | Glioma |
| 54961 | SSH3 | 1.951567208 | 1.40E-51 | Glioma |
| 8778 | SIGLEC5 | 1.951928108 | 3.39E-46 | Glioma |
| 4081 | MAB21L1 | 1.952293772 | 1.76E-20 | Glioma |
| 8832 | CD84 | 1.953285772 | 2.34E-34 | Glioma |
| 81848 | SPRY4 | 1.954753016 | 1.58E-24 | Glioma |
| 26253 | CLEC4E | 1.954846467 | 1.08E-17 | Glioma |
| 7940 | LST1 | 1.955079708 | 1.22E-57 | Glioma |
| 91543 | RSAD2 | 1.955539504 | 1.05E-31 | Glioma |
| 286042 | None | 1.955864565 | 1.64E-30 | Glioma |
| 203427 | SLC25A43 | 1.956062463 | 6.16E-34 | Glioma |
| 5176 | SERPINF1 | 1.956147131 | 4.84E-30 | Glioma |
| 55220 | KLHDC8A | 1.956594903 | 2.26E-29 | Glioma |
| 760 | CA2 | 1.957284736 | 5.54E-45 | Glioma |
| 6693 | SPN | 1.958069324 | 3.06E-53 | Glioma |
| 53834 | FGFRL1 | 1.958396804 | 5.20E-48 | Glioma |
| 160857 | CCDC122 | 1.958911688 | 3.20E-33 | Glioma |
| 1263 | PLK3 | 1.95958321 | 1.16E-98 | Glioma |
| 23166 | STAB1 | 1.960062864 | 1.39E-48 | Glioma |
| 9290 | GPR55 | 1.960087538 | 5.33E-32 | Glioma |
| 25891 | PAMR1 | 1.960550337 | 1.42E-35 | Glioma |
| 83449 | PMFBP1 | 1.960698727 | 2.97E-43 | Glioma |
| 343521 | TCTEX1D4 | 1.9609755 | 5.62E-35 | Glioma |
| 26136 | TES | 1.961710695 | 1.70E-60 | Glioma |
| 79442 | LRRC2 | 1.961808653 | 1.06E-16 | Glioma |
| 8228 | PNPLA4 | 1.962229311 | 1.46E-28 | Glioma |
| 4067 | LYN | 1.962701784 | 8.62E-85 | Glioma |
| 54751 | FBLIM1 | 1.963053634 | 8.33E-30 | Glioma |
| 839 | CASP6 | 1.964194227 | 1.64E-79 | Glioma |
| 644150 | WIPF3 | 1.965126793 | 6.12E-20 | Glioma |
| 948 | CD36 | 1.965871185 | 4.16E-21 | Glioma |
| 55603 | TENT5A | 1.966346546 | 5.75E-55 | Glioma |
| 83483 | PLVAP | 1.966536253 | 3.87E-39 | Glioma |
| 7444 | VRK2 | 1.967672302 | 8.37E-57 | Glioma |
| 1958 | EGR1 | 1.967832851 | 9.72E-28 | Glioma |
| 338557 | FFAR4 | 1.968401497 | 5.16E-33 | Glioma |
| 50848 | F11R | 1.96882482 | 8.27E-80 | Glioma |
| 54935 | DUSP23 | 1.969270758 | 2.23E-60 | Glioma |
| 5013 | OTX1 | 1.970037068 | 3.93E-19 | Glioma |
| 11000 | SLC27A3 | 1.970669663 | 4.11E-58 | Glioma |
| 2512 | FTL | 1.971580273 | 1.42E-74 | Glioma |
| 11098 | PRSS23 | 1.972863148 | 2.07E-45 | Glioma |
| 26301 | GBGT1 | 1.973617999 | 9.76E-71 | Glioma |
| 434 | ASIP | 1.973952763 | 3.89E-29 | Glioma |
| 53840 | TRIM34 | 1.974573951 | 1.31E-72 | Glioma |
| 8353 | HIST1H3E | 1.975696922 | 8.72E-30 | Glioma |
| 340075 | ARSI | 1.976418133 | 7.46E-24 | Glioma |
| 114827 | FHAD1 | 1.976475281 | 5.15E-26 | Glioma |
| 2745 | GLRX | 1.976627015 | 6.18E-72 | Glioma |
| 3665 | IRF7 | 1.97712632 | 2.61E-65 | Glioma |
| 84174 | SLA2 | 1.977158692 | 5.24E-51 | Glioma |
| 5777 | PTPN6 | 1.977639135 | 1.12E-67 | Glioma |
| 3215 | HOXB5 | 1.97803086 | 3.05E-19 | Glioma |
| 23630 | KCNE5 | 1.97863238 | 3.40E-13 | Glioma |
| 137902 | PXDNL | 1.979973788 | 1.87E-21 | Glioma |
| 51384 | WNT16 | 1.980655564 | 7.42E-15 | Glioma |
| 2669 | GEM | 1.980692677 | 1.18E-34 | Glioma |
| 2643 | GCH1 | 1.980903904 | 7.02E-69 | Glioma |
| 11004 | KIF2C | 1.981228291 | 6.76E-22 | Glioma |
| 2897 | GRIK1 | 1.981392754 | 2.72E-17 | Glioma |
| 5603 | MAPK13 | 1.981753171 | 5.36E-32 | Glioma |
| 140766 | ADAMTS14 | 1.981872149 | 2.40E-23 | Glioma |
| 54587 | MXRA8 | 1.982499989 | 1.70E-56 | Glioma |
| 10954 | PDIA5 | 1.983549433 | 6.64E-48 | Glioma |
| 9104 | RGN | 1.983763775 | 1.91E-26 | Glioma |
| 4547 | MTTP | 1.984819755 | 3.34E-25 | Glioma |
| 389549 | FEZF1 | 1.985338835 | 2.89E-17 | Glioma |
| 4615 | MYD88 | 1.985974798 | 4.96E-63 | Glioma |
| 121355 | GTSF1 | 1.988311764 | 5.17E-29 | Glioma |
| 92340 | PRR29 | 1.988692487 | 1.74E-29 | Glioma |
| 11006 | LILRB4 | 1.989538731 | 2.20E-47 | Glioma |
| 10403 | NDC80 | 1.990182934 | 1.08E-16 | Glioma |
| 3726 | JUNB | 1.990532981 | 3.82E-45 | Glioma |
| 414152 | C10orf105 | 1.992320013 | 3.15E-17 | Glioma |
| 56925 | LXN | 1.994765625 | 1.13E-46 | Glioma |
| 255104 | TMCO4 | 1.99516582 | 6.70E-62 | Glioma |
| 3912 | LAMB1 | 1.995381661 | 1.47E-33 | Glioma |
| 143888 | KDELC2 | 1.995537001 | 7.61E-60 | Glioma |
| 51268 | PIPOX | 1.996522068 | 3.79E-41 | Glioma |
| 644815 | FAM83G | 1.998473867 | 1.63E-28 | Glioma |
| 6510 | SLC1A5 | 1.998622892 | 5.37E-46 | Glioma |
| 6192 | RPS4Y1 | 1.999249015 | 0.00048355 | Glioma |
| 7409 | VAV1 | 1.999992225 | 1.15E-53 | Glioma |
| 374872 | PEAK3 | 2.000938154 | 2.44E-55 | Glioma |
| 219623 | TMEM26 | 2.001374466 | 3.83E-28 | Glioma |
| 221421 | RSPH9 | 2.002367277 | 2.32E-16 | Glioma |
| 6195 | RPS6KA1 | 2.003321431 | 4.27E-67 | Glioma |
| 55244 | SLC47A1 | 2.003863143 | 9.06E-33 | Glioma |
| 3055 | HCK | 2.006244952 | 7.51E-63 | Glioma |
| 10610 | ST6GALNAC2 | 2.006313989 | 1.07E-27 | Glioma |
| 4055 | LTBR | 2.00654331 | 2.70E-82 | Glioma |
| 3554 | IL1R1 | 2.006593328 | 5.65E-33 | Glioma |
| 55143 | CDCA8 | 2.00982514 | 2.76E-25 | Glioma |
| 4542 | MYO1F | 2.009838944 | 6.81E-68 | Glioma |
| 84624 | FNDC1 | 2.010079357 | 2.09E-15 | Glioma |
| 9398 | CD101 | 2.0107051 | 1.74E-44 | Glioma |
| 8475 | None | 2.012822587 | 1.63E-15 | Glioma |
| 343413 | FCRL6 | 2.013054532 | 8.51E-45 | Glioma |
| 150771 | ITPRIPL1 | 2.01335484 | 1.57E-32 | Glioma |
| 128209 | KLF17 | 2.013812875 | 6.46E-28 | Glioma |
| 3339 | HSPG2 | 2.015829002 | 3.61E-36 | Glioma |
| 219855 | SLC37A2 | 2.017021128 | 1.14E-52 | Glioma |
| 10586 | MAB21L2 | 2.017195374 | 4.23E-17 | Glioma |
| 148741 | ANKRD35 | 2.017367632 | 8.11E-26 | Glioma |
| 115701 | ALPK2 | 2.01746204 | 3.57E-17 | Glioma |
| 9034 | CCRL2 | 2.018551814 | 3.61E-67 | Glioma |
| 3071 | NCKAP1L | 2.019954737 | 2.51E-56 | Glioma |
| 340206 | None | 2.020254402 | 9.26E-29 | Glioma |
| 143689 | PIWIL4 | 2.020956555 | 5.23E-30 | Glioma |
| 89790 | SIGLEC10 | 2.022457087 | 4.60E-51 | Glioma |
| 6284 | S100A13 | 2.023151551 | 3.29E-43 | Glioma |
| 10161 | LPAR6 | 2.023371996 | 3.70E-75 | Glioma |
| 3965 | LGALS9 | 2.024368744 | 1.98E-66 | Glioma |
| 51296 | SLC15A3 | 2.025351257 | 1.98E-74 | Glioma |
| 140458 | ASB5 | 2.025669518 | 3.32E-17 | Glioma |
| 4071 | TM4SF1 | 2.026107531 | 3.88E-40 | Glioma |
| 199 | AIF1 | 2.02619187 | 5.68E-55 | Glioma |
| 23650 | TRIM29 | 2.028251199 | 2.97E-16 | Glioma |
| 9940 | DLEC1 | 2.029039896 | 7.46E-24 | Glioma |
| 958 | CD40 | 2.030369352 | 1.09E-73 | Glioma |
| 257101 | ZNF683 | 2.033605165 | 5.38E-33 | Glioma |
| 9493 | KIF23 | 2.03433622 | 3.93E-22 | Glioma |
| 79865 | TREML2 | 2.034671392 | 9.63E-37 | Glioma |
| 116844 | LRG1 | 2.03529812 | 4.98E-41 | Glioma |
| 10406 | WFDC2 | 2.036423873 | 4.43E-24 | Glioma |
| 26524 | LATS2 | 2.036669358 | 4.01E-69 | Glioma |
| 2173 | FABP7 | 2.037377111 | 6.33E-26 | Glioma |
| 5002 | SLC22A18 | 2.037555753 | 5.94E-45 | Glioma |
| 8794 | TNFRSF10C | 2.038193086 | 8.98E-51 | Glioma |
| 85480 | TSLP | 2.038497322 | 3.96E-26 | Glioma |
| 127707 | KLHDC7A | 2.038663279 | 1.41E-21 | Glioma |
| 256329 | LMNTD2 | 2.038787118 | 1.41E-34 | Glioma |
| 2139 | EYA2 | 2.039465342 | 3.74E-30 | Glioma |
| 3936 | LCP1 | 2.041181323 | 1.95E-63 | Glioma |
| 10475 | TRIM38 | 2.04130907 | 9.26E-82 | Glioma |
| 8418 | None | 2.04206648 | 2.67E-58 | Glioma |
| 89848 | FCHSD1 | 2.04232278 | 2.37E-67 | Glioma |
| 6614 | SIGLEC1 | 2.042344407 | 1.67E-25 | Glioma |
| 374907 | B3GNT8 | 2.042848361 | 2.96E-47 | Glioma |
| 126432 | RINL | 2.043163542 | 1.74E-59 | Glioma |
| 9122 | SLC16A4 | 2.044065341 | 3.49E-56 | Glioma |
| 1305 | COL13A1 | 2.044134977 | 1.24E-17 | Glioma |
| 7373 | COL14A1 | 2.045159959 | 8.03E-22 | Glioma |
| 64411 | ARAP3 | 2.045331649 | 1.30E-56 | Glioma |
| 55106 | SLFN12 | 2.0460596 | 9.56E-61 | Glioma |
| 285830 | None | 2.046247611 | 1.97E-43 | Glioma |
| 2766 | GMPR | 2.04737097 | 1.70E-35 | Glioma |
| 976 | ADGRE5 | 2.049380357 | 1.36E-58 | Glioma |
| 2537 | IFI6 | 2.050270021 | 6.22E-41 | Glioma |
| 256380 | SCML4 | 2.051160658 | 4.15E-29 | Glioma |
| 11117 | EMILIN1 | 2.055891881 | 1.20E-56 | Glioma |
| 3939 | LDHA | 2.056162375 | 1.56E-55 | Glioma |
| 10581 | IFITM2 | 2.056204891 | 5.00E-66 | Glioma |
| 23533 | PIK3R5 | 2.05649203 | 9.72E-60 | Glioma |
| 100132417 | None | 2.056507319 | 1.83E-42 | Glioma |
| 2118 | ETV4 | 2.056695841 | 1.61E-13 | Glioma |
| 131450 | CD200R1 | 2.056706016 | 3.94E-37 | Glioma |
| 1051 | CEBPB | 2.057430668 | 1.10E-71 | Glioma |
| 6518 | SLC2A5 | 2.057880605 | 7.45E-39 | Glioma |
| 57228 | SMAGP | 2.060517454 | 2.95E-49 | Glioma |
| 117245 | HRASLS5 | 2.06053999 | 1.44E-20 | Glioma |
| 345895 | RSPH4A | 2.060712069 | 4.93E-28 | Glioma |
| 397 | ARHGDIB | 2.061770959 | 2.02E-85 | Glioma |
| 84270 | CARD19 | 2.062214871 | 8.82E-52 | Glioma |
| 2219 | FCN1 | 2.062923325 | 8.47E-21 | Glioma |
| 81930 | KIF18A | 2.063269136 | 1.35E-25 | Glioma |
| 432369 | None | 2.063379208 | 3.28E-44 | Glioma |
| 4499 | MT1M | 2.063531519 | 2.38E-33 | Glioma |
| 9450 | LY86 | 2.065359132 | 1.65E-54 | Glioma |
| 11010 | GLIPR1 | 2.066294056 | 7.94E-58 | Glioma |
| 2700 | GJA3 | 2.067268408 | 6.01E-17 | Glioma |
| 113277 | TMEM106A | 2.068850762 | 7.73E-58 | Glioma |
| 255239 | ANKK1 | 2.06905831 | 3.94E-32 | Glioma |
| 9232 | PTTG1 | 2.070347778 | 3.56E-32 | Glioma |
| 27295 | PDLIM3 | 2.070789005 | 3.14E-47 | Glioma |
| 3081 | HGD | 2.071497137 | 2.02E-21 | Glioma |
| 9 | NAT1 | 2.072634839 | 2.44E-63 | Glioma |
| 9212 | AURKB | 2.073864329 | 5.38E-16 | Glioma |
| 5224 | PGAM2 | 2.073924636 | 3.26E-32 | Glioma |
| 9582 | APOBEC3B | 2.074446934 | 2.37E-29 | Glioma |
| 4312 | MMP1 | 2.074509861 | 1.13E-17 | Glioma |
| 283131 | None | 2.074569324 | 3.08E-46 | Glioma |
| 185 | AGTR1 | 2.078588357 | 1.37E-26 | Glioma |
| 53827 | FXYD5 | 2.080398524 | 3.99E-72 | Glioma |
| 2827 | GPR3 | 2.080676076 | 1.76E-36 | Glioma |
| 26873 | OPLAH | 2.081200225 | 6.67E-39 | Glioma |
| 8532 | CPZ | 2.081327637 | 2.23E-18 | Glioma |
| 284110 | GSDMA | 2.081386874 | 6.70E-21 | Glioma |
| 3490 | IGFBP7 | 2.0852567 | 5.00E-60 | Glioma |
| 10577 | NPC2 | 2.087318348 | 4.20E-86 | Glioma |
| 10095 | ARPC1B | 2.088190607 | 1.67E-79 | Glioma |
| 157313 | CDCA2 | 2.089503557 | 7.21E-23 | Glioma |
| 695 | BTK | 2.090838537 | 8.86E-61 | Glioma |
| 7291 | TWIST1 | 2.092193793 | 2.03E-28 | Glioma |
| 64333 | ARHGAP9 | 2.092568847 | 5.19E-73 | Glioma |
| 51816 | ADA2 | 2.094393717 | 7.01E-66 | Glioma |
| 2124 | EVI2B | 2.095542691 | 1.61E-56 | Glioma |
| 79924 | ADM2 | 2.095570221 | 1.11E-26 | Glioma |
| 11123 | RCAN3 | 2.095720703 | 7.08E-45 | Glioma |
| 925 | CD8A | 2.095728557 | 1.07E-29 | Glioma |
| 2863 | GPR39 | 2.096975292 | 5.27E-44 | Glioma |
| 57134 | MAN1C1 | 2.097292647 | 1.80E-49 | Glioma |
| 117144 | CATSPER1 | 2.09813164 | 5.32E-34 | Glioma |
| 79630 | C1orf54 | 2.099055827 | 4.04E-70 | Glioma |
| 84885 | ZDHHC12 | 2.100419245 | 7.87E-70 | Glioma |
| 1440 | CSF3 | 2.100998433 | 2.83E-17 | Glioma |
| 10024 | TROAP | 2.104607338 | 7.96E-18 | Glioma |
| 7293 | TNFRSF4 | 2.105541907 | 9.94E-35 | Glioma |
| 55723 | ASF1B | 2.105824005 | 2.91E-26 | Glioma |
| 56253 | CRTAM | 2.106012264 | 2.61E-37 | Glioma |
| 6876 | TAGLN | 2.10607073 | 2.48E-40 | Glioma |
| 4496 | MT1H | 2.106748632 | 1.94E-21 | Glioma |
| 27128 | CYTH4 | 2.107164499 | 3.90E-64 | Glioma |
| 58475 | MS4A7 | 2.107448551 | 1.32E-53 | Glioma |
| 29957 | SLC25A24 | 2.10770086 | 1.36E-66 | Glioma |
| 5698 | PSMB9 | 2.109948485 | 3.09E-66 | Glioma |
| 9636 | ISG15 | 2.11008226 | 6.93E-37 | Glioma |
| 5294 | PIK3CG | 2.111864135 | 3.91E-42 | Glioma |
| 857 | CAV1 | 2.112049317 | 1.47E-34 | Glioma |
| 5920 | RARRES3 | 2.112075195 | 1.26E-50 | Glioma |
| 4063 | LY9 | 2.112762916 | 7.45E-40 | Glioma |
| 64761 | PARP12 | 2.11331218 | 2.71E-80 | Glioma |
| 1441 | CSF3R | 2.114052 | 3.37E-60 | Glioma |
| 401027 | C2orf66 | 2.114822243 | 1.75E-31 | Glioma |
| 345274 | SLC10A6 | 2.115232208 | 4.89E-29 | Glioma |
| 29125 | C11orf21 | 2.115299909 | 2.28E-43 | Glioma |
| 9535 | GMFG | 2.116965068 | 1.39E-77 | Glioma |
| 1647 | GADD45A | 2.117201755 | 9.51E-59 | Glioma |
| 29933 | GPR132 | 2.117405584 | 5.59E-59 | Glioma |
| 401494 | HACD4 | 2.117939558 | 6.62E-64 | Glioma |
| 10647 | SCGB1D2 | 2.119913053 | 5.38E-21 | Glioma |
| 285888 | CNPY1 | 2.12037753 | 1.08E-21 | Glioma |
| 187 | APLNR | 2.121626164 | 3.43E-26 | Glioma |
| 64105 | CENPK | 2.123931829 | 2.34E-24 | Glioma |
| 51450 | PRRX2 | 2.124137726 | 1.46E-22 | Glioma |
| 2519 | FUCA2 | 2.124153785 | 6.05E-62 | Glioma |
| 112616 | CMTM7 | 2.124339817 | 7.25E-62 | Glioma |
| 9050 | PSTPIP2 | 2.124887585 | 1.14E-60 | Glioma |
| 84106 | PRAM1 | 2.125425979 | 7.76E-51 | Glioma |
| 151056 | PLB1 | 2.125965309 | 1.05E-74 | Glioma |
| 151242 | PPP1R1C | 2.127007968 | 4.57E-30 | Glioma |
| 1643 | DDB2 | 2.127983912 | 2.61E-89 | Glioma |
| 8807 | IL18RAP | 2.12874001 | 3.15E-34 | Glioma |
| 140 | ADORA3 | 2.129699348 | 1.00E-51 | Glioma |
| 923 | CD6 | 2.129777147 | 1.87E-35 | Glioma |
| 80835 | TAS1R1 | 2.12997869 | 1.14E-31 | Glioma |
| 347735 | SERINC2 | 2.13017453 | 2.32E-30 | Glioma |
| 55247 | NEIL3 | 2.130874587 | 6.14E-20 | Glioma |
| 201299 | RDM1 | 2.132177551 | 4.63E-32 | Glioma |
| 151534 | None | 2.132517871 | 2.23E-65 | Glioma |
| 3594 | IL12RB1 | 2.135244604 | 1.76E-53 | Glioma |
| 7412 | VCAM1 | 2.135403438 | 8.45E-22 | Glioma |
| 719 | C3AR1 | 2.136717422 | 3.26E-52 | Glioma |
| 85478 | CCDC65 | 2.138396012 | 1.93E-31 | Glioma |
| 65999 | LRRC61 | 2.138401548 | 2.78E-23 | Glioma |
| 170591 | S100Z | 2.138825034 | 3.69E-48 | Glioma |
| 3587 | IL10RA | 2.140194122 | 6.62E-61 | Glioma |
| 10561 | IFI44 | 2.140461681 | 2.21E-68 | Glioma |
| 220388 | CCDC89 | 2.141027103 | 7.86E-42 | Glioma |
| 400043 | None | 2.141286954 | 3.04E-37 | Glioma |
| 1493 | CTLA4 | 2.142438304 | 1.94E-33 | Glioma |
| 143630 | UBQLNL | 2.143031426 | 6.75E-42 | Glioma |
| 1522 | CTSZ | 2.144174279 | 2.00E-78 | Glioma |
| 64092 | SAMSN1 | 2.145432853 | 1.36E-52 | Glioma |
| 91523 | PCED1B | 2.147610089 | 4.89E-69 | Glioma |
| 25903 | OLFML2B | 2.147883255 | 1.98E-37 | Glioma |
| 26157 | GIMAP2 | 2.148050066 | 2.13E-88 | Glioma |
| 3937 | LCP2 | 2.149700142 | 4.44E-81 | Glioma |
| 8676 | STX11 | 2.150694852 | 2.14E-63 | Glioma |
| 9768 | PCLAF | 2.151160281 | 4.53E-21 | Glioma |
| 10627 | MYL12A | 2.154504253 | 7.47E-100 | Glioma |
| 283431 | GAS2L3 | 2.15570607 | 8.33E-25 | Glioma |
| 135228 | CD109 | 2.156751464 | 2.93E-49 | Glioma |
| 163702 | IFNLR1 | 2.157422076 | 5.83E-39 | Glioma |
| 1536 | CYBB | 2.158173775 | 4.20E-49 | Glioma |
| 633 | BGN | 2.158923236 | 1.15E-49 | Glioma |
| 100129792 | CCDC152 | 2.159206528 | 1.82E-42 | Glioma |
| 7462 | LAT2 | 2.160366798 | 2.15E-64 | Glioma |
| 7117 | None | 2.161412705 | 2.16E-91 | Glioma |
| 2335 | FN1 | 2.162204181 | 3.24E-46 | Glioma |
| 3106 | HLA-B | 2.162265934 | 8.12E-74 | Glioma |
| 54102 | CLIC6 | 2.162643423 | 3.24E-21 | Glioma |
| 11027 | LILRA2 | 2.163367287 | 1.15E-50 | Glioma |
| 389161 | ANKUB1 | 2.163446505 | 7.12E-21 | Glioma |
| 6340 | SCNN1G | 2.163492155 | 6.07E-21 | Glioma |
| 146850 | PIK3R6 | 2.163585978 | 1.62E-61 | Glioma |
| 9133 | CCNB2 | 2.163765288 | 1.98E-17 | Glioma |
| 10100 | TSPAN2 | 2.163993763 | 7.05E-26 | Glioma |
| 10666 | CD226 | 2.164074996 | 2.53E-46 | Glioma |
| 9473 | THEMIS2 | 2.170393089 | 4.84E-66 | Glioma |
| 9111 | NMI | 2.170974005 | 1.20E-79 | Glioma |
| 4500 | None | 2.173042444 | 2.32E-42 | Glioma |
| 154313 | CFAP206 | 2.173067748 | 3.59E-32 | Glioma |
| 4689 | NCF4 | 2.17405248 | 2.06E-68 | Glioma |
| 3488 | IGFBP5 | 2.17410885 | 9.82E-42 | Glioma |
| 3691 | ITGB4 | 2.174219974 | 2.85E-30 | Glioma |
| 164668 | APOBEC3H | 2.174754094 | 3.48E-51 | Glioma |
| 11065 | UBE2C | 2.176122975 | 1.61E-16 | Glioma |
| 4688 | NCF2 | 2.177335805 | 1.09E-74 | Glioma |
| 5743 | PTGS2 | 2.177396468 | 3.38E-25 | Glioma |
| 951 | CD37 | 2.177648102 | 2.07E-55 | Glioma |
| 4239 | MFAP4 | 2.177890884 | 4.50E-38 | Glioma |
| 4277 | MICB | 2.178008845 | 8.26E-60 | Glioma |
| 9227 | LRAT | 2.180786316 | 9.67E-23 | Glioma |
| 51226 | COPZ2 | 2.180946499 | 7.04E-55 | Glioma |
| 8935 | SKAP2 | 2.181164179 | 1.45E-50 | Glioma |
| 8324 | FZD7 | 2.184554731 | 1.61E-34 | Glioma |
| 283152 | CCDC153 | 2.184900399 | 1.91E-38 | Glioma |
| 3059 | HCLS1 | 2.187484693 | 1.18E-65 | Glioma |
| 81493 | SYNC | 2.18969145 | 9.75E-46 | Glioma |
| 2205 | FCER1A | 2.191085773 | 1.31E-21 | Glioma |
| 199964 | TMEM61 | 2.19277789 | 2.47E-35 | Glioma |
| 51364 | ZMYND10 | 2.193678463 | 5.13E-29 | Glioma |
| 84229 | DRC7 | 2.193718826 | 6.41E-20 | Glioma |
| 2793 | GNGT2 | 2.194775668 | 9.22E-73 | Glioma |
| 8764 | TNFRSF14 | 2.195686866 | 4.88E-87 | Glioma |
| 129804 | FBLN7 | 2.197762919 | 5.51E-24 | Glioma |
| 26509 | MYOF | 2.198450152 | 2.14E-68 | Glioma |
| 873 | CBR1 | 2.198783708 | 1.15E-40 | Glioma |
| 51673 | TPPP3 | 2.199034876 | 1.12E-28 | Glioma |
| 349565 | NMNAT3 | 2.200056098 | 6.10E-30 | Glioma |
| 7050 | TGIF1 | 2.200673451 | 1.03E-54 | Glioma |
| 84617 | TUBB6 | 2.201797922 | 4.47E-58 | Glioma |
| 4599 | MX1 | 2.20371268 | 9.19E-45 | Glioma |
| 641700 | ECSCR | 2.205740762 | 1.82E-39 | Glioma |
| 7096 | TLR1 | 2.207235594 | 2.07E-64 | Glioma |
| 5552 | SRGN | 2.207510491 | 1.46E-69 | Glioma |
| 80216 | ALPK1 | 2.207792092 | 1.40E-72 | Glioma |
| 2028 | ENPEP | 2.209203064 | 1.73E-34 | Glioma |
| 87769 | GGACT | 2.209420554 | 1.77E-59 | Glioma |
| 7127 | TNFAIP2 | 2.209573477 | 5.61E-55 | Glioma |
| 11067 | DEPP1 | 2.210186859 | 7.47E-52 | Glioma |
| 161502 | CFAP161 | 2.210549455 | 1.74E-21 | Glioma |
| 683 | BST1 | 2.213752203 | 1.47E-54 | Glioma |
| 3676 | ITGA4 | 2.213981245 | 6.97E-40 | Glioma |
| 2202 | EFEMP1 | 2.215653688 | 1.39E-36 | Glioma |
| 141 | ADPRH | 2.216267491 | 1.64E-102 | Glioma |
| 3128 | None | 2.218430778 | 2.32E-20 | Glioma |
| 83706 | FERMT3 | 2.218624746 | 7.89E-85 | Glioma |
| 170487 | ACTL10 | 2.219383101 | 1.70E-70 | Glioma |
| 56944 | OLFML3 | 2.221852907 | 1.56E-70 | Glioma |
| 154141 | MBOAT1 | 2.22243816 | 1.53E-57 | Glioma |
| 7205 | TRIP6 | 2.222479402 | 1.58E-79 | Glioma |
| 80122 | MAP3K19 | 2.223163601 | 2.28E-13 | Glioma |
| 602 | BCL3 | 2.223455118 | 7.32E-68 | Glioma |
| 87 | ACTN1 | 2.224898116 | 5.24E-59 | Glioma |
| 400746 | NCMAP | 2.225052034 | 3.12E-21 | Glioma |
| 11328 | FKBP9 | 2.225127651 | 8.45E-66 | Glioma |
| 79025 | FNDC11 | 2.227219032 | 2.78E-36 | Glioma |
| 6503 | SLA | 2.22952382 | 2.05E-62 | Glioma |
| 1847 | DUSP5 | 2.229657533 | 1.60E-36 | Glioma |
| 5157 | PDGFRL | 2.231328085 | 4.30E-38 | Glioma |
| 3075 | CFH | 2.231360911 | 3.69E-53 | Glioma |
| 5959 | RDH5 | 2.232671652 | 4.21E-60 | Glioma |
| 51225 | ABI3 | 2.233776421 | 3.41E-76 | Glioma |
| 493861 | EID3 | 2.234317372 | 4.08E-37 | Glioma |
| 8631 | SKAP1 | 2.234764101 | 5.75E-39 | Glioma |
| 92610 | TIFA | 2.235540391 | 3.43E-80 | Glioma |
| 10252 | SPRY1 | 2.236558757 | 4.98E-50 | Glioma |
| 10346 | TRIM22 | 2.236727738 | 6.92E-77 | Glioma |
| 332 | BIRC5 | 2.237273858 | 3.25E-19 | Glioma |
| 112464 | CAVIN3 | 2.23962509 | 1.68E-41 | Glioma |
| 5341 | PLEK | 2.241307346 | 2.82E-64 | Glioma |
| 414 | ARSD | 2.242502794 | 7.15E-66 | Glioma |
| 132671 | SPATA18 | 2.244523564 | 8.85E-25 | Glioma |
| 971 | CD72 | 2.245875599 | 4.04E-58 | Glioma |
| 3783 | KCNN4 | 2.248829018 | 6.74E-31 | Glioma |
| 8190 | MIA | 2.249389817 | 1.40E-19 | Glioma |
| 945 | CD33 | 2.250215164 | 3.40E-63 | Glioma |
| 1118 | CHIT1 | 2.250733794 | 2.48E-18 | Glioma |
| 5480 | PPIC | 2.25093518 | 2.10E-70 | Glioma |
| 150368 | PHETA2 | 2.252778255 | 1.91E-79 | Glioma |
| 22996 | TTC39A | 2.253129635 | 2.23E-32 | Glioma |
| 7004 | TEAD4 | 2.254082084 | 1.71E-38 | Glioma |
| 1903 | S1PR3 | 2.254653508 | 1.92E-54 | Glioma |
| 353376 | TICAM2 | 2.260257368 | 1.32E-57 | Glioma |
| 8547 | FCN3 | 2.261165403 | 1.05E-27 | Glioma |
| 54739 | XAF1 | 2.262103187 | 4.27E-54 | Glioma |
| 3315 | HSPB1 | 2.264001442 | 8.84E-78 | Glioma |
| 51191 | HERC5 | 2.264215272 | 1.65E-45 | Glioma |
| 1439 | CSF2RB | 2.265966518 | 9.46E-64 | Glioma |
| 6504 | SLAMF1 | 2.26609765 | 1.87E-45 | Glioma |
| 29108 | PYCARD | 2.267004381 | 7.18E-69 | Glioma |
| 3222 | HOXC5 | 2.267190894 | 7.83E-25 | Glioma |
| 26872 | STEAP1 | 2.269078853 | 1.25E-26 | Glioma |
| 3659 | IRF1 | 2.269164579 | 5.34E-82 | Glioma |
| 348807 | CFAP100 | 2.271126945 | 2.24E-18 | Glioma |
| 79930 | DOK3 | 2.271726328 | 9.89E-90 | Glioma |
| 56606 | SLC2A9 | 2.272455516 | 3.42E-56 | Glioma |
| 1396 | CRIP1 | 2.272950249 | 3.10E-51 | Glioma |
| 56241 | SUSD2 | 2.273224177 | 2.21E-55 | Glioma |
| 2921 | CXCL3 | 2.273893681 | 2.74E-21 | Glioma |
| 3140 | MR1 | 2.2739935 | 2.62E-72 | Glioma |
| 64098 | PARVG | 2.276134081 | 2.30E-72 | Glioma |
| 79852 | EPHX3 | 2.277040189 | 8.50E-47 | Glioma |
| 91319 | DERL3 | 2.27738312 | 1.75E-52 | Glioma |
| 5739 | PTGIR | 2.278979806 | 2.30E-49 | Glioma |
| 326342 | None | 2.281365316 | 1.01E-34 | Glioma |
| 6039 | RNASE6 | 2.281575416 | 5.26E-66 | Glioma |
| 29785 | CYP2S1 | 2.282082692 | 1.76E-71 | Glioma |
| 10268 | RAMP3 | 2.28238446 | 2.34E-33 | Glioma |
| 4192 | MDK | 2.283000054 | 2.02E-42 | Glioma |
| 25937 | WWTR1 | 2.284131491 | 1.06E-49 | Glioma |
| 10859 | LILRB1 | 2.286280702 | 2.73E-57 | Glioma |
| 55065 | SLC52A1 | 2.288052068 | 1.51E-42 | Glioma |
| 22915 | MMRN1 | 2.288723542 | 5.52E-36 | Glioma |
| 22797 | TFEC | 2.29090978 | 5.21E-67 | Glioma |
| 8463 | TEAD2 | 2.291443227 | 6.41E-58 | Glioma |
| 3293 | HSD17B3 | 2.292679282 | 1.40E-28 | Glioma |
| 9508 | ADAMTS3 | 2.293735264 | 3.74E-24 | Glioma |
| 25825 | BACE2 | 2.294286906 | 4.06E-77 | Glioma |
| 55423 | SIRPG | 2.296056113 | 4.10E-44 | Glioma |
| 4017 | LOXL2 | 2.296913791 | 1.44E-38 | Glioma |
| 4973 | OLR1 | 2.297186232 | 5.21E-37 | Glioma |
| 7535 | ZAP70 | 2.297301247 | 1.22E-46 | Glioma |
| 9833 | MELK | 2.29797371 | 2.52E-19 | Glioma |
| 6688 | SPI1 | 2.299240465 | 2.97E-76 | Glioma |
| 55282 | LRRC36 | 2.300080542 | 9.32E-34 | Glioma |
| 10312 | TCIRG1 | 2.300583106 | 2.31E-88 | Glioma |
| 942 | CD86 | 2.300599882 | 1.50E-62 | Glioma |
| 864 | RUNX3 | 2.300739316 | 2.24E-59 | Glioma |
| 128346 | C1orf162 | 2.300887364 | 1.41E-63 | Glioma |
| 26074 | CFAP61 | 2.300903119 | 1.20E-38 | Glioma |
| 4070 | TACSTD2 | 2.301466909 | 1.33E-31 | Glioma |
| 3772 | KCNJ15 | 2.304524445 | 2.02E-34 | Glioma |
| 2289 | FKBP5 | 2.305712712 | 2.33E-36 | Glioma |
| 284 | ANGPT1 | 2.305899511 | 5.87E-33 | Glioma |
| 3695 | ITGB7 | 2.309365658 | 1.56E-60 | Glioma |
| 2626 | GATA4 | 2.309841127 | 2.43E-18 | Glioma |
| 634 | CEACAM1 | 2.313358955 | 5.65E-44 | Glioma |
| 968 | CD68 | 2.313485204 | 1.21E-73 | Glioma |
| 284417 | TMEM150B | 2.314018231 | 3.25E-35 | Glioma |
| 83742 | MARVELD1 | 2.315474674 | 5.00E-67 | Glioma |
| 55194 | EVA1B | 2.316889905 | 1.62E-65 | Glioma |
| 4064 | CD180 | 2.317081857 | 3.84E-61 | Glioma |
| 127795 | C1orf87 | 2.317563097 | 2.11E-25 | Glioma |
| 166929 | SGMS2 | 2.318519995 | 3.53E-60 | Glioma |
| 4605 | MYBL2 | 2.319966109 | 4.61E-17 | Glioma |
| 6241 | RRM2 | 2.321755502 | 1.14E-19 | Glioma |
| 1535 | CYBA | 2.322325103 | 2.75E-88 | Glioma |
| 1770 | DNAH9 | 2.32449462 | 8.11E-38 | Glioma |
| 79733 | E2F8 | 2.324972073 | 3.64E-29 | Glioma |
| 11035 | RIPK3 | 2.32729605 | 1.27E-78 | Glioma |
| 29113 | C6orf15 | 2.327681419 | 7.88E-17 | Glioma |
| 2348 | FOLR1 | 2.328148499 | 6.75E-28 | Glioma |
| 586 | BCAT1 | 2.328797921 | 1.59E-38 | Glioma |
| 284119 | CAVIN1 | 2.333743246 | 5.13E-72 | Glioma |
| 92689 | FAM114A1 | 2.334481552 | 7.25E-63 | Glioma |
| 9955 | HS3ST3A1 | 2.334894904 | 1.84E-24 | Glioma |
| 84722 | PSRC1 | 2.336348919 | 1.00E-65 | Glioma |
| 355 | FAS | 2.336822485 | 1.45E-67 | Glioma |
| 1230 | CCR1 | 2.338282965 | 7.03E-62 | Glioma |
| 79819 | WDR78 | 2.34000529 | 3.12E-49 | Glioma |
| 144501 | KRT80 | 2.340300311 | 1.42E-31 | Glioma |
| 84217 | ZMYND12 | 2.340450862 | 2.07E-38 | Glioma |
| 83853 | ROPN1L | 2.341538951 | 2.44E-22 | Glioma |
| 8542 | APOL1 | 2.341960784 | 1.92E-58 | Glioma |
| 29126 | CD274 | 2.343096035 | 4.60E-40 | Glioma |
| 3227 | HOXC11 | 2.343571383 | 3.69E-22 | Glioma |
| 93663 | ARHGAP18 | 2.343910642 | 8.50E-84 | Glioma |
| 266675 | BEST4 | 2.344960526 | 1.79E-39 | Glioma |
| 126014 | OSCAR | 2.348191985 | 6.46E-81 | Glioma |
| 579 | NKX3-2 | 2.34871042 | 6.05E-25 | Glioma |
| 54360 | CYTL1 | 2.349794182 | 1.76E-42 | Glioma |
| 283385 | MORN3 | 2.350559356 | 1.52E-30 | Glioma |
| 57619 | SHROOM3 | 2.350747676 | 5.99E-36 | Glioma |
| 84868 | HAVCR2 | 2.350873038 | 1.43E-67 | Glioma |
| 115761 | ARL11 | 2.351280856 | 9.69E-75 | Glioma |
| 1489 | CTF1 | 2.351320653 | 2.26E-64 | Glioma |
| 81622 | UNC93B1 | 2.351748301 | 1.03E-87 | Glioma |
| 100188953 | None | 2.352504423 | 1.09E-33 | Glioma |
| 115004 | CGAS | 2.353962072 | 4.27E-71 | Glioma |
| 3082 | HGF | 2.354058243 | 1.03E-34 | Glioma |
| 54097 | FAM3B | 2.355524273 | 1.35E-31 | Glioma |
| 2888 | GRB14 | 2.356428599 | 1.00E-19 | Glioma |
| 80342 | TRAF3IP3 | 2.356694191 | 7.67E-67 | Glioma |
| 84680 | ACCS | 2.357616068 | 3.26E-46 | Glioma |
| 4489 | MT1A | 2.358439025 | 1.65E-28 | Glioma |
| 1066 | CES1 | 2.359177212 | 3.32E-20 | Glioma |
| 133923 | ZNF474 | 2.360546446 | 2.66E-32 | Glioma |
| 22918 | CD93 | 2.360624855 | 5.06E-47 | Glioma |
| 284759 | SIRPB2 | 2.361563969 | 9.25E-62 | Glioma |
| 55165 | CEP55 | 2.363229539 | 1.25E-26 | Glioma |
| 440836 | ODF3B | 2.366741264 | 7.46E-44 | Glioma |
| 1842 | ECM2 | 2.367282762 | 2.78E-48 | Glioma |
| 921 | CD5 | 2.368276374 | 8.87E-43 | Glioma |
| 80310 | PDGFD | 2.369296012 | 6.71E-25 | Glioma |
| 401944 | LDLRAD2 | 2.369644261 | 2.23E-48 | Glioma |
| 80131 | LRRC8E | 2.369923024 | 2.94E-32 | Glioma |
| 219285 | SAMD9L | 2.371784436 | 8.73E-71 | Glioma |
| 10232 | MSLN | 2.372456188 | 7.09E-21 | Glioma |
| 10791 | VAMP5 | 2.373338805 | 2.35E-83 | Glioma |
| 347902 | AMIGO2 | 2.374437189 | 1.64E-42 | Glioma |
| 8645 | KCNK5 | 2.375626875 | 1.27E-28 | Glioma |
| 158158 | RASEF | 2.376099189 | 3.04E-24 | Glioma |
| 2645 | GCK | 2.376854125 | 1.02E-35 | Glioma |
| 7805 | LAPTM5 | 2.377284728 | 1.70E-74 | Glioma |
| 963 | CD53 | 2.38190502 | 2.55E-72 | Glioma |
| 2204 | FCAR | 2.382387363 | 2.50E-31 | Glioma |
| 7132 | TNFRSF1A | 2.384812006 | 8.29E-87 | Glioma |
| 430 | ASCL2 | 2.385387956 | 3.37E-69 | Glioma |
| 11314 | CD300A | 2.385834646 | 1.10E-78 | Glioma |
| 84282 | RNF135 | 2.3863586 | 1.94E-95 | Glioma |
| 7852 | CXCR4 | 2.387212125 | 6.49E-70 | Glioma |
| 5788 | PTPRC | 2.389231926 | 7.94E-63 | Glioma |
| 57212 | None | 2.39014639 | 6.63E-64 | Glioma |
| 140876 | RIPOR3 | 2.39088268 | 1.39E-43 | Glioma |
| 100049587 | SIGLEC14 | 2.390934913 | 9.51E-39 | Glioma |
| 286204 | CRB2 | 2.392842078 | 2.53E-35 | Glioma |
| 339768 | ESPNL | 2.393770301 | 4.09E-27 | Glioma |
| 64114 | TMBIM1 | 2.395096235 | 1.14E-94 | Glioma |
| 9902 | None | 2.397414667 | 1.05E-65 | Glioma |
| 54440 | SASH3 | 2.398169295 | 7.08E-76 | Glioma |
| 284276 | None | 2.400579749 | 5.12E-29 | Glioma |
| 283897 | C16orf54 | 2.403676289 | 1.47E-69 | Glioma |
| 63910 | SLC17A9 | 2.403722003 | 1.07E-66 | Glioma |
| 4166 | CHST6 | 2.408300493 | 5.75E-46 | Glioma |
| 4233 | MET | 2.408461416 | 7.71E-20 | Glioma |
| 259307 | IL4I1 | 2.410138211 | 9.34E-71 | Glioma |
| 4068 | SH2D1A | 2.410434541 | 7.89E-47 | Glioma |
| 27129 | HSPB7 | 2.410589299 | 1.48E-39 | Glioma |
| 123 | PLIN2 | 2.411046353 | 1.65E-61 | Glioma |
| 285 | ANGPT2 | 2.411516752 | 2.58E-36 | Glioma |
| 7056 | THBD | 2.412101739 | 4.90E-48 | Glioma |
| 153643 | FAM81B | 2.412491019 | 7.38E-17 | Glioma |
| 9056 | SLC7A7 | 2.415254505 | 1.75E-80 | Glioma |
| 3002 | GZMB | 2.416724285 | 3.76E-37 | Glioma |
| 140578 | CHODL | 2.41729051 | 1.47E-26 | Glioma |
| 2810 | SFN | 2.417748893 | 5.67E-32 | Glioma |
| 3875 | KRT18 | 2.418767822 | 2.94E-32 | Glioma |
| 4939 | OAS2 | 2.420291679 | 3.42E-58 | Glioma |
| 6590 | SLPI | 2.420760376 | 7.51E-25 | Glioma |
| 991 | CDC20 | 2.421006851 | 4.79E-28 | Glioma |
| 4920 | ROR2 | 2.421084241 | 3.91E-30 | Glioma |
| 433 | ASGR2 | 2.421105531 | 6.40E-45 | Glioma |
| 64332 | NFKBIZ | 2.423097168 | 5.50E-62 | Glioma |
| 247 | ALOX15B | 2.425025732 | 1.51E-18 | Glioma |
| 6490 | PMEL | 2.428354536 | 7.33E-46 | Glioma |
| 29909 | GPR171 | 2.428836369 | 4.09E-50 | Glioma |
| 83888 | FGFBP2 | 2.431282285 | 1.75E-24 | Glioma |
| 54829 | ASPN | 2.432232212 | 6.45E-36 | Glioma |
| 4267 | CD99 | 2.432737256 | 9.30E-48 | Glioma |
| 3205 | HOXA9 | 2.436520214 | 1.27E-20 | Glioma |
| 116832 | RPL39L | 2.437585568 | 6.97E-36 | Glioma |
| 10788 | IQGAP2 | 2.437778447 | 1.09E-37 | Glioma |
| 3779 | KCNMB1 | 2.439650416 | 8.70E-60 | Glioma |
| 55509 | BATF3 | 2.439733079 | 1.46E-51 | Glioma |
| 2919 | CXCL1 | 2.440082783 | 9.08E-27 | Glioma |
| 79611 | ACSS3 | 2.440221212 | 1.16E-39 | Glioma |
| 80329 | ULBP1 | 2.44124469 | 3.09E-26 | Glioma |
| 340205 | TREML1 | 2.442340357 | 1.21E-50 | Glioma |
| 10875 | FGL2 | 2.44260347 | 2.08E-69 | Glioma |
| 348174 | CLEC18A | 2.443473829 | 5.86E-50 | Glioma |
| 255231 | MCOLN2 | 2.44491907 | 1.28E-36 | Glioma |
| 353345 | GPR141 | 2.445505867 | 1.81E-47 | Glioma |
| 3718 | JAK3 | 2.445742863 | 1.31E-49 | Glioma |
| 134121 | C5orf49 | 2.446242645 | 1.94E-19 | Glioma |
| 100270710 | None | 2.447743437 | 2.66E-67 | Glioma |
| 55268 | ECHDC2 | 2.448121259 | 1.70E-57 | Glioma |
| 3229 | HOXC13 | 2.448435957 | 8.79E-19 | Glioma |
| 10516 | FBLN5 | 2.449133339 | 1.44E-53 | Glioma |
| 157506 | RDH10 | 2.449251244 | 1.13E-57 | Glioma |
| 1992 | SERPINB1 | 2.45187007 | 5.93E-100 | Glioma |
| 7538 | None | 2.452993697 | 4.42E-54 | Glioma |
| 2215 | FCGR3B | 2.453301998 | 6.68E-31 | Glioma |
| 283208 | P4HA3 | 2.453759896 | 1.93E-39 | Glioma |
| 152195 | None | 2.455319046 | 2.52E-73 | Glioma |
| 202333 | CMYA5 | 2.45872773 | 7.70E-41 | Glioma |
| 84941 | HSH2D | 2.458797146 | 8.00E-55 | Glioma |
| 79962 | DNAJC22 | 2.45915217 | 1.34E-62 | Glioma |
| 1041 | CDSN | 2.460832767 | 1.83E-34 | Glioma |
| 414236 | C10orf55 | 2.461909196 | 1.03E-45 | Glioma |
| 841 | CASP8 | 2.462012388 | 1.60E-103 | Glioma |
| 3683 | ITGAL | 2.462541977 | 1.72E-59 | Glioma |
| 3371 | TNC | 2.462640056 | 7.67E-44 | Glioma |
| 10112 | KIF20A | 2.464453838 | 6.50E-21 | Glioma |
| 284467 | FAM19A3 | 2.470828596 | 2.64E-36 | Glioma |
| 6277 | S100A6 | 2.470889125 | 2.43E-79 | Glioma |
| 10870 | HCST | 2.47095368 | 1.60E-65 | Glioma |
| 5251 | PHEX | 2.471911925 | 3.09E-35 | Glioma |
| 3579 | CXCR2 | 2.473004407 | 4.65E-47 | Glioma |
| 9609 | RAB36 | 2.473165249 | 9.30E-37 | Glioma |
| 54209 | TREM2 | 2.474302157 | 4.87E-58 | Glioma |
| 54 | ACP5 | 2.478923582 | 7.53E-36 | Glioma |
| 7422 | VEGFA | 2.480087365 | 1.79E-36 | Glioma |
| 2635 | GBP3 | 2.485198811 | 2.24E-36 | Glioma |
| 283314 | None | 2.485235858 | 2.51E-58 | Glioma |
| 84267 | C9orf64 | 2.485298438 | 1.27E-62 | Glioma |
| 348378 | SHISAL2A | 2.486640465 | 7.96E-58 | Glioma |
| 3491 | CCN1 | 2.4887083 | 8.54E-36 | Glioma |
| 1075 | CTSC | 2.48906441 | 3.40E-73 | Glioma |
| 684 | BST2 | 2.489205278 | 1.83E-71 | Glioma |
| 84073 | MYCBPAP | 2.490135456 | 4.54E-37 | Glioma |
| 5742 | PTGS1 | 2.490884502 | 3.35E-70 | Glioma |
| 9547 | CXCL14 | 2.491806882 | 5.41E-20 | Glioma |
| 81932 | HDHD3 | 2.495109458 | 3.23E-53 | Glioma |
| 79792 | GSDMD | 2.496133911 | 1.52E-96 | Glioma |
| 7305 | TYROBP | 2.49756417 | 8.02E-80 | Glioma |
| 3689 | ITGB2 | 2.498034914 | 4.89E-74 | Glioma |
| 10404 | CPQ | 2.504947215 | 3.32E-83 | Glioma |
| 375307 | CATIP | 2.507085773 | 1.62E-31 | Glioma |
| 79669 | C3orf52 | 2.507577366 | 1.89E-38 | Glioma |
| 467 | ATF3 | 2.509430316 | 6.04E-57 | Glioma |
| 10866 | None | 2.50975301 | 2.23E-69 | Glioma |
| 970 | None | 2.509777354 | 1.91E-25 | Glioma |
| 5148 | None | 2.510033947 | 1.00E-61 | Glioma |
| 83596 | BCL2L12 | 2.510350324 | 9.00E-72 | Glioma |
| 10008 | KCNE3 | 2.510878054 | 6.61E-74 | Glioma |
| 6385 | SDC4 | 2.512038864 | 5.99E-36 | Glioma |
| 83987 | CCDC8 | 2.51286271 | 8.39E-41 | Glioma |
| 1520 | CTSS | 2.515374828 | 3.08E-78 | Glioma |
| 10673 | TNFSF13B | 2.515863647 | 6.66E-34 | Glioma |
| 145376 | PPP1R36 | 2.516972307 | 7.62E-40 | Glioma |
| 3553 | IL1B | 2.517232789 | 1.42E-27 | Glioma |
| 714 | C1QC | 2.518288147 | 2.53E-77 | Glioma |
| 29842 | TFCP2L1 | 2.518376675 | 8.61E-29 | Glioma |
| 5836 | PYGL | 2.518416272 | 4.83E-78 | Glioma |
| 3606 | IL18 | 2.518745175 | 2.23E-70 | Glioma |
| 374 | AREG | 2.520098892 | 2.17E-25 | Glioma |
| 93010 | B3GNT7 | 2.520586383 | 3.26E-52 | Glioma |
| 59271 | EVA1C | 2.520826679 | 4.06E-79 | Glioma |
| 1236 | CCR7 | 2.521064019 | 1.26E-41 | Glioma |
| 2152 | F3 | 2.52237371 | 2.38E-56 | Glioma |
| 11061 | CNMD | 2.523033153 | 4.51E-22 | Glioma |
| 1378 | CR1 | 2.525096929 | 1.03E-26 | Glioma |
| 10410 | IFITM3 | 2.528113227 | 1.13E-77 | Glioma |
| 654816 | None | 2.528904169 | 5.25E-60 | Glioma |
| 10135 | NAMPT | 2.529206186 | 1.07E-56 | Glioma |
| 81788 | NUAK2 | 2.530390502 | 2.38E-75 | Glioma |
| 974 | CD79B | 2.531920549 | 2.95E-50 | Glioma |
| 10481 | HOXB13 | 2.53377758 | 3.26E-19 | Glioma |
| 944 | TNFSF8 | 2.534763946 | 4.72E-56 | Glioma |
| 56892 | TCIM | 2.53516415 | 6.71E-47 | Glioma |
| 170679 | PSORS1C1 | 2.535666239 | 7.06E-39 | Glioma |
| 8835 | SOCS2 | 2.538520741 | 1.61E-45 | Glioma |
| 50615 | IL21R | 2.545360008 | 2.51E-29 | Glioma |
| 6038 | RNASE4 | 2.54616677 | 2.13E-68 | Glioma |
| 11167 | FSTL1 | 2.547331638 | 1.79E-85 | Glioma |
| 2625 | GATA3 | 2.548035937 | 1.03E-34 | Glioma |
| 23704 | KCNE4 | 2.548404388 | 1.36E-50 | Glioma |
| 2000 | ELF4 | 2.548898036 | 4.37E-79 | Glioma |
| 27071 | DAPP1 | 2.549900732 | 2.29E-64 | Glioma |
| 5732 | PTGER2 | 2.551662616 | 1.12E-52 | Glioma |
| 89886 | SLAMF9 | 2.551800845 | 1.37E-27 | Glioma |
| 23480 | SEC61G | 2.551910002 | 1.19E-31 | Glioma |
| 29774 | None | 2.555122033 | 2.79E-46 | Glioma |
| 58472 | SQOR | 2.556340556 | 4.07E-107 | Glioma |
| 6692 | SPINT1 | 2.556415367 | 2.33E-69 | Glioma |
| 441054 | C4orf47 | 2.561074757 | 5.07E-48 | Glioma |
| 1261 | CNGA3 | 2.56360908 | 6.02E-28 | Glioma |
| 160365 | CLECL1 | 2.564454031 | 5.06E-46 | Glioma |
| 83938 | LRMDA | 2.57158313 | 2.42E-68 | Glioma |
| 3112 | HLA-DOB | 2.573633885 | 2.46E-47 | Glioma |
| 6615 | SNAI1 | 2.576507811 | 3.11E-50 | Glioma |
| 144455 | E2F7 | 2.577661025 | 4.22E-31 | Glioma |
| 9787 | DLGAP5 | 2.577720169 | 9.74E-21 | Glioma |
| 55970 | GNG12 | 2.57913983 | 4.16E-52 | Glioma |
| 1366 | CLDN7 | 2.582031359 | 5.50E-60 | Glioma |
| 712 | C1QA | 2.582098968 | 2.50E-74 | Glioma |
| 27350 | APOBEC3C | 2.58277273 | 4.32E-81 | Glioma |
| 10871 | CD300C | 2.585950735 | 1.09E-66 | Glioma |
| 55214 | P3H2 | 2.586015202 | 2.01E-70 | Glioma |
| 55635 | DEPDC1 | 2.58608263 | 7.89E-29 | Glioma |
| 3224 | None | 2.588674406 | 1.74E-26 | Glioma |
| 55024 | BANK1 | 2.591285124 | 5.12E-34 | Glioma |
| 128344 | PIFO | 2.593460108 | 1.33E-48 | Glioma |
| 54504 | CPVL | 2.594948624 | 1.62E-68 | Glioma |
| 200316 | APOBEC3F | 2.595195637 | 3.60E-91 | Glioma |
| 8673 | VAMP8 | 2.595517951 | 1.20E-85 | Glioma |
| 1058 | CENPA | 2.595831824 | 5.45E-29 | Glioma |
| 399949 | C11orf88 | 2.597788501 | 3.33E-24 | Glioma |
| 93349 | SP140L | 2.59869001 | 1.06E-96 | Glioma |
| 5570 | PKIB | 2.603353887 | 6.30E-39 | Glioma |
| 940 | CD28 | 2.60415536 | 5.47E-45 | Glioma |
| 117854 | TRIM6 | 2.604361388 | 5.69E-68 | Glioma |
| 132946 | ARL9 | 2.604472164 | 1.51E-33 | Glioma |
| 284021 | MILR1 | 2.604517097 | 2.44E-65 | Glioma |
| 3574 | IL7 | 2.605977717 | 3.42E-62 | Glioma |
| 9976 | CLEC2B | 2.607001881 | 6.63E-71 | Glioma |
| 10462 | CLEC10A | 2.61205813 | 4.23E-28 | Glioma |
| 9119 | KRT75 | 2.613025365 | 2.16E-25 | Glioma |
| 1296 | COL8A2 | 2.613399652 | 9.82E-57 | Glioma |
| 2020 | EN2 | 2.614275555 | 1.36E-34 | Glioma |
| 5031 | P2RY6 | 2.616141123 | 2.97E-69 | Glioma |
| 3678 | ITGA5 | 2.6185607 | 1.25E-74 | Glioma |
| 1880 | GPR183 | 2.62307907 | 6.50E-60 | Glioma |
| 646424 | SPINK8 | 2.623368622 | 8.50E-38 | Glioma |
| 6672 | SP100 | 2.624318532 | 7.28E-119 | Glioma |
| 125111 | GJD3 | 2.624334537 | 1.92E-38 | Glioma |
| 6372 | CXCL6 | 2.629000622 | 9.49E-24 | Glioma |
| 1675 | CFD | 2.631512373 | 6.30E-55 | Glioma |
| 1290 | COL5A2 | 2.632265993 | 1.15E-49 | Glioma |
| 653333 | FAM86B2 | 2.634536218 | 3.19E-40 | Glioma |
| 25878 | MXRA5 | 2.634624503 | 5.55E-39 | Glioma |
| 3956 | LGALS1 | 2.636691204 | 6.22E-75 | Glioma |
| 3552 | IL1A | 2.639397574 | 1.22E-41 | Glioma |
| 79465 | ULBP3 | 2.639488663 | 5.31E-40 | Glioma |
| 54103 | GSAP | 2.641384004 | 3.76E-75 | Glioma |
| 286530 | P2RY8 | 2.641836338 | 4.90E-46 | Glioma |
| 4117 | MAK | 2.643257046 | 4.24E-45 | Glioma |
| 389799 | CFAP77 | 2.643419914 | 1.30E-26 | Glioma |
| 85027 | SMIM3 | 2.643824435 | 6.21E-71 | Glioma |
| 81031 | SLC2A10 | 2.644293389 | 1.86E-58 | Glioma |
| 1089 | CEACAM4 | 2.647021763 | 9.31E-49 | Glioma |
| 145781 | GCOM1 | 2.649838881 | 3.17E-42 | Glioma |
| 5734 | PTGER4 | 2.650359428 | 4.39E-68 | Glioma |
| 3903 | LAIR1 | 2.651086568 | 2.96E-82 | Glioma |
| 2302 | FOXJ1 | 2.652796287 | 2.40E-38 | Glioma |
| 838 | CASP5 | 2.653670534 | 4.16E-56 | Glioma |
| 100124700 | None | 2.654027028 | 6.09E-24 | Glioma |
| 1306 | COL15A1 | 2.658860386 | 4.53E-36 | Glioma |
| 387885 | CFAP73 | 2.658874834 | 2.29E-28 | Glioma |
| 2209 | FCGR1A | 2.659410858 | 5.96E-58 | Glioma |
| 4938 | OAS1 | 2.659540131 | 4.83E-63 | Glioma |
| 8740 | TNFSF14 | 2.659788495 | 2.68E-47 | Glioma |
| 374403 | TBC1D10C | 2.660147072 | 1.13E-85 | Glioma |
| 5918 | RARRES1 | 2.662595311 | 7.26E-35 | Glioma |
| 201799 | TMEM154 | 2.667452806 | 3.55E-83 | Glioma |
| 64843 | ISL2 | 2.671689687 | 9.80E-30 | Glioma |
| 126393 | HSPB6 | 2.675108036 | 9.23E-47 | Glioma |
| 80258 | EFHC2 | 2.67568874 | 3.69E-32 | Glioma |
| 941 | CD80 | 2.67647822 | 1.16E-53 | Glioma |
| 5359 | PLSCR1 | 2.677864965 | 7.26E-107 | Glioma |
| 5820 | None | 2.683661063 | 6.50E-43 | Glioma |
| 3216 | HOXB6 | 2.684876042 | 1.47E-30 | Glioma |
| 83659 | TEKT1 | 2.68503289 | 3.12E-22 | Glioma |
| 54502 | RBM47 | 2.687917346 | 5.80E-83 | Glioma |
| 871 | SERPINH1 | 2.690772791 | 8.63E-70 | Glioma |
| 240 | ALOX5 | 2.69181678 | 1.07E-77 | Glioma |
| 11326 | VSIG4 | 2.694343873 | 7.23E-52 | Glioma |
| 120425 | JAML | 2.698694889 | 1.17E-39 | Glioma |
| 255743 | NPNT | 2.699867111 | 8.69E-36 | Glioma |
| 140564 | APOBEC3D | 2.703881631 | 1.23E-83 | Glioma |
| 5327 | PLAT | 2.706292827 | 8.08E-51 | Glioma |
| 6273 | S100A2 | 2.706776046 | 3.91E-55 | Glioma |
| 929 | CD14 | 2.707539067 | 3.09E-66 | Glioma |
| 416 | ARSF | 2.709068851 | 1.12E-24 | Glioma |
| 3108 | HLA-DMA | 2.709903373 | 1.17E-89 | Glioma |
| 94240 | EPSTI1 | 2.710733783 | 4.04E-79 | Glioma |
| 7136 | TNNI2 | 2.711317657 | 6.03E-59 | Glioma |
| 80332 | ADAM33 | 2.716229327 | 2.41E-40 | Glioma |
| 9953 | HS3ST3B1 | 2.716552047 | 2.66E-34 | Glioma |
| 79949 | PLEKHS1 | 2.717685205 | 2.78E-27 | Glioma |
| 3162 | HMOX1 | 2.718863138 | 4.12E-72 | Glioma |
| 123036 | TC2N | 2.719909494 | 6.55E-38 | Glioma |
| 6999 | TDO2 | 2.720508995 | 2.58E-27 | Glioma |
| 2634 | GBP2 | 2.72222233 | 1.68E-53 | Glioma |
| 55113 | XKR8 | 2.727578623 | 6.53E-83 | Glioma |
| 80183 | RUBCNL | 2.728675023 | 1.46E-72 | Glioma |
| 170825 | GSX2 | 2.729952564 | 2.25E-29 | Glioma |
| 136332 | LRGUK | 2.730284049 | 3.27E-47 | Glioma |
| 126820 | WDR63 | 2.730796453 | 1.59E-27 | Glioma |
| 713 | C1QB | 2.732417996 | 8.42E-80 | Glioma |
| 348013 | TMEM255B | 2.732475186 | 8.73E-77 | Glioma |
| 4323 | MMP14 | 2.735742591 | 9.20E-67 | Glioma |
| 2012 | EMP1 | 2.735914855 | 1.29E-53 | Glioma |
| 79998 | ANKRD53 | 2.738505345 | 1.37E-56 | Glioma |
| 79258 | MMEL1 | 2.740287294 | 1.73E-28 | Glioma |
| 126364 | LRRC25 | 2.74114701 | 5.34E-88 | Glioma |
| 2999 | GZMH | 2.741847202 | 2.38E-45 | Glioma |
| 131578 | LRRC15 | 2.743137004 | 2.55E-28 | Glioma |
| 6768 | ST14 | 2.744231531 | 6.80E-54 | Glioma |
| 137075 | CLDN23 | 2.751775403 | 7.75E-71 | Glioma |
| 165631 | PARP15 | 2.754156801 | 8.84E-58 | Glioma |
| 10981 | RAB32 | 2.754377998 | 1.40E-82 | Glioma |
| 9620 | CELSR1 | 2.756623186 | 1.85E-36 | Glioma |
| 220 | ALDH1A3 | 2.759350498 | 8.16E-20 | Glioma |
| 6588 | SLN | 2.759717101 | 2.32E-21 | Glioma |
| 81030 | ZBP1 | 2.761312243 | 5.03E-44 | Glioma |
| 51338 | MS4A4A | 2.767700531 | 6.77E-61 | Glioma |
| 200162 | SPAG17 | 2.769653133 | 8.14E-26 | Glioma |
| 3127 | HLA-DRB5 | 2.770603197 | 7.19E-42 | Glioma |
| 1284 | COL4A2 | 2.771687975 | 1.28E-49 | Glioma |
| 5118 | PCOLCE | 2.772397322 | 1.76E-48 | Glioma |
| 23643 | LY96 | 2.777018174 | 1.65E-70 | Glioma |
| 79642 | ARSJ | 2.779253295 | 1.86E-44 | Glioma |
| 6649 | SOD3 | 2.779385754 | 2.71E-51 | Glioma |
| 924 | CD7 | 2.779641225 | 1.46E-75 | Glioma |
| 2591 | GALNT3 | 2.780323888 | 3.49E-49 | Glioma |
| 8876 | VNN1 | 2.781842617 | 2.19E-54 | Glioma |
| 834 | CASP1 | 2.781901308 | 3.08E-96 | Glioma |
| 7005 | TEAD3 | 2.783438504 | 1.67E-72 | Glioma |
| 861 | RUNX1 | 2.786281456 | 6.75E-63 | Glioma |
| 10417 | SPON2 | 2.78723803 | 1.53E-40 | Glioma |
| 352909 | DNAAF3 | 2.788176803 | 8.35E-38 | Glioma |
| 7044 | LEFTY2 | 2.792594744 | 5.97E-23 | Glioma |
| 1043 | CD52 | 2.793292082 | 3.30E-52 | Glioma |
| 7378 | UPP1 | 2.793621021 | 4.08E-83 | Glioma |
| 57664 | PLEKHA4 | 2.79387751 | 3.82E-60 | Glioma |
| 6004 | RGS16 | 2.794300754 | 2.07E-54 | Glioma |
| 170692 | ADAMTS18 | 2.79677544 | 5.11E-30 | Glioma |
| 7980 | TFPI2 | 2.80970497 | 1.75E-27 | Glioma |
| 9595 | CYTIP | 2.811681874 | 1.70E-83 | Glioma |
| 388325 | SCIMP | 2.815077385 | 2.14E-70 | Glioma |
| 7042 | TGFB2 | 2.815460667 | 8.62E-55 | Glioma |
| 79883 | PODNL1 | 2.817320307 | 9.89E-44 | Glioma |
| 23682 | RAB38 | 2.819089985 | 2.21E-63 | Glioma |
| 285966 | TCAF2 | 2.821171292 | 3.19E-64 | Glioma |
| 400823 | FAM177B | 2.824805593 | 3.93E-49 | Glioma |
| 9744 | ACAP1 | 2.829502478 | 2.37E-79 | Glioma |
| 9046 | DOK2 | 2.830227058 | 3.94E-53 | Glioma |
| 330 | BIRC3 | 2.831926578 | 3.84E-53 | Glioma |
| 3575 | IL7R | 2.837415272 | 2.84E-40 | Glioma |
| 57010 | CABP4 | 2.838132333 | 1.27E-78 | Glioma |
| 84935 | MEDAG | 2.839346571 | 1.76E-30 | Glioma |
| 4320 | MMP11 | 2.841102837 | 2.34E-49 | Glioma |
| 6648 | SOD2 | 2.842025307 | 3.48E-72 | Glioma |
| 3077 | HFE | 2.846524879 | 1.31E-84 | Glioma |
| 84002 | B3GNT5 | 2.8467412 | 5.09E-88 | Glioma |
| 4478 | MSN | 2.850140319 | 1.81E-107 | Glioma |
| 3820 | KLRB1 | 2.852508518 | 1.37E-47 | Glioma |
| 79841 | AGBL2 | 2.858482454 | 2.27E-46 | Glioma |
| 57124 | CD248 | 2.86025661 | 2.25E-51 | Glioma |
| 29984 | RHOD | 2.862591595 | 9.40E-37 | Glioma |
| 154761 | None | 2.863461862 | 9.12E-55 | Glioma |
| 10265 | IRX5 | 2.87000781 | 3.64E-27 | Glioma |
| 124599 | CD300LB | 2.87367469 | 1.18E-69 | Glioma |
| 27074 | LAMP3 | 2.873695622 | 1.10E-59 | Glioma |
| 399 | RHOH | 2.8737481 | 1.07E-76 | Glioma |
| 3240 | HP | 2.877796436 | 8.32E-27 | Glioma |
| 2207 | FCER1G | 2.877812301 | 1.67E-87 | Glioma |
| 718 | C3 | 2.878865243 | 3.58E-61 | Glioma |
| 3486 | IGFBP3 | 2.883821686 | 1.08E-35 | Glioma |
| 25816 | TNFAIP8 | 2.885565906 | 2.21E-93 | Glioma |
| 128153 | SPATA17 | 2.885612779 | 2.00E-51 | Glioma |
| 285386 | TPRG1 | 2.885661788 | 2.72E-77 | Glioma |
| 10578 | GNLY | 2.88812624 | 9.51E-42 | Glioma |
| 4007 | PRICKLE3 | 2.889035021 | 6.07E-101 | Glioma |
| 1052 | CEBPD | 2.892127867 | 4.86E-73 | Glioma |
| 9123 | SLC16A3 | 2.893312898 | 3.16E-88 | Glioma |
| 64866 | CDCP1 | 2.89426928 | 1.71E-72 | Glioma |
| 344 | APOC2 | 2.900724889 | 3.56E-59 | Glioma |
| 7097 | TLR2 | 2.902606465 | 1.97E-74 | Glioma |
| 10288 | LILRB2 | 2.90282068 | 1.89E-71 | Glioma |
| 2706 | GJB2 | 2.903419116 | 2.95E-27 | Glioma |
| 80307 | None | 2.906843813 | 3.42E-38 | Glioma |
| 30008 | EFEMP2 | 2.910971999 | 1.83E-77 | Glioma |
| 6355 | CCL8 | 2.916105432 | 6.90E-34 | Glioma |
| 717 | C2 | 2.917087547 | 1.10E-78 | Glioma |
| 11015 | KDELR3 | 2.918538345 | 8.52E-59 | Glioma |
| 146722 | CD300LF | 2.919931848 | 1.99E-74 | Glioma |
| 30817 | ADGRE2 | 2.920426665 | 2.44E-82 | Glioma |
| 3561 | IL2RG | 2.922219147 | 1.22E-79 | Glioma |
| 2203 | FBP1 | 2.923919996 | 6.14E-82 | Glioma |
| 5133 | PDCD1 | 2.926044444 | 1.85E-60 | Glioma |
| 2833 | CXCR3 | 2.929713971 | 1.30E-51 | Glioma |
| 27287 | VENTX | 2.930096297 | 2.22E-58 | Glioma |
| 11185 | INMT | 2.934702346 | 6.36E-45 | Glioma |
| 283 | ANG | 2.935205792 | 8.92E-84 | Glioma |
| 3669 | ISG20 | 2.936448106 | 1.50E-88 | Glioma |
| 960 | CD44 | 2.937486684 | 2.41E-59 | Glioma |
| 116496 | FAM129A | 2.937901177 | 2.08E-82 | Glioma |
| 115290 | FBXO17 | 2.94466569 | 1.00E-50 | Glioma |
| 5880 | RAC2 | 2.944897385 | 3.66E-96 | Glioma |
| 5077 | PAX3 | 2.945987238 | 1.15E-25 | Glioma |
| 55034 | MOCOS | 2.949433186 | 1.01E-51 | Glioma |
| 440585 | FAM183A | 2.951232987 | 1.05E-27 | Glioma |
| 115908 | CTHRC1 | 2.952756431 | 1.36E-39 | Glioma |
| 3109 | HLA-DMB | 2.953019543 | 5.44E-87 | Glioma |
| 3383 | ICAM1 | 2.953054172 | 3.90E-74 | Glioma |
| 10537 | UBD | 2.954215693 | 5.31E-43 | Glioma |
| 60489 | APOBEC3G | 2.956227785 | 3.24E-101 | Glioma |
| 3620 | IDO1 | 2.95623757 | 3.86E-34 | Glioma |
| 55365 | TMEM176A | 2.956741123 | 1.66E-64 | Glioma |
| 64581 | CLEC7A | 2.956867509 | 2.17E-71 | Glioma |
| 202 | CRYBG1 | 2.957843986 | 7.87E-62 | Glioma |
| 54970 | TTC12 | 2.959413693 | 7.71E-51 | Glioma |
| 29015 | SLC43A3 | 2.964804094 | 4.55E-83 | Glioma |
| 171558 | PTCRA | 2.966148954 | 2.45E-52 | Glioma |
| 7431 | VIM | 2.968793911 | 3.74E-63 | Glioma |
| 358 | AQP1 | 2.968880009 | 1.03E-34 | Glioma |
| 90187 | EMILIN3 | 2.9707796 | 6.59E-23 | Glioma |
| 3225 | None | 2.972357577 | 6.83E-34 | Glioma |
| 9674 | KIAA0040 | 2.976703666 | 1.13E-81 | Glioma |
| 116039 | OSR2 | 2.977774611 | 6.39E-29 | Glioma |
| 10568 | SLC34A2 | 2.97913375 | 9.65E-30 | Glioma |
| 167838 | TXLNB | 2.983221065 | 3.53E-48 | Glioma |
| 5551 | PRF1 | 2.996663857 | 3.00E-59 | Glioma |
| 28959 | TMEM176B | 2.998640173 | 8.22E-76 | Glioma |
| 4600 | MX2 | 3.008609087 | 1.03E-71 | Glioma |
| 94031 | HTRA3 | 3.008999171 | 1.99E-46 | Glioma |
| 221091 | LRRN4CL | 3.009459578 | 2.83E-45 | Glioma |
| 8651 | SOCS1 | 3.010454938 | 3.88E-79 | Glioma |
| 51200 | CPA4 | 3.011467962 | 7.27E-41 | Glioma |
| 6281 | S100A10 | 3.014050345 | 1.59E-64 | Glioma |
| 6037 | RNASE3 | 3.016366766 | 8.13E-58 | Glioma |
| 4818 | NKG7 | 3.02990854 | 6.16E-57 | Glioma |
| 85477 | SCIN | 3.035015375 | 1.06E-46 | Glioma |
| 8638 | OASL | 3.036790497 | 5.16E-57 | Glioma |
| 965 | CD58 | 3.038064279 | 3.07E-91 | Glioma |
| 6920 | TCEA3 | 3.041041557 | 5.45E-55 | Glioma |
| 10205 | MPZL2 | 3.0430553 | 5.90E-54 | Glioma |
| 54507 | ADAMTSL4 | 3.045779026 | 6.64E-90 | Glioma |
| 27180 | SIGLEC9 | 3.054721237 | 4.76E-80 | Glioma |
| 2212 | FCGR2A | 3.055339723 | 3.41E-90 | Glioma |
| 55843 | ARHGAP15 | 3.056602076 | 6.88E-73 | Glioma |
| 1154 | CISH | 3.060063625 | 1.22E-87 | Glioma |
| 50507 | NOX4 | 3.062674135 | 1.35E-42 | Glioma |
| 388512 | CLEC17A | 3.064439799 | 5.74E-41 | Glioma |
| 1521 | CTSW | 3.064634647 | 7.01E-61 | Glioma |
| 3855 | KRT7 | 3.073927267 | 8.21E-43 | Glioma |
| 164284 | APCDD1L | 3.074287729 | 3.91E-28 | Glioma |
| 3690 | ITGB3 | 3.075105072 | 1.32E-59 | Glioma |
| 3115 | HLA-DPB1 | 3.076075465 | 2.22E-75 | Glioma |
| 93190 | C1orf158 | 3.076092876 | 1.66E-24 | Glioma |
| 822 | CAPG | 3.076916967 | 3.34E-95 | Glioma |
| 1278 | COL1A2 | 3.078297485 | 3.51E-47 | Glioma |
| 8564 | KMO | 3.078756542 | 2.47E-53 | Glioma |
| 115572 | TENT5B | 3.080248334 | 3.71E-51 | Glioma |
| 84171 | LOXL4 | 3.083341879 | 4.31E-51 | Glioma |
| 90332 | EXOC3L2 | 3.091789444 | 7.03E-39 | Glioma |
| 9180 | OSMR | 3.095344433 | 3.20E-77 | Glioma |
| 7057 | THBS1 | 3.106550225 | 2.99E-42 | Glioma |
| 51279 | C1RL | 3.112291324 | 1.34E-99 | Glioma |
| 51311 | TLR8 | 3.11744952 | 8.03E-65 | Glioma |
| 3119 | HLA-DQB1 | 3.119168524 | 3.83E-50 | Glioma |
| 629 | CFB | 3.119290629 | 3.72E-66 | Glioma |
| 5996 | RGS1 | 3.120329811 | 5.52E-49 | Glioma |
| 1588 | CYP19A1 | 3.121351217 | 2.12E-54 | Glioma |
| 8091 | HMGA2 | 3.123291629 | 1.26E-27 | Glioma |
| 283592 | None | 3.130456958 | 7.00E-50 | Glioma |
| 121506 | ERP27 | 3.13537261 | 6.77E-56 | Glioma |
| 9333 | TGM5 | 3.138253019 | 3.10E-40 | Glioma |
| 716 | C1S | 3.138288293 | 4.85E-83 | Glioma |
| 154664 | ABCA13 | 3.139730085 | 1.33E-37 | Glioma |
| 972 | CD74 | 3.142760662 | 1.55E-88 | Glioma |
| 3123 | HLA-DRB1 | 3.148157793 | 1.22E-71 | Glioma |
| 1134 | CHRNA1 | 3.14964191 | 2.96E-27 | Glioma |
| 3221 | HOXC4 | 3.151778244 | 1.46E-35 | Glioma |
| 653361 | NCF1 | 3.152387142 | 6.52E-78 | Glioma |
| 113730 | KLHDC7B | 3.152917209 | 1.32E-76 | Glioma |
| 7262 | PHLDA2 | 3.15776845 | 1.79E-41 | Glioma |
| 118932 | ANKRD22 | 3.158585381 | 2.44E-45 | Glioma |
| 3958 | LGALS3 | 3.159088977 | 8.49E-64 | Glioma |
| 3512 | JCHAIN | 3.163524921 | 7.45E-28 | Glioma |
| 391059 | FRRS1 | 3.164475477 | 1.72E-62 | Glioma |
| 8701 | DNAH11 | 3.169454532 | 1.37E-36 | Glioma |
| 8407 | TAGLN2 | 3.171166716 | 3.70E-100 | Glioma |
| 100233209 | None | 3.172129077 | 3.52E-64 | Glioma |
| 57823 | SLAMF7 | 3.173950542 | 8.03E-51 | Glioma |
| 4261 | CIITA | 3.175282572 | 3.77E-76 | Glioma |
| 3111 | HLA-DOA | 3.185495959 | 1.16E-75 | Glioma |
| 6364 | CCL20 | 3.185989723 | 1.30E-37 | Glioma |
| 4237 | MFAP2 | 3.195449596 | 9.59E-33 | Glioma |
| 3199 | HOXA2 | 3.198670881 | 9.58E-30 | Glioma |
| 728 | C5AR1 | 3.200288227 | 1.12E-76 | Glioma |
| 27036 | SIGLEC7 | 3.200403487 | 1.26E-73 | Glioma |
| 55 | ACPP | 3.204004085 | 1.32E-55 | Glioma |
| 10158 | PDZK1IP1 | 3.204883217 | 5.24E-48 | Glioma |
| 84034 | EMILIN2 | 3.205739923 | 7.13E-68 | Glioma |
| 7850 | IL1R2 | 3.20692797 | 4.37E-37 | Glioma |
| 169044 | COL22A1 | 3.21140147 | 5.67E-27 | Glioma |
| 3113 | HLA-DPA1 | 3.211438088 | 8.42E-82 | Glioma |
| 10225 | CD96 | 3.211895258 | 1.39E-64 | Glioma |
| 654817 | None | 3.211911445 | 5.11E-70 | Glioma |
| 27240 | SIT1 | 3.214829005 | 5.10E-70 | Glioma |
| 162517 | FBXO39 | 3.215578636 | 2.39E-61 | Glioma |
| 2359 | FPR3 | 3.215874237 | 1.20E-45 | Glioma |
| 8685 | MARCO | 3.216846258 | 1.80E-27 | Glioma |
| 158314 | None | 3.217241607 | 4.99E-34 | Glioma |
| 316 | AOX1 | 3.21999665 | 9.52E-52 | Glioma |
| 2070 | EYA4 | 3.220510059 | 2.47E-27 | Glioma |
| 2318 | FLNC | 3.220763606 | 3.97E-49 | Glioma |
| 27299 | ADAMDEC1 | 3.222808568 | 1.79E-30 | Glioma |
| 4060 | LUM | 3.22507641 | 4.26E-43 | Glioma |
| 256355 | None | 3.226553618 | 9.95E-74 | Glioma |
| 624 | BDKRB2 | 3.226995549 | 7.72E-47 | Glioma |
| 283971 | CLEC18C | 3.237777692 | 1.67E-55 | Glioma |
| 11025 | LILRB3 | 3.238397071 | 4.44E-76 | Glioma |
| 143503 | OR51E1 | 3.239771015 | 2.51E-35 | Glioma |
| 218 | ALDH3A1 | 3.240315722 | 3.27E-45 | Glioma |
| 2357 | FPR1 | 3.241140425 | 4.37E-67 | Glioma |
| 114836 | SLAMF6 | 3.242334722 | 8.86E-69 | Glioma |
| 72 | ACTG2 | 3.248580082 | 5.29E-31 | Glioma |
| 9077 | DIRAS3 | 3.252481902 | 8.65E-62 | Glioma |
| 3932 | LCK | 3.254151167 | 3.93E-66 | Glioma |
| 2191 | FAP | 3.259312962 | 3.26E-48 | Glioma |
| 83871 | RAB34 | 3.263251106 | 1.59E-93 | Glioma |
| 4256 | MGP | 3.264711258 | 2.32E-51 | Glioma |
| 51513 | ETV7 | 3.266008724 | 9.62E-70 | Glioma |
| 3560 | IL2RB | 3.27265131 | 1.59E-66 | Glioma |
| 1282 | COL4A1 | 3.272875838 | 6.18E-51 | Glioma |
| 7045 | TGFBI | 3.272893249 | 1.37E-54 | Glioma |
| 122046 | TEX26 | 3.276858564 | 4.79E-44 | Glioma |
| 53831 | GPR84 | 3.281461893 | 2.21E-68 | Glioma |
| 366 | AQP9 | 3.281904464 | 2.11E-37 | Glioma |
| 10451 | VAV3 | 3.283265501 | 1.59E-56 | Glioma |
| 401551 | WDR38 | 3.285166978 | 7.78E-29 | Glioma |
| 3118 | HLA-DQA2 | 3.291113278 | 2.76E-34 | Glioma |
| 100130776 | None | 3.293598443 | 7.97E-59 | Glioma |
| 6398 | SECTM1 | 3.294826451 | 4.55E-71 | Glioma |
| 150677 | OTOS | 3.297752019 | 2.73E-34 | Glioma |
| 4129 | MAOB | 3.304645565 | 4.66E-38 | Glioma |
| 1800 | DPEP1 | 3.30469833 | 6.88E-28 | Glioma |
| 837 | CASP4 | 3.306239788 | 1.01E-112 | Glioma |
| 3426 | CFI | 3.313453494 | 1.94E-71 | Glioma |
| 132299 | OCIAD2 | 3.314510463 | 4.71E-51 | Glioma |
| 1192 | CLIC1 | 3.315839396 | 2.82E-115 | Glioma |
| 115273 | RAB42 | 3.31793866 | 2.33E-71 | Glioma |
| 79864 | JHY | 3.323215462 | 7.99E-47 | Glioma |
| 441168 | CALHM6 | 3.328406115 | 3.18E-84 | Glioma |
| 197021 | LCTL | 3.328872013 | 5.35E-59 | Glioma |
| 79961 | DENND2D | 3.330609212 | 7.44E-97 | Glioma |
| 9124 | PDLIM1 | 3.33204732 | 3.70E-81 | Glioma |
| 10457 | GPNMB | 3.333494743 | 2.65E-54 | Glioma |
| 353514 | LILRA5 | 3.336022896 | 2.02E-53 | Glioma |
| 6424 | SFRP4 | 3.343424564 | 7.71E-64 | Glioma |
| 56245 | C21orf62 | 3.346194922 | 8.45E-56 | Glioma |
| 644139 | PIRT | 3.346550185 | 5.38E-43 | Glioma |
| 100131187 | TSTD1 | 3.346733315 | 5.69E-42 | Glioma |
| 9047 | SH2D2A | 3.348383314 | 1.82E-74 | Glioma |
| 6352 | CCL5 | 3.355032274 | 1.00E-71 | Glioma |
| 2825 | GPR1 | 3.359648142 | 1.57E-40 | Glioma |
| 6338 | SCNN1B | 3.360668229 | 2.96E-28 | Glioma |
| 79168 | LILRA6 | 3.364592084 | 4.18E-69 | Glioma |
| 3120 | HLA-DQB2 | 3.36779366 | 1.02E-43 | Glioma |
| 3235 | HOXD9 | 3.372418032 | 8.71E-27 | Glioma |
| 26166 | RGS22 | 3.374245087 | 2.14E-34 | Glioma |
| 8530 | CST7 | 3.374821736 | 4.46E-79 | Glioma |
| 2633 | GBP1 | 3.386199801 | 7.53E-86 | Glioma |
| 1806 | DPYD | 3.391839983 | 7.25E-94 | Glioma |
| 1803 | DPP4 | 3.394325051 | 2.15E-47 | Glioma |
| 10644 | IGF2BP2 | 3.411742111 | 7.50E-40 | Glioma |
| 715 | C1R | 3.412751035 | 5.68E-91 | Glioma |
| 7421 | VDR | 3.413483959 | 1.21E-69 | Glioma |
| 54757 | FAM20A | 3.414384538 | 7.59E-65 | Glioma |
| 5008 | OSM | 3.420015657 | 7.71E-53 | Glioma |
| 56833 | SLAMF8 | 3.431669168 | 2.41E-84 | Glioma |
| 302 | ANXA2 | 3.442258005 | 8.61E-97 | Glioma |
| 497190 | CLEC18B | 3.444893138 | 2.33E-64 | Glioma |
| 79887 | PLBD1 | 3.447435688 | 6.35E-98 | Glioma |
| 135398 | C6orf141 | 3.453900959 | 2.29E-38 | Glioma |
| 346389 | MACC1 | 3.454066374 | 2.14E-67 | Glioma |
| 10643 | IGF2BP3 | 3.457375866 | 4.11E-45 | Glioma |
| 10538 | BATF | 3.458521281 | 2.58E-80 | Glioma |
| 114990 | VASN | 3.469183215 | 1.30E-76 | Glioma |
| 64231 | MS4A6A | 3.471262047 | 3.61E-66 | Glioma |
| 1234 | CCR5 | 3.471307748 | 7.51E-85 | Glioma |
| 6282 | S100A11 | 3.471795174 | 5.31E-111 | Glioma |
| 5329 | PLAUR | 3.472415322 | 1.19E-96 | Glioma |
| 202309 | GAPT | 3.479940312 | 1.11E-72 | Glioma |
| 92749 | DRC1 | 3.480647388 | 4.94E-29 | Glioma |
| 133 | ADM | 3.484201244 | 1.73E-53 | Glioma |
| 710 | SERPING1 | 3.485375107 | 2.49E-104 | Glioma |
| 1292 | COL6A2 | 3.488642694 | 1.38E-53 | Glioma |
| 5355 | PLP2 | 3.489216738 | 4.37E-88 | Glioma |
| 3600 | IL15 | 3.497104197 | 1.77E-87 | Glioma |
| 4046 | LSP1 | 3.511749731 | 4.40E-80 | Glioma |
| 56911 | MAP3K7CL | 3.515313654 | 4.00E-80 | Glioma |
| 8875 | VNN2 | 3.524959877 | 5.28E-77 | Glioma |
| 969 | CD69 | 3.525168706 | 7.57E-68 | Glioma |
| 8061 | FOSL1 | 3.52686958 | 1.85E-62 | Glioma |
| 114769 | CARD16 | 3.528460523 | 3.00E-96 | Glioma |
| 647946 | None | 3.531273718 | 1.94E-45 | Glioma |
| 241 | ALOX5AP | 3.536166435 | 5.29E-82 | Glioma |
| 4316 | MMP7 | 3.537260611 | 2.11E-29 | Glioma |
| 165 | AEBP1 | 3.539268588 | 1.76E-60 | Glioma |
| 10663 | CXCR6 | 3.540596122 | 4.14E-66 | Glioma |
| 2214 | FCGR3A | 3.55092226 | 4.99E-90 | Glioma |
| 2151 | F2RL2 | 3.55480764 | 3.91E-56 | Glioma |
| 4016 | LOXL1 | 3.555527995 | 8.34E-56 | Glioma |
| 256236 | None | 3.556874213 | 2.14E-49 | Glioma |
| 541471 | None | 3.558600903 | 5.80E-66 | Glioma |
| 3003 | GZMK | 3.559296153 | 2.77E-57 | Glioma |
| 11227 | GALNT5 | 3.563288158 | 7.25E-40 | Glioma |
| 3101 | HK3 | 3.574906551 | 1.20E-75 | Glioma |
| 3702 | ITK | 3.577393383 | 6.12E-63 | Glioma |
| 8942 | KYNU | 3.585688287 | 3.82E-83 | Glioma |
| 25790 | CFAP45 | 3.591154286 | 6.93E-45 | Glioma |
| 8755 | None | 3.60232337 | 4.99E-31 | Glioma |
| 3122 | HLA-DRA | 3.602377461 | 6.83E-89 | Glioma |
| 1289 | COL5A1 | 3.612287656 | 4.61E-50 | Glioma |
| 915 | CD3D | 3.627229081 | 8.79E-64 | Glioma |
| 84790 | TUBA1C | 3.632153776 | 4.87E-95 | Glioma |
| 4015 | LOX | 3.634136289 | 3.02E-55 | Glioma |
| 90050 | FAM181A | 3.651132354 | 1.08E-48 | Glioma |
| 3310 | HSPA6 | 3.655719252 | 6.06E-82 | Glioma |
| 303 | None | 3.661513608 | 4.15E-84 | Glioma |
| 51316 | PLAC8 | 3.666599627 | 7.49E-58 | Glioma |
| 3586 | IL10 | 3.667970443 | 2.18E-71 | Glioma |
| 1293 | COL6A3 | 3.678254274 | 1.70E-39 | Glioma |
| 3239 | HOXD13 | 3.686911509 | 1.02E-28 | Glioma |
| 400759 | None | 3.701705119 | 5.52E-70 | Glioma |
| 114614 | None | 3.70505406 | 1.37E-83 | Glioma |
| 5947 | RBP1 | 3.708027501 | 4.51E-66 | Glioma |
| 2171 | FABP5 | 3.710467831 | 1.51E-57 | Glioma |
| 5328 | PLAU | 3.722339886 | 5.81E-75 | Glioma |
| 6274 | S100A3 | 3.724881256 | 2.42E-53 | Glioma |
| 3223 | HOXC6 | 3.746312299 | 1.16E-45 | Glioma |
| 916 | CD3E | 3.747844334 | 6.21E-69 | Glioma |
| 10040 | TOM1L1 | 3.749008975 | 7.31E-50 | Glioma |
| 137835 | TMEM71 | 3.757088742 | 7.92E-84 | Glioma |
| 80380 | PDCD1LG2 | 3.7625822 | 2.23E-84 | Glioma |
| 2358 | FPR2 | 3.76361875 | 8.06E-59 | Glioma |
| 3559 | IL2RA | 3.768321161 | 9.90E-42 | Glioma |
| 5307 | PITX1 | 3.782805634 | 3.44E-41 | Glioma |
| 2162 | F13A1 | 3.78875874 | 3.87E-44 | Glioma |
| 4481 | MSR1 | 3.7894084 | 1.81E-93 | Glioma |
| 79730 | NSUN7 | 3.793207585 | 2.35E-78 | Glioma |
| 3557 | IL1RN | 3.798022434 | 7.09E-63 | Glioma |
| 55013 | MCUB | 3.802695434 | 1.42E-106 | Glioma |
| 160364 | CLEC12A | 3.802713337 | 1.79E-62 | Glioma |
| 8038 | ADAM12 | 3.808185203 | 3.51E-70 | Glioma |
| 729230 | CCR2 | 3.811688078 | 9.85E-70 | Glioma |
| 55240 | STEAP3 | 3.813061744 | 5.45E-92 | Glioma |
| 80832 | APOL4 | 3.816938103 | 1.79E-55 | Glioma |
| 6676 | SPAG4 | 3.82053235 | 1.73E-60 | Glioma |
| 4069 | LYZ | 3.821201577 | 1.19E-66 | Glioma |
| 6373 | CXCL11 | 3.830110689 | 1.02E-54 | Glioma |
| 8477 | GPR65 | 3.832751679 | 2.00E-89 | Glioma |
| 5265 | SERPINA1 | 3.83777823 | 1.44E-102 | Glioma |
| 136288 | C7orf57 | 3.841760277 | 8.21E-37 | Glioma |
| 4283 | CXCL9 | 3.844874778 | 1.21E-55 | Glioma |
| 27197 | GPR82 | 3.848545744 | 5.98E-64 | Glioma |
| 5778 | PTPN7 | 3.867624678 | 1.05E-81 | Glioma |
| 7130 | TNFAIP6 | 3.875056659 | 1.40E-58 | Glioma |
| 64005 | MYO1G | 3.875865486 | 1.67E-95 | Glioma |
| 10437 | IFI30 | 3.884510213 | 4.46E-107 | Glioma |
| 3117 | HLA-DQA1 | 3.887212362 | 7.35E-61 | Glioma |
| 6781 | STC1 | 3.892598029 | 3.07E-48 | Glioma |
| 1890 | TYMP | 3.894663838 | 2.83E-106 | Glioma |
| 6347 | CCL2 | 3.907743511 | 3.74E-68 | Glioma |
| 112597 | None | 3.908079759 | 3.27E-71 | Glioma |
| 132884 | EVC2 | 3.908763332 | 1.67E-71 | Glioma |
| 3237 | HOXD11 | 3.914389716 | 1.69E-39 | Glioma |
| 1674 | DES | 3.920484234 | 1.83E-37 | Glioma |
| 6556 | SLC11A1 | 3.922407282 | 5.02E-95 | Glioma |
| 768 | CA9 | 3.923701535 | 1.07E-30 | Glioma |
| 4982 | TNFRSF11B | 3.92567452 | 4.59E-76 | Glioma |
| 597 | BCL2A1 | 3.930408217 | 3.21E-76 | Glioma |
| 3001 | GZMA | 3.936033032 | 1.48E-70 | Glioma |
| 84419 | C15orf48 | 3.938413419 | 3.97E-62 | Glioma |
| 8857 | FCGBP | 3.943431402 | 1.15E-60 | Glioma |
| 6696 | SPP1 | 3.955342444 | 8.72E-74 | Glioma |
| 8840 | CCN4 | 3.957584236 | 7.13E-53 | Glioma |
| 1829 | DSG2 | 3.970665014 | 5.01E-39 | Glioma |
| 8572 | PDLIM4 | 4.00277835 | 3.96E-59 | Glioma |
| 9052 | GPRC5A | 4.003535709 | 2.07E-59 | Glioma |
| 55584 | CHRNA9 | 4.01026858 | 1.23E-41 | Glioma |
| 5322 | PLA2G5 | 4.012906599 | 2.16E-50 | Glioma |
| 5806 | PTX3 | 4.012980162 | 3.19E-60 | Glioma |
| 3217 | HOXB7 | 4.017629018 | 9.70E-57 | Glioma |
| 23529 | CLCF1 | 4.025415021 | 3.27E-84 | Glioma |
| 283120 | None | 4.026709832 | 1.04E-26 | Glioma |
| 6036 | RNASE2 | 4.036099529 | 1.74E-79 | Glioma |
| 3569 | IL6 | 4.039880169 | 7.95E-53 | Glioma |
| 59352 | LGR6 | 4.041787593 | 2.05E-37 | Glioma |
| 3236 | HOXD10 | 4.046311403 | 3.98E-38 | Glioma |
| 1281 | COL3A1 | 4.072924985 | 7.45E-44 | Glioma |
| 115362 | GBP5 | 4.118913964 | 1.05E-89 | Glioma |
| 26191 | PTPN22 | 4.1233911 | 2.35E-97 | Glioma |
| 914 | CD2 | 4.126564028 | 2.45E-80 | Glioma |
| 304 | None | 4.128665552 | 5.08E-101 | Glioma |
| 962 | CD48 | 4.142812453 | 3.16E-71 | Glioma |
| 9021 | SOCS3 | 4.143163952 | 2.82E-77 | Glioma |
| 440738 | MAP1LC3C | 4.144243997 | 7.27E-67 | Glioma |
| 5266 | PI3 | 4.171033136 | 1.04E-29 | Glioma |
| 1356 | CP | 4.181519937 | 3.50E-54 | Glioma |
| 1482 | NKX2-5 | 4.186273995 | 6.33E-42 | Glioma |
| 51330 | TNFRSF12A | 4.188893196 | 9.64E-89 | Glioma |
| 26499 | PLEK2 | 4.196569846 | 4.28E-79 | Glioma |
| 3311 | None | 4.219036802 | 1.48E-85 | Glioma |
| 3198 | HOXA1 | 4.226670789 | 1.12E-79 | Glioma |
| 6279 | S100A8 | 4.235151129 | 7.89E-58 | Glioma |
| 2015 | ADGRE1 | 4.27129991 | 1.89E-72 | Glioma |
| 6769 | STAC | 4.277200257 | 1.77E-49 | Glioma |
| 63898 | SH2D4A | 4.278483657 | 3.94E-94 | Glioma |
| 3485 | IGFBP2 | 4.278715411 | 1.18E-65 | Glioma |
| 3214 | HOXB4 | 4.28688361 | 6.27E-50 | Glioma |
| 6289 | SAA2 | 4.291715202 | 2.78E-35 | Glioma |
| 3200 | HOXA3 | 4.308278764 | 7.45E-46 | Glioma |
| 2331 | FMOD | 4.309339929 | 6.78E-62 | Glioma |
| 200132 | TCTEX1D1 | 4.312815218 | 1.77E-54 | Glioma |
| 493869 | GPX8 | 4.333566598 | 1.30E-80 | Glioma |
| 3242 | HPD | 4.3389328 | 1.24E-60 | Glioma |
| 6280 | S100A9 | 4.34265689 | 1.99E-65 | Glioma |
| 362 | AQP5 | 4.357414438 | 8.64E-48 | Glioma |
| 11082 | ESM1 | 4.372904189 | 1.51E-45 | Glioma |
| 5919 | RARRES2 | 4.386767923 | 8.81E-63 | Glioma |
| 9103 | FCGR2A | 4.387193478 | 2.73E-90 | Glioma |
| 3206 | HOXA10 | 4.396407196 | 2.62E-38 | Glioma |
| 3202 | HOXA5 | 4.396577338 | 4.04E-50 | Glioma |
| 26002 | MOXD1 | 4.396684401 | 4.82E-40 | Glioma |
| 57817 | HAMP | 4.413698475 | 4.99E-71 | Glioma |
| 115265 | DDIT4L | 4.423690131 | 9.26E-52 | Glioma |
| 196410 | METTL7B | 4.437842428 | 1.30E-74 | Glioma |
| 3976 | LIF | 4.443088207 | 6.47E-66 | Glioma |
| 301 | ANXA1 | 4.443089036 | 5.81E-103 | Glioma |
| 9518 | GDF15 | 4.44342558 | 9.53E-66 | Glioma |
| 23440 | OTP | 4.448934053 | 1.20E-48 | Glioma |
| 1277 | COL1A1 | 4.462117049 | 2.04E-53 | Glioma |
| 3212 | HOXB2 | 4.471504629 | 2.42E-57 | Glioma |
| 5054 | SERPINE1 | 4.485449147 | 3.45E-69 | Glioma |
| 1295 | COL8A1 | 4.487547733 | 1.28E-59 | Glioma |
| 3576 | CXCL8 | 4.538934386 | 2.48E-41 | Glioma |
| 63950 | DMRTA2 | 4.54405824 | 4.45E-46 | Glioma |
| 3598 | IL13RA2 | 4.562579633 | 1.70E-40 | Glioma |
| 6275 | S100A4 | 4.578010294 | 9.98E-96 | Glioma |
| 1475 | CSTA | 4.582729888 | 1.11E-90 | Glioma |
| 50486 | G0S2 | 4.5848869 | 2.37E-70 | Glioma |
| 4608 | MYBPH | 4.614603359 | 1.96E-67 | Glioma |
| 407977 | TNFSF12-TNFSF13 | 4.632677001 | 4.16E-74 | Glioma |
| 3201 | HOXA4 | 4.634657542 | 5.52E-43 | Glioma |
| 2019 | EN1 | 4.636561386 | 4.44E-48 | Glioma |
| 27286 | SRPX2 | 4.65304695 | 5.27E-72 | Glioma |
| 3226 | HOXC10 | 4.694069757 | 2.04E-42 | Glioma |
| 79054 | TRPM8 | 4.728359703 | 2.07E-63 | Glioma |
| 90853 | SPOCD1 | 4.751962214 | 3.58E-63 | Glioma |
| 9332 | CD163 | 4.758353896 | 5.26E-68 | Glioma |
| 761 | CA3 | 4.851354005 | 1.49E-59 | Glioma |
| 3204 | HOXA7 | 4.863205792 | 3.00E-39 | Glioma |
| 146802 | SLC47A2 | 4.889451658 | 1.54E-67 | Glioma |
| 7076 | TIMP1 | 4.981225456 | 2.35E-98 | Glioma |
| 6288 | SAA1 | 5.134356576 | 2.73E-42 | Glioma |
| 2014 | EMP3 | 5.220261201 | 1.13E-109 | Glioma |
| 2213 | FCGR2B | 5.239312872 | 3.15E-80 | Glioma |
| 12 | SERPINA3 | 5.283250399 | 2.14E-81 | Glioma |
| 6474 | SHOX2 | 5.300387978 | 1.29E-56 | Glioma |
| 5104 | SERPINA5 | 5.448809002 | 1.26E-84 | Glioma |
| 3381 | IBSP | 5.460527195 | 1.53E-49 | Glioma |
| 1117 | CHI3L2 | 5.485878697 | 1.35E-77 | Glioma |
| 54210 | TREM1 | 5.486258019 | 3.28E-82 | Glioma |
| 10630 | PDPN | 5.492687553 | 2.07E-99 | Glioma |
| 4318 | MMP9 | 5.545704196 | 1.34E-51 | Glioma |
| 4837 | NNMT | 5.575204648 | 2.60E-81 | Glioma |
| 3627 | CXCL10 | 5.604058763 | 2.55E-88 | Glioma |
| 3213 | HOXB3 | 5.616568865 | 4.26E-66 | Glioma |
| 4223 | MEOX2 | 5.62532406 | 8.55E-54 | Glioma |
| 23601 | CLEC5A | 5.637474625 | 2.10E-85 | Glioma |
| 5320 | PLA2G2A | 5.828855922 | 1.09E-46 | Glioma |
| 8714 | ABCC3 | 6.523033729 | 6.63E-99 | Glioma |
| 10631 | POSTN | 6.71400145 | 1.48E-53 | Glioma |
| 1116 | CHI3L1 | 7.71031451 | 3.99E-95 | Glioma |
| 4057 | LTF | 7.92124444 | 2.72E-71 | Glioma |
| 6440 | SFTPC | -4.124218873 | 1.00E-10 | Lung |
| 7356 | SCGB1A1 | -4.025364887 | 3.63E-13 | Lung |
| 931 | MS4A1 | -3.779360317 | 1.45E-32 | Lung |
| 260436 | FDCSP | -3.638762386 | 2.38E-24 | Lung |
| 6289 | SAA2 | -3.60867922 | 1.38E-22 | Lung |
| 2069 | EREG | -3.595292146 | 3.55E-15 | Lung |
| 6363 | CCL19 | -3.582822478 | 1.99E-35 | Lung |
| 26279 | PLA2G2D | -3.485802322 | 2.57E-31 | Lung |
| 9447 | AIM2 | -3.409966026 | 4.19E-29 | Lung |
| 6288 | SAA1 | -3.374234314 | 4.14E-20 | Lung |
| 10563 | CXCL13 | -3.304743526 | 2.76E-30 | Lung |
| 115352 | FCRL3 | -3.285941647 | 2.55E-40 | Lung |
| 4057 | LTF | -3.274332771 | 4.34E-18 | Lung |
| 8115 | None | -3.268096938 | 1.57E-29 | Lung |
| 163351 | GBP6 | -3.264430937 | 1.79E-26 | Lung |
| 930 | CD19 | -3.223634428 | 3.62E-27 | Lung |
| 640 | BLK | -3.168059182 | 8.54E-29 | Lung |
| 2208 | FCER2 | -3.16559853 | 1.03E-28 | Lung |
| 115350 | FCRL1 | -3.15291643 | 6.90E-33 | Lung |
| 4283 | CXCL9 | -3.14255907 | 1.49E-28 | Lung |
| 6373 | CXCL11 | -3.097014473 | 5.20E-31 | Lung |
| 653145 | ANXA8 | -3.086931871 | 9.86E-20 | Lung |
| 1118 | CHIT1 | -3.085801452 | 7.69E-20 | Lung |
| 27299 | ADAMDEC1 | -3.073610468 | 3.59E-22 | Lung |
| 10537 | UBD | -3.062743191 | 5.99E-29 | Lung |
| 53836 | GPR87 | -2.99521237 | 8.29E-13 | Lung |
| 244 | None | -2.971157205 | 3.15E-20 | Lung |
| 6689 | SPIB | -2.969008591 | 1.82E-33 | Lung |
| 3580 | None | -2.924042221 | 3.61E-27 | Lung |
| 84824 | FCRLA | -2.921090921 | 7.45E-28 | Lung |
| 3957 | LGALS2 | -2.895355241 | 2.63E-30 | Lung |
| 1269 | CNR2 | -2.879701112 | 3.83E-36 | Lung |
| 3003 | GZMK | -2.871141035 | 2.48E-40 | Lung |
| 401124 | DTHD1 | -2.855892509 | 5.48E-27 | Lung |
| 3713 | IVL | -2.849733257 | 8.57E-10 | Lung |
| 3627 | CXCL10 | -2.831582926 | 3.52E-30 | Lung |
| 4316 | MMP7 | -2.828079604 | 2.01E-16 | Lung |
| 10462 | CLEC10A | -2.823037489 | 1.46E-43 | Lung |
| 26228 | STAP1 | -2.814291193 | 3.87E-35 | Lung |
| 266977 | ADGRF1 | -2.798209616 | 2.30E-11 | Lung |
| 144809 | FAM216B | -2.789034275 | 2.69E-14 | Lung |
| 3575 | IL7R | -2.784341909 | 2.56E-40 | Lung |
| 199786 | FAM129C | -2.783955816 | 5.65E-25 | Lung |
| 3852 | KRT5 | -2.783307371 | 2.04E-14 | Lung |
| 3458 | IFNG | -2.782071804 | 4.56E-26 | Lung |
| 80326 | WNT10A | -2.778883534 | 2.40E-25 | Lung |
| 50852 | TRAT1 | -2.777399064 | 1.74E-44 | Lung |
| 3604 | TNFRSF9 | -2.773953581 | 3.50E-36 | Lung |
| 140947 | DCANP1 | -2.762972528 | 1.33E-36 | Lung |
| 643 | CXCR5 | -2.756839192 | 1.58E-34 | Lung |
| 2015 | ADGRE1 | -2.708900855 | 1.64E-25 | Lung |
| 259197 | NCR3 | -2.706605928 | 6.97E-41 | Lung |
| 149628 | PYHIN1 | -2.703701393 | 9.43E-44 | Lung |
| 6372 | CXCL6 | -2.699232049 | 9.68E-14 | Lung |
| 441168 | CALHM6 | -2.696564159 | 2.60E-37 | Lung |
| 81793 | TLR10 | -2.695538564 | 2.26E-32 | Lung |
| 23495 | TNFRSF13B | -2.6951614 | 1.02E-23 | Lung |
| 1510 | CTSE | -2.692034045 | 2.40E-09 | Lung |
| 29851 | ICOS | -2.689995371 | 1.10E-42 | Lung |
| 3897 | L1CAM | -2.689690828 | 3.68E-20 | Lung |
| 4068 | SH2D1A | -2.687164508 | 4.51E-46 | Lung |
| 4314 | MMP3 | -2.684448921 | 1.29E-15 | Lung |
| 497189 | TIFAB | -2.678498648 | 1.42E-38 | Lung |
| 23547 | LILRA4 | -2.674237275 | 3.27E-32 | Lung |
| 4606 | MYBPC2 | -2.66551236 | 5.57E-22 | Lung |
| 3117 | HLA-DQA1 | -2.658614171 | 1.94E-34 | Lung |
| 64805 | P2RY12 | -2.647621834 | 1.46E-29 | Lung |
| 125 | ADH1B | -2.639495818 | 3.52E-12 | Lung |
| 79368 | FCRL2 | -2.633358066 | 7.93E-21 | Lung |
| 84636 | GPR174 | -2.627787089 | 8.56E-41 | Lung |
| 313 | AOAH | -2.627108506 | 1.49E-41 | Lung |
| 388512 | CLEC17A | -2.624505939 | 3.22E-28 | Lung |
| 3702 | ITK | -2.623364983 | 2.36E-50 | Lung |
| 115362 | GBP5 | -2.604734756 | 5.70E-35 | Lung |
| 6291 | SAA4 | -2.595275282 | 3.51E-19 | Lung |
| 79931 | TNIP3 | -2.581079124 | 1.31E-22 | Lung |
| 116379 | IL22RA2 | -2.575882806 | 2.83E-19 | Lung |
| 55423 | SIRPG | -2.5742775 | 3.72E-40 | Lung |
| 151888 | BTLA | -2.573680091 | 3.22E-40 | Lung |
| 3620 | IDO1 | -2.573295753 | 2.14E-25 | Lung |
| 56253 | CRTAM | -2.557201225 | 1.60E-46 | Lung |
| 1236 | CCR7 | -2.55587299 | 2.73E-42 | Lung |
| 910 | CD1B | -2.549842066 | 2.32E-19 | Lung |
| 9834 | None | -2.549734111 | 6.36E-21 | Lung |
| 128611 | ZNF831 | -2.54680162 | 7.62E-36 | Lung |
| 339145 | FAM92B | -2.542522085 | 6.84E-16 | Lung |
| 970 | None | -2.541417411 | 2.98E-31 | Lung |
| 933 | CD22 | -2.539711985 | 6.50E-29 | Lung |
| 128602 | C20orf85 | -2.536317914 | 3.41E-09 | Lung |
| 80380 | PDCD1LG2 | -2.534516041 | 1.05E-42 | Lung |
| 387357 | THEMIS | -2.533945399 | 2.74E-37 | Lung |
| 6699 | SPRR1B | -2.533877667 | 3.27E-10 | Lung |
| 257101 | ZNF683 | -2.533083034 | 1.65E-24 | Lung |
| 4753 | NELL2 | -2.531524019 | 2.54E-26 | Lung |
| 654817 | None | -2.531006862 | 9.58E-34 | Lung |
| 9576 | SPAG6 | -2.526389226 | 3.13E-10 | Lung |
| 26253 | CLEC4E | -2.526365161 | 1.96E-28 | Lung |
| 27240 | SIT1 | -2.524017322 | 3.67E-43 | Lung |
| 94025 | MUC16 | -2.523246229 | 3.10E-07 | Lung |
| 8685 | MARCO | -2.517060953 | 1.73E-17 | Lung |
| 7535 | ZAP70 | -2.516779686 | 7.33E-39 | Lung |
| 51311 | TLR8 | -2.515835929 | 2.14E-35 | Lung |
| 9840 | TESPA1 | -2.515458522 | 7.97E-42 | Lung |
| 1770 | DNAH9 | -2.510313978 | 6.72E-10 | Lung |
| 57863 | CADM3 | -2.510186817 | 1.53E-24 | Lung |
| 7476 | WNT7A | -2.508321275 | 5.03E-13 | Lung |
| 388325 | SCIMP | -2.504571996 | 6.57E-40 | Lung |
| 2568 | GABRP | -2.502044358 | 1.78E-09 | Lung |
| 91828 | EXOC3L4 | -2.496967067 | 1.71E-24 | Lung |
| 160365 | CLECL1 | -2.494146117 | 7.12E-38 | Lung |
| 83416 | FCRL5 | -2.493544456 | 3.60E-20 | Lung |
| 22914 | KLRK1 | -2.492779412 | 3.07E-39 | Lung |
| 201633 | TIGIT | -2.491893355 | 1.28E-41 | Lung |
| 5079 | PAX5 | -2.490293482 | 3.56E-20 | Lung |
| 4033 | LRMP | -2.49025201 | 1.85E-38 | Lung |
| 3586 | IL10 | -2.486923598 | 3.35E-39 | Lung |
| 973 | CD79A | -2.486787225 | 7.22E-22 | Lung |
| 154075 | SAMD3 | -2.475845274 | 6.92E-47 | Lung |
| 6362 | CCL18 | -2.470172097 | 1.95E-24 | Lung |
| 127254 | ERICH3 | -2.467445763 | 4.50E-11 | Lung |
| 400759 | None | -2.460284587 | 2.16E-34 | Lung |
| 6368 | CCL23 | -2.458097427 | 6.43E-25 | Lung |
| 1755 | DMBT1 | -2.45743742 | 1.77E-07 | Lung |
| 8900 | CCNA1 | -2.452036078 | 1.33E-14 | Lung |
| 969 | CD69 | -2.449002261 | 6.40E-38 | Lung |
| 256380 | SCML4 | -2.447454413 | 2.69E-39 | Lung |
| 5133 | PDCD1 | -2.444912326 | 1.70E-36 | Lung |
| 6003 | RGS13 | -2.442253478 | 1.25E-25 | Lung |
| 338339 | CLEC4D | -2.440777423 | 2.19E-30 | Lung |
| 8807 | IL18RAP | -2.438427248 | 7.20E-38 | Lung |
| 3594 | IL12RB1 | -2.438172151 | 2.08E-48 | Lung |
| 4049 | LTA | -2.43758366 | 7.80E-42 | Lung |
| 11148 | HHLA2 | -2.433916855 | 1.75E-06 | Lung |
| 9744 | ACAP1 | -2.433668134 | 2.18E-43 | Lung |
| 256076 | COL6A5 | -2.432200458 | 1.47E-20 | Lung |
| 917 | CD3G | -2.431123842 | 4.68E-32 | Lung |
| 3559 | IL2RA | -2.430878302 | 8.55E-35 | Lung |
| 974 | CD79B | -2.430162981 | 4.67E-32 | Lung |
| 962 | CD48 | -2.427942891 | 2.63E-46 | Lung |
| 2532 | ACKR1 | -2.42602166 | 2.81E-16 | Lung |
| 1233 | CCR4 | -2.425598067 | 1.75E-30 | Lung |
| 6504 | SLAMF1 | -2.424876527 | 4.98E-42 | Lung |
| 11006 | LILRB4 | -2.416466884 | 6.07E-35 | Lung |
| 22797 | TFEC | -2.41635758 | 8.81E-46 | Lung |
| 6361 | CCL17 | -2.416109476 | 7.49E-18 | Lung |
| 3111 | HLA-DOA | -2.414566293 | 3.98E-37 | Lung |
| 6402 | SELL | -2.412645585 | 6.34E-40 | Lung |
| 7634 | ZNF80 | -2.412256887 | 1.80E-29 | Lung |
| 114836 | SLAMF6 | -2.404519639 | 3.12E-41 | Lung |
| 8832 | CD84 | -2.396008643 | 1.04E-36 | Lung |
| 3821 | KLRC1 | -2.395283949 | 4.26E-23 | Lung |
| 30009 | TBX21 | -2.395004492 | 4.02E-31 | Lung |
| 3753 | KCNE1 | -2.394791389 | 5.46E-20 | Lung |
| 79645 | EFCAB1 | -2.39300224 | 1.13E-11 | Lung |
| 92304 | SCGB3A1 | -2.390987391 | 8.15E-07 | Lung |
| 5788 | PTPRC | -2.388059871 | 1.04E-49 | Lung |
| 399949 | C11orf88 | -2.380706572 | 1.52E-11 | Lung |
| 6356 | CCL11 | -2.379529743 | 3.78E-19 | Lung |
| 89790 | SIGLEC10 | -2.37747768 | 1.75E-38 | Lung |
| 3824 | KLRD1 | -2.375884794 | 2.13E-32 | Lung |
| 25890 | ABI3BP | -2.375816688 | 3.39E-26 | Lung |
| 1493 | CTLA4 | -2.37460431 | 6.86E-34 | Lung |
| 1359 | CPA3 | -2.374467754 | 1.11E-17 | Lung |
| 53347 | UBASH3A | -2.37348316 | 1.81E-43 | Lung |
| 5730 | PTGDS | -2.373309461 | 5.16E-29 | Lung |
| 5320 | PLA2G2A | -2.371108031 | 2.82E-12 | Lung |
| 1776 | DNASE1L3 | -2.369845061 | 2.05E-16 | Lung |
| 50615 | IL21R | -2.367919919 | 5.02E-44 | Lung |
| 27334 | P2RY10 | -2.365385208 | 1.06E-35 | Lung |
| 4818 | NKG7 | -2.363780733 | 5.37E-35 | Lung |
| 654816 | None | -2.36325329 | 4.84E-34 | Lung |
| 925 | CD8A | -2.362526107 | 3.06E-34 | Lung |
| 1378 | CR1 | -2.362252032 | 5.07E-31 | Lung |
| 374403 | TBC1D10C | -2.362139062 | 3.76E-47 | Lung |
| 100233209 | None | -2.358666919 | 3.05E-44 | Lung |
| 79413 | ZBED2 | -2.358641304 | 6.02E-27 | Lung |
| 3552 | IL1A | -2.350532901 | 1.75E-16 | Lung |
| 959 | CD40LG | -2.349012465 | 6.59E-30 | Lung |
| 81030 | ZBP1 | -2.346729205 | 1.78E-31 | Lung |
| 225 | ABCD2 | -2.345538409 | 3.19E-40 | Lung |
| 1536 | CYBB | -2.345331784 | 3.05E-39 | Lung |
| 2322 | FLT3 | -2.343501443 | 2.84E-30 | Lung |
| 3001 | GZMA | -2.341789606 | 8.02E-35 | Lung |
| 941 | CD80 | -2.341497961 | 1.56E-39 | Lung |
| 915 | CD3D | -2.340548806 | 1.48E-37 | Lung |
| 53829 | P2RY13 | -2.338217384 | 8.51E-40 | Lung |
| 284749 | None | -2.334624173 | 2.80E-26 | Lung |
| 8320 | EOMES | -2.333186985 | 5.28E-29 | Lung |
| 2999 | GZMH | -2.329913294 | 6.37E-30 | Lung |
| 5790 | PTPRCAP | -2.329736985 | 2.68E-45 | Lung |
| 114614 | None | -2.327509632 | 5.72E-45 | Lung |
| 2358 | FPR2 | -2.326061447 | 1.17E-23 | Lung |
| 83539 | CHST9 | -2.321953223 | 1.70E-08 | Lung |
| 7503 | None | -2.320315917 | 0.001240971 | Lung |
| 10235 | RASGRP2 | -2.318592569 | 1.43E-37 | Lung |
| 3002 | GZMB | -2.317703631 | 5.00E-24 | Lung |
| 356 | FASLG | -2.31766116 | 1.05E-28 | Lung |
| 2357 | FPR1 | -2.315825148 | 3.42E-36 | Lung |
| 6352 | CCL5 | -2.315524295 | 3.36E-39 | Lung |
| 401563 | C9orf139 | -2.315440774 | 2.17E-31 | Lung |
| 57823 | SLAMF7 | -2.314977836 | 3.36E-30 | Lung |
| 221476 | PI16 | -2.314477553 | 1.26E-14 | Lung |
| 389118 | CDHR4 | -2.311647563 | 3.96E-10 | Lung |
| 653361 | NCF1 | -2.308947217 | 1.15E-36 | Lung |
| 944 | TNFSF8 | -2.307952816 | 3.94E-38 | Lung |
| 6098 | ROS1 | -2.307701414 | 3.41E-11 | Lung |
| 713 | C1QB | -2.304841525 | 3.38E-35 | Lung |
| 3820 | KLRB1 | -2.30348122 | 3.94E-33 | Lung |
| 730 | C7 | -2.302571024 | 8.47E-15 | Lung |
| 939 | CD27 | -2.299073869 | 4.77E-32 | Lung |
| 5341 | PLEK | -2.298947394 | 6.25E-48 | Lung |
| 914 | CD2 | -2.297507531 | 2.05E-40 | Lung |
| 919 | CD247 | -2.295848434 | 6.03E-50 | Lung |
| 3394 | IRF8 | -2.291510206 | 3.90E-48 | Lung |
| 3112 | HLA-DOB | -2.289903756 | 4.08E-30 | Lung |
| 9332 | CD163 | -2.289355885 | 2.54E-32 | Lung |
| 54900 | LAX1 | -2.289122671 | 6.36E-26 | Lung |
| 2533 | FYB1 | -2.286652584 | 2.73E-47 | Lung |
| 916 | CD3E | -2.283384607 | 1.62E-46 | Lung |
| 2529 | FUT7 | -2.27964634 | 1.19E-34 | Lung |
| 3101 | HK3 | -2.278974272 | 9.94E-31 | Lung |
| 203100 | HTRA4 | -2.277834104 | 1.74E-25 | Lung |
| 387751 | None | -2.277434504 | 1.91E-42 | Lung |
| 29126 | CD274 | -2.276807355 | 8.41E-25 | Lung |
| 6357 | CCL13 | -2.272129587 | 4.68E-20 | Lung |
| 11326 | VSIG4 | -2.271390936 | 1.10E-29 | Lung |
| 2841 | GPR18 | -2.268880266 | 5.09E-39 | Lung |
| 608 | TNFRSF17 | -2.268432168 | 4.80E-17 | Lung |
| 10170 | DHRS9 | -2.267027939 | 3.62E-19 | Lung |
| 2209 | FCGR1A | -2.265095943 | 1.65E-34 | Lung |
| 695 | BTK | -2.263165518 | 3.43E-42 | Lung |
| 3122 | HLA-DRA | -2.262105485 | 2.12E-37 | Lung |
| 9051 | PSTPIP1 | -2.260607931 | 9.79E-50 | Lung |
| 3067 | HDC | -2.25927607 | 4.58E-22 | Lung |
| 5368 | PNOC | -2.258536705 | 3.56E-19 | Lung |
| 2162 | F13A1 | -2.255635045 | 9.00E-27 | Lung |
| 124460 | SNX20 | -2.254866195 | 1.66E-45 | Lung |
| 5724 | PTAFR | -2.250213866 | 7.65E-32 | Lung |
| 10859 | LILRB1 | -2.249154056 | 3.39E-41 | Lung |
| 4050 | LTB | -2.248322312 | 1.41E-33 | Lung |
| 80342 | TRAF3IP3 | -2.248161052 | 2.62E-52 | Lung |
| 197135 | PATL2 | -2.247276928 | 1.18E-28 | Lung |
| 343413 | FCRL6 | -2.246518407 | 2.83E-30 | Lung |
| 712 | C1QA | -2.246239519 | 1.93E-37 | Lung |
| 64499 | TPSB2 | -2.24581905 | 2.14E-19 | Lung |
| 5027 | P2RX7 | -2.245493674 | 2.16E-31 | Lung |
| 344 | APOC2 | -2.245482092 | 1.95E-21 | Lung |
| 83417 | FCRL4 | -2.241644273 | 1.01E-21 | Lung |
| 3071 | NCKAP1L | -2.24082865 | 1.47E-42 | Lung |
| 53831 | GPR84 | -2.238971878 | 3.42E-29 | Lung |
| 6846 | XCL2 | -2.238418627 | 3.91E-28 | Lung |
| 326342 | None | -2.23765806 | 9.82E-23 | Lung |
| 4318 | MMP9 | -2.235534738 | 4.63E-21 | Lung |
| 3123 | HLA-DRB1 | -2.234901181 | 3.52E-29 | Lung |
| 645784 | None | -2.234327617 | 3.02E-15 | Lung |
| 9547 | CXCL14 | -2.233180161 | 5.87E-09 | Lung |
| 4360 | MRC1 | -2.231325269 | 7.99E-26 | Lung |
| 6614 | SIGLEC1 | -2.230976998 | 1.23E-30 | Lung |
| 8875 | VNN2 | -2.229691124 | 6.56E-28 | Lung |
| 29802 | VPREB3 | -2.229199003 | 6.03E-23 | Lung |
| 85479 | DNAJC5B | -2.227457838 | 5.61E-27 | Lung |
| 131450 | CD200R1 | -2.22710698 | 5.45E-41 | Lung |
| 8091 | HMGA2 | -2.227096333 | 3.90E-06 | Lung |
| 26191 | PTPN22 | -2.22702224 | 5.15E-38 | Lung |
| 11095 | ADAMTS8 | -2.226020436 | 2.30E-14 | Lung |
| 89857 | KLHL6 | -2.225415682 | 9.79E-43 | Lung |
| 221188 | ADGRG5 | -2.22396184 | 3.27E-31 | Lung |
| 3116 | None | -2.223493228 | 7.09E-24 | Lung |
| 4499 | MT1M | -2.2233772 | 2.93E-19 | Lung |
| 4332 | MNDA | -2.221122321 | 2.27E-35 | Lung |
| 913 | CD1E | -2.22026725 | 1.51E-14 | Lung |
| 10666 | CD226 | -2.219374417 | 1.29E-39 | Lung |
| 6351 | CCL4 | -2.214770094 | 4.59E-39 | Lung |
| 131873 | COL6A6 | -2.2138467 | 1.90E-15 | Lung |
| 10990 | LILRB5 | -2.212312383 | 6.38E-26 | Lung |
| 814 | CAMK4 | -2.21184627 | 2.25E-34 | Lung |
| 100049587 | SIGLEC14 | -2.211302733 | 4.54E-21 | Lung |
| 714 | C1QC | -2.210066583 | 6.65E-36 | Lung |
| 27181 | SIGLEC8 | -2.209955703 | 3.23E-20 | Lung |
| 51676 | ASB2 | -2.209513969 | 6.82E-38 | Lung |
| 1794 | DOCK2 | -2.208703874 | 2.13E-45 | Lung |
| 2633 | GBP1 | -2.208310388 | 1.38E-37 | Lung |
| 4069 | LYZ | -2.208074071 | 9.67E-23 | Lung |
| 951 | CD37 | -2.207812246 | 2.57E-47 | Lung |
| 64231 | MS4A6A | -2.207545788 | 5.04E-44 | Lung |
| 170371 | TMEM273 | -2.206634827 | 1.50E-34 | Lung |
| 921 | CD5 | -2.205709748 | 1.19E-31 | Lung |
| 3853 | KRT6A | -2.204814289 | 5.11E-06 | Lung |
| 3115 | HLA-DPB1 | -2.204139113 | 1.73E-35 | Lung |
| 51208 | CLDN18 | -2.203813539 | 2.47E-05 | Lung |
| 10148 | EBI3 | -2.203746572 | 9.22E-37 | Lung |
| 10288 | LILRB2 | -2.202169494 | 4.06E-39 | Lung |
| 6236 | RRAD | -2.202139383 | 1.53E-19 | Lung |
| 80183 | RUBCNL | -2.202012308 | 3.65E-41 | Lung |
| 114548 | NLRP3 | -2.201924436 | 6.16E-43 | Lung |
| 4321 | MMP12 | -2.200904812 | 7.80E-12 | Lung |
| 64333 | ARHGAP9 | -2.20074388 | 2.07E-51 | Lung |
| 3568 | IL5RA | -2.198174486 | 7.23E-18 | Lung |
| 3290 | HSD11B1 | -2.197638775 | 7.82E-35 | Lung |
| 56833 | SLAMF8 | -2.197529694 | 4.03E-41 | Lung |
| 91937 | TIMD4 | -2.197289526 | 3.95E-21 | Lung |
| 389643 | NUGGC | -2.196948731 | 1.85E-22 | Lung |
| 729230 | CCR2 | -2.196689031 | 8.39E-34 | Lung |
| 117289 | TAGAP | -2.19593621 | 1.24E-46 | Lung |
| 2051 | EPHB6 | -2.195570346 | 3.99E-23 | Lung |
| 10077 | TSPAN32 | -2.19551444 | 8.72E-30 | Lung |
| 3118 | HLA-DQA2 | -2.195376818 | 4.44E-21 | Lung |
| 51237 | MZB1 | -2.195188743 | 4.36E-16 | Lung |
| 10225 | CD96 | -2.194516411 | 5.88E-31 | Lung |
| 23460 | ABCA6 | -2.194085338 | 2.27E-29 | Lung |
| 3127 | HLA-DRB5 | -2.193909637 | 7.29E-20 | Lung |
| 23650 | TRIM29 | -2.187970711 | 1.24E-07 | Lung |
| 7177 | TPSAB1 | -2.187946003 | 1.06E-19 | Lung |
| 283897 | C16orf54 | -2.187517402 | 7.53E-39 | Lung |
| 10663 | CXCR6 | -2.187219143 | 2.26E-40 | Lung |
| 6507 | SLC1A3 | -2.183271909 | 2.64E-32 | Lung |
| 54440 | SASH3 | -2.18321904 | 1.07E-52 | Lung |
| 3120 | HLA-DQB2 | -2.182622896 | 2.88E-14 | Lung |
| 10871 | CD300C | -2.182415765 | 3.04E-33 | Lung |
| 1234 | CCR5 | -2.179214233 | 8.41E-49 | Lung |
| 284417 | TMEM150B | -2.178429859 | 7.90E-24 | Lung |
| 50856 | CLEC4A | -2.178292866 | 1.70E-41 | Lung |
| 147744 | TMEM190 | -2.176936459 | 1.14E-08 | Lung |
| 11184 | MAP4K1 | -2.174559547 | 2.10E-38 | Lung |
| 160364 | CLEC12A | -2.174244816 | 2.97E-23 | Lung |
| 3932 | LCK | -2.169699486 | 5.02E-45 | Lung |
| 2213 | FCGR2B | -2.169687183 | 2.58E-33 | Lung |
| 126259 | TMIGD2 | -2.169286041 | 1.40E-31 | Lung |
| 122618 | PLD4 | -2.169175213 | 1.01E-20 | Lung |
| 6285 | S100B | -2.168824254 | 7.02E-19 | Lung |
| 6252 | RTN1 | -2.168801879 | 4.57E-27 | Lung |
| 29125 | C11orf21 | -2.166725663 | 1.14E-30 | Lung |
| 2707 | GJB3 | -2.165950495 | 8.63E-10 | Lung |
| 8477 | GPR65 | -2.165937031 | 1.85E-48 | Lung |
| 64092 | SAMSN1 | -2.164284849 | 9.54E-42 | Lung |
| 1439 | CSF2RB | -2.163903439 | 3.08E-44 | Lung |
| 3055 | HCK | -2.163830287 | 4.13E-39 | Lung |
| 945 | CD33 | -2.16279501 | 1.51E-37 | Lung |
| 4261 | CIITA | -2.160997763 | 2.74E-33 | Lung |
| 8483 | CILP | -2.159932195 | 7.96E-14 | Lung |
| 3113 | HLA-DPA1 | -2.157240624 | 1.46E-33 | Lung |
| 3662 | IRF4 | -2.156277122 | 1.58E-24 | Lung |
| 81832 | NETO1 | -2.15544512 | 6.40E-13 | Lung |
| 10673 | TNFSF13B | -2.152736132 | 8.14E-38 | Lung |
| 55843 | ARHGAP15 | -2.151396669 | 1.82E-45 | Lung |
| 963 | CD53 | -2.150116955 | 7.25E-50 | Lung |
| 2196 | FAT2 | -2.149875508 | 3.18E-11 | Lung |
| 84868 | HAVCR2 | -2.149397994 | 4.18E-43 | Lung |
| 1474 | CST6 | -2.14914818 | 5.22E-12 | Lung |
| 5294 | PIK3CG | -2.146749063 | 7.66E-37 | Lung |
| 164668 | APOBEC3H | -2.146694805 | 9.62E-24 | Lung |
| 7136 | TNNI2 | -2.146371429 | 7.76E-20 | Lung |
| 7124 | TNF | -2.145519773 | 6.57E-23 | Lung |
| 597 | BCL2A1 | -2.145160804 | 1.72E-38 | Lung |
| 27063 | ANKRD1 | -2.144832847 | 6.89E-10 | Lung |
| 6999 | TDO2 | -2.143529116 | 1.12E-19 | Lung |
| 80008 | TMEM156 | -2.142787886 | 3.25E-19 | Lung |
| 255231 | MCOLN2 | -2.142760232 | 1.32E-31 | Lung |
| 83659 | TEKT1 | -2.141742859 | 2.83E-08 | Lung |
| 1043 | CD52 | -2.141669153 | 3.31E-38 | Lung |
| 80129 | CCDC170 | -2.140710842 | 2.63E-21 | Lung |
| 100132417 | None | -2.139751009 | 1.30E-30 | Lung |
| 2350 | FOLR2 | -2.139351053 | 2.64E-29 | Lung |
| 64078 | SLC28A3 | -2.138033279 | 1.60E-13 | Lung |
| 5923 | RASGRF1 | -2.136642987 | 2.67E-11 | Lung |
| 51284 | TLR7 | -2.136451288 | 3.92E-33 | Lung |
| 9982 | FGFBP1 | -2.135177307 | 4.22E-09 | Lung |
| 120425 | JAML | -2.134441781 | 1.21E-38 | Lung |
| 5778 | PTPN7 | -2.133380117 | 2.25E-47 | Lung |
| 64407 | RGS18 | -2.13232569 | 3.95E-35 | Lung |
| 1089 | CEACAM4 | -2.131015416 | 1.00E-19 | Lung |
| 374 | AREG | -2.128997599 | 7.06E-12 | Lung |
| 3902 | LAG3 | -2.128701168 | 6.16E-29 | Lung |
| 3681 | ITGAD | -2.128393765 | 1.47E-19 | Lung |
| 115361 | GBP4 | -2.127413531 | 1.39E-37 | Lung |
| 10578 | GNLY | -2.126357342 | 1.65E-20 | Lung |
| 164118 | TTC24 | -2.125640808 | 8.50E-27 | Lung |
| 342615 | None | -2.125115814 | 2.20E-34 | Lung |
| 266747 | RGL4 | -2.125012115 | 1.76E-39 | Lung |
| 10320 | IKZF1 | -2.124987557 | 3.61E-47 | Lung |
| 3683 | ITGAL | -2.124660316 | 2.26E-43 | Lung |
| 10261 | IGSF6 | -2.124056115 | 6.71E-38 | Lung |
| 5453 | POU3F1 | -2.124051548 | 2.71E-25 | Lung |
| 6279 | S100A8 | -2.123806887 | 7.46E-17 | Lung |
| 8876 | VNN1 | -2.120890144 | 7.61E-16 | Lung |
| 3109 | HLA-DMB | -2.118168548 | 9.31E-38 | Lung |
| 5450 | POU2AF1 | -2.117794056 | 2.76E-16 | Lung |
| 147920 | IGFL2 | -2.116377818 | 2.54E-11 | Lung |
| 6366 | CCL21 | -2.115169801 | 2.08E-18 | Lung |
| 115701 | ALPK2 | -2.114046364 | 4.29E-19 | Lung |
| 1674 | DES | -2.114008842 | 1.86E-13 | Lung |
| 3903 | LAIR1 | -2.112941 | 9.29E-40 | Lung |
| 5800 | PTPRO | -2.111584063 | 7.45E-31 | Lung |
| 838 | CASP5 | -2.110354057 | 2.63E-24 | Lung |
| 4222 | MEOX1 | -2.109514458 | 4.31E-19 | Lung |
| 4914 | NTRK1 | -2.107197405 | 1.98E-27 | Lung |
| 27033 | ZBTB32 | -2.105740769 | 1.04E-33 | Lung |
| 926 | CD8B | -2.104977428 | 9.35E-24 | Lung |
| 940 | CD28 | -2.104088387 | 1.03E-41 | Lung |
| 9103 | FCGR2A | -2.103797514 | 2.11E-35 | Lung |
| 971 | CD72 | -2.103492823 | 3.78E-43 | Lung |
| 911 | CD1C | -2.100422513 | 4.02E-16 | Lung |
| 10875 | FGL2 | -2.099947433 | 2.91E-31 | Lung |
| 50943 | FOXP3 | -2.095605224 | 2.44E-36 | Lung |
| 4063 | LY9 | -2.09441185 | 7.56E-30 | Lung |
| 2123 | EVI2A | -2.094273123 | 1.86E-42 | Lung |
| 2210 | FCGR1B | -2.094028758 | 7.46E-33 | Lung |
| 4322 | MMP13 | -2.090665033 | 3.79E-07 | Lung |
| 6355 | CCL8 | -2.090567179 | 8.58E-17 | Lung |
| 4973 | OLR1 | -2.089280171 | 7.61E-24 | Lung |
| 5918 | RARRES1 | -2.088783585 | 5.02E-25 | Lung |
| 719 | C3AR1 | -2.088500758 | 4.41E-38 | Lung |
| 1438 | CSF2RA | -2.087377054 | 4.10E-29 | Lung |
| 202309 | GAPT | -2.086468224 | 6.86E-30 | Lung |
| 81501 | DCSTAMP | -2.085055771 | 8.81E-18 | Lung |
| 150372 | NFAM1 | -2.084845389 | 1.09E-37 | Lung |
| 6358 | CCL14 | -2.084184674 | 3.60E-17 | Lung |
| 285180 | RUFY4 | -2.083040882 | 3.23E-17 | Lung |
| 27036 | SIGLEC7 | -2.081342688 | 1.21E-32 | Lung |
| 8778 | SIGLEC5 | -2.079843566 | 4.27E-28 | Lung |
| 5734 | PTGER4 | -2.079525201 | 1.18E-41 | Lung |
| 11026 | None | -2.078739658 | 5.19E-17 | Lung |
| 2206 | MS4A2 | -2.078278813 | 2.60E-15 | Lung |
| 284759 | SIRPB2 | -2.076662632 | 4.87E-32 | Lung |
| 127795 | C1orf87 | -2.075912675 | 9.83E-10 | Lung |
| 3119 | HLA-DQB1 | -2.075454796 | 7.16E-22 | Lung |
| 27180 | SIGLEC9 | -2.075097222 | 6.79E-33 | Lung |
| 3868 | KRT16 | -2.074007223 | 1.41E-07 | Lung |
| 9450 | LY86 | -2.073051454 | 9.72E-34 | Lung |
| 64926 | RASAL3 | -2.072473096 | 4.60E-46 | Lung |
| 8530 | CST7 | -2.072343101 | 2.21E-32 | Lung |
| 3766 | KCNJ10 | -2.071966381 | 6.64E-18 | Lung |
| 909 | CD1A | -2.071749781 | 4.49E-09 | Lung |
| 9046 | DOK2 | -2.071361458 | 4.07E-42 | Lung |
| 285025 | CCDC141 | -2.069387024 | 6.90E-20 | Lung |
| 284021 | MILR1 | -2.068508412 | 2.32E-28 | Lung |
| 26051 | PPP1R16B | -2.067501897 | 1.95E-37 | Lung |
| 8755 | None | -2.066443157 | 3.20E-15 | Lung |
| 64174 | DPEP2 | -2.066311402 | 8.25E-32 | Lung |
| 2124 | EVI2B | -2.066111488 | 2.91E-43 | Lung |
| 923 | CD6 | -2.065979113 | 4.46E-39 | Lung |
| 93978 | CLEC6A | -2.065496548 | 8.56E-23 | Lung |
| 6769 | STAC | -2.065069127 | 6.97E-16 | Lung |
| 3762 | KCNJ5 | -2.064401943 | 9.31E-19 | Lung |
| 64098 | PARVG | -2.061996847 | 4.46E-44 | Lung |
| 245972 | ATP6V0D2 | -2.060457827 | 1.31E-18 | Lung |
| 79168 | LILRA6 | -2.060243142 | 1.55E-28 | Lung |
| 1240 | CMKLR1 | -2.059438628 | 5.80E-38 | Lung |
| 3512 | JCHAIN | -2.059177641 | 2.11E-14 | Lung |
| 51338 | MS4A4A | -2.058477029 | 3.72E-33 | Lung |
| 64581 | CLEC7A | -2.057972681 | 7.47E-32 | Lung |
| 171558 | PTCRA | -2.056363661 | 4.62E-26 | Lung |
| 401551 | WDR38 | -2.056115087 | 2.35E-08 | Lung |
| 3587 | IL10RA | -2.055575838 | 6.52E-50 | Lung |
| 3553 | IL1B | -2.054736535 | 6.08E-25 | Lung |
| 221472 | FGD2 | -2.054430789 | 1.97E-42 | Lung |
| 2359 | FPR3 | -2.051043671 | 1.43E-33 | Lung |
| 91851 | CHRDL1 | -2.049950102 | 3.54E-13 | Lung |
| 1880 | GPR183 | -2.048147687 | 1.05E-34 | Lung |
| 2327 | FMO2 | -2.047460228 | 6.34E-22 | Lung |
| 3363 | HTR7 | -2.044465533 | 1.04E-26 | Lung |
| 259307 | IL4I1 | -2.044446582 | 2.15E-29 | Lung |
| 3598 | IL13RA2 | -2.044070939 | 5.60E-12 | Lung |
| 27128 | CYTH4 | -2.043259724 | 5.93E-49 | Lung |
| 7940 | LST1 | -2.041474854 | 9.33E-33 | Lung |
| 5521 | PPP2R2B | -2.040525534 | 8.99E-27 | Lung |
| 115650 | TNFRSF13C | -2.039965957 | 1.41E-19 | Lung |
| 6442 | SGCA | -2.038874138 | 1.69E-16 | Lung |
| 3684 | ITGAM | -2.03790762 | 1.39E-30 | Lung |
| 54716 | SLC6A20 | -2.037486758 | 4.15E-08 | Lung |
| 165631 | PARP15 | -2.036209229 | 1.83E-22 | Lung |
| 2214 | FCGR3A | -2.035281633 | 1.75E-30 | Lung |
| 3561 | IL2RG | -2.032026034 | 3.38E-36 | Lung |
| 90273 | CEACAM21 | -2.030218347 | 3.73E-25 | Lung |
| 2833 | CXCR3 | -2.028625747 | 3.94E-33 | Lung |
| 11024 | LILRA1 | -2.028311552 | 4.03E-31 | Lung |
| 3004 | GZMM | -2.027063783 | 1.89E-21 | Lung |
| 11262 | SP140 | -2.026695792 | 1.57E-41 | Lung |
| 4145 | MATK | -2.025555297 | 1.46E-33 | Lung |
| 3560 | IL2RB | -2.024629947 | 4.37E-37 | Lung |
| 4481 | MSR1 | -2.023727997 | 2.41E-27 | Lung |
| 946 | SIGLEC6 | -2.023633954 | 4.61E-17 | Lung |
| 3689 | ITGB2 | -2.023007033 | 6.37E-31 | Lung |
| 146722 | CD300LF | -2.021816623 | 1.61E-30 | Lung |
| 5579 | PRKCB | -2.020595278 | 1.37E-33 | Lung |
| 2167 | FABP4 | -2.020254405 | 3.30E-11 | Lung |
| 55340 | GIMAP5 | -2.018284217 | 8.72E-49 | Lung |
| 653509 | SFTPA1 | -2.017037509 | 9.22E-05 | Lung |
| 3581 | IL9R | -2.016514803 | 1.41E-33 | Lung |
| 942 | CD86 | -2.015662815 | 4.02E-40 | Lung |
| 30835 | CD209 | -2.01241307 | 5.97E-25 | Lung |
| 330 | BIRC3 | -2.012260287 | 1.75E-27 | Lung |
| 22854 | NTNG1 | -2.011580578 | 1.36E-11 | Lung |
| 60489 | APOBEC3G | -2.010603092 | 2.02E-36 | Lung |
| 84688 | C9orf24 | -2.008267763 | 2.45E-09 | Lung |
| 1731 | Sep-01 | -2.007523766 | 2.39E-37 | Lung |
| 3603 | IL16 | -2.005379674 | 1.20E-48 | Lung |
| 9173 | IL1RL1 | -2.005114175 | 4.28E-11 | Lung |
| 51738 | GHRL | -2.004671884 | 3.78E-23 | Lung |
| 165904 | XIRP1 | -2.004217116 | 5.43E-16 | Lung |
| 6693 | SPN | -2.003381073 | 2.62E-35 | Lung |
| 3792 | KEL | -2.001491109 | 7.96E-18 | Lung |
| 93082 | NEURL3 | -2.000695797 | 2.88E-14 | Lung |
| 10964 | IFI44L | -1.999996623 | 6.42E-17 | Lung |
| 2219 | FCN1 | -1.998626817 | 7.31E-22 | Lung |
| 199 | AIF1 | -1.997805947 | 1.26E-41 | Lung |
| 96610 | None | -1.99231021 | 2.42E-15 | Lung |
| 10326 | SIRPB1 | -1.991096249 | 1.85E-28 | Lung |
| 283420 | CLEC9A | -1.989247476 | 4.05E-23 | Lung |
| 100188949 | None | -1.988888569 | 2.07E-37 | Lung |
| 64399 | HHIP | -1.98888387 | 7.03E-07 | Lung |
| 9023 | CH25H | -1.987752271 | 5.24E-18 | Lung |
| 2268 | FGR | -1.987570512 | 3.25E-41 | Lung |
| 6518 | SLC2A5 | -1.987469206 | 3.68E-19 | Lung |
| 25984 | KRT23 | -1.986183238 | 4.36E-06 | Lung |
| 84174 | SLA2 | -1.985243459 | 1.80E-38 | Lung |
| 26585 | GREM1 | -1.984816761 | 9.32E-11 | Lung |
| 388372 | CCL4L2 | -1.984124597 | 2.77E-23 | Lung |
| 11309 | SLCO2B1 | -1.983763488 | 3.19E-33 | Lung |
| 79827 | CLMP | -1.983385196 | 3.29E-19 | Lung |
| 59285 | CACNG6 | -1.982703045 | 5.48E-10 | Lung |
| 9953 | HS3ST3B1 | -1.982434072 | 2.07E-25 | Lung |
| 150365 | MEI1 | -1.981561194 | 2.46E-26 | Lung |
| 64072 | CDH23 | -1.981178875 | 5.19E-23 | Lung |
| 5816 | PVALB | -1.981161741 | 4.88E-20 | Lung |
| 10154 | PLXNC1 | -1.981010478 | 1.04E-26 | Lung |
| 27071 | DAPP1 | -1.980448372 | 6.11E-29 | Lung |
| 3936 | LCP1 | -1.980150975 | 2.27E-38 | Lung |
| 7454 | WAS | -1.979572897 | 3.37E-46 | Lung |
| 5172 | SLC26A4 | -1.978756155 | 9.78E-10 | Lung |
| 51744 | CD244 | -1.97761535 | 5.73E-33 | Lung |
| 219972 | MPEG1 | -1.976476813 | 1.47E-38 | Lung |
| 10870 | HCST | -1.97643904 | 2.50E-46 | Lung |
| 972 | CD74 | -1.975758046 | 6.25E-30 | Lung |
| 4969 | OGN | -1.973579483 | 8.42E-12 | Lung |
| 241 | ALOX5AP | -1.972258162 | 8.54E-30 | Lung |
| 9595 | CYTIP | -1.971233043 | 1.26E-40 | Lung |
| 118788 | PIK3AP1 | -1.967291905 | 8.54E-36 | Lung |
| 64005 | MYO1G | -1.966664543 | 3.66E-21 | Lung |
| 57091 | CASS4 | -1.966623888 | 4.61E-31 | Lung |
| 1237 | CCR8 | -1.966242578 | 5.53E-30 | Lung |
| 348378 | SHISAL2A | -1.965639791 | 6.94E-27 | Lung |
| 440738 | MAP1LC3C | -1.964691475 | 1.35E-13 | Lung |
| 6503 | SLA | -1.964530491 | 3.79E-44 | Lung |
| 1117 | CHI3L2 | -1.964378362 | 3.57E-12 | Lung |
| 4239 | MFAP4 | -1.96397149 | 1.53E-20 | Lung |
| 6688 | SPI1 | -1.963862397 | 6.70E-36 | Lung |
| 3687 | ITGAX | -1.963540576 | 5.83E-37 | Lung |
| 348938 | NIPAL4 | -1.962937451 | 2.05E-16 | Lung |
| 23533 | PIK3R5 | -1.961985926 | 2.50E-41 | Lung |
| 1511 | CTSG | -1.961845735 | 2.73E-13 | Lung |
| 375686 | SPATC1 | -1.961327226 | 1.87E-36 | Lung |
| 2672 | GFI1 | -1.960993571 | 2.75E-35 | Lung |
| 54988 | ACSM5 | -1.960896148 | 2.15E-20 | Lung |
| 51411 | BIN2 | -1.960715713 | 1.90E-41 | Lung |
| 1380 | CR2 | -1.960489678 | 3.18E-10 | Lung |
| 7305 | TYROBP | -1.958417037 | 8.62E-35 | Lung |
| 4489 | MT1A | -1.958174048 | 1.28E-10 | Lung |
| 1436 | CSF1R | -1.957731628 | 9.83E-33 | Lung |
| 7805 | LAPTM5 | -1.957229653 | 7.10E-42 | Lung |
| 7512 | XPNPEP2 | -1.956923399 | 7.22E-19 | Lung |
| 83706 | FERMT3 | -1.956308042 | 7.08E-45 | Lung |
| 126014 | OSCAR | -1.953789754 | 1.31E-30 | Lung |
| 3823 | KLRC3 | -1.952846894 | 8.43E-19 | Lung |
| 6489 | ST8SIA1 | -1.952252905 | 1.30E-26 | Lung |
| 29992 | PILRA | -1.950229926 | 2.50E-34 | Lung |
| 8787 | RGS9 | -1.950002158 | 1.26E-23 | Lung |
| 165186 | TOGARAM2 | -1.94842218 | 9.59E-17 | Lung |
| 4210 | MEFV | -1.947723518 | 3.87E-31 | Lung |
| 5729 | PTGDR | -1.947396403 | 2.06E-30 | Lung |
| 199675 | MCEMP1 | -1.947215596 | 2.92E-11 | Lung |
| 29909 | GPR171 | -1.946837904 | 9.97E-20 | Lung |
| 4064 | CD180 | -1.946703613 | 6.35E-33 | Lung |
| 729238 | SFTPA2 | -1.946310292 | 6.32E-05 | Lung |
| 3937 | LCP2 | -1.945547923 | 2.04E-47 | Lung |
| 6348 | CCL3 | -1.94280629 | 1.19E-26 | Lung |
| 79626 | TNFAIP8L2 | -1.941691491 | 7.76E-37 | Lung |
| 9214 | FCMR | -1.940805834 | 1.09E-35 | Lung |
| 3822 | KLRC2 | -1.939837069 | 8.80E-16 | Lung |
| 58511 | DNASE2B | -1.938374414 | 2.18E-14 | Lung |
| 9635 | CLCA2 | -1.93699517 | 5.50E-11 | Lung |
| 3135 | HLA-G | -1.936528259 | 2.50E-17 | Lung |
| 113730 | KLHDC7B | -1.934186146 | 1.45E-21 | Lung |
| 9402 | GRAP2 | -1.933728154 | 1.84E-29 | Lung |
| 200315 | APOBEC3A | -1.93341986 | 6.52E-20 | Lung |
| 58475 | MS4A7 | -1.932471432 | 2.15E-34 | Lung |
| 5023 | P2RX1 | -1.930663281 | 1.22E-25 | Lung |
| 6703 | SPRR2D | -1.92949386 | 5.57E-08 | Lung |
| 5996 | RGS1 | -1.928219757 | 4.87E-26 | Lung |
| 80122 | MAP3K19 | -1.927843601 | 4.39E-09 | Lung |
| 27306 | HPGDS | -1.927251469 | 1.85E-15 | Lung |
| 440712 | RHEX | -1.926811785 | 2.26E-15 | Lung |
| 389799 | CFAP77 | -1.924962927 | 2.91E-09 | Lung |
| 7412 | VCAM1 | -1.922825921 | 4.58E-27 | Lung |
| 414236 | C10orf55 | -1.922583392 | 3.46E-21 | Lung |
| 2823 | GPM6A | -1.918330142 | 1.49E-11 | Lung |
| 115727 | RASGRP4 | -1.917950043 | 1.10E-31 | Lung |
| 5328 | PLAU | -1.917248221 | 4.25E-17 | Lung |
| 5551 | PRF1 | -1.916874726 | 1.32E-29 | Lung |
| 84106 | PRAM1 | -1.916125663 | 4.13E-25 | Lung |
| 8200 | GDF5 | -1.912316572 | 2.84E-12 | Lung |
| 11025 | LILRB3 | -1.912003998 | 2.23E-35 | Lung |
| 57817 | HAMP | -1.911656356 | 2.08E-14 | Lung |
| 2847 | MCHR1 | -1.911104777 | 4.47E-19 | Lung |
| 366 | AQP9 | -1.910051493 | 5.44E-17 | Lung |
| 161176 | SYNE3 | -1.909803861 | 2.42E-27 | Lung |
| 1620 | BRINP1 | -1.908202962 | 7.08E-06 | Lung |
| 3371 | TNC | -1.907653111 | 3.55E-13 | Lung |
| 8516 | ITGA8 | -1.905982917 | 1.22E-13 | Lung |
| 219670 | ENKUR | -1.905933934 | 1.50E-10 | Lung |
| 1235 | CCR6 | -1.904693904 | 1.75E-30 | Lung |
| 128346 | C1orf162 | -1.904589054 | 1.13E-36 | Lung |
| 146850 | PIK3R6 | -1.903558713 | 3.25E-29 | Lung |
| 55016 | Mar-01 | -1.902892076 | 1.62E-38 | Lung |
| 920 | CD4 | -1.902375893 | 3.35E-40 | Lung |
| 8605 | PLA2G4C | -1.901115407 | 7.57E-30 | Lung |
| 64446 | DNAI2 | -1.899968838 | 3.25E-08 | Lung |
| 145864 | HAPLN3 | -1.899879237 | 2.38E-37 | Lung |
| 270 | AMPD1 | -1.89913018 | 1.15E-12 | Lung |
| 2207 | FCER1G | -1.899029525 | 2.07E-35 | Lung |
| 6559 | SLC12A3 | -1.898572187 | 2.04E-21 | Lung |
| 4689 | NCF4 | -1.898192777 | 1.94E-35 | Lung |
| 958 | CD40 | -1.896920092 | 1.90E-35 | Lung |
| 3134 | HLA-F | -1.896630596 | 3.87E-35 | Lung |
| 7099 | TLR4 | -1.895973468 | 5.74E-41 | Lung |
| 164781 | DAW1 | -1.895448806 | 2.73E-09 | Lung |
| 794 | CALB2 | -1.894642844 | 2.90E-09 | Lung |
| 5348 | FXYD1 | -1.894606717 | 2.27E-15 | Lung |
| 2709 | GJB5 | -1.894055868 | 5.06E-08 | Lung |
| 433 | ASGR2 | -1.891428993 | 1.55E-15 | Lung |
| 389384 | C6orf222 | -1.889497752 | 3.90E-08 | Lung |
| 153769 | SH3RF2 | -1.887286646 | 7.32E-10 | Lung |
| 8809 | IL18R1 | -1.886945166 | 3.63E-30 | Lung |
| 8676 | STX11 | -1.884367619 | 1.02E-38 | Lung |
| 240 | ALOX5 | -1.880759292 | 1.10E-28 | Lung |
| 26166 | RGS22 | -1.880375031 | 2.44E-11 | Lung |
| 167838 | TXLNB | -1.879748117 | 5.74E-24 | Lung |
| 23430 | TPSD1 | -1.878225556 | 2.78E-09 | Lung |
| 56670 | SUCNR1 | -1.877009984 | 2.18E-11 | Lung |
| 221393 | ADGRF4 | -1.876604936 | 7.05E-08 | Lung |
| 27040 | LAT | -1.875474465 | 2.61E-40 | Lung |
| 5199 | CFP | -1.872787187 | 1.80E-29 | Lung |
| 83894 | TTC29 | -1.872607167 | 3.40E-09 | Lung |
| 5920 | RARRES3 | -1.872063116 | 1.48E-20 | Lung |
| 284654 | RSPO1 | -1.869084155 | 3.26E-17 | Lung |
| 2326 | FMO1 | -1.868196088 | 3.23E-15 | Lung |
| 80117 | ARL14 | -1.867459057 | 5.39E-07 | Lung |
| 92241 | RCSD1 | -1.866372557 | 2.57E-41 | Lung |
| 114827 | FHAD1 | -1.865981033 | 3.14E-18 | Lung |
| 1308 | COL17A1 | -1.864077933 | 1.07E-05 | Lung |
| 341 | APOC1 | -1.863507517 | 3.49E-21 | Lung |
| 978 | CDA | -1.862555663 | 4.07E-10 | Lung |
| 219855 | SLC37A2 | -1.860594343 | 2.07E-26 | Lung |
| 864 | RUNX3 | -1.860483903 | 1.31E-34 | Lung |
| 27197 | GPR82 | -1.859905714 | 3.21E-21 | Lung |
| 90019 | SYT8 | -1.858262158 | 4.76E-07 | Lung |
| 2857 | GPR34 | -1.85725238 | 5.55E-27 | Lung |
| 1805 | DPT | -1.856950678 | 7.20E-18 | Lung |
| 54209 | TREM2 | -1.856399288 | 5.44E-24 | Lung |
| 219285 | SAMD9L | -1.856150486 | 2.24E-33 | Lung |
| 929 | CD14 | -1.855828783 | 3.00E-34 | Lung |
| 79365 | BHLHE41 | -1.855253898 | 4.47E-27 | Lung |
| 9535 | GMFG | -1.854682312 | 7.81E-46 | Lung |
| 834 | CASP1 | -1.853251016 | 1.38E-41 | Lung |
| 186 | AGTR2 | -1.852892723 | 7.18E-06 | Lung |
| 6404 | SELPLG | -1.851705393 | 1.14E-37 | Lung |
| 440823 | None | -1.851034824 | 4.33E-14 | Lung |
| 257106 | ARHGAP30 | -1.849600383 | 9.38E-48 | Lung |
| 5737 | PTGFR | -1.849265521 | 1.78E-12 | Lung |
| 11010 | GLIPR1 | -1.848255727 | 1.26E-40 | Lung |
| 8626 | TP63 | -1.847596974 | 2.63E-10 | Lung |
| 80231 | CXorf21 | -1.847446905 | 1.69E-33 | Lung |
| 79740 | ZBBX | -1.84706304 | 2.71E-08 | Lung |
| 3696 | ITGB8 | -1.847009905 | 9.33E-11 | Lung |
| 388011 | None | -1.846845585 | 1.96E-14 | Lung |
| 80086 | TUBA4B | -1.845750147 | 5.66E-08 | Lung |
| 256236 | None | -1.845132025 | 1.79E-21 | Lung |
| 55026 | TMEM255A | -1.842392673 | 1.42E-17 | Lung |
| 55303 | GIMAP4 | -1.840971558 | 4.62E-50 | Lung |
| 4542 | MYO1F | -1.840572397 | 8.03E-45 | Lung |
| 345895 | RSPH4A | -1.840275712 | 3.03E-10 | Lung |
| 85480 | TSLP | -1.84008303 | 4.70E-16 | Lung |
| 80833 | APOL3 | -1.839883513 | 9.38E-42 | Lung |
| 5148 | None | -1.839762484 | 2.20E-24 | Lung |
| 730112 | FAM166B | -1.83867797 | 6.40E-11 | Lung |
| 89795 | NAV3 | -1.837846143 | 9.67E-22 | Lung |
| 54435 | None | -1.836773009 | 9.14E-13 | Lung |
| 3106 | HLA-B | -1.836675578 | 1.55E-40 | Lung |
| 284434 | NWD1 | -1.834988104 | 2.73E-07 | Lung |
| 1521 | CTSW | -1.833699448 | 1.68E-19 | Lung |
| 79444 | BIRC7 | -1.833204411 | 4.57E-14 | Lung |
| 924 | CD7 | -1.833186489 | 1.66E-25 | Lung |
| 9363 | RAB33A | -1.832774945 | 2.60E-36 | Lung |
| 152007 | GLIPR2 | -1.832421758 | 5.30E-39 | Lung |
| 203190 | LGI3 | -1.831455846 | 2.32E-06 | Lung |
| 4688 | NCF2 | -1.829439597 | 5.54E-36 | Lung |
| 126364 | LRRC25 | -1.827425147 | 2.60E-32 | Lung |
| 170575 | GIMAP1 | -1.827385967 | 5.22E-43 | Lung |
| 6349 | CCL3L1 | -1.826334163 | 2.99E-15 | Lung |
| 5698 | PSMB9 | -1.826274684 | 1.30E-32 | Lung |
| 168537 | GIMAP7 | -1.825524234 | 4.19E-42 | Lung |
| 348 | APOE | -1.825118418 | 5.56E-22 | Lung |
| 3037 | HAS2 | -1.82334439 | 1.22E-16 | Lung |
| 23601 | CLEC5A | -1.821339582 | 1.01E-18 | Lung |
| 1960 | EGR3 | -1.819172493 | 3.37E-24 | Lung |
| 27019 | DNAI1 | -1.81887947 | 1.10E-07 | Lung |
| 9242 | MSC | -1.81833746 | 9.63E-30 | Lung |
| 2919 | CXCL1 | -1.817354217 | 3.22E-13 | Lung |
| 197358 | NLRC3 | -1.816655955 | 8.98E-41 | Lung |
| 8740 | TNFSF14 | -1.816428897 | 5.62E-18 | Lung |
| 56659 | KCNK13 | -1.812912531 | 1.66E-25 | Lung |
| 6039 | RNASE6 | -1.812675418 | 8.46E-41 | Lung |
| 54504 | CPVL | -1.810815858 | 1.48E-22 | Lung |
| 51561 | IL23A | -1.810733363 | 1.61E-19 | Lung |
| 5540 | NPY4R | -1.810684965 | 2.33E-10 | Lung |
| 4916 | NTRK3 | -1.810385424 | 5.79E-18 | Lung |
| 84830 | ADTRP | -1.809187396 | 2.58E-17 | Lung |
| 5806 | PTX3 | -1.809087051 | 2.36E-16 | Lung |
| 127534 | GJB4 | -1.808703226 | 7.14E-08 | Lung |
| 167359 | NIM1K | -1.808347784 | 1.55E-13 | Lung |
| 8522 | GAS7 | -1.806101287 | 6.47E-33 | Lung |
| 57105 | CYSLTR2 | -1.80506202 | 3.69E-30 | Lung |
| 116986 | AGAP2 | -1.803747903 | 1.67E-37 | Lung |
| 2681 | None | -1.803564042 | 3.63E-31 | Lung |
| 84417 | C2orf40 | -1.8023386 | 7.54E-09 | Lung |
| 1116 | CHI3L1 | -1.802053496 | 2.97E-14 | Lung |
| 158248 | TTC16 | -1.801682927 | 2.63E-15 | Lung |
| 146177 | VWA3A | -1.801206637 | 1.77E-08 | Lung |
| 353514 | LILRA5 | -1.799740483 | 9.33E-24 | Lung |
| 201625 | DNAH12 | -1.79938069 | 9.84E-08 | Lung |
| 12 | SERPINA3 | -1.797977033 | 1.43E-07 | Lung |
| 342510 | CD300E | -1.797054493 | 6.30E-17 | Lung |
| 5099 | PCDH7 | -1.796280005 | 4.24E-13 | Lung |
| 79865 | TREML2 | -1.796217117 | 1.33E-20 | Lung |
| 93035 | PKHD1L1 | -1.794633545 | 2.76E-14 | Lung |
| 3699 | ITIH3 | -1.793640216 | 7.16E-19 | Lung |
| 3738 | KCNA3 | -1.791593922 | 3.86E-15 | Lung |
| 254956 | MORN5 | -1.79018966 | 3.72E-08 | Lung |
| 11151 | CORO1A | -1.788867474 | 1.89E-45 | Lung |
| 1230 | CCR1 | -1.788449815 | 9.67E-30 | Lung |
| 2674 | GFRA1 | -1.786445683 | 1.49E-09 | Lung |
| 10351 | ABCA8 | -1.785631489 | 7.75E-10 | Lung |
| 54518 | APBB1IP | -1.785503873 | 6.60E-31 | Lung |
| 10457 | GPNMB | -1.783630566 | 4.97E-27 | Lung |
| 729 | C6 | -1.783070474 | 4.33E-07 | Lung |
| 57699 | CPNE5 | -1.782272084 | 8.37E-23 | Lung |
| 346171 | ZFP57 | -1.781906712 | 1.41E-08 | Lung |
| 126410 | CYP4F22 | -1.781699061 | 1.94E-21 | Lung |
| 114769 | CARD16 | -1.778877947 | 3.37E-31 | Lung |
| 7128 | TNFAIP3 | -1.778269403 | 4.98E-40 | Lung |
| 151790 | WDR49 | -1.777711424 | 4.02E-10 | Lung |
| 55619 | DOCK10 | -1.776976602 | 5.21E-28 | Lung |
| 1414 | CRYBB1 | -1.776956455 | 2.13E-21 | Lung |
| 149483 | CCDC17 | -1.775882562 | 1.11E-10 | Lung |
| 7941 | PLA2G7 | -1.774886478 | 2.68E-22 | Lung |
| 10223 | GPA33 | -1.772009209 | 7.65E-12 | Lung |
| 445347 | TRGC1 | -1.771366395 | 2.88E-15 | Lung |
| 283316 | CD163L1 | -1.771050694 | 5.06E-18 | Lung |
| 284348 | LYPD5 | -1.770642037 | 3.23E-18 | Lung |
| 11009 | IL24 | -1.769674978 | 3.44E-20 | Lung |
| 9935 | MAFB | -1.769387467 | 9.44E-44 | Lung |
| 9459 | ARHGEF6 | -1.767002668 | 4.12E-46 | Lung |
| 64919 | BCL11B | -1.766920709 | 5.52E-21 | Lung |
| 5452 | POU2F2 | -1.766553953 | 5.39E-28 | Lung |
| 6317 | SERPINB3 | -1.765346171 | 0.000189926 | Lung |
| 58484 | NLRC4 | -1.765337439 | 3.61E-36 | Lung |
| 3861 | KRT14 | -1.764668785 | 1.21E-06 | Lung |
| 94240 | EPSTI1 | -1.763683572 | 3.23E-27 | Lung |
| 3854 | KRT6B | -1.7634031 | 5.08E-05 | Lung |
| 7462 | LAT2 | -1.763220622 | 5.15E-34 | Lung |
| 55130 | ARMC4 | -1.76151236 | 1.55E-08 | Lung |
| 8528 | DDO | -1.761295666 | 4.50E-19 | Lung |
| 1294 | COL7A1 | -1.760417066 | 1.66E-08 | Lung |
| 353345 | GPR141 | -1.760368018 | 1.49E-19 | Lung |
| 154 | ADRB2 | -1.759868332 | 1.41E-18 | Lung |
| 147372 | CCBE1 | -1.759238481 | 8.05E-10 | Lung |
| 943 | TNFRSF8 | -1.758508953 | 1.09E-29 | Lung |
| 114132 | SIGLEC11 | -1.757120768 | 1.23E-19 | Lung |
| 8728 | ADAM19 | -1.757078348 | 2.39E-29 | Lung |
| 10630 | PDPN | -1.756953247 | 6.31E-20 | Lung |
| 912 | CD1D | -1.756478992 | 4.03E-29 | Lung |
| 340547 | VSIG1 | -1.754384504 | 2.83E-05 | Lung |
| 286530 | P2RY8 | -1.754115466 | 1.62E-25 | Lung |
| 6943 | TCF21 | -1.754089459 | 2.28E-15 | Lung |
| 2215 | FCGR3B | -1.753724054 | 5.09E-13 | Lung |
| 5880 | RAC2 | -1.75338829 | 2.67E-41 | Lung |
| 6583 | SLC22A4 | -1.753238864 | 1.96E-23 | Lung |
| 3039 | HBA1 | -1.752665972 | 8.66E-06 | Lung |
| 1475 | CSTA | -1.751986676 | 1.42E-20 | Lung |
| 137835 | TMEM71 | -1.751830148 | 6.69E-20 | Lung |
| 84541 | KBTBD8 | -1.751820935 | 5.17E-36 | Lung |
| 83661 | MS4A8 | -1.751706402 | 6.40E-05 | Lung |
| 6101 | RP1 | -1.751201769 | 1.80E-07 | Lung |
| 2915 | GRM5 | -1.750999266 | 5.51E-09 | Lung |
| 5552 | SRGN | -1.749427147 | 9.05E-29 | Lung |
| 286336 | FAM78A | -1.749168715 | 4.34E-43 | Lung |
| 9945 | GFPT2 | -1.747383147 | 8.28E-24 | Lung |
| 5322 | PLA2G5 | -1.747299802 | 2.36E-24 | Lung |
| 339390 | CLEC4G | -1.746369221 | 1.20E-14 | Lung |
| 283663 | None | -1.744777872 | 1.92E-17 | Lung |
| 645432 | ARRDC5 | -1.744706902 | 4.57E-25 | Lung |
| 8110 | DPF3 | -1.744487334 | 3.15E-22 | Lung |
| 80852 | GRIP2 | -1.744396449 | 2.62E-18 | Lung |
| 221091 | LRRN4CL | -1.744196033 | 2.70E-21 | Lung |
| 352961 | None | -1.743899017 | 1.03E-26 | Lung |
| 9750 | RIPOR2 | -1.743844527 | 6.97E-23 | Lung |
| 139716 | GAB3 | -1.743591021 | 1.71E-39 | Lung |
| 340205 | TREML1 | -1.742165355 | 7.63E-24 | Lung |
| 92211 | CDHR1 | -1.742161388 | 4.33E-16 | Lung |
| 56938 | ARNTL2 | -1.741955087 | 5.83E-14 | Lung |
| 6367 | CCL22 | -1.741951897 | 8.93E-16 | Lung |
| 4046 | LSP1 | -1.740027187 | 1.39E-31 | Lung |
| 285596 | FAM153A | -1.737091105 | 2.33E-10 | Lung |
| 474344 | GIMAP6 | -1.736058241 | 2.73E-40 | Lung |
| 6280 | S100A9 | -1.734593033 | 8.63E-10 | Lung |
| 3600 | IL15 | -1.73343551 | 1.23E-29 | Lung |
| 1001 | CDH3 | -1.732841758 | 1.15E-09 | Lung |
| 27092 | CACNG4 | -1.732508177 | 5.67E-06 | Lung |
| 170690 | ADAMTS16 | -1.731131787 | 1.24E-10 | Lung |
| 2172 | FABP6 | -1.729574585 | 1.40E-07 | Lung |
| 4958 | OMD | -1.729252087 | 5.82E-11 | Lung |
| 4693 | NDP | -1.726452641 | 2.36E-08 | Lung |
| 1437 | CSF2 | -1.725954205 | 1.12E-09 | Lung |
| 1959 | EGR2 | -1.725942686 | 3.10E-29 | Lung |
| 276 | AMY1A | -1.724575048 | 8.64E-06 | Lung |
| 3805 | KIR2DL4 | -1.723740839 | 2.09E-11 | Lung |
| 201305 | SPNS3 | -1.723403353 | 1.48E-19 | Lung |
| 5008 | OSM | -1.72302283 | 1.02E-18 | Lung |
| 64170 | CARD9 | -1.722416371 | 1.03E-20 | Lung |
| 3569 | IL6 | -1.721536385 | 9.97E-13 | Lung |
| 389206 | BEND4 | -1.721203227 | 4.65E-13 | Lung |
| 361 | AQP4 | -1.721022397 | 0.000118398 | Lung |
| 84689 | None | -1.720079987 | 8.90E-23 | Lung |
| 3574 | IL7 | -1.71987275 | 5.29E-23 | Lung |
| 54626 | HES2 | -1.718715272 | 2.08E-13 | Lung |
| 246329 | STAC3 | -1.718361789 | 2.21E-29 | Lung |
| 339524 | None | -1.717994857 | 1.41E-29 | Lung |
| 118932 | ANKRD22 | -1.714761015 | 2.46E-13 | Lung |
| 80114 | BICC1 | -1.714501829 | 2.75E-13 | Lung |
| 146206 | CARMIL2 | -1.713732937 | 7.54E-14 | Lung |
| 4319 | MMP10 | -1.713610941 | 1.32E-05 | Lung |
| 9290 | GPR55 | -1.712165182 | 1.09E-23 | Lung |
| 53832 | IL20RA | -1.711511627 | 2.37E-11 | Lung |
| 399 | RHOH | -1.711393691 | 7.34E-24 | Lung |
| 2829 | XCR1 | -1.710373497 | 1.17E-16 | Lung |
| 2354 | FOSB | -1.709496758 | 7.16E-08 | Lung |
| 420 | ART4 | -1.709100536 | 8.92E-15 | Lung |
| 51393 | TRPV2 | -1.708292099 | 5.50E-38 | Lung |
| 1318 | SLC31A2 | -1.704715953 | 7.54E-50 | Lung |
| 3595 | IL12RB2 | -1.703977313 | 3.91E-12 | Lung |
| 2273 | FHL1 | -1.703815776 | 1.54E-19 | Lung |
| 92747 | BPIFB1 | -1.703365058 | 0.001565075 | Lung |
| 140885 | SIRPA | -1.703134855 | 5.03E-32 | Lung |
| 13 | AADAC | -1.702183134 | 2.39E-06 | Lung |
| 10216 | PRG4 | -1.699028734 | 1.23E-07 | Lung |
| 26157 | GIMAP2 | -1.698584347 | 1.17E-29 | Lung |
| 3695 | ITGB7 | -1.697846425 | 5.92E-34 | Lung |
| 57471 | ERMN | -1.696382348 | 1.74E-17 | Lung |
| 286002 | None | -1.695808293 | 5.41E-08 | Lung |
| 3557 | IL1RN | -1.695340209 | 6.32E-18 | Lung |
| 168090 | C6orf118 | -1.694971916 | 3.39E-08 | Lung |
| 653567 | TMEM236 | -1.694514662 | 4.92E-17 | Lung |
| 401612 | SLC25A53 | -1.693707009 | 1.42E-29 | Lung |
| 6423 | SFRP2 | -1.69280526 | 7.49E-10 | Lung |
| 8482 | SEMA7A | -1.692300746 | 3.69E-23 | Lung |
| 79686 | None | -1.691497463 | 4.78E-29 | Lung |
| 117157 | SH2D1B | -1.691195779 | 2.77E-17 | Lung |
| 4312 | MMP1 | -1.690391477 | 1.18E-06 | Lung |
| 1513 | CTSK | -1.690296054 | 1.94E-23 | Lung |
| 202 | CRYBG1 | -1.689539741 | 1.12E-20 | Lung |
| 120939 | TMEM52B | -1.688056834 | 2.37E-23 | Lung |
| 121355 | GTSF1 | -1.687104399 | 4.39E-07 | Lung |
| 83853 | ROPN1L | -1.686590145 | 1.28E-07 | Lung |
| 9976 | CLEC2B | -1.686059711 | 2.80E-29 | Lung |
| 255809 | C19orf38 | -1.686043409 | 5.02E-29 | Lung |
| 55283 | MCOLN3 | -1.685435353 | 2.99E-11 | Lung |
| 728 | C5AR1 | -1.684356132 | 3.38E-28 | Lung |
| 64386 | MMP25 | -1.684244275 | 1.87E-28 | Lung |
| 6347 | CCL2 | -1.683285842 | 7.27E-26 | Lung |
| 2793 | GNGT2 | -1.683274991 | 6.78E-29 | Lung |
| 9955 | HS3ST3A1 | -1.682241168 | 5.18E-19 | Lung |
| 129293 | TRABD2A | -1.682025007 | 9.43E-15 | Lung |
| 56606 | SLC2A9 | -1.681690358 | 5.44E-34 | Lung |
| 150248 | C22orf15 | -1.681186722 | 5.28E-10 | Lung |
| 10800 | CYSLTR1 | -1.680221482 | 7.90E-19 | Lung |
| 140691 | TRIM69 | -1.679636323 | 2.11E-26 | Lung |
| 3059 | HCLS1 | -1.679563055 | 1.48E-30 | Lung |
| 79895 | ATP8B4 | -1.678620891 | 1.15E-33 | Lung |
| 1044 | CDX1 | -1.67766707 | 3.03E-21 | Lung |
| 8572 | PDLIM4 | -1.67744705 | 2.73E-15 | Lung |
| 284367 | None | -1.676936428 | 2.73E-16 | Lung |
| 79974 | CPED1 | -1.676724164 | 3.43E-23 | Lung |
| 7127 | TNFAIP2 | -1.675768136 | 6.96E-22 | Lung |
| 6387 | CXCL12 | -1.675753833 | 4.39E-23 | Lung |
| 6327 | SCN2B | -1.674671437 | 5.81E-15 | Lung |
| 355 | FAS | -1.67415451 | 1.19E-26 | Lung |
| 140862 | ISM1 | -1.672645085 | 6.88E-14 | Lung |
| 90865 | IL33 | -1.671860239 | 2.25E-14 | Lung |
| 126820 | WDR63 | -1.671197718 | 1.70E-09 | Lung |
| 140597 | TCEAL2 | -1.670462835 | 2.64E-12 | Lung |
| 80332 | ADAM33 | -1.669738707 | 4.39E-13 | Lung |
| 177 | AGER | -1.668941377 | 4.56E-07 | Lung |
| 2735 | GLI1 | -1.668688249 | 3.13E-14 | Lung |
| 8564 | KMO | -1.667460546 | 2.63E-25 | Lung |
| 25900 | IFFO1 | -1.667314764 | 3.79E-37 | Lung |
| 1634 | DCN | -1.66622101 | 1.94E-24 | Lung |
| 81704 | DOCK8 | -1.665390077 | 4.36E-33 | Lung |
| 338773 | TMEM119 | -1.664792409 | 9.33E-23 | Lung |
| 845 | CASQ2 | -1.664337212 | 1.31E-11 | Lung |
| 286887 | KRT6C | -1.662846672 | 1.28E-05 | Lung |
| 51296 | SLC15A3 | -1.661502205 | 4.41E-32 | Lung |
| 2995 | GYPC | -1.660871452 | 1.52E-41 | Lung |
| 57718 | PPP4R4 | -1.660571652 | 3.15E-10 | Lung |
| 2675 | GFRA2 | -1.660535668 | 5.66E-19 | Lung |
| 22806 | IKZF3 | -1.660185588 | 5.23E-17 | Lung |
| 51554 | ACKR4 | -1.659884583 | 5.23E-17 | Lung |
| 7409 | VAV1 | -1.658580491 | 1.89E-23 | Lung |
| 1687 | GSDME | -1.658087286 | 9.44E-19 | Lung |
| 2848 | GPR25 | -1.657105082 | 3.24E-18 | Lung |
| 3311 | None | -1.656994466 | 1.43E-17 | Lung |
| 7291 | TWIST1 | -1.655243768 | 2.28E-09 | Lung |
| 57722 | IGDCC4 | -1.654745588 | 1.03E-22 | Lung |
| 140 | ADORA3 | -1.654414597 | 5.54E-23 | Lung |
| 5026 | P2RX5 | -1.653626117 | 3.88E-20 | Lung |
| 653786 | None | -1.653493265 | 2.60E-15 | Lung |
| 10350 | ABCA9 | -1.653016394 | 2.23E-21 | Lung |
| 2014 | EMP3 | -1.652029745 | 5.31E-35 | Lung |
| 53833 | IL20RB | -1.651844955 | 1.13E-07 | Lung |
| 10164 | CHST4 | -1.649871896 | 7.21E-08 | Lung |
| 22943 | DKK1 | -1.648992187 | 0.000123241 | Lung |
| 91752 | ZNF804A | -1.648020897 | 2.92E-18 | Lung |
| 5104 | SERPINA5 | -1.64788269 | 8.33E-07 | Lung |
| 9056 | SLC7A7 | -1.647313726 | 1.87E-30 | Lung |
| 143903 | LAYN | -1.645402623 | 1.38E-23 | Lung |
| 2949 | GSTM5 | -1.643306935 | 6.10E-18 | Lung |
| 9806 | SPOCK2 | -1.64286063 | 4.38E-22 | Lung |
| 3742 | GALNT8 | -1.642335857 | 2.32E-14 | Lung |
| 246778 | IL27 | -1.641253524 | 4.39E-24 | Lung |
| 55350 | VNN3 | -1.640504938 | 5.72E-08 | Lung |
| 6036 | RNASE2 | -1.639726414 | 2.32E-17 | Lung |
| 11314 | CD300A | -1.639091408 | 1.31E-30 | Lung |
| 7490 | WT1 | -1.638217347 | 5.92E-10 | Lung |
| 160728 | SLC5A8 | -1.637889447 | 2.45E-05 | Lung |
| 149069 | DCDC2B | -1.636045782 | 6.49E-08 | Lung |
| 112755 | STX1B | -1.635535401 | 1.73E-18 | Lung |
| 3676 | ITGA4 | -1.633870678 | 1.15E-30 | Lung |
| 9938 | ARHGAP25 | -1.633860481 | 4.70E-48 | Lung |
| 66002 | CYP4F12 | -1.633743318 | 8.27E-07 | Lung |
| 8000 | PSCA | -1.633569466 | 0.000356149 | Lung |
| 200132 | TCTEX1D1 | -1.633350342 | 2.70E-16 | Lung |
| 80761 | None | -1.632869551 | 1.28E-06 | Lung |
| 6354 | CCL7 | -1.631133938 | 4.84E-09 | Lung |
| 158326 | FREM1 | -1.630682914 | 2.50E-10 | Lung |
| 7456 | WIPF1 | -1.630525769 | 6.86E-46 | Lung |
| 56062 | KLHL4 | -1.630303102 | 4.89E-15 | Lung |
| 223117 | SEMA3D | -1.629957014 | 4.98E-13 | Lung |
| 84419 | C15orf48 | -1.629916572 | 7.40E-10 | Lung |
| 1621 | DBH | -1.628197578 | 2.25E-15 | Lung |
| 56479 | KCNQ5 | -1.627821191 | 2.27E-08 | Lung |
| 6571 | SLC18A2 | -1.62409039 | 3.55E-09 | Lung |
| 9404 | LPXN | -1.623133183 | 1.32E-47 | Lung |
| 952 | CD38 | -1.623092718 | 4.09E-12 | Lung |
| 146433 | IL34 | -1.62234075 | 1.10E-19 | Lung |
| 400696 | None | -1.622174845 | 6.91E-12 | Lung |
| 120892 | LRRK2 | -1.621043675 | 1.43E-07 | Lung |
| 6332 | SCN7A | -1.620913133 | 3.66E-10 | Lung |
| 7294 | TXK | -1.62064186 | 2.46E-16 | Lung |
| 25939 | SAMHD1 | -1.62033859 | 1.79E-33 | Lung |
| 7185 | TRAF1 | -1.620258578 | 4.17E-47 | Lung |
| 133923 | ZNF474 | -1.619965514 | 2.02E-14 | Lung |
| 6441 | SFTPD | -1.619562418 | 1.81E-05 | Lung |
| 1833 | EPYC | -1.618476272 | 4.43E-07 | Lung |
| 1844 | DUSP2 | -1.617977031 | 2.62E-30 | Lung |
| 29933 | GPR132 | -1.617884182 | 2.07E-26 | Lung |
| 342897 | NCCRP1 | -1.617838771 | 2.57E-06 | Lung |
| 89780 | WNT3A | -1.616327032 | 6.27E-10 | Lung |
| 2625 | GATA3 | -1.615851411 | 7.26E-20 | Lung |
| 79037 | PVRIG | -1.615778766 | 1.87E-34 | Lung |
| 374872 | PEAK3 | -1.615739579 | 7.18E-26 | Lung |
| 1036 | CDO1 | -1.615678018 | 2.71E-15 | Lung |
| 145581 | LRFN5 | -1.615547483 | 2.69E-16 | Lung |
| 389634 | None | -1.615348823 | 2.02E-20 | Lung |
| 7852 | CXCR4 | -1.614914534 | 6.80E-35 | Lung |
| 6775 | STAT4 | -1.614082046 | 9.39E-28 | Lung |
| 3778 | KCNMA1 | -1.613390753 | 2.86E-17 | Lung |
| 644353 | ZCCHC18 | -1.61274019 | 5.70E-21 | Lung |
| 10346 | TRIM22 | -1.612377945 | 4.64E-30 | Lung |
| 133874 | C5orf58 | -1.612188102 | 1.61E-21 | Lung |
| 79987 | SVEP1 | -1.612153575 | 5.94E-18 | Lung |
| 84166 | NLRC5 | -1.611613346 | 2.78E-34 | Lung |
| 3043 | HBB | -1.610067441 | 7.01E-09 | Lung |
| 2205 | FCER1A | -1.609074251 | 7.18E-07 | Lung |
| 116535 | MRGPRF | -1.607984368 | 2.24E-24 | Lung |
| 2247 | FGF2 | -1.607604507 | 2.07E-18 | Lung |
| 10561 | IFI44 | -1.606671596 | 2.94E-16 | Lung |
| 91526 | ANKRD44 | -1.606289005 | 2.59E-21 | Lung |
| 133690 | CAPSL | -1.605907725 | 6.81E-06 | Lung |
| 5858 | PZP | -1.605727416 | 4.79E-07 | Lung |
| 79628 | SH3TC2 | -1.604458675 | 5.88E-12 | Lung |
| 83758 | RBP5 | -1.604453489 | 1.48E-24 | Lung |
| 54674 | LRRN3 | -1.603214305 | 1.80E-14 | Lung |
| 79605 | PGBD5 | -1.603211424 | 8.19E-14 | Lung |
| 716 | C1S | -1.602930958 | 9.93E-29 | Lung |
| 10866 | None | -1.601236742 | 1.21E-25 | Lung |
| 6274 | S100A3 | -1.60058127 | 2.25E-18 | Lung |
| 117581 | TWIST2 | -1.599867932 | 1.58E-14 | Lung |
| 144448 | TSPAN19 | -1.599836871 | 9.22E-07 | Lung |
| 3137 | None | -1.599140851 | 4.41E-16 | Lung |
| 9398 | CD101 | -1.598685617 | 7.47E-28 | Lung |
| 11213 | IRAK3 | -1.597718319 | 1.32E-18 | Lung |
| 81553 | FAM49A | -1.595001223 | 1.02E-23 | Lung |
| 710 | SERPING1 | -1.594937859 | 2.33E-36 | Lung |
| 140564 | APOBEC3D | -1.59409208 | 2.49E-27 | Lung |
| 149499 | LRRC71 | -1.593150686 | 8.80E-08 | Lung |
| 51225 | ABI3 | -1.593077388 | 7.28E-39 | Lung |
| 403314 | APOBEC4 | -1.592815124 | 2.00E-07 | Lung |
| 7079 | TIMP4 | -1.592382069 | 1.51E-09 | Lung |
| 64108 | RTP4 | -1.591933263 | 1.32E-22 | Lung |
| 3779 | KCNMB1 | -1.591896169 | 2.34E-21 | Lung |
| 7045 | TGFBI | -1.591485087 | 1.02E-15 | Lung |
| 7130 | TNFAIP6 | -1.590482032 | 1.65E-15 | Lung |
| 161003 | STOML3 | -1.588321502 | 2.18E-06 | Lung |
| 54507 | ADAMTSL4 | -1.587704038 | 1.94E-20 | Lung |
| 9770 | RASSF2 | -1.58751293 | 8.91E-33 | Lung |
| 2204 | FCAR | -1.586979408 | 7.08E-12 | Lung |
| 8302 | KLRC4 | -1.58691382 | 7.35E-16 | Lung |
| 3108 | HLA-DMA | -1.586821857 | 2.66E-24 | Lung |
| 1825 | DSC3 | -1.586394634 | 3.57E-06 | Lung |
| 150771 | ITPRIPL1 | -1.585993486 | 4.60E-23 | Lung |
| 131578 | LRRC15 | -1.585185822 | 3.28E-09 | Lung |
| 27287 | VENTX | -1.584539739 | 2.65E-18 | Lung |
| 6318 | SERPINB4 | -1.584363614 | 3.40E-05 | Lung |
| 6403 | SELP | -1.583234852 | 7.71E-15 | Lung |
| 2996 | GYPE | -1.582721446 | 2.55E-17 | Lung |
| 117144 | CATSPER1 | -1.582480425 | 2.83E-08 | Lung |
| 56729 | RETN | -1.581889641 | 1.05E-06 | Lung |
| 8840 | CCN4 | -1.580933911 | 3.25E-18 | Lung |
| 642987 | TMEM232 | -1.580870857 | 5.45E-08 | Lung |
| 22915 | MMRN1 | -1.58086716 | 2.84E-13 | Lung |
| 340152 | ZC3H12D | -1.580508831 | 2.37E-24 | Lung |
| 27319 | BHLHE22 | -1.578956894 | 2.62E-21 | Lung |
| 3635 | INPP5D | -1.578413288 | 5.70E-26 | Lung |
| 761 | CA3 | -1.577613431 | 8.42E-09 | Lung |
| 10100 | TSPAN2 | -1.576843757 | 7.21E-20 | Lung |
| 114905 | C1QTNF7 | -1.576584281 | 9.94E-11 | Lung |
| 92749 | DRC1 | -1.575336102 | 1.82E-05 | Lung |
| 51364 | ZMYND10 | -1.575000108 | 4.10E-07 | Lung |
| 89846 | FGD3 | -1.57417241 | 4.10E-32 | Lung |
| 8638 | OASL | -1.574003796 | 2.35E-15 | Lung |
| 8076 | MFAP5 | -1.573174115 | 1.16E-06 | Lung |
| 80896 | NPL | -1.57271162 | 7.00E-27 | Lung |
| 147138 | TMC8 | -1.572171899 | 1.39E-30 | Lung |
| 8190 | MIA | -1.572146001 | 2.23E-06 | Lung |
| 203328 | SUSD3 | -1.570877719 | 3.24E-26 | Lung |
| 4313 | MMP2 | -1.570416065 | 5.57E-23 | Lung |
| 9734 | HDAC9 | -1.570318993 | 7.26E-14 | Lung |
| 3579 | CXCR2 | -1.569352048 | 5.20E-14 | Lung |
| 1084 | CEACAM3 | -1.568527319 | 2.32E-15 | Lung |
| 155038 | GIMAP8 | -1.567675726 | 1.26E-32 | Lung |
| 79930 | DOK3 | -1.567639032 | 2.19E-31 | Lung |
| 79148 | MMP28 | -1.567468335 | 9.07E-07 | Lung |
| 55576 | STAB2 | -1.56720542 | 8.09E-11 | Lung |
| 9651 | PLCH2 | -1.56701331 | 2.94E-10 | Lung |
| 84969 | TOX2 | -1.566904285 | 3.66E-24 | Lung |
| 84951 | TNS4 | -1.566302394 | 1.08E-05 | Lung |
| 55816 | DOK5 | -1.565514113 | 4.12E-12 | Lung |
| 968 | CD68 | -1.564885176 | 7.71E-30 | Lung |
| 79630 | C1orf54 | -1.564450683 | 3.81E-33 | Lung |
| 6401 | SELE | -1.564156952 | 2.47E-11 | Lung |
| 5153 | PDE1B | -1.563917221 | 7.28E-23 | Lung |
| 9899 | SV2B | -1.563888824 | 1.55E-13 | Lung |
| 221336 | BEND6 | -1.561756789 | 8.86E-14 | Lung |
| 26002 | MOXD1 | -1.561545126 | 1.48E-18 | Lung |
| 558 | AXL | -1.560341962 | 2.63E-26 | Lung |
| 8082 | SSPN | -1.560265679 | 3.52E-24 | Lung |
| 80760 | ITIH5 | -1.559653892 | 4.79E-12 | Lung |
| 4118 | MAL | -1.559583848 | 1.21E-11 | Lung |
| 81035 | COLEC12 | -1.559459308 | 1.50E-15 | Lung |
| 79187 | FSD1 | -1.558806052 | 9.47E-09 | Lung |
| 4045 | LSAMP | -1.558782005 | 4.81E-19 | Lung |
| 254559 | None | -1.558412966 | 5.57E-08 | Lung |
| 4502 | MT2A | -1.558340106 | 1.12E-17 | Lung |
| 348807 | CFAP100 | -1.557766337 | 6.26E-07 | Lung |
| 9839 | ZEB2 | -1.55769238 | 3.64E-36 | Lung |
| 8745 | ADAM23 | -1.556601221 | 5.64E-10 | Lung |
| 6586 | SLIT3 | -1.555326933 | 4.57E-14 | Lung |
| 646658 | SYNDIG1L | -1.55527935 | 1.25E-09 | Lung |
| 84815 | None | -1.554306817 | 6.54E-18 | Lung |
| 72 | ACTG2 | -1.552508558 | 3.91E-13 | Lung |
| 57689 | LRRC4C | -1.551752151 | 6.29E-17 | Lung |
| 9934 | P2RY14 | -1.551362337 | 1.64E-26 | Lung |
| 23166 | STAB1 | -1.550441782 | 1.02E-29 | Lung |
| 642587 | None | -1.550261724 | 2.67E-05 | Lung |
| 11240 | PADI2 | -1.55012258 | 1.42E-14 | Lung |
| 3718 | JAK3 | -1.550104224 | 7.56E-35 | Lung |
| 64167 | ERAP2 | -1.549880794 | 1.92E-09 | Lung |
| 55086 | RADX | -1.548985961 | 2.30E-12 | Lung |
| 606724 | None | -1.548711727 | 5.13E-30 | Lung |
| 50486 | G0S2 | -1.548689495 | 8.81E-13 | Lung |
| 51314 | NME8 | -1.548226409 | 2.69E-21 | Lung |
| 266727 | MDGA1 | -1.5477453 | 2.91E-18 | Lung |
| 4773 | NFATC2 | -1.546752857 | 2.41E-15 | Lung |
| 155185 | AMZ1 | -1.546733472 | 5.75E-14 | Lung |
| 9235 | IL32 | -1.545172891 | 5.70E-23 | Lung |
| 4915 | NTRK2 | -1.543519957 | 7.40E-10 | Lung |
| 84966 | IGSF21 | -1.542638485 | 2.31E-10 | Lung |
| 6375 | XCL1 | -1.542072494 | 5.89E-15 | Lung |
| 11122 | PTPRT | -1.541986208 | 5.39E-05 | Lung |
| 8651 | SOCS1 | -1.541816719 | 2.00E-28 | Lung |
| 9452 | ITM2A | -1.541200225 | 7.64E-23 | Lung |
| 6323 | SCN1A | -1.540735516 | 8.43E-07 | Lung |
| 781 | CACNA2D1 | -1.540542944 | 6.27E-09 | Lung |
| 80832 | APOL4 | -1.540369574 | 2.43E-21 | Lung |
| 6445 | SGCG | -1.54011506 | 2.01E-14 | Lung |
| 79690 | GAL3ST4 | -1.539334597 | 1.61E-28 | Lung |
| 440073 | IQSEC3 | -1.539331711 | 1.55E-15 | Lung |
| 100126784 | None | -1.53891422 | 3.05E-11 | Lung |
| 347454 | SOWAHD | -1.537511213 | 2.73E-23 | Lung |
| 6344 | SCTR | -1.536281397 | 0.00029507 | Lung |
| 375387 | NRROS | -1.535922515 | 6.97E-33 | Lung |
| 9068 | ANGPTL1 | -1.535640492 | 3.23E-16 | Lung |
| 57121 | LPAR5 | -1.534005613 | 3.84E-22 | Lung |
| 9034 | CCRL2 | -1.53345253 | 6.30E-23 | Lung |
| 144347 | RFLNA | -1.532330993 | 1.17E-08 | Lung |
| 88 | ACTN2 | -1.53156007 | 1.19E-11 | Lung |
| 5272 | SERPINB9 | -1.531374102 | 3.72E-27 | Lung |
| 146845 | CFAP52 | -1.530949218 | 3.68E-06 | Lung |
| 89876 | MAATS1 | -1.530542683 | 1.37E-08 | Lung |
| 374467 | None | -1.528276546 | 6.98E-11 | Lung |
| 64506 | CPEB1 | -1.527770764 | 4.19E-14 | Lung |
| 26074 | CFAP61 | -1.526967682 | 3.94E-08 | Lung |
| 285195 | SLC9A9 | -1.525438791 | 5.34E-30 | Lung |
| 89932 | PAPLN | -1.52471718 | 1.09E-22 | Lung |
| 63876 | PKNOX2 | -1.523054691 | 4.28E-15 | Lung |
| 57565 | KLHL14 | -1.522725092 | 4.86E-09 | Lung |
| 219527 | LRRC55 | -1.521422448 | 2.00E-08 | Lung |
| 284266 | SIGLEC15 | -1.519239853 | 3.71E-12 | Lung |
| 5054 | SERPINE1 | -1.518354751 | 4.61E-14 | Lung |
| 56265 | CPXM1 | -1.518093196 | 4.09E-16 | Lung |
| 254173 | TTLL10 | -1.517928032 | 4.55E-08 | Lung |
| 85409 | NKD2 | -1.517927955 | 2.87E-15 | Lung |
| 5055 | SERPINB2 | -1.517437284 | 1.11E-06 | Lung |
| 407977 | TNFSF12-TNFSF13 | -1.517328945 | 4.37E-14 | Lung |
| 3274 | HRH2 | -1.516607316 | 1.25E-13 | Lung |
| 3082 | HGF | -1.516386455 | 2.69E-13 | Lung |
| 391712 | TRIM61 | -1.515339527 | 3.71E-12 | Lung |
| 91543 | RSAD2 | -1.514670241 | 3.08E-17 | Lung |
| 387758 | FIBIN | -1.514327858 | 5.88E-16 | Lung |
| 563 | AZGP1 | -1.514120094 | 0.000361848 | Lung |
| 285830 | None | -1.513824078 | 6.68E-24 | Lung |
| 8038 | ADAM12 | -1.513675416 | 8.75E-10 | Lung |
| 23498 | HAAO | -1.513503633 | 2.63E-29 | Lung |
| 54739 | XAF1 | -1.512941222 | 4.51E-24 | Lung |
| 3036 | HAS1 | -1.512646343 | 6.52E-09 | Lung |
| 2277 | VEGFD | -1.512393326 | 1.35E-05 | Lung |
| 2212 | FCGR2A | -1.511345312 | 5.58E-26 | Lung |
| 80206 | FHOD3 | -1.510099604 | 2.02E-09 | Lung |
| 146183 | OTOA | -1.50926436 | 1.81E-16 | Lung |
| 134 | ADORA1 | -1.509106305 | 4.81E-08 | Lung |
| 3128 | None | -1.508661954 | 2.99E-06 | Lung |
| 30817 | ADGRE2 | -1.508347329 | 2.34E-20 | Lung |
| 1907 | EDN2 | -1.508256623 | 9.15E-07 | Lung |
| 6890 | TAP1 | -1.50730121 | 8.58E-30 | Lung |
| 222487 | ADGRG3 | -1.506981735 | 5.05E-12 | Lung |
| 3671 | ISLR | -1.506428963 | 1.56E-17 | Lung |
| 2323 | FLT3LG | -1.506403966 | 3.24E-35 | Lung |
| 5468 | PPARG | -1.505460646 | 7.78E-11 | Lung |
| 25816 | TNFAIP8 | -1.504670199 | 3.33E-31 | Lung |
| 6262 | RYR2 | -1.504079155 | 2.51E-12 | Lung |
| 10993 | SDS | -1.503951328 | 1.93E-11 | Lung |
| 7074 | TIAM1 | -1.503781911 | 3.22E-18 | Lung |
| 2313 | FLI1 | -1.50363571 | 5.22E-36 | Lung |
| 140469 | MYO3B | -1.503574396 | 2.98E-06 | Lung |
| 124602 | KIF19 | -1.503519329 | 1.75E-06 | Lung |
| 3690 | ITGB3 | -1.503514932 | 2.37E-11 | Lung |
| 2634 | GBP2 | -1.503319187 | 9.89E-29 | Lung |
| 715 | C1R | -1.503032163 | 1.04E-34 | Lung |
| 26872 | STEAP1 | -1.502752684 | 2.52E-10 | Lung |
| 655 | BMP7 | -1.502562079 | 7.51E-06 | Lung |
| 151887 | CCDC80 | -1.502120972 | 3.94E-18 | Lung |
| 80274 | SCUBE1 | -1.502083731 | 1.88E-09 | Lung |
| 2619 | GAS1 | -1.500877197 | 1.01E-15 | Lung |
| 5569 | PKIA | -1.50022144 | 9.34E-12 | Lung |
| 140683 | BPIFA2 | 1.512822566 | 0.000102705 | Lung |
| 7031 | TFF1 | 1.513053063 | 0.005952485 | Lung |
| 375057 | STUM | 1.516312656 | 3.63E-05 | Lung |
| 3767 | KCNJ11 | 1.523748959 | 5.95E-12 | Lung |
| 2243 | FGA | 1.530880559 | 0.005582938 | Lung |
| 132625 | ZFP42 | 1.532579555 | 7.64E-05 | Lung |
| 283869 | NPW | 1.554836933 | 1.76E-05 | Lung |
| 190 | NR0B1 | 1.58509243 | 0.000233392 | Lung |
| 1644 | DDC | 1.588066499 | 0.000470526 | Lung |
| 64757 | Mar-01 | 1.605510125 | 1.70E-15 | Lung |
| 256764 | WDR72 | 1.620084059 | 0.000260601 | Lung |
| 4477 | MSMB | 1.625639969 | 0.002711177 | Lung |
| 64850 | ETNPPL | 1.647499785 | 5.67E-07 | Lung |
| 11189 | CELF3 | 1.663735394 | 1.20E-05 | Lung |
| 388323 | GLTPD2 | 1.672372858 | 1.17E-07 | Lung |
| 80341 | BPIFB2 | 1.679237225 | 0.001015837 | Lung |
| 57524 | CASKIN1 | 1.719047352 | 8.78E-11 | Lung |
| 2027 | ENO3 | 1.730254309 | 2.29E-09 | Lung |
| 1646 | AKR1C2 | 1.7406296 | 0.000934675 | Lung |
| 5053 | PAH | 1.764892584 | 1.28E-05 | Lung |
| 22977 | AKR7A3 | 1.855675946 | 8.76E-07 | Lung |
| 5308 | PITX2 | 1.910564467 | 8.93E-06 | Lung |
| 43849 | KLK12 | 1.912356975 | 0.000180755 | Lung |
| 10814 | CPLX2 | 1.923943432 | 0.000117129 | Lung |
| 259 | AMBP | 1.961519258 | 2.90E-06 | Lung |
| 53828 | FXYD4 | 1.966301847 | 4.28E-08 | Lung |
| 3623 | INHA | 1.97715742 | 1.55E-06 | Lung |
| 56033 | BARX1 | 2.014092217 | 0.000106006 | Lung |
| 429 | ASCL1 | 2.030459785 | 0.000127692 | Lung |
| 2877 | GPX2 | 2.040566913 | 0.000243016 | Lung |
| 84570 | COL25A1 | 2.094496649 | 2.08E-06 | Lung |
| 100190940 | None | 2.183205429 | 5.10E-09 | Lung |
| 145837 | None | 2.234074245 | 1.09E-07 | Lung |
| 90632 | None | 2.25477846 | 6.57E-07 | Lung |
| 8170 | SLC14A2 | 2.261454561 | 2.22E-07 | Lung |
| 796 | CALCA | 2.300981284 | 8.82E-05 | Lung |
| 2244 | FGB | 2.368101204 | 0.000218751 | Lung |
| 2267 | FGL1 | 2.630146852 | 6.37E-07 | Lung |
| 1159 | CKMT1B | -4.977588128 | 1.95E-47 | Pan-kidney |
| 50617 | ATP6V0A4 | -4.751604544 | 4.47E-25 | Pan-kidney |
| 3934 | LCN2 | -4.672823827 | 1.42E-37 | Pan-kidney |
| 548596 | CKMT1A | -4.640867685 | 9.58E-48 | Pan-kidney |
| 27134 | TJP3 | -4.263209599 | 7.08E-50 | Pan-kidney |
| 80157 | CWH43 | -4.244163389 | 1.58E-37 | Pan-kidney |
| 6456 | SH3GL2 | -4.186216972 | 5.06E-43 | Pan-kidney |
| 155006 | TMEM213 | -4.123801683 | 8.39E-20 | Pan-kidney |
| 1188 | CLCNKB | -3.983745249 | 3.56E-25 | Pan-kidney |
| 55753 | OGDHL | -3.961343759 | 1.57E-68 | Pan-kidney |
| 197257 | LDHD | -3.827947583 | 7.35E-90 | Pan-kidney |
| 7113 | TMPRSS2 | -3.798800747 | 4.77E-29 | Pan-kidney |
| 135892 | TRIM50 | -3.790363796 | 3.16E-29 | Pan-kidney |
| 3855 | KRT7 | -3.689863026 | 5.07E-29 | Pan-kidney |
| 158326 | FREM1 | -3.662077009 | 9.43E-44 | Pan-kidney |
| 5121 | PCP4 | -3.627080367 | 1.79E-25 | Pan-kidney |
| 10752 | CHL1 | -3.596704718 | 1.89E-31 | Pan-kidney |
| 1586 | CYP17A1 | -3.588806826 | 6.61E-38 | Pan-kidney |
| 199964 | TMEM61 | -3.586495504 | 3.85E-35 | Pan-kidney |
| 4118 | MAL | -3.511523853 | 2.34E-36 | Pan-kidney |
| 51458 | RHCG | -3.507289221 | 5.29E-13 | Pan-kidney |
| 8796 | SCEL | -3.489467184 | 6.22E-24 | Pan-kidney |
| 266977 | ADGRF1 | -3.487356936 | 1.04E-24 | Pan-kidney |
| 55040 | EPN3 | -3.455105169 | 1.35E-28 | Pan-kidney |
| 57127 | RHBG | -3.450526321 | 2.36E-20 | Pan-kidney |
| 388135 | INSYN1 | -3.432388061 | 1.56E-32 | Pan-kidney |
| 5816 | PVALB | -3.422060255 | 3.86E-14 | Pan-kidney |
| 3816 | KLK1 | -3.402448432 | 6.09E-15 | Pan-kidney |
| 341359 | SYT10 | -3.390249116 | 2.95E-34 | Pan-kidney |
| 2254 | FGF9 | -3.353515947 | 5.39E-22 | Pan-kidney |
| 6549 | SLC9A2 | -3.347708802 | 5.01E-28 | Pan-kidney |
| 84553 | FAXC | -3.318181608 | 4.72E-39 | Pan-kidney |
| 128853 | DUSP15 | -3.279002718 | 1.06E-40 | Pan-kidney |
| 54886 | PLPPR1 | -3.277774633 | 5.39E-34 | Pan-kidney |
| 255189 | PLA2G4F | -3.27033316 | 1.97E-17 | Pan-kidney |
| 151126 | ZNF385B | -3.251574014 | 9.68E-40 | Pan-kidney |
| 54845 | ESRP1 | -3.188612512 | 1.05E-20 | Pan-kidney |
| 27324 | TOX3 | -3.181240069 | 1.41E-34 | Pan-kidney |
| 2299 | FOXI1 | -3.179431601 | 3.95E-13 | Pan-kidney |
| 5067 | CNTN3 | -3.171205018 | 1.97E-30 | Pan-kidney |
| 1080 | CFTR | -3.163923324 | 3.96E-14 | Pan-kidney |
| 163183 | SYNE4 | -3.159007829 | 1.00E-27 | Pan-kidney |
| 27124 | INPP5J | -3.139277398 | 2.84E-27 | Pan-kidney |
| 137970 | UNC5D | -3.135877806 | 5.49E-24 | Pan-kidney |
| 6337 | SCNN1A | -3.102253329 | 4.85E-31 | Pan-kidney |
| 5596 | MAPK4 | -3.061861574 | 1.42E-29 | Pan-kidney |
| 79170 | PRR15L | -3.053289651 | 1.82E-28 | Pan-kidney |
| 79152 | FA2H | -3.012680956 | 5.51E-33 | Pan-kidney |
| 124975 | GGT6 | -2.992905505 | 3.47E-18 | Pan-kidney |
| 29842 | TFCP2L1 | -2.979458566 | 1.51E-28 | Pan-kidney |
| 525 | ATP6V1B1 | -2.972539955 | 2.24E-15 | Pan-kidney |
| 10891 | PPARGC1A | -2.963252436 | 1.38E-55 | Pan-kidney |
| 1152 | CKB | -2.942901706 | 6.91E-64 | Pan-kidney |
| 9622 | KLK4 | -2.923150878 | 1.05E-16 | Pan-kidney |
| 816 | CAMK2B | -2.918350093 | 4.35E-29 | Pan-kidney |
| 5284 | PIGR | -2.908384789 | 2.15E-15 | Pan-kidney |
| 3767 | KCNJ11 | -2.904185161 | 9.11E-53 | Pan-kidney |
| 27255 | CNTN6 | -2.903359076 | 4.48E-16 | Pan-kidney |
| 253012 | HEPACAM2 | -2.897627932 | 7.66E-13 | Pan-kidney |
| 79041 | TMEM38A | -2.893776102 | 1.21E-42 | Pan-kidney |
| 9699 | RIMS2 | -2.886166901 | 3.62E-25 | Pan-kidney |
| 135138 | PACRG | -2.868017011 | 9.09E-32 | Pan-kidney |
| 10117 | ENAM | -2.84981578 | 3.20E-41 | Pan-kidney |
| 9073 | CLDN8 | -2.842937836 | 1.38E-12 | Pan-kidney |
| 8431 | NR0B2 | -2.837844554 | 1.60E-15 | Pan-kidney |
| 2642 | GCGR | -2.833318975 | 6.40E-16 | Pan-kidney |
| 1187 | CLCNKA | -2.82358848 | 5.26E-18 | Pan-kidney |
| 27112 | FAM155B | -2.788754284 | 6.65E-42 | Pan-kidney |
| 1645 | AKR1C1 | -2.760477308 | 1.15E-38 | Pan-kidney |
| 7809 | BSND | -2.753285789 | 1.42E-15 | Pan-kidney |
| 255928 | SYT14 | -2.723536657 | 9.71E-36 | Pan-kidney |
| 84467 | FBN3 | -2.722715994 | 3.99E-15 | Pan-kidney |
| 622 | BDH1 | -2.715861438 | 3.35E-51 | Pan-kidney |
| 84264 | HAGHL | -2.706802819 | 5.71E-31 | Pan-kidney |
| 80763 | SPX | -2.703211436 | 5.58E-32 | Pan-kidney |
| 29114 | TAGLN3 | -2.699756051 | 9.92E-22 | Pan-kidney |
| 5639 | PRRG2 | -2.696803282 | 4.32E-39 | Pan-kidney |
| 6492 | SIM1 | -2.681395379 | 1.92E-25 | Pan-kidney |
| 27344 | PCSK1N | -2.667121212 | 4.60E-21 | Pan-kidney |
| 401250 | MCCD1 | -2.656580911 | 6.14E-20 | Pan-kidney |
| 285489 | DOK7 | -2.653723554 | 4.89E-31 | Pan-kidney |
| 10655 | DMRT2 | -2.641296288 | 1.01E-10 | Pan-kidney |
| 6712 | SPTBN2 | -2.604383961 | 1.07E-20 | Pan-kidney |
| 203859 | ANO5 | -2.600161839 | 2.40E-27 | Pan-kidney |
| 123264 | SLC51B | -2.573436095 | 8.74E-32 | Pan-kidney |
| 9058 | SLC13A2 | -2.566613156 | 4.24E-20 | Pan-kidney |
| 120071 | LARGE2 | -2.515048248 | 9.00E-34 | Pan-kidney |
| 127124 | ATP6V1G3 | -2.512057987 | 5.81E-14 | Pan-kidney |
| 339983 | NAT8L | -2.506150301 | 7.17E-20 | Pan-kidney |
| 2155 | F7 | -2.500420908 | 1.20E-29 | Pan-kidney |
| 22843 | PPM1E | -2.490490182 | 3.16E-33 | Pan-kidney |
| 146336 | None | -2.48870326 | 3.86E-22 | Pan-kidney |
| 25928 | SOSTDC1 | -2.479906586 | 3.05E-12 | Pan-kidney |
| 655 | BMP7 | -2.472332036 | 3.61E-14 | Pan-kidney |
| 4634 | MYL3 | -2.469496699 | 3.09E-15 | Pan-kidney |
| 149466 | C1orf210 | -2.419389238 | 8.28E-29 | Pan-kidney |
| 54566 | EPB41L4B | -2.415968012 | 3.70E-20 | Pan-kidney |
| 27330 | RPS6KA6 | -2.410401581 | 1.83E-27 | Pan-kidney |
| 27231 | NMRK2 | -2.408395494 | 1.99E-14 | Pan-kidney |
| 3177 | SLC29A2 | -2.405478873 | 1.38E-28 | Pan-kidney |
| 1672 | DEFB1 | -2.392743695 | 2.20E-18 | Pan-kidney |
| 5865 | RAB3B | -2.390971349 | 9.73E-28 | Pan-kidney |
| 148252 | DIRAS1 | -2.382598638 | 8.92E-18 | Pan-kidney |
| 643008 | SMIM5 | -2.376379505 | 4.47E-22 | Pan-kidney |
| 55554 | KLK15 | -2.350392436 | 1.56E-15 | Pan-kidney |
| 26287 | ANKRD2 | -2.348199985 | 2.63E-15 | Pan-kidney |
| 10361 | NPM2 | -2.342194493 | 6.18E-37 | Pan-kidney |
| 4935 | GPR143 | -2.335297486 | 1.33E-20 | Pan-kidney |
| 6439 | SFTPB | -2.315903165 | 2.72E-11 | Pan-kidney |
| 1583 | CYP11A1 | -2.309891988 | 6.13E-14 | Pan-kidney |
| 347733 | TUBB2B | -2.304941448 | 3.96E-31 | Pan-kidney |
| 10840 | ALDH1L1 | -2.302737836 | 2.12E-24 | Pan-kidney |
| 5909 | RAP1GAP | -2.297908085 | 1.54E-33 | Pan-kidney |
| 610 | HCN2 | -2.292947527 | 4.69E-13 | Pan-kidney |
| 54716 | SLC6A20 | -2.289395522 | 1.03E-11 | Pan-kidney |
| 85477 | SCIN | -2.287559684 | 1.40E-29 | Pan-kidney |
| 347730 | LRRTM1 | -2.285747525 | 1.49E-14 | Pan-kidney |
| 170690 | ADAMTS16 | -2.272175763 | 1.61E-19 | Pan-kidney |
| 363 | AQP6 | -2.267668738 | 2.86E-12 | Pan-kidney |
| 54836 | BSPRY | -2.265307129 | 9.34E-29 | Pan-kidney |
| 65975 | STK33 | -2.26501785 | 1.27E-24 | Pan-kidney |
| 440503 | PLIN5 | -2.258532143 | 1.42E-27 | Pan-kidney |
| 2302 | FOXJ1 | -2.245490959 | 5.99E-14 | Pan-kidney |
| 644943 | RASSF10 | -2.237951067 | 3.73E-27 | Pan-kidney |
| 9762 | LZTS3 | -2.235891954 | 6.07E-71 | Pan-kidney |
| 9143 | SYNGR3 | -2.2349731 | 5.97E-17 | Pan-kidney |
| 6326 | SCN2A | -2.23327958 | 1.01E-21 | Pan-kidney |
| 1496 | CTNNA2 | -2.230282396 | 2.01E-15 | Pan-kidney |
| 5017 | OVOL1 | -2.225675645 | 4.22E-22 | Pan-kidney |
| 79784 | MYH14 | -2.223452759 | 4.70E-20 | Pan-kidney |
| 354 | KLK3 | -2.19755838 | 4.74E-16 | Pan-kidney |
| 134285 | TMEM171 | -2.197068635 | 1.50E-37 | Pan-kidney |
| 7108 | TM7SF2 | -2.193189845 | 3.49E-60 | Pan-kidney |
| 2104 | ESRRG | -2.188591718 | 3.75E-27 | Pan-kidney |
| 26038 | CHD5 | -2.170218908 | 1.11E-15 | Pan-kidney |
| 10053 | AP1M2 | -2.169662867 | 4.25E-28 | Pan-kidney |
| 23779 | ARHGAP8 | -2.164346615 | 2.13E-40 | Pan-kidney |
| 25859 | None | -2.157683278 | 2.03E-12 | Pan-kidney |
| 3083 | HGFAC | -2.155765959 | 1.20E-14 | Pan-kidney |
| 124872 | B4GALNT2 | -2.154674237 | 6.49E-15 | Pan-kidney |
| 338651 | None | -2.147119153 | 5.18E-30 | Pan-kidney |
| 245972 | ATP6V0D2 | -2.142152501 | 1.08E-06 | Pan-kidney |
| 83875 | BCO2 | -2.139193416 | 1.17E-32 | Pan-kidney |
| 5019 | OXCT1 | -2.122909777 | 1.53E-39 | Pan-kidney |
| 51454 | GULP1 | -2.122525461 | 7.10E-49 | Pan-kidney |
| 119391 | GSTO2 | -2.116129931 | 3.55E-24 | Pan-kidney |
| 9001 | HAP1 | -2.114363067 | 4.14E-24 | Pan-kidney |
| 9229 | DLGAP1 | -2.114059756 | 1.45E-15 | Pan-kidney |
| 136227 | COL26A1 | -2.104311904 | 1.63E-21 | Pan-kidney |
| 388531 | RGS9BP | -2.103186692 | 5.98E-45 | Pan-kidney |
| 55859 | BEX1 | -2.100866958 | 7.99E-15 | Pan-kidney |
| 643763 | None | -2.097675928 | 1.15E-16 | Pan-kidney |
| 199731 | CADM4 | -2.092282669 | 9.43E-36 | Pan-kidney |
| 54894 | RNF43 | -2.092192976 | 6.54E-41 | Pan-kidney |
| 83959 | SLC4A11 | -2.079963899 | 1.79E-23 | Pan-kidney |
| 3769 | KCNJ13 | -2.07413481 | 2.08E-14 | Pan-kidney |
| 55561 | CDC42BPG | -2.071942214 | 1.23E-29 | Pan-kidney |
| 256764 | WDR72 | -2.070404124 | 1.33E-29 | Pan-kidney |
| 84803 | GPAT3 | -2.070129504 | 4.25E-34 | Pan-kidney |
| 4072 | EPCAM | -2.069855825 | 2.75E-36 | Pan-kidney |
| 58495 | OVOL2 | -2.069072668 | 4.82E-24 | Pan-kidney |
| 26027 | ACOT11 | -2.059446698 | 3.09E-47 | Pan-kidney |
| 57111 | RAB25 | -2.058795001 | 7.21E-09 | Pan-kidney |
| 4950 | None | -2.055931401 | 1.15E-35 | Pan-kidney |
| 126695 | KDF1 | -2.040678181 | 3.04E-30 | Pan-kidney |
| 27165 | GLS2 | -2.040272224 | 3.57E-42 | Pan-kidney |
| 7780 | SLC30A2 | -2.038625329 | 2.29E-12 | Pan-kidney |
| 389434 | IYD | -2.037845577 | 7.71E-15 | Pan-kidney |
| 92558 | BICDL1 | -2.037368347 | 1.41E-37 | Pan-kidney |
| 254887 | ZDHHC23 | -2.035636741 | 4.86E-49 | Pan-kidney |
| 79098 | C1orf116 | -2.032893433 | 2.56E-16 | Pan-kidney |
| 375791 | CYSRT1 | -2.032017025 | 2.17E-34 | Pan-kidney |
| 136288 | C7orf57 | -2.027615951 | 1.83E-22 | Pan-kidney |
| 9649 | RALGPS1 | -2.020820367 | 1.64E-52 | Pan-kidney |
| 202374 | STK32A | -2.010941852 | 1.83E-15 | Pan-kidney |
| 200879 | LIPH | -2.009045385 | 2.54E-14 | Pan-kidney |
| 79679 | VTCN1 | -1.998836373 | 1.66E-13 | Pan-kidney |
| 283392 | None | -1.996968107 | 3.76E-14 | Pan-kidney |
| 10382 | TUBB4A | -1.994705555 | 1.43E-23 | Pan-kidney |
| 56956 | LHX9 | -1.980569006 | 5.27E-13 | Pan-kidney |
| 377841 | ENTPD8 | -1.976021206 | 8.18E-20 | Pan-kidney |
| 203190 | LGI3 | -1.971837243 | 4.24E-20 | Pan-kidney |
| 51286 | CEND1 | -1.961715689 | 3.67E-14 | Pan-kidney |
| 136306 | SVOPL | -1.950591698 | 6.42E-15 | Pan-kidney |
| 1364 | CLDN4 | -1.949854351 | 3.63E-37 | Pan-kidney |
| 51090 | PLLP | -1.949117596 | 3.06E-33 | Pan-kidney |
| 38 | ACAT1 | -1.945335112 | 9.15E-62 | Pan-kidney |
| 91862 | MARVELD3 | -1.942246465 | 2.74E-30 | Pan-kidney |
| 28992 | MACROD1 | -1.94145307 | 1.90E-49 | Pan-kidney |
| 153562 | MARVELD2 | -1.93841992 | 6.07E-51 | Pan-kidney |
| 27146 | FAM184B | -1.931330581 | 6.78E-14 | Pan-kidney |
| 9576 | SPAG6 | -1.928740443 | 2.09E-12 | Pan-kidney |
| 4329 | ALDH6A1 | -1.927271501 | 3.94E-44 | Pan-kidney |
| 401431 | None | -1.922453369 | 3.87E-36 | Pan-kidney |
| 1040 | CDS1 | -1.922207019 | 5.87E-61 | Pan-kidney |
| 399948 | COLCA1 | -1.916468718 | 5.33E-27 | Pan-kidney |
| 143098 | MPP7 | -1.910213074 | 1.44E-42 | Pan-kidney |
| 553158 | PRR5-ARHGAP8 | -1.90937793 | 1.15E-26 | Pan-kidney |
| 7164 | TPD52L1 | -1.907399625 | 1.10E-40 | Pan-kidney |
| 3219 | HOXB9 | -1.90369978 | 6.63E-20 | Pan-kidney |
| 9455 | HOMER2 | -1.900357983 | 1.39E-34 | Pan-kidney |
| 10309 | CCNO | -1.900095147 | 1.72E-16 | Pan-kidney |
| 79730 | NSUN7 | -1.899264685 | 2.19E-30 | Pan-kidney |
| 147798 | TMC4 | -1.895979784 | 1.85E-18 | Pan-kidney |
| 147700 | KLC3 | -1.893195439 | 7.49E-22 | Pan-kidney |
| 26232 | FBXO2 | -1.889885951 | 1.97E-21 | Pan-kidney |
| 54848 | ARHGEF38 | -1.889466305 | 2.34E-21 | Pan-kidney |
| 140458 | ASB5 | -1.889037799 | 3.24E-13 | Pan-kidney |
| 400916 | CHCHD10 | -1.88841885 | 2.48E-45 | Pan-kidney |
| 4246 | SCGB2A1 | -1.884911088 | 1.04E-17 | Pan-kidney |
| 4099 | MAG | -1.881317824 | 4.20E-16 | Pan-kidney |
| 1365 | CLDN3 | -1.873895545 | 2.69E-14 | Pan-kidney |
| 796 | CALCA | -1.871936533 | 7.61E-12 | Pan-kidney |
| 23171 | GPD1L | -1.869619167 | 7.31E-53 | Pan-kidney |
| 78997 | GDAP1L1 | -1.86484255 | 2.72E-21 | Pan-kidney |
| 22798 | LAMB4 | -1.864339783 | 3.68E-16 | Pan-kidney |
| 155368 | METTL27 | -1.862402573 | 2.30E-26 | Pan-kidney |
| 2743 | GLRB | -1.855299999 | 9.54E-14 | Pan-kidney |
| 286676 | ILDR1 | -1.85141879 | 3.40E-32 | Pan-kidney |
| 7137 | None | -1.851159083 | 1.44E-16 | Pan-kidney |
| 29106 | SCG3 | -1.848515329 | 3.76E-10 | Pan-kidney |
| 1114 | CHGB | -1.84714425 | 2.72E-13 | Pan-kidney |
| 6768 | ST14 | -1.837061349 | 2.88E-25 | Pan-kidney |
| 127731 | VWA5B1 | -1.831817152 | 1.33E-11 | Pan-kidney |
| 389493 | NUPR2 | -1.826674484 | 1.25E-12 | Pan-kidney |
| 5652 | PRSS8 | -1.82470239 | 1.56E-25 | Pan-kidney |
| 113828 | FAM83F | -1.824517573 | 9.01E-09 | Pan-kidney |
| 122416 | ANKRD9 | -1.818591408 | 3.61E-34 | Pan-kidney |
| 30818 | KCNIP3 | -1.818321606 | 2.22E-23 | Pan-kidney |
| 57821 | CCDC181 | -1.817521149 | 2.70E-15 | Pan-kidney |
| 56934 | CA10 | -1.811019728 | 5.04E-11 | Pan-kidney |
| 143162 | FRMPD2 | -1.804292587 | 5.01E-24 | Pan-kidney |
| 23530 | NNT | -1.803352697 | 5.78E-56 | Pan-kidney |
| 10610 | ST6GALNAC2 | -1.796933392 | 1.42E-25 | Pan-kidney |
| 54756 | IL17RD | -1.796589323 | 3.66E-32 | Pan-kidney |
| 1773 | DNASE1 | -1.796226921 | 5.31E-17 | Pan-kidney |
| 23624 | CBLC | -1.790044925 | 6.48E-14 | Pan-kidney |
| 401474 | SAMD12 | -1.787646876 | 1.12E-33 | Pan-kidney |
| 378108 | STAG3L3 | -1.787092653 | 1.40E-22 | Pan-kidney |
| 83697 | SLC4A9 | -1.785585517 | 4.58E-10 | Pan-kidney |
| 8395 | PIP5K1B | -1.781048499 | 1.03E-27 | Pan-kidney |
| 414332 | LCN10 | -1.780278536 | 3.59E-13 | Pan-kidney |
| 644809 | C15orf56 | -1.776895144 | 5.40E-17 | Pan-kidney |
| 6755 | SSTR5 | -1.770741781 | 4.49E-17 | Pan-kidney |
| 306 | ANXA3 | -1.769759957 | 2.71E-15 | Pan-kidney |
| 3993 | LLGL2 | -1.769166476 | 2.23E-56 | Pan-kidney |
| 166348 | KBTBD12 | -1.767939469 | 7.85E-10 | Pan-kidney |
| 399823 | FOXI2 | -1.76645105 | 7.82E-08 | Pan-kidney |
| 134548 | SOWAHA | -1.765018895 | 1.11E-14 | Pan-kidney |
| 222008 | VSTM2A | -1.763787407 | 2.79E-10 | Pan-kidney |
| 124093 | CCDC78 | -1.758341895 | 8.67E-17 | Pan-kidney |
| 115111 | SLC26A7 | -1.755422677 | 1.43E-06 | Pan-kidney |
| 114786 | XKR4 | -1.753447868 | 8.16E-18 | Pan-kidney |
| 196051 | PLPP4 | -1.75310391 | 1.78E-15 | Pan-kidney |
| 1128 | CHRM1 | -1.741576181 | 1.25E-13 | Pan-kidney |
| 23242 | COBL | -1.741351347 | 6.11E-24 | Pan-kidney |
| 222171 | PRR15 | -1.740797293 | 1.37E-18 | Pan-kidney |
| 1950 | EGF | -1.740734643 | 8.01E-12 | Pan-kidney |
| 400961 | PAIP2B | -1.739620251 | 5.95E-36 | Pan-kidney |
| 28999 | KLF15 | -1.732584876 | 3.00E-28 | Pan-kidney |
| 10647 | SCGB1D2 | -1.724984264 | 1.73E-17 | Pan-kidney |
| 113655 | MFSD3 | -1.723675208 | 8.48E-50 | Pan-kidney |
| 8825 | LIN7A | -1.719637177 | 2.25E-19 | Pan-kidney |
| 79935 | CNTD2 | -1.718906546 | 1.58E-17 | Pan-kidney |
| 5208 | PFKFB2 | -1.714630488 | 1.02E-37 | Pan-kidney |
| 51804 | SIX4 | -1.712562175 | 1.92E-15 | Pan-kidney |
| 254050 | LRRC43 | -1.709709311 | 1.33E-17 | Pan-kidney |
| 5304 | PIP | -1.709684121 | 2.80E-11 | Pan-kidney |
| 379 | ARL4D | -1.708811953 | 2.44E-26 | Pan-kidney |
| 83543 | AIF1L | -1.707723101 | 1.88E-18 | Pan-kidney |
| 58473 | PLEKHB1 | -1.699314008 | 9.00E-22 | Pan-kidney |
| 57830 | KRTAP5-8 | -1.698881425 | 3.10E-09 | Pan-kidney |
| 10202 | DHRS2 | -1.697659595 | 1.25E-06 | Pan-kidney |
| 57467 | HHATL | -1.696797956 | 3.33E-08 | Pan-kidney |
| 100240734 | None | -1.695637847 | 1.19E-27 | Pan-kidney |
| 100131213 | None | -1.695004684 | 9.07E-56 | Pan-kidney |
| 643224 | None | -1.694976507 | 1.08E-13 | Pan-kidney |
| 112703 | FAM71E1 | -1.693344555 | 1.70E-31 | Pan-kidney |
| 10991 | SLC38A3 | -1.692426197 | 2.82E-10 | Pan-kidney |
| 7069 | THRSP | -1.68389384 | 1.60E-08 | Pan-kidney |
| 10644 | IGF2BP2 | -1.683163924 | 5.27E-13 | Pan-kidney |
| 25837 | RAB26 | -1.67861909 | 3.22E-20 | Pan-kidney |
| 1014 | CDH16 | -1.677849197 | 1.74E-18 | Pan-kidney |
| 2805 | GOT1 | -1.673563487 | 8.09E-70 | Pan-kidney |
| 51161 | C3orf18 | -1.670456804 | 6.06E-42 | Pan-kidney |
| 254956 | MORN5 | -1.668175702 | 1.27E-14 | Pan-kidney |
| 3664 | IRF6 | -1.666353168 | 4.63E-16 | Pan-kidney |
| 154664 | ABCA13 | -1.664999832 | 1.36E-10 | Pan-kidney |
| 10110 | SGK2 | -1.66202962 | 2.47E-33 | Pan-kidney |
| 92815 | HIST3H2A | -1.654264205 | 1.90E-20 | Pan-kidney |
| 57460 | PPM1H | -1.653847556 | 1.88E-43 | Pan-kidney |
| 1791 | DNTT | -1.652080159 | 4.45E-17 | Pan-kidney |
| 266722 | HS6ST3 | -1.650833944 | 9.47E-07 | Pan-kidney |
| 57475 | PLEKHH1 | -1.650369265 | 2.09E-43 | Pan-kidney |
| 30812 | SOX8 | -1.648355229 | 3.17E-13 | Pan-kidney |
| 7384 | UQCRC1 | -1.645626367 | 1.29E-68 | Pan-kidney |
| 2653 | GCSH | -1.645206907 | 1.03E-33 | Pan-kidney |
| 196047 | None | -1.644213707 | 6.84E-20 | Pan-kidney |
| 116449 | CLNK | -1.643793921 | 3.51E-07 | Pan-kidney |
| 5774 | PTPN3 | -1.642652569 | 1.84E-55 | Pan-kidney |
| 79885 | HDAC11 | -1.640528707 | 2.31E-68 | Pan-kidney |
| 375057 | STUM | -1.639459576 | 6.71E-11 | Pan-kidney |
| 57619 | SHROOM3 | -1.636789609 | 6.90E-28 | Pan-kidney |
| 10243 | GPHN | -1.636401875 | 1.11E-62 | Pan-kidney |
| 4902 | NRTN | -1.635560222 | 2.95E-20 | Pan-kidney |
| 57017 | COQ9 | -1.632403794 | 5.83E-72 | Pan-kidney |
| 114783 | None | -1.632052779 | 4.26E-15 | Pan-kidney |
| 79007 | DBNDD1 | -1.630696177 | 3.01E-32 | Pan-kidney |
| 10913 | EDAR | -1.630691315 | 4.52E-10 | Pan-kidney |
| 27098 | CLUL1 | -1.629595178 | 1.42E-21 | Pan-kidney |
| 146802 | SLC47A2 | -1.628556475 | 7.85E-07 | Pan-kidney |
| 400224 | PLEKHD1 | -1.627494186 | 9.81E-11 | Pan-kidney |
| 27199 | OXGR1 | -1.625108711 | 2.17E-08 | Pan-kidney |
| 1917 | EEF1A2 | -1.61925187 | 1.11E-06 | Pan-kidney |
| 254295 | PHYHD1 | -1.618001173 | 6.36E-12 | Pan-kidney |
| 115948 | CCDC151 | -1.617877217 | 6.87E-16 | Pan-kidney |
| 6521 | SLC4A1 | -1.617170323 | 0.000244821 | Pan-kidney |
| 760 | CA2 | -1.617136442 | 2.58E-23 | Pan-kidney |
| 25924 | MYRIP | -1.61447561 | 2.75E-12 | Pan-kidney |
| 389015 | SLC9A4 | -1.613212486 | 2.49E-09 | Pan-kidney |
| 9542 | NRG2 | -1.611350876 | 1.49E-18 | Pan-kidney |
| 1286 | COL4A4 | -1.609148716 | 5.44E-30 | Pan-kidney |
| 222545 | GPRC6A | -1.608992586 | 6.94E-10 | Pan-kidney |
| 4645 | MYO5B | -1.60841212 | 6.77E-46 | Pan-kidney |
| 84229 | DRC7 | -1.608284883 | 1.45E-08 | Pan-kidney |
| 388722 | C1orf53 | -1.606719749 | 5.53E-27 | Pan-kidney |
| 2886 | GRB7 | -1.60163937 | 1.58E-34 | Pan-kidney |
| 80737 | VWA7 | -1.601406695 | 2.80E-30 | Pan-kidney |
| 25841 | ABTB2 | -1.60054737 | 1.41E-33 | Pan-kidney |
| 152206 | CCDC13 | -1.59789943 | 7.68E-26 | Pan-kidney |
| 126820 | WDR63 | -1.593273278 | 1.82E-23 | Pan-kidney |
| 345079 | SOWAHB | -1.59048108 | 4.95E-22 | Pan-kidney |
| 57530 | CGN | -1.590203925 | 6.47E-22 | Pan-kidney |
| 168002 | DACT2 | -1.589115269 | 2.22E-07 | Pan-kidney |
| 284252 | KCTD1 | -1.58091579 | 1.25E-46 | Pan-kidney |
| 284100 | None | -1.579144196 | 1.04E-18 | Pan-kidney |
| 130576 | LYPD6B | -1.572744137 | 7.81E-10 | Pan-kidney |
| 23544 | SEZ6L | -1.571467064 | 1.70E-10 | Pan-kidney |
| 84688 | C9orf24 | -1.571171314 | 3.55E-13 | Pan-kidney |
| 646 | BNC1 | -1.56977242 | 5.93E-09 | Pan-kidney |
| 3149 | HMGB3 | -1.569379551 | 1.73E-44 | Pan-kidney |
| 9099 | USP2 | -1.569264494 | 8.26E-22 | Pan-kidney |
| 8739 | HRK | -1.567138848 | 7.87E-17 | Pan-kidney |
| 150590 | C2orf15 | -1.563784119 | 7.36E-45 | Pan-kidney |
| 5672 | PSG4 | -1.561783794 | 2.74E-10 | Pan-kidney |
| 10966 | RAB40B | -1.555441822 | 2.74E-52 | Pan-kidney |
| 776 | CACNA1D | -1.553534973 | 1.21E-20 | Pan-kidney |
| 590 | BCHE | -1.553504367 | 6.05E-07 | Pan-kidney |
| 116238 | TLCD1 | -1.550281627 | 8.57E-33 | Pan-kidney |
| 60680 | CELF5 | -1.549138211 | 5.19E-09 | Pan-kidney |
| 83473 | KATNAL2 | -1.546984464 | 1.35E-24 | Pan-kidney |
| 3218 | HOXB8 | -1.541504157 | 1.18E-18 | Pan-kidney |
| 7276 | TTR | -1.539316461 | 1.81E-07 | Pan-kidney |
| 84651 | SPINK7 | -1.538476973 | 1.86E-13 | Pan-kidney |
| 2021 | ENDOG | -1.538020081 | 1.22E-44 | Pan-kidney |
| 1632 | ECI1 | -1.535154351 | 3.00E-53 | Pan-kidney |
| 55605 | KIF21A | -1.532233723 | 2.00E-33 | Pan-kidney |
| 5745 | PTH1R | -1.530193071 | 6.20E-09 | Pan-kidney |
| 137872 | ADHFE1 | -1.530136475 | 9.87E-39 | Pan-kidney |
| 4045 | LSAMP | -1.52904114 | 2.20E-09 | Pan-kidney |
| 51181 | DCXR | -1.527469466 | 4.99E-39 | Pan-kidney |
| 401052 | None | -1.524624174 | 3.46E-35 | Pan-kidney |
| 9515 | STXBP5L | -1.522289301 | 3.78E-10 | Pan-kidney |
| 155066 | ATP6V0E2 | -1.519687373 | 6.98E-44 | Pan-kidney |
| 638 | BIK | -1.51717507 | 8.01E-16 | Pan-kidney |
| 1795 | DOCK3 | -1.516925848 | 6.04E-13 | Pan-kidney |
| 5095 | PCCA | -1.516866713 | 3.19E-60 | Pan-kidney |
| 5264 | PHYH | -1.516757824 | 1.52E-56 | Pan-kidney |
| 79608 | RIC3 | -1.513755959 | 4.85E-11 | Pan-kidney |
| 387914 | SHISA2 | -1.513503214 | 3.24E-12 | Pan-kidney |
| 440556 | None | -1.509421534 | 1.28E-06 | Pan-kidney |
| 113230 | MISP3 | -1.509345686 | 3.10E-35 | Pan-kidney |
| 83451 | ABHD11 | -1.508954817 | 2.40E-43 | Pan-kidney |
| 153328 | SLC25A48 | -1.50817703 | 1.06E-10 | Pan-kidney |
| 2118 | ETV4 | -1.507774495 | 4.57E-11 | Pan-kidney |
| 5833 | PCYT2 | -1.507492674 | 2.16E-48 | Pan-kidney |
| 3613 | IMPA2 | -1.506240246 | 5.83E-50 | Pan-kidney |
| 92359 | CRB3 | -1.504839132 | 1.21E-37 | Pan-kidney |
| 8715 | NOL4 | -1.502297362 | 9.06E-09 | Pan-kidney |
| 283238 | SLC22A24 | -1.500723253 | 4.91E-15 | Pan-kidney |
| 116496 | FAM129A | 1.500006411 | 1.77E-39 | Pan-kidney |
| 10633 | RASL10A | 1.500818771 | 3.82E-27 | Pan-kidney |
| 4267 | CD99 | 1.500842849 | 9.43E-45 | Pan-kidney |
| 81563 | C1orf21 | 1.501044238 | 5.27E-31 | Pan-kidney |
| 23432 | GPR161 | 1.501065217 | 7.26E-36 | Pan-kidney |
| 3714 | JAG2 | 1.501134784 | 3.26E-41 | Pan-kidney |
| 3139 | None | 1.501508265 | 1.57E-38 | Pan-kidney |
| 91683 | SYT12 | 1.501925048 | 2.19E-09 | Pan-kidney |
| 3209 | HOXA13 | 1.502080297 | 1.76E-11 | Pan-kidney |
| 7424 | VEGFC | 1.50230669 | 2.66E-31 | Pan-kidney |
| 4776 | NFATC4 | 1.503669285 | 3.32E-28 | Pan-kidney |
| 55013 | MCUB | 1.504276579 | 6.57E-38 | Pan-kidney |
| 653786 | None | 1.505219196 | 2.93E-19 | Pan-kidney |
| 88 | ACTN2 | 1.507015611 | 1.89E-13 | Pan-kidney |
| 51555 | PEX5L | 1.507223698 | 7.54E-19 | Pan-kidney |
| 5540 | NPY4R | 1.507364196 | 1.52E-18 | Pan-kidney |
| 3479 | IGF1 | 1.508079073 | 2.29E-15 | Pan-kidney |
| 6793 | STK10 | 1.509566962 | 3.46E-91 | Pan-kidney |
| 80129 | CCDC170 | 1.510045865 | 2.43E-21 | Pan-kidney |
| 2012 | EMP1 | 1.510413554 | 3.17E-31 | Pan-kidney |
| 6563 | SLC14A1 | 1.510503553 | 5.59E-16 | Pan-kidney |
| 340205 | TREML1 | 1.511132841 | 7.39E-31 | Pan-kidney |
| 9636 | ISG15 | 1.512879187 | 8.07E-36 | Pan-kidney |
| 167359 | NIM1K | 1.513026404 | 1.90E-22 | Pan-kidney |
| 23025 | UNC13A | 1.513172626 | 5.93E-16 | Pan-kidney |
| 80329 | ULBP1 | 1.513610966 | 1.63E-23 | Pan-kidney |
| 7056 | THBD | 1.51443492 | 5.85E-38 | Pan-kidney |
| 441869 | ANKRD65 | 1.514515269 | 7.07E-21 | Pan-kidney |
| 9466 | IL27RA | 1.514859843 | 2.15E-41 | Pan-kidney |
| 5125 | PCSK5 | 1.515088624 | 4.53E-19 | Pan-kidney |
| 64151 | NCAPG | 1.515104164 | 1.72E-30 | Pan-kidney |
| 64061 | TSPYL2 | 1.515384572 | 1.49E-49 | Pan-kidney |
| 1062 | CENPE | 1.515393911 | 2.22E-31 | Pan-kidney |
| 8578 | SCARF1 | 1.51549343 | 3.29E-41 | Pan-kidney |
| 1326 | MAP3K8 | 1.51582814 | 6.12E-60 | Pan-kidney |
| 23251 | MINAR1 | 1.515952841 | 6.49E-32 | Pan-kidney |
| 7161 | TP73 | 1.516603606 | 2.69E-20 | Pan-kidney |
| 56667 | MUC13 | 1.517346743 | 2.15E-08 | Pan-kidney |
| 8424 | BBOX1 | 1.517678869 | 1.77E-05 | Pan-kidney |
| 348158 | ACSM2B | 1.518072729 | 8.34E-05 | Pan-kidney |
| 285598 | ARL10 | 1.51919174 | 2.28E-29 | Pan-kidney |
| 157773 | C8orf48 | 1.519703355 | 3.34E-23 | Pan-kidney |
| 57491 | AHRR | 1.520004245 | 1.06E-27 | Pan-kidney |
| 7448 | VTN | 1.520102705 | 1.58E-10 | Pan-kidney |
| 6038 | RNASE4 | 1.520729366 | 4.52E-17 | Pan-kidney |
| 84502 | JPH4 | 1.521041017 | 1.35E-34 | Pan-kidney |
| 22887 | FOXJ3 | 1.523507522 | 6.95E-48 | Pan-kidney |
| 9437 | NCR1 | 1.52351188 | 2.65E-35 | Pan-kidney |
| 1588 | CYP19A1 | 1.523789628 | 4.35E-22 | Pan-kidney |
| 6582 | SLC22A2 | 1.524166236 | 0.000115844 | Pan-kidney |
| 3592 | IL12A | 1.52495587 | 1.32E-36 | Pan-kidney |
| 2534 | FYN | 1.52520711 | 3.51E-43 | Pan-kidney |
| 56106 | PCDHGA10 | 1.525479241 | 1.08E-15 | Pan-kidney |
| 2157 | F8 | 1.525584379 | 4.74E-32 | Pan-kidney |
| 84848 | None | 1.525601338 | 6.20E-18 | Pan-kidney |
| 147699 | PPM1N | 1.526433358 | 9.52E-31 | Pan-kidney |
| 347454 | SOWAHD | 1.526801077 | 8.80E-33 | Pan-kidney |
| 116441 | TM4SF18 | 1.527160871 | 9.11E-25 | Pan-kidney |
| 6368 | CCL23 | 1.527217841 | 1.74E-31 | Pan-kidney |
| 22822 | PHLDA1 | 1.527721706 | 1.37E-23 | Pan-kidney |
| 80301 | PLEKHO2 | 1.527802953 | 8.98E-80 | Pan-kidney |
| 146779 | EFCAB3 | 1.528068691 | 6.83E-32 | Pan-kidney |
| 6398 | SECTM1 | 1.528122016 | 1.07E-19 | Pan-kidney |
| 84709 | MGARP | 1.529954113 | 1.90E-11 | Pan-kidney |
| 10791 | VAMP5 | 1.530299405 | 5.25E-51 | Pan-kidney |
| 93109 | TMEM44 | 1.530603302 | 2.32E-34 | Pan-kidney |
| 26052 | DNM3 | 1.530610349 | 1.73E-34 | Pan-kidney |
| 5137 | PDE1C | 1.531714009 | 3.45E-08 | Pan-kidney |
| 7122 | CLDN5 | 1.532109318 | 9.26E-32 | Pan-kidney |
| 8847 | None | 1.533655516 | 1.68E-48 | Pan-kidney |
| 10410 | IFITM3 | 1.534917606 | 2.64E-51 | Pan-kidney |
| 119587 | CPXM2 | 1.535284335 | 1.59E-08 | Pan-kidney |
| 55315 | SLC29A3 | 1.535659763 | 5.86E-42 | Pan-kidney |
| 2242 | FES | 1.535828008 | 1.79E-47 | Pan-kidney |
| 503538 | None | 1.537140509 | 1.55E-40 | Pan-kidney |
| 286122 | None | 1.537594371 | 2.58E-16 | Pan-kidney |
| 338557 | FFAR4 | 1.537957239 | 9.93E-21 | Pan-kidney |
| 28959 | TMEM176B | 1.538274086 | 8.55E-11 | Pan-kidney |
| 221336 | BEND6 | 1.538360408 | 3.60E-16 | Pan-kidney |
| 285512 | None | 1.538764908 | 3.41E-31 | Pan-kidney |
| 84733 | CBX2 | 1.538825871 | 6.21E-35 | Pan-kidney |
| 150221 | RIMBP3C | 1.539445013 | 2.79E-43 | Pan-kidney |
| 56126 | PCDHB10 | 1.539448369 | 4.11E-28 | Pan-kidney |
| 55691 | FRMD4A | 1.540215847 | 1.53E-44 | Pan-kidney |
| 909 | CD1A | 1.540455731 | 2.73E-18 | Pan-kidney |
| 641 | BLM | 1.541008042 | 3.89E-50 | Pan-kidney |
| 79191 | IRX3 | 1.541143856 | 1.97E-11 | Pan-kidney |
| 55510 | DDX43 | 1.541652363 | 9.75E-10 | Pan-kidney |
| 23023 | TMCC1 | 1.541974907 | 5.02E-61 | Pan-kidney |
| 51702 | PADI3 | 1.542082852 | 2.92E-10 | Pan-kidney |
| 57162 | PELI1 | 1.542338096 | 1.08E-58 | Pan-kidney |
| 100302736 | TMED7-TICAM2 | 1.543188387 | 4.95E-19 | Pan-kidney |
| 25992 | SNED1 | 1.544054462 | 6.92E-38 | Pan-kidney |
| 286554 | None | 1.544915341 | 2.83E-35 | Pan-kidney |
| 100270710 | None | 1.54499718 | 6.72E-24 | Pan-kidney |
| 3726 | JUNB | 1.546486644 | 2.50E-39 | Pan-kidney |
| 168620 | BHLHA15 | 1.547854118 | 1.15E-21 | Pan-kidney |
| 22821 | RASA3 | 1.548863898 | 5.93E-39 | Pan-kidney |
| 338596 | ST8SIA6 | 1.54950312 | 1.57E-10 | Pan-kidney |
| 9881 | TRANK1 | 1.549805779 | 2.25E-55 | Pan-kidney |
| 5627 | PROS1 | 1.550329657 | 1.94E-21 | Pan-kidney |
| 10888 | GPR83 | 1.550378093 | 1.00E-25 | Pan-kidney |
| 389634 | None | 1.550718049 | 7.26E-24 | Pan-kidney |
| 9963 | SLC23A1 | 1.550781325 | 1.98E-08 | Pan-kidney |
| 81930 | KIF18A | 1.551245185 | 5.36E-32 | Pan-kidney |
| 30845 | EHD3 | 1.551592423 | 3.50E-39 | Pan-kidney |
| 152273 | FGD5 | 1.551747259 | 5.59E-28 | Pan-kidney |
| 4071 | TM4SF1 | 1.551760805 | 1.37E-36 | Pan-kidney |
| 6572 | SLC18A3 | 1.552324456 | 2.27E-10 | Pan-kidney |
| 153579 | BTNL9 | 1.552637127 | 4.57E-15 | Pan-kidney |
| 4744 | NEFH | 1.553522838 | 2.49E-29 | Pan-kidney |
| 57165 | GJC2 | 1.553877153 | 3.77E-21 | Pan-kidney |
| 79890 | RIN3 | 1.554745651 | 1.79E-53 | Pan-kidney |
| 100132215 | None | 1.554861718 | 3.83E-26 | Pan-kidney |
| 202134 | FAM153B | 1.555291473 | 1.20E-12 | Pan-kidney |
| 5168 | ENPP2 | 1.555526037 | 3.57E-22 | Pan-kidney |
| 3134 | HLA-F | 1.556553438 | 8.08E-48 | Pan-kidney |
| 445347 | TRGC1 | 1.557977087 | 1.88E-11 | Pan-kidney |
| 1296 | COL8A2 | 1.560655693 | 1.77E-20 | Pan-kidney |
| 4321 | MMP12 | 1.561802616 | 2.11E-11 | Pan-kidney |
| 50651 | SLC45A1 | 1.562533874 | 2.23E-41 | Pan-kidney |
| 55196 | RESF1 | 1.562692359 | 1.50E-76 | Pan-kidney |
| 9956 | HS3ST2 | 1.564751864 | 3.51E-11 | Pan-kidney |
| 94015 | TTYH2 | 1.565043266 | 5.60E-54 | Pan-kidney |
| 25975 | EGFL6 | 1.565089742 | 2.21E-11 | Pan-kidney |
| 22895 | RPH3A | 1.565714223 | 1.54E-26 | Pan-kidney |
| 57562 | CEP126 | 1.566318798 | 2.37E-27 | Pan-kidney |
| 56121 | PCDHB15 | 1.566939247 | 1.05E-22 | Pan-kidney |
| 347902 | AMIGO2 | 1.567026731 | 3.03E-19 | Pan-kidney |
| 29108 | PYCARD | 1.568565722 | 8.19E-31 | Pan-kidney |
| 2814 | GP5 | 1.568652147 | 1.13E-35 | Pan-kidney |
| 158471 | PRUNE2 | 1.56933729 | 4.04E-09 | Pan-kidney |
| 7079 | TIMP4 | 1.569486983 | 3.29E-20 | Pan-kidney |
| 7099 | TLR4 | 1.569678069 | 1.81E-42 | Pan-kidney |
| 1524 | CX3CR1 | 1.569810519 | 1.02E-21 | Pan-kidney |
| 11037 | STON1 | 1.569867086 | 1.03E-33 | Pan-kidney |
| 8829 | NRP1 | 1.571061572 | 9.68E-48 | Pan-kidney |
| 5352 | PLOD2 | 1.571272239 | 8.17E-43 | Pan-kidney |
| 4914 | NTRK1 | 1.571741003 | 2.89E-25 | Pan-kidney |
| 167838 | TXLNB | 1.571907528 | 6.25E-20 | Pan-kidney |
| 3371 | TNC | 1.572255227 | 3.13E-15 | Pan-kidney |
| 283349 | RASSF3 | 1.572367899 | 1.44E-32 | Pan-kidney |
| 874 | CBR3 | 1.573324093 | 1.66E-24 | Pan-kidney |
| 57507 | ZNF608 | 1.574723713 | 7.99E-25 | Pan-kidney |
| 6690 | SPINK1 | 1.57513783 | 2.72E-06 | Pan-kidney |
| 5163 | PDK1 | 1.575932277 | 1.43E-46 | Pan-kidney |
| 85028 | None | 1.5761776 | 7.30E-36 | Pan-kidney |
| 342510 | CD300E | 1.576454382 | 1.28E-26 | Pan-kidney |
| 389289 | ANXA2R | 1.577144399 | 1.40E-45 | Pan-kidney |
| 28514 | DLL1 | 1.577671157 | 1.18E-15 | Pan-kidney |
| 90952 | ESAM | 1.577904066 | 3.39E-32 | Pan-kidney |
| 79987 | SVEP1 | 1.578171683 | 1.00E-11 | Pan-kidney |
| 338339 | CLEC4D | 1.578551288 | 1.15E-31 | Pan-kidney |
| 64856 | VWA1 | 1.581258807 | 2.89E-43 | Pan-kidney |
| 25849 | PARM1 | 1.581342424 | 1.27E-19 | Pan-kidney |
| 10736 | SIX2 | 1.583774113 | 6.49E-15 | Pan-kidney |
| 339488 | TFAP2E | 1.584055473 | 4.96E-43 | Pan-kidney |
| 54518 | APBB1IP | 1.584123803 | 8.41E-16 | Pan-kidney |
| 131583 | FAM43A | 1.587017164 | 3.69E-35 | Pan-kidney |
| 56963 | RGMA | 1.587475998 | 3.99E-24 | Pan-kidney |
| 27185 | DISC1 | 1.587666918 | 8.12E-59 | Pan-kidney |
| 1030 | CDKN2B | 1.587716407 | 3.38E-39 | Pan-kidney |
| 53405 | CLIC5 | 1.589111599 | 8.02E-21 | Pan-kidney |
| 148979 | GLIS1 | 1.589176662 | 6.15E-09 | Pan-kidney |
| 339105 | PRSS53 | 1.590000097 | 1.25E-37 | Pan-kidney |
| 3170 | FOXA2 | 1.590375508 | 7.49E-14 | Pan-kidney |
| 50846 | DHH | 1.590761154 | 2.24E-27 | Pan-kidney |
| 2019 | EN1 | 1.591011231 | 5.92E-16 | Pan-kidney |
| 151295 | SLC23A3 | 1.591120377 | 7.68E-10 | Pan-kidney |
| 11118 | BTN3A2 | 1.591247261 | 1.39E-58 | Pan-kidney |
| 100133205 | None | 1.591449262 | 3.90E-25 | Pan-kidney |
| 4211 | MEIS1 | 1.591512848 | 1.62E-23 | Pan-kidney |
| 1958 | EGR1 | 1.592367532 | 1.14E-19 | Pan-kidney |
| 9232 | PTTG1 | 1.59247453 | 3.28E-33 | Pan-kidney |
| 81544 | GDPD5 | 1.593571692 | 6.95E-44 | Pan-kidney |
| 10855 | HPSE | 1.593807268 | 1.25E-19 | Pan-kidney |
| 140606 | SELENOM | 1.593839536 | 8.32E-22 | Pan-kidney |
| 286467 | None | 1.594369028 | 2.97E-24 | Pan-kidney |
| 162517 | FBXO39 | 1.595258188 | 1.19E-32 | Pan-kidney |
| 28951 | TRIB2 | 1.595331556 | 2.59E-42 | Pan-kidney |
| 84953 | MICAL2 | 1.595373593 | 1.71E-36 | Pan-kidney |
| 7124 | TNF | 1.596619201 | 1.16E-21 | Pan-kidney |
| 197358 | NLRC3 | 1.596708286 | 5.35E-68 | Pan-kidney |
| 89870 | TRIM15 | 1.597598943 | 1.60E-08 | Pan-kidney |
| 3745 | KCNB1 | 1.597800488 | 3.88E-16 | Pan-kidney |
| 91662 | NLRP12 | 1.597863338 | 3.76E-30 | Pan-kidney |
| 3812 | KIR3DL2 | 1.598596893 | 9.95E-34 | Pan-kidney |
| 7078 | TIMP3 | 1.598733429 | 9.87E-22 | Pan-kidney |
| 54443 | ANLN | 1.598766862 | 5.10E-28 | Pan-kidney |
| 10797 | MTHFD2 | 1.599055046 | 5.14E-41 | Pan-kidney |
| 7100 | TLR5 | 1.599193108 | 3.22E-50 | Pan-kidney |
| 79370 | BCL2L14 | 1.599319083 | 2.62E-32 | Pan-kidney |
| 84734 | FAM167B | 1.599598464 | 1.63E-38 | Pan-kidney |
| 79901 | CYBRD1 | 1.600320116 | 4.81E-28 | Pan-kidney |
| 10544 | PROCR | 1.601254714 | 7.12E-40 | Pan-kidney |
| 9644 | SH3PXD2A | 1.602070004 | 3.64E-55 | Pan-kidney |
| 283314 | None | 1.602675087 | 1.51E-21 | Pan-kidney |
| 375298 | CERKL | 1.602676515 | 9.62E-30 | Pan-kidney |
| 619373 | MBOAT4 | 1.603110809 | 5.04E-41 | Pan-kidney |
| 11119 | BTN3A1 | 1.604561123 | 3.98E-73 | Pan-kidney |
| 343450 | KCNT2 | 1.605043576 | 8.46E-10 | Pan-kidney |
| 221188 | ADGRG5 | 1.606385873 | 3.96E-29 | Pan-kidney |
| 6271 | S100A1 | 1.606492028 | 1.76E-09 | Pan-kidney |
| 8996 | None | 1.60683067 | 4.43E-33 | Pan-kidney |
| 595 | CCND1 | 1.607907516 | 1.44E-31 | Pan-kidney |
| 6935 | ZEB1 | 1.607941919 | 1.85E-50 | Pan-kidney |
| 25780 | RASGRP3 | 1.607976988 | 8.41E-51 | Pan-kidney |
| 91543 | RSAD2 | 1.60826952 | 1.70E-42 | Pan-kidney |
| 1272 | CNTN1 | 1.608373536 | 3.08E-13 | Pan-kidney |
| 83742 | MARVELD1 | 1.60889614 | 5.59E-30 | Pan-kidney |
| 1831 | TSC22D3 | 1.609222348 | 1.20E-36 | Pan-kidney |
| 147968 | CAPN12 | 1.609403233 | 5.37E-19 | Pan-kidney |
| 9497 | SLC4A7 | 1.609913824 | 2.90E-43 | Pan-kidney |
| 84867 | PTPN5 | 1.609996226 | 2.64E-21 | Pan-kidney |
| 9771 | RAPGEF5 | 1.610225123 | 7.98E-35 | Pan-kidney |
| 9212 | AURKB | 1.610321609 | 9.89E-26 | Pan-kidney |
| 11219 | TREX2 | 1.610374188 | 1.59E-18 | Pan-kidney |
| 7097 | TLR2 | 1.610467702 | 7.08E-61 | Pan-kidney |
| 166863 | RBM46 | 1.610507961 | 1.69E-24 | Pan-kidney |
| 6319 | SCD | 1.611600085 | 4.32E-27 | Pan-kidney |
| 248 | ALPI | 1.611758176 | 5.82E-07 | Pan-kidney |
| 4067 | LYN | 1.612352192 | 4.28E-76 | Pan-kidney |
| 440854 | CAPN14 | 1.612902116 | 1.40E-18 | Pan-kidney |
| 3310 | HSPA6 | 1.613342221 | 6.64E-32 | Pan-kidney |
| 50937 | CDON | 1.613448255 | 7.30E-29 | Pan-kidney |
| 7136 | TNNI2 | 1.613503806 | 1.23E-25 | Pan-kidney |
| 85409 | NKD2 | 1.614987508 | 1.29E-13 | Pan-kidney |
| 5033 | P4HA1 | 1.617197164 | 4.43E-59 | Pan-kidney |
| 1396 | CRIP1 | 1.618663522 | 1.36E-17 | Pan-kidney |
| 388646 | GBP7 | 1.619151678 | 2.70E-22 | Pan-kidney |
| 2859 | GPR35 | 1.619597479 | 1.64E-17 | Pan-kidney |
| 153768 | PRELID2 | 1.62010727 | 1.12E-37 | Pan-kidney |
| 2627 | GATA6 | 1.620407944 | 8.23E-29 | Pan-kidney |
| 129607 | CMPK2 | 1.620532867 | 6.69E-57 | Pan-kidney |
| 137835 | TMEM71 | 1.621182252 | 1.90E-28 | Pan-kidney |
| 4915 | NTRK2 | 1.621289183 | 3.23E-14 | Pan-kidney |
| 81501 | DCSTAMP | 1.622550016 | 1.32E-19 | Pan-kidney |
| 114904 | C1QTNF6 | 1.623026847 | 1.90E-42 | Pan-kidney |
| 401944 | LDLRAD2 | 1.623113812 | 1.19E-38 | Pan-kidney |
| 57405 | SPC25 | 1.623556711 | 1.05E-28 | Pan-kidney |
| 64218 | SEMA4A | 1.623767778 | 1.88E-40 | Pan-kidney |
| 5327 | PLAT | 1.624759756 | 9.90E-39 | Pan-kidney |
| 4004 | LMO1 | 1.625965521 | 2.59E-17 | Pan-kidney |
| 126282 | TNFAIP8L1 | 1.626257885 | 2.28E-53 | Pan-kidney |
| 80111 | SLCO2A1 | 1.626374215 | 2.89E-22 | Pan-kidney |
| 440712 | RHEX | 1.627449103 | 3.92E-07 | Pan-kidney |
| 976 | ADGRE5 | 1.628852588 | 1.21E-38 | Pan-kidney |
| 221476 | PI16 | 1.629428171 | 7.17E-13 | Pan-kidney |
| 79037 | PVRIG | 1.629439958 | 9.37E-49 | Pan-kidney |
| 89796 | NAV1 | 1.629631958 | 1.18E-50 | Pan-kidney |
| 60437 | CDH26 | 1.629635028 | 6.19E-39 | Pan-kidney |
| 163782 | KANK4 | 1.629662824 | 2.75E-22 | Pan-kidney |
| 7292 | TNFSF4 | 1.62971636 | 1.19E-62 | Pan-kidney |
| 80727 | TTYH3 | 1.629888523 | 1.06E-33 | Pan-kidney |
| 6252 | RTN1 | 1.629903763 | 4.09E-34 | Pan-kidney |
| 1944 | EFNA3 | 1.63077844 | 7.56E-43 | Pan-kidney |
| 257407 | C2orf72 | 1.631611765 | 6.09E-12 | Pan-kidney |
| 407977 | TNFSF12-TNFSF13 | 1.631877731 | 7.69E-11 | Pan-kidney |
| 80714 | PBX4 | 1.632107034 | 6.31E-22 | Pan-kidney |
| 4199 | ME1 | 1.632394868 | 2.27E-21 | Pan-kidney |
| 1545 | CYP1B1 | 1.632480033 | 2.40E-20 | Pan-kidney |
| 140885 | SIRPA | 1.633303893 | 5.83E-25 | Pan-kidney |
| 8507 | ENC1 | 1.633755238 | 1.13E-52 | Pan-kidney |
| 4237 | MFAP2 | 1.634106917 | 1.53E-13 | Pan-kidney |
| 7432 | VIP | 1.634980175 | 5.19E-24 | Pan-kidney |
| 163115 | ZNF781 | 1.635209052 | 1.73E-28 | Pan-kidney |
| 11027 | LILRA2 | 1.635273159 | 1.03E-27 | Pan-kidney |
| 165215 | FAM171B | 1.635799111 | 7.72E-20 | Pan-kidney |
| 9050 | PSTPIP2 | 1.637776887 | 1.53E-51 | Pan-kidney |
| 440193 | CCDC88C | 1.638257139 | 3.62E-38 | Pan-kidney |
| 636 | BICD1 | 1.638739175 | 2.09E-34 | Pan-kidney |
| 4001 | LMNB1 | 1.638896684 | 8.63E-51 | Pan-kidney |
| 4647 | MYO7A | 1.639299874 | 4.52E-22 | Pan-kidney |
| 654346 | LGALS9C | 1.643481952 | 4.51E-33 | Pan-kidney |
| 10384 | BTN3A3 | 1.643840517 | 1.29E-75 | Pan-kidney |
| 5997 | RGS2 | 1.643881548 | 1.70E-37 | Pan-kidney |
| 244 | None | 1.644239882 | 7.07E-15 | Pan-kidney |
| 9381 | OTOF | 1.645060328 | 2.61E-28 | Pan-kidney |
| 3576 | CXCL8 | 1.645533216 | 4.89E-14 | Pan-kidney |
| 5266 | PI3 | 1.646554686 | 1.91E-10 | Pan-kidney |
| 55876 | GSDMB | 1.646904794 | 2.72E-22 | Pan-kidney |
| 5256 | PHKA2 | 1.647344959 | 6.30E-63 | Pan-kidney |
| 80332 | ADAM33 | 1.647608186 | 1.52E-12 | Pan-kidney |
| 11167 | FSTL1 | 1.648138234 | 2.96E-37 | Pan-kidney |
| 2919 | CXCL1 | 1.648578818 | 5.44E-09 | Pan-kidney |
| 2204 | FCAR | 1.649875708 | 2.83E-29 | Pan-kidney |
| 2568 | GABRP | 1.65011006 | 8.81E-13 | Pan-kidney |
| 256227 | STEAP1B | 1.650266342 | 2.37E-28 | Pan-kidney |
| 3957 | LGALS2 | 1.650857434 | 1.30E-07 | Pan-kidney |
| 1409 | CRYAA | 1.651375759 | 1.20E-12 | Pan-kidney |
| 6291 | SAA4 | 1.652832573 | 1.59E-13 | Pan-kidney |
| 2921 | CXCL3 | 1.652919457 | 3.58E-18 | Pan-kidney |
| 7262 | PHLDA2 | 1.653623522 | 5.24E-27 | Pan-kidney |
| 114036 | None | 1.653637913 | 3.12E-43 | Pan-kidney |
| 283 | ANG | 1.654216936 | 6.69E-18 | Pan-kidney |
| 7188 | TRAF5 | 1.654597427 | 3.03E-58 | Pan-kidney |
| 5507 | PPP1R3C | 1.654685025 | 2.11E-28 | Pan-kidney |
| 3687 | ITGAX | 1.655854246 | 5.60E-31 | Pan-kidney |
| 112464 | CAVIN3 | 1.656163717 | 3.34E-29 | Pan-kidney |
| 10643 | IGF2BP3 | 1.656960469 | 5.77E-16 | Pan-kidney |
| 2982 | GUCY1A1 | 1.659223472 | 7.58E-36 | Pan-kidney |
| 7042 | TGFB2 | 1.659735388 | 1.88E-37 | Pan-kidney |
| 968 | CD68 | 1.659996498 | 3.10E-28 | Pan-kidney |
| 5924 | RASGRF2 | 1.661550207 | 6.01E-14 | Pan-kidney |
| 57595 | PDZD4 | 1.66181966 | 1.98E-34 | Pan-kidney |
| 800 | CALD1 | 1.66223402 | 1.18E-45 | Pan-kidney |
| 6091 | ROBO1 | 1.662268433 | 1.67E-27 | Pan-kidney |
| 22873 | DZIP1 | 1.662573185 | 1.22E-20 | Pan-kidney |
| 4500 | None | 1.662900831 | 6.57E-15 | Pan-kidney |
| 5360 | PLTP | 1.662918685 | 9.35E-20 | Pan-kidney |
| 80274 | SCUBE1 | 1.664036367 | 3.99E-15 | Pan-kidney |
| 10219 | KLRG1 | 1.664084852 | 6.33E-43 | Pan-kidney |
| 2208 | FCER2 | 1.664203119 | 4.68E-24 | Pan-kidney |
| 8972 | MGAM | 1.664959341 | 7.44E-09 | Pan-kidney |
| 5791 | PTPRE | 1.665868925 | 1.64E-69 | Pan-kidney |
| 10398 | MYL9 | 1.666687101 | 2.56E-42 | Pan-kidney |
| 4036 | LRP2 | 1.666880586 | 5.10E-05 | Pan-kidney |
| 100130889 | None | 1.667254698 | 3.30E-17 | Pan-kidney |
| 400831 | C20orf202 | 1.669967575 | 1.18E-35 | Pan-kidney |
| 3795 | KHK | 1.670082915 | 3.24E-14 | Pan-kidney |
| 10125 | RASGRP1 | 1.670276066 | 7.70E-32 | Pan-kidney |
| 5801 | PTPRR | 1.670393741 | 3.94E-22 | Pan-kidney |
| 57121 | LPAR5 | 1.67060438 | 4.66E-47 | Pan-kidney |
| 27287 | VENTX | 1.671267591 | 5.96E-35 | Pan-kidney |
| 185 | AGTR1 | 1.67138765 | 4.76E-14 | Pan-kidney |
| 9156 | EXO1 | 1.671919002 | 4.76E-33 | Pan-kidney |
| 25789 | None | 1.673161416 | 6.47E-20 | Pan-kidney |
| 9891 | NUAK1 | 1.673631622 | 1.13E-68 | Pan-kidney |
| 5241 | PGR | 1.675355606 | 1.16E-10 | Pan-kidney |
| 6708 | SPTA1 | 1.675777262 | 1.01E-32 | Pan-kidney |
| 148398 | SAMD11 | 1.675961305 | 7.59E-12 | Pan-kidney |
| 4804 | NGFR | 1.676051391 | 1.03E-13 | Pan-kidney |
| 414236 | C10orf55 | 1.676988392 | 3.75E-32 | Pan-kidney |
| 675 | BRCA2 | 1.679044911 | 6.67E-37 | Pan-kidney |
| 56130 | PCDHB6 | 1.679240695 | 4.49E-21 | Pan-kidney |
| 6857 | SYT1 | 1.680481522 | 1.13E-20 | Pan-kidney |
| 64167 | ERAP2 | 1.680813335 | 4.76E-19 | Pan-kidney |
| 84000 | TMPRSS13 | 1.681177099 | 5.61E-21 | Pan-kidney |
| 4035 | LRP1 | 1.681605419 | 1.32E-59 | Pan-kidney |
| 126731 | CCSAP | 1.682604403 | 7.25E-81 | Pan-kidney |
| 392636 | AGMO | 1.683465261 | 1.26E-08 | Pan-kidney |
| 55165 | CEP55 | 1.683551508 | 1.64E-37 | Pan-kidney |
| 79734 | KCTD17 | 1.683635054 | 2.62E-38 | Pan-kidney |
| 7103 | TSPAN8 | 1.683717747 | 2.69E-09 | Pan-kidney |
| 9715 | FAM131B | 1.684182046 | 4.74E-36 | Pan-kidney |
| 120329 | None | 1.684350922 | 4.35E-26 | Pan-kidney |
| 6356 | CCL11 | 1.684613992 | 2.12E-19 | Pan-kidney |
| 387119 | CEP85L | 1.684729224 | 4.44E-38 | Pan-kidney |
| 81607 | NECTIN4 | 1.684788797 | 4.10E-16 | Pan-kidney |
| 24137 | KIF4A | 1.685479653 | 1.60E-31 | Pan-kidney |
| 29992 | PILRA | 1.685687661 | 2.23E-49 | Pan-kidney |
| 55711 | FAR2 | 1.6864746 | 1.73E-36 | Pan-kidney |
| 7480 | WNT10B | 1.686745587 | 1.58E-20 | Pan-kidney |
| 2004 | ELK3 | 1.687792826 | 9.90E-40 | Pan-kidney |
| 56938 | ARNTL2 | 1.688402698 | 5.41E-26 | Pan-kidney |
| 4916 | NTRK3 | 1.689177355 | 9.91E-18 | Pan-kidney |
| 287 | ANK2 | 1.689928169 | 1.37E-16 | Pan-kidney |
| 199675 | MCEMP1 | 1.691359873 | 2.62E-22 | Pan-kidney |
| 90249 | UNC5A | 1.691384877 | 5.92E-17 | Pan-kidney |
| 55454 | CSGALNACT2 | 1.691729675 | 6.54E-79 | Pan-kidney |
| 23604 | DAPK2 | 1.6924677 | 1.89E-26 | Pan-kidney |
| 126410 | CYP4F22 | 1.693132363 | 6.96E-34 | Pan-kidney |
| 9095 | TBX19 | 1.693144806 | 2.53E-45 | Pan-kidney |
| 3766 | KCNJ10 | 1.69336639 | 2.66E-15 | Pan-kidney |
| 25790 | CFAP45 | 1.694229212 | 6.40E-26 | Pan-kidney |
| 2784 | GNB3 | 1.694409511 | 1.43E-21 | Pan-kidney |
| 10161 | LPAR6 | 1.695135991 | 2.28E-44 | Pan-kidney |
| 56670 | SUCNR1 | 1.695243585 | 2.10E-11 | Pan-kidney |
| 728 | C5AR1 | 1.695308754 | 7.02E-56 | Pan-kidney |
| 148345 | C1orf127 | 1.695479379 | 8.95E-34 | Pan-kidney |
| 653820 | FAM72B | 1.695603852 | 1.82E-49 | Pan-kidney |
| 27147 | DENND2A | 1.696539745 | 2.48E-23 | Pan-kidney |
| 55803 | ADAP2 | 1.697002592 | 1.25E-58 | Pan-kidney |
| 8870 | IER3 | 1.699458094 | 6.63E-38 | Pan-kidney |
| 3904 | LAIR2 | 1.699714032 | 3.61E-27 | Pan-kidney |
| 1748 | DLX4 | 1.700048701 | 2.21E-23 | Pan-kidney |
| 83700 | JAM3 | 1.70022426 | 1.24E-31 | Pan-kidney |
| 84570 | COL25A1 | 1.700755148 | 1.04E-16 | Pan-kidney |
| 286133 | SCARA5 | 1.701853853 | 2.51E-15 | Pan-kidney |
| 9173 | IL1RL1 | 1.702924893 | 1.03E-11 | Pan-kidney |
| 51285 | RASL12 | 1.703087045 | 9.46E-28 | Pan-kidney |
| 80723 | SLC35G2 | 1.705070061 | 8.93E-45 | Pan-kidney |
| 148170 | CDC42EP5 | 1.705552712 | 1.10E-38 | Pan-kidney |
| 59352 | LGR6 | 1.707028069 | 1.37E-27 | Pan-kidney |
| 9971 | NR1H4 | 1.707606394 | 1.00E-09 | Pan-kidney |
| 2662 | GDF10 | 1.707714608 | 8.42E-25 | Pan-kidney |
| 6555 | SLC10A2 | 1.70891227 | 5.93E-08 | Pan-kidney |
| 138065 | RNF183 | 1.709017882 | 1.59E-12 | Pan-kidney |
| 80328 | ULBP2 | 1.709056568 | 1.15E-35 | Pan-kidney |
| 23127 | COLGALT2 | 1.709253731 | 5.92E-23 | Pan-kidney |
| 653145 | ANXA8 | 1.709367254 | 1.93E-15 | Pan-kidney |
| 57480 | PLEKHG1 | 1.710457634 | 2.30E-50 | Pan-kidney |
| 203522 | INTS6L | 1.710746134 | 5.25E-49 | Pan-kidney |
| 7732 | RNF112 | 1.710835612 | 2.44E-30 | Pan-kidney |
| 4605 | MYBL2 | 1.711425488 | 1.77E-26 | Pan-kidney |
| 64388 | GREM2 | 1.711920361 | 5.31E-19 | Pan-kidney |
| 11259 | FILIP1L | 1.712423978 | 1.88E-37 | Pan-kidney |
| 7464 | CORO2A | 1.712920007 | 5.54E-33 | Pan-kidney |
| 6349 | CCL3L1 | 1.713212926 | 6.67E-17 | Pan-kidney |
| 57622 | LRFN1 | 1.713624381 | 7.19E-44 | Pan-kidney |
| 126014 | OSCAR | 1.714518919 | 3.15E-36 | Pan-kidney |
| 57636 | ARHGAP23 | 1.7148311 | 6.06E-49 | Pan-kidney |
| 2153 | F5 | 1.715722018 | 1.36E-09 | Pan-kidney |
| 79785 | RERGL | 1.715787039 | 1.72E-16 | Pan-kidney |
| 55809 | TRERF1 | 1.71617069 | 3.56E-46 | Pan-kidney |
| 81610 | FAM83D | 1.716790859 | 1.50E-36 | Pan-kidney |
| 347 | APOD | 1.717542401 | 3.33E-17 | Pan-kidney |
| 3241 | HPCAL1 | 1.717710655 | 2.05E-49 | Pan-kidney |
| 9473 | THEMIS2 | 1.717908937 | 1.81E-54 | Pan-kidney |
| 1750 | DLX6 | 1.718956207 | 4.34E-31 | Pan-kidney |
| 3339 | HSPG2 | 1.719889749 | 7.29E-43 | Pan-kidney |
| 150726 | FBXO41 | 1.720690437 | 1.90E-26 | Pan-kidney |
| 148113 | CILP2 | 1.720772237 | 4.15E-19 | Pan-kidney |
| 24 | ABCA4 | 1.721185597 | 9.25E-25 | Pan-kidney |
| 326624 | RAB37 | 1.722495342 | 3.80E-37 | Pan-kidney |
| 9705 | ST18 | 1.722517029 | 2.08E-39 | Pan-kidney |
| 135398 | C6orf141 | 1.72293073 | 2.15E-17 | Pan-kidney |
| 57616 | TSHZ3 | 1.723284288 | 5.03E-39 | Pan-kidney |
| 150468 | CKAP2L | 1.723405633 | 2.87E-31 | Pan-kidney |
| 404037 | HAPLN4 | 1.723538689 | 4.20E-17 | Pan-kidney |
| 3956 | LGALS1 | 1.723753028 | 1.15E-48 | Pan-kidney |
| 6261 | RYR1 | 1.724193158 | 1.34E-26 | Pan-kidney |
| 440603 | BCL2L15 | 1.72430622 | 2.75E-20 | Pan-kidney |
| 4948 | OCA2 | 1.724587507 | 6.07E-16 | Pan-kidney |
| 55619 | DOCK10 | 1.724810492 | 1.76E-28 | Pan-kidney |
| 84541 | KBTBD8 | 1.725426321 | 5.59E-52 | Pan-kidney |
| 100129034 | None | 1.726181436 | 5.25E-27 | Pan-kidney |
| 283663 | None | 1.726308442 | 1.49E-40 | Pan-kidney |
| 256236 | None | 1.726351812 | 1.53E-22 | Pan-kidney |
| 3109 | HLA-DMB | 1.726686335 | 8.27E-59 | Pan-kidney |
| 6003 | RGS13 | 1.728007148 | 5.04E-34 | Pan-kidney |
| 650655 | None | 1.728260965 | 4.26E-25 | Pan-kidney |
| 51559 | NT5DC3 | 1.72843418 | 5.68E-39 | Pan-kidney |
| 6422 | SFRP1 | 1.728504114 | 3.35E-13 | Pan-kidney |
| 50614 | GALNT9 | 1.728604806 | 1.19E-10 | Pan-kidney |
| 84941 | HSH2D | 1.728851051 | 2.87E-30 | Pan-kidney |
| 10769 | PLK2 | 1.72887246 | 5.59E-45 | Pan-kidney |
| 9467 | SH3BP5 | 1.730601149 | 1.37E-34 | Pan-kidney |
| 64221 | ROBO3 | 1.731244631 | 4.85E-30 | Pan-kidney |
| 6916 | TBXAS1 | 1.73131965 | 5.35E-54 | Pan-kidney |
| 200132 | TCTEX1D1 | 1.73209615 | 1.01E-49 | Pan-kidney |
| 90381 | TICRR | 1.732375521 | 4.81E-39 | Pan-kidney |
| 1513 | CTSK | 1.732453681 | 1.19E-23 | Pan-kidney |
| 4688 | NCF2 | 1.732791723 | 1.49E-40 | Pan-kidney |
| 56129 | PCDHB7 | 1.733531388 | 5.25E-25 | Pan-kidney |
| 5010 | CLDN11 | 1.733848713 | 1.01E-21 | Pan-kidney |
| 84675 | TRIM55 | 1.734910932 | 1.04E-13 | Pan-kidney |
| 79660 | PPP1R3B | 1.73531309 | 2.90E-51 | Pan-kidney |
| 3169 | FOXA1 | 1.73596634 | 1.99E-17 | Pan-kidney |
| 80176 | SPSB1 | 1.736229333 | 8.72E-36 | Pan-kidney |
| 995 | CDC25C | 1.737674826 | 4.63E-27 | Pan-kidney |
| 143903 | LAYN | 1.738345985 | 9.79E-36 | Pan-kidney |
| 54578 | UGT1A6 | 1.738924529 | 6.61E-08 | Pan-kidney |
| 199786 | FAM129C | 1.740409293 | 4.78E-29 | Pan-kidney |
| 114088 | TRIM9 | 1.740504874 | 1.84E-16 | Pan-kidney |
| 64115 | VSIR | 1.740523562 | 1.77E-67 | Pan-kidney |
| 254559 | None | 1.742430811 | 8.14E-11 | Pan-kidney |
| 54898 | ELOVL2 | 1.742719673 | 6.43E-21 | Pan-kidney |
| 2638 | GC | 1.742730706 | 2.43E-09 | Pan-kidney |
| 7092 | TLL1 | 1.743297051 | 5.90E-21 | Pan-kidney |
| 4066 | LYL1 | 1.743530513 | 5.46E-58 | Pan-kidney |
| 5654 | HTRA1 | 1.743540052 | 8.19E-53 | Pan-kidney |
| 5972 | REN | 1.74425116 | 6.91E-07 | Pan-kidney |
| 170591 | S100Z | 1.744271184 | 1.83E-47 | Pan-kidney |
| 84034 | EMILIN2 | 1.745583713 | 5.88E-53 | Pan-kidney |
| 3730 | ANOS1 | 1.746416404 | 2.15E-30 | Pan-kidney |
| 9514 | GAL3ST1 | 1.746650974 | 4.49E-18 | Pan-kidney |
| 729533 | FAM72A | 1.747175725 | 3.69E-39 | Pan-kidney |
| 2906 | GRIN2D | 1.748638337 | 3.63E-34 | Pan-kidney |
| 26762 | HAVCR1 | 1.74952441 | 3.50E-07 | Pan-kidney |
| 55124 | PIWIL2 | 1.749916565 | 3.32E-33 | Pan-kidney |
| 163071 | ZNF114 | 1.750144223 | 1.43E-13 | Pan-kidney |
| 1307 | COL16A1 | 1.750269117 | 5.07E-27 | Pan-kidney |
| 26002 | MOXD1 | 1.750309758 | 2.95E-20 | Pan-kidney |
| 6514 | SLC2A2 | 1.751419678 | 2.32E-07 | Pan-kidney |
| 55350 | VNN3 | 1.753012233 | 8.18E-27 | Pan-kidney |
| 27295 | PDLIM3 | 1.753527259 | 1.21E-27 | Pan-kidney |
| 85452 | CFAP74 | 1.753635072 | 1.80E-33 | Pan-kidney |
| 112597 | None | 1.755314321 | 3.48E-51 | Pan-kidney |
| 22871 | NLGN1 | 1.755416574 | 3.42E-13 | Pan-kidney |
| 8510 | MMP23B | 1.755487663 | 6.89E-29 | Pan-kidney |
| 6512 | SLC1A7 | 1.755917278 | 3.90E-17 | Pan-kidney |
| 91450 | None | 1.756783125 | 4.76E-38 | Pan-kidney |
| 10346 | TRIM22 | 1.756853734 | 1.66E-51 | Pan-kidney |
| 353376 | TICAM2 | 1.757002856 | 6.11E-73 | Pan-kidney |
| 5271 | SERPINB8 | 1.757522043 | 7.20E-73 | Pan-kidney |
| 83540 | NUF2 | 1.758710919 | 3.55E-31 | Pan-kidney |
| 10112 | KIF20A | 1.759180861 | 5.28E-29 | Pan-kidney |
| 23255 | MTCL1 | 1.759808608 | 1.33E-24 | Pan-kidney |
| 1602 | DACH1 | 1.759953287 | 2.95E-17 | Pan-kidney |
| 8844 | KSR1 | 1.76061433 | 8.21E-45 | Pan-kidney |
| 23616 | SH3BP1 | 1.760900089 | 2.19E-66 | Pan-kidney |
| 84868 | HAVCR2 | 1.761258757 | 2.39E-22 | Pan-kidney |
| 121551 | BTBD11 | 1.762257453 | 6.51E-14 | Pan-kidney |
| 1606 | DGKA | 1.763432592 | 3.97E-40 | Pan-kidney |
| 6660 | SOX5 | 1.763591881 | 4.39E-17 | Pan-kidney |
| 863 | CBFA2T3 | 1.764239549 | 3.01E-49 | Pan-kidney |
| 121268 | RHEBL1 | 1.76435555 | 9.11E-52 | Pan-kidney |
| 246126 | None | 1.764879235 | 4.64E-10 | Pan-kidney |
| 6676 | SPAG4 | 1.765912618 | 2.75E-38 | Pan-kidney |
| 4288 | MKI67 | 1.766054225 | 3.11E-39 | Pan-kidney |
| 9953 | HS3ST3B1 | 1.766635606 | 1.03E-27 | Pan-kidney |
| 56849 | TCEAL7 | 1.767285252 | 6.27E-28 | Pan-kidney |
| 5155 | PDGFB | 1.767501593 | 7.94E-58 | Pan-kidney |
| 400581 | GRAPL | 1.768368643 | 1.19E-34 | Pan-kidney |
| 2625 | GATA3 | 1.769166451 | 1.35E-14 | Pan-kidney |
| 3577 | CXCR1 | 1.77033484 | 5.38E-23 | Pan-kidney |
| 26032 | SUSD5 | 1.770773638 | 8.62E-21 | Pan-kidney |
| 440836 | ODF3B | 1.770892425 | 4.77E-23 | Pan-kidney |
| 9595 | CYTIP | 1.770906413 | 5.19E-31 | Pan-kidney |
| 7781 | SLC30A3 | 1.771869778 | 1.10E-27 | Pan-kidney |
| 55893 | ZNF395 | 1.772145762 | 1.87E-51 | Pan-kidney |
| 259307 | IL4I1 | 1.774138052 | 2.32E-31 | Pan-kidney |
| 57088 | PLSCR4 | 1.774820901 | 1.89E-39 | Pan-kidney |
| 79100 | None | 1.775110003 | 2.65E-18 | Pan-kidney |
| 23495 | TNFRSF13B | 1.775340434 | 2.08E-30 | Pan-kidney |
| 84620 | ST6GAL2 | 1.776006357 | 2.61E-14 | Pan-kidney |
| 146909 | KIF18B | 1.776856348 | 2.68E-33 | Pan-kidney |
| 10019 | SH2B3 | 1.777520533 | 3.48E-79 | Pan-kidney |
| 29933 | GPR132 | 1.777653277 | 1.38E-46 | Pan-kidney |
| 253018 | None | 1.778251002 | 3.22E-29 | Pan-kidney |
| 79092 | CARD14 | 1.778269083 | 2.53E-24 | Pan-kidney |
| 4277 | MICB | 1.780249543 | 4.13E-65 | Pan-kidney |
| 2192 | FBLN1 | 1.78025293 | 7.39E-26 | Pan-kidney |
| 22846 | VASH1 | 1.780844291 | 8.70E-75 | Pan-kidney |
| 11040 | PIM2 | 1.78104564 | 3.00E-66 | Pan-kidney |
| 51816 | ADA2 | 1.781102756 | 8.75E-35 | Pan-kidney |
| 57493 | HEG1 | 1.782607075 | 2.89E-54 | Pan-kidney |
| 25907 | TMEM158 | 1.782865723 | 8.87E-26 | Pan-kidney |
| 624 | BDKRB2 | 1.783475194 | 1.92E-14 | Pan-kidney |
| 350 | APOH | 1.783557823 | 3.68E-14 | Pan-kidney |
| 7399 | USH2A | 1.783780272 | 4.48E-41 | Pan-kidney |
| 827 | CAPN6 | 1.784353025 | 5.44E-08 | Pan-kidney |
| 54209 | TREM2 | 1.785749197 | 4.65E-32 | Pan-kidney |
| 25999 | CLIP3 | 1.78575926 | 1.23E-30 | Pan-kidney |
| 219285 | SAMD9L | 1.78586802 | 8.09E-55 | Pan-kidney |
| 391059 | FRRS1 | 1.786224801 | 1.59E-29 | Pan-kidney |
| 54596 | L1TD1 | 1.787313433 | 5.79E-33 | Pan-kidney |
| 151306 | GPBAR1 | 1.787638095 | 2.62E-37 | Pan-kidney |
| 285596 | FAM153A | 1.788690765 | 2.73E-12 | Pan-kidney |
| 575 | ADGRB1 | 1.788720535 | 3.26E-15 | Pan-kidney |
| 5453 | POU3F1 | 1.789441744 | 1.73E-44 | Pan-kidney |
| 4900 | NRGN | 1.790382579 | 5.83E-45 | Pan-kidney |
| 728833 | FAM72D | 1.790573131 | 2.27E-45 | Pan-kidney |
| 389058 | SP5 | 1.790680092 | 4.43E-16 | Pan-kidney |
| 57094 | CPA6 | 1.791332198 | 1.86E-21 | Pan-kidney |
| 2049 | EPHB3 | 1.791724577 | 3.05E-22 | Pan-kidney |
| 150 | ADRA2A | 1.791945982 | 2.29E-22 | Pan-kidney |
| 91828 | EXOC3L4 | 1.791953027 | 4.13E-11 | Pan-kidney |
| 51268 | PIPOX | 1.792068349 | 4.99E-16 | Pan-kidney |
| 148229 | ATP8B3 | 1.792785527 | 2.66E-21 | Pan-kidney |
| 8736 | MYOM1 | 1.79475381 | 2.34E-36 | Pan-kidney |
| 115123 | Mar-03 | 1.795000357 | 2.93E-57 | Pan-kidney |
| 221393 | ADGRF4 | 1.795006168 | 1.72E-17 | Pan-kidney |
| 9398 | CD101 | 1.795567828 | 7.20E-63 | Pan-kidney |
| 55635 | DEPDC1 | 1.795693964 | 7.28E-31 | Pan-kidney |
| 140564 | APOBEC3D | 1.795713091 | 1.84E-41 | Pan-kidney |
| 9201 | DCLK1 | 1.797439586 | 9.84E-20 | Pan-kidney |
| 7033 | TFF3 | 1.798614813 | 8.40E-18 | Pan-kidney |
| 3579 | CXCR2 | 1.801067297 | 7.67E-30 | Pan-kidney |
| 7138 | TNNT1 | 1.801781022 | 5.11E-11 | Pan-kidney |
| 9856 | KIAA0319 | 1.802635717 | 2.51E-18 | Pan-kidney |
| 135250 | RAET1E | 1.803068992 | 1.73E-34 | Pan-kidney |
| 51705 | EMCN | 1.804230495 | 3.79E-26 | Pan-kidney |
| 8514 | KCNAB2 | 1.80508721 | 2.53E-40 | Pan-kidney |
| 9787 | DLGAP5 | 1.805969779 | 1.14E-29 | Pan-kidney |
| 54922 | RASIP1 | 1.806027575 | 3.89E-38 | Pan-kidney |
| 3640 | INSL3 | 1.806341224 | 4.07E-32 | Pan-kidney |
| 27033 | ZBTB32 | 1.806582212 | 2.65E-42 | Pan-kidney |
| 53345 | TM6SF2 | 1.80709616 | 5.88E-12 | Pan-kidney |
| 5199 | CFP | 1.808916805 | 5.79E-34 | Pan-kidney |
| 284611 | FAM102B | 1.808979148 | 2.17E-59 | Pan-kidney |
| 4493 | MT1E | 1.809406489 | 2.82E-18 | Pan-kidney |
| 114132 | SIGLEC11 | 1.8110334 | 3.63E-32 | Pan-kidney |
| 57096 | RPGRIP1 | 1.812297144 | 2.50E-50 | Pan-kidney |
| 57194 | ATP10A | 1.812419917 | 2.33E-22 | Pan-kidney |
| 55365 | TMEM176A | 1.812428223 | 6.31E-12 | Pan-kidney |
| 8942 | KYNU | 1.813851075 | 2.05E-23 | Pan-kidney |
| 84808 | PERM1 | 1.815607727 | 1.79E-15 | Pan-kidney |
| 10581 | IFITM2 | 1.81664759 | 1.08E-52 | Pan-kidney |
| 83879 | CDCA7 | 1.816863309 | 5.39E-29 | Pan-kidney |
| 5376 | PMP22 | 1.817336512 | 1.90E-42 | Pan-kidney |
| 10184 | LHFPL2 | 1.81966762 | 9.18E-72 | Pan-kidney |
| 118663 | BTBD16 | 1.819752561 | 9.66E-20 | Pan-kidney |
| 389125 | MUSTN1 | 1.820266609 | 1.10E-33 | Pan-kidney |
| 780776 | TVP23A | 1.821530223 | 6.88E-64 | Pan-kidney |
| 23460 | ABCA6 | 1.823566379 | 4.14E-20 | Pan-kidney |
| 9573 | GDF3 | 1.823818144 | 4.66E-30 | Pan-kidney |
| 339977 | LRRC66 | 1.824357604 | 1.89E-25 | Pan-kidney |
| 11075 | STMN2 | 1.824796231 | 5.17E-18 | Pan-kidney |
| 79722 | ANKRD55 | 1.826749424 | 1.49E-44 | Pan-kidney |
| 56107 | PCDHGA9 | 1.826778144 | 3.23E-18 | Pan-kidney |
| 55901 | THSD1 | 1.827722803 | 2.68E-46 | Pan-kidney |
| 8542 | APOL1 | 1.828355036 | 1.02E-33 | Pan-kidney |
| 27439 | TMEM121B | 1.82851672 | 6.89E-46 | Pan-kidney |
| 8061 | FOSL1 | 1.829639193 | 4.33E-26 | Pan-kidney |
| 168667 | BMPER | 1.829918793 | 6.65E-27 | Pan-kidney |
| 11149 | BVES | 1.830354487 | 1.74E-30 | Pan-kidney |
| 54809 | SAMD9 | 1.830479084 | 1.28E-56 | Pan-kidney |
| 57188 | ADAMTSL3 | 1.830480472 | 8.36E-20 | Pan-kidney |
| 3239 | HOXD13 | 1.831951637 | 4.90E-29 | Pan-kidney |
| 10865 | ARID5A | 1.833084608 | 1.06E-70 | Pan-kidney |
| 80709 | AKNA | 1.833105639 | 4.76E-83 | Pan-kidney |
| 57713 | SFMBT2 | 1.835868983 | 6.67E-37 | Pan-kidney |
| 10083 | USH1C | 1.836432481 | 3.26E-07 | Pan-kidney |
| 333 | APLP1 | 1.837200232 | 1.00E-18 | Pan-kidney |
| 8404 | SPARCL1 | 1.837273926 | 9.02E-17 | Pan-kidney |
| 389119 | INKA1 | 1.838079992 | 3.53E-49 | Pan-kidney |
| 6678 | SPARC | 1.83817132 | 7.10E-46 | Pan-kidney |
| 64805 | P2RY12 | 1.838325181 | 7.06E-26 | Pan-kidney |
| 221692 | PHACTR1 | 1.838799453 | 1.39E-41 | Pan-kidney |
| 3299 | HSF4 | 1.840477004 | 1.06E-26 | Pan-kidney |
| 1558 | CYP2C8 | 1.840515358 | 9.45E-16 | Pan-kidney |
| 58504 | ARHGAP22 | 1.84344666 | 1.12E-44 | Pan-kidney |
| 5413 | Sep-05 | 1.844201696 | 1.42E-38 | Pan-kidney |
| 339834 | CCDC36 | 1.844351838 | 1.54E-38 | Pan-kidney |
| 9086 | EIF1AY | 1.844476913 | 1.71E-08 | Pan-kidney |
| 8082 | SSPN | 1.845151728 | 4.88E-42 | Pan-kidney |
| 353500 | BMP8A | 1.845176121 | 1.23E-50 | Pan-kidney |
| 6329 | SCN4A | 1.845824291 | 7.54E-18 | Pan-kidney |
| 10875 | FGL2 | 1.846079728 | 1.06E-32 | Pan-kidney |
| 8912 | CACNA1H | 1.846254797 | 1.21E-28 | Pan-kidney |
| 8643 | PTCH2 | 1.847771517 | 1.14E-39 | Pan-kidney |
| 4843 | NOS2 | 1.848329929 | 8.16E-24 | Pan-kidney |
| 284367 | None | 1.849265273 | 4.32E-38 | Pan-kidney |
| 55240 | STEAP3 | 1.849717741 | 9.90E-30 | Pan-kidney |
| 100048912 | None | 1.85019865 | 3.40E-34 | Pan-kidney |
| 283849 | EXOC3L1 | 1.850579115 | 1.45E-45 | Pan-kidney |
| 341 | APOC1 | 1.851563137 | 4.00E-22 | Pan-kidney |
| 10266 | RAMP2 | 1.851675798 | 4.09E-39 | Pan-kidney |
| 2274 | FHL2 | 1.85202813 | 1.60E-28 | Pan-kidney |
| 4638 | MYLK | 1.85235072 | 1.32E-43 | Pan-kidney |
| 10740 | None | 1.852719785 | 2.64E-45 | Pan-kidney |
| 7462 | LAT2 | 1.853172803 | 2.74E-58 | Pan-kidney |
| 3067 | HDC | 1.853235116 | 3.78E-20 | Pan-kidney |
| 240 | ALOX5 | 1.853394792 | 1.94E-25 | Pan-kidney |
| 4162 | None | 1.854700822 | 1.51E-50 | Pan-kidney |
| 3824 | KLRD1 | 1.854716099 | 1.02E-44 | Pan-kidney |
| 4241 | MELTF | 1.85590295 | 9.79E-20 | Pan-kidney |
| 2674 | GFRA1 | 1.856189551 | 3.27E-13 | Pan-kidney |
| 7171 | TPM4 | 1.856830749 | 1.76E-71 | Pan-kidney |
| 255809 | C19orf38 | 1.857810093 | 6.34E-64 | Pan-kidney |
| 79570 | NKAIN1 | 1.859591639 | 5.53E-24 | Pan-kidney |
| 284417 | TMEM150B | 1.860290394 | 4.12E-25 | Pan-kidney |
| 146433 | IL34 | 1.861357269 | 6.63E-36 | Pan-kidney |
| 6424 | SFRP4 | 1.86188308 | 9.03E-20 | Pan-kidney |
| 9928 | KIF14 | 1.861886134 | 3.20E-34 | Pan-kidney |
| 653390 | None | 1.862223137 | 1.75E-42 | Pan-kidney |
| 152007 | GLIPR2 | 1.862255594 | 2.28E-59 | Pan-kidney |
| 144455 | E2F7 | 1.862328435 | 6.28E-38 | Pan-kidney |
| 85407 | NKD1 | 1.863081533 | 2.83E-39 | Pan-kidney |
| 26548 | ITGB1BP2 | 1.863996772 | 4.42E-44 | Pan-kidney |
| 2034 | EPAS1 | 1.86403166 | 2.58E-48 | Pan-kidney |
| 221395 | ADGRF5 | 1.864040331 | 1.17E-17 | Pan-kidney |
| 4671 | NAIP | 1.864111744 | 1.95E-58 | Pan-kidney |
| 7351 | UCP2 | 1.864593405 | 2.62E-44 | Pan-kidney |
| 3293 | HSD17B3 | 1.864627799 | 2.18E-19 | Pan-kidney |
| 79739 | TTLL7 | 1.864886119 | 8.04E-20 | Pan-kidney |
| 4499 | MT1M | 1.864925901 | 3.05E-16 | Pan-kidney |
| 7096 | TLR1 | 1.865555276 | 1.23E-55 | Pan-kidney |
| 1812 | DRD1 | 1.865814006 | 1.92E-33 | Pan-kidney |
| 64174 | DPEP2 | 1.866689427 | 2.02E-41 | Pan-kidney |
| 6943 | TCF21 | 1.86781579 | 5.32E-24 | Pan-kidney |
| 9537 | TP53I11 | 1.868390023 | 5.31E-33 | Pan-kidney |
| 54674 | LRRN3 | 1.868866242 | 6.99E-35 | Pan-kidney |
| 8483 | CILP | 1.869512463 | 1.11E-24 | Pan-kidney |
| 54847 | SIDT1 | 1.870014329 | 3.51E-38 | Pan-kidney |
| 7062 | TCHH | 1.87101541 | 8.58E-27 | Pan-kidney |
| 1435 | CSF1 | 1.871072704 | 4.60E-61 | Pan-kidney |
| 205 | AK4 | 1.871983861 | 9.01E-20 | Pan-kidney |
| 1490 | CCN2 | 1.873934896 | 1.87E-42 | Pan-kidney |
| 6280 | S100A9 | 1.87453497 | 7.86E-30 | Pan-kidney |
| 117289 | TAGAP | 1.874685313 | 3.56E-42 | Pan-kidney |
| 121355 | GTSF1 | 1.875140483 | 8.68E-32 | Pan-kidney |
| 85376 | RIMBP3 | 1.875540288 | 5.21E-53 | Pan-kidney |
| 586 | BCAT1 | 1.875779149 | 1.14E-31 | Pan-kidney |
| 1675 | CFD | 1.876128865 | 7.54E-30 | Pan-kidney |
| 290 | ANPEP | 1.876142859 | 6.47E-10 | Pan-kidney |
| 116835 | HSPA12B | 1.878040368 | 1.25E-44 | Pan-kidney |
| 9424 | KCNK6 | 1.878104736 | 1.84E-64 | Pan-kidney |
| 26222 | None | 1.878571317 | 4.67E-24 | Pan-kidney |
| 7051 | TGM1 | 1.878748231 | 7.93E-22 | Pan-kidney |
| 125206 | SLC5A10 | 1.880031847 | 2.80E-08 | Pan-kidney |
| 399959 | None | 1.880057325 | 3.31E-24 | Pan-kidney |
| 2893 | GRIA4 | 1.880336597 | 1.24E-08 | Pan-kidney |
| 79368 | FCRL2 | 1.880619072 | 8.67E-27 | Pan-kidney |
| 1946 | EFNA5 | 1.882385871 | 1.33E-17 | Pan-kidney |
| 8115 | None | 1.882772097 | 1.22E-27 | Pan-kidney |
| 2275 | FHL3 | 1.885265253 | 1.11E-67 | Pan-kidney |
| 348938 | NIPAL4 | 1.88576231 | 2.46E-21 | Pan-kidney |
| 5346 | PLIN1 | 1.885901814 | 5.79E-27 | Pan-kidney |
| 55355 | HJURP | 1.886215135 | 4.40E-37 | Pan-kidney |
| 115350 | FCRL1 | 1.886336696 | 1.25E-32 | Pan-kidney |
| 8284 | KDM5D | 1.886960344 | 5.45E-07 | Pan-kidney |
| 23414 | ZFPM2 | 1.887216938 | 2.92E-32 | Pan-kidney |
| 51313 | GASK1B | 1.887669852 | 3.60E-22 | Pan-kidney |
| 57575 | PCDH10 | 1.88928897 | 1.21E-10 | Pan-kidney |
| 91010 | FMNL3 | 1.889349783 | 2.89E-68 | Pan-kidney |
| 219855 | SLC37A2 | 1.889402049 | 4.40E-46 | Pan-kidney |
| 1755 | DMBT1 | 1.889926245 | 2.96E-30 | Pan-kidney |
| 8817 | FGF18 | 1.891528129 | 2.72E-39 | Pan-kidney |
| 2306 | FOXD2 | 1.89179661 | 3.15E-58 | Pan-kidney |
| 387890 | TMEM233 | 1.89207472 | 9.53E-19 | Pan-kidney |
| 6495 | SIX1 | 1.892728255 | 8.01E-16 | Pan-kidney |
| 54360 | CYTL1 | 1.893747492 | 2.62E-28 | Pan-kidney |
| 3635 | INPP5D | 1.894899024 | 3.13E-70 | Pan-kidney |
| 2697 | GJA1 | 1.895169992 | 3.74E-37 | Pan-kidney |
| 6556 | SLC11A1 | 1.895262442 | 2.70E-41 | Pan-kidney |
| 64595 | None | 1.895574357 | 4.07E-13 | Pan-kidney |
| 22891 | ZNF365 | 1.895884229 | 1.88E-19 | Pan-kidney |
| 9023 | CH25H | 1.896579185 | 3.34E-32 | Pan-kidney |
| 23601 | CLEC5A | 1.896708157 | 1.63E-26 | Pan-kidney |
| 30846 | EHD2 | 1.896864115 | 4.82E-41 | Pan-kidney |
| 140706 | CCM2L | 1.897291504 | 1.68E-39 | Pan-kidney |
| 158376 | SPAAR | 1.897373569 | 6.33E-38 | Pan-kidney |
| 22943 | DKK1 | 1.897823357 | 7.64E-24 | Pan-kidney |
| 118471 | PRAP1 | 1.899078479 | 5.41E-07 | Pan-kidney |
| 4129 | MAOB | 1.901180933 | 1.52E-12 | Pan-kidney |
| 4747 | NEFL | 1.902006587 | 3.35E-08 | Pan-kidney |
| 10170 | DHRS9 | 1.902920349 | 7.65E-25 | Pan-kidney |
| 23057 | NMNAT2 | 1.903183495 | 1.42E-22 | Pan-kidney |
| 27197 | GPR82 | 1.90495988 | 1.16E-35 | Pan-kidney |
| 387707 | CC2D2B | 1.905785569 | 1.97E-45 | Pan-kidney |
| 9645 | MICAL2 | 1.906700303 | 1.86E-56 | Pan-kidney |
| 4033 | LRMP | 1.906843426 | 3.87E-42 | Pan-kidney |
| 3669 | ISG20 | 1.907519036 | 5.39E-49 | Pan-kidney |
| 432 | ASGR1 | 1.908125206 | 8.70E-38 | Pan-kidney |
| 7538 | None | 1.909621556 | 2.95E-47 | Pan-kidney |
| 10326 | SIRPB1 | 1.910311152 | 6.24E-31 | Pan-kidney |
| 6347 | CCL2 | 1.910502534 | 1.65E-27 | Pan-kidney |
| 132720 | FAM241A | 1.910726644 | 3.75E-35 | Pan-kidney |
| 64236 | PDLIM2 | 1.911815074 | 5.62E-59 | Pan-kidney |
| 143689 | PIWIL4 | 1.91274093 | 3.32E-45 | Pan-kidney |
| 9697 | TRAM2 | 1.91311795 | 5.88E-74 | Pan-kidney |
| 140738 | TMEM37 | 1.913584978 | 1.25E-25 | Pan-kidney |
| 1608 | DGKG | 1.91499564 | 2.06E-27 | Pan-kidney |
| 56097 | PCDHGC5 | 1.920879103 | 3.32E-30 | Pan-kidney |
| 55359 | STYK1 | 1.922103133 | 1.61E-30 | Pan-kidney |
| 160428 | ALDH1L2 | 1.92260744 | 1.88E-25 | Pan-kidney |
| 64170 | CARD9 | 1.923607633 | 2.03E-44 | Pan-kidney |
| 10216 | PRG4 | 1.924184813 | 1.78E-26 | Pan-kidney |
| 2596 | GAP43 | 1.924191632 | 1.10E-33 | Pan-kidney |
| 85443 | DCLK3 | 1.924342394 | 2.08E-26 | Pan-kidney |
| 117248 | GALNT15 | 1.92547612 | 1.93E-27 | Pan-kidney |
| 7043 | TGFB3 | 1.92562097 | 1.51E-65 | Pan-kidney |
| 29785 | CYP2S1 | 1.926057379 | 1.47E-47 | Pan-kidney |
| 10344 | CCL26 | 1.926484854 | 2.70E-34 | Pan-kidney |
| 65997 | RASL11B | 1.927384873 | 6.28E-21 | Pan-kidney |
| 9603 | NFE2L3 | 1.927940898 | 1.34E-56 | Pan-kidney |
| 7364 | UGT2B7 | 1.928109934 | 6.10E-11 | Pan-kidney |
| 922 | CD5L | 1.930015642 | 2.98E-13 | Pan-kidney |
| 7941 | PLA2G7 | 1.930059111 | 1.61E-26 | Pan-kidney |
| 10178 | TENM1 | 1.93032014 | 3.30E-15 | Pan-kidney |
| 4057 | LTF | 1.930753949 | 1.10E-09 | Pan-kidney |
| 6688 | SPI1 | 1.930915256 | 2.69E-64 | Pan-kidney |
| 285973 | ATG9B | 1.93123297 | 1.31E-25 | Pan-kidney |
| 8013 | NR4A3 | 1.931436552 | 3.13E-21 | Pan-kidney |
| 169834 | None | 1.932242824 | 8.68E-22 | Pan-kidney |
| 51655 | RASD1 | 1.932244625 | 5.69E-15 | Pan-kidney |
| 7058 | THBS2 | 1.932489696 | 6.75E-23 | Pan-kidney |
| 2152 | F3 | 1.932530167 | 5.10E-29 | Pan-kidney |
| 1382 | CRABP2 | 1.933564857 | 6.59E-19 | Pan-kidney |
| 3899 | AFF3 | 1.933653796 | 3.57E-20 | Pan-kidney |
| 116039 | OSR2 | 1.935119316 | 1.51E-20 | Pan-kidney |
| 64091 | POPDC2 | 1.936829308 | 3.43E-50 | Pan-kidney |
| 79812 | MMRN2 | 1.937443128 | 5.26E-43 | Pan-kidney |
| 913 | CD1E | 1.938904937 | 8.91E-26 | Pan-kidney |
| 55784 | MCTP2 | 1.939033117 | 1.58E-27 | Pan-kidney |
| 64220 | STRA6 | 1.940287656 | 4.99E-19 | Pan-kidney |
| 100130776 | None | 1.941020364 | 1.64E-52 | Pan-kidney |
| 202309 | GAPT | 1.941711064 | 7.08E-39 | Pan-kidney |
| 57419 | SLC24A3 | 1.943695057 | 8.65E-24 | Pan-kidney |
| 283420 | CLEC9A | 1.944931067 | 4.38E-26 | Pan-kidney |
| 11185 | INMT | 1.94512586 | 4.74E-31 | Pan-kidney |
| 404550 | C16orf74 | 1.945462776 | 3.79E-23 | Pan-kidney |
| 59269 | HIVEP3 | 1.945698623 | 2.33E-78 | Pan-kidney |
| 84663 | None | 1.945926219 | 2.58E-13 | Pan-kidney |
| 4606 | MYBPC2 | 1.94666313 | 2.26E-23 | Pan-kidney |
| 6283 | S100A12 | 1.947396355 | 1.69E-30 | Pan-kidney |
| 146862 | UNC45B | 1.949771702 | 7.17E-47 | Pan-kidney |
| 8287 | USP9Y | 1.949872071 | 3.43E-08 | Pan-kidney |
| 3880 | KRT19 | 1.950104393 | 1.21E-08 | Pan-kidney |
| 51296 | SLC15A3 | 1.95092975 | 1.34E-63 | Pan-kidney |
| 9935 | MAFB | 1.95224067 | 3.29E-59 | Pan-kidney |
| 9022 | CLIC3 | 1.953503769 | 2.22E-22 | Pan-kidney |
| 10561 | IFI44 | 1.953966156 | 1.51E-63 | Pan-kidney |
| 79365 | BHLHE41 | 1.95476721 | 3.90E-32 | Pan-kidney |
| 6330 | SCN4B | 1.954816871 | 9.76E-27 | Pan-kidney |
| 55243 | KIRREL1 | 1.956271936 | 3.42E-31 | Pan-kidney |
| 27122 | DKK3 | 1.956598746 | 1.72E-36 | Pan-kidney |
| 1438 | CSF2RA | 1.95687057 | 2.40E-47 | Pan-kidney |
| 1734 | DIO2 | 1.958916476 | 8.48E-23 | Pan-kidney |
| 4922 | NTS | 1.960496402 | 2.57E-22 | Pan-kidney |
| 100133669 | None | 1.961754222 | 2.31E-26 | Pan-kidney |
| 9032 | TM4SF5 | 1.961952694 | 2.15E-12 | Pan-kidney |
| 727936 | GXYLT2 | 1.962732874 | 4.22E-24 | Pan-kidney |
| 57471 | ERMN | 1.964026085 | 2.66E-35 | Pan-kidney |
| 2022 | ENG | 1.964096708 | 2.62E-64 | Pan-kidney |
| 11248 | NXPH3 | 1.964339047 | 9.71E-36 | Pan-kidney |
[truncated: 90,835 more chars]
